# Supplementary material for: Spatial-temporal trends and risk factors for undernutrition and obesity among children (<5 years) in South Africa, 2008–2017: findings from a nationally representative longitudinal panel survey
Source: BMJ Open. 2020 Apr 8;10(4):e034476. doi: 10.1136/bmjopen-2019-034476 (PMC7245449; doi:10.1136/bmjopen-2019-034476)
Supplement: Supplementary data [file bmjopen-2019-034476supp001.pdf]

Supplementary Material

Supplementary 1: STROBE Statement—Checklist of items that should be included in reports of cross-sectional studies

	Item No	Recommendation	Page/line numbers
**Title and abstract**	1	(*a*) Indicate the study's design with a commonly used term in the title or the abstract	2/30
		(*b*) Provide in the abstract an informative and balanced summary of what was done and what was found	2/28-47
**Introduction**			
Background/rationale	2	Explain the scientific background and rationale for the investigation being reported	3-4/67-84
Objectives	3	State specific objectives, including any prespecified hypotheses	4/82-84
**Methods**			
Study design	4	Present key elements of study design early in the paper	4/89-100
Setting	5	Describe the setting, locations, and relevant dates, including periods of recruitment, exposure, follow-up, and data collection	4/89-100
Participants	6	(*a*) Give the eligibility criteria, and the sources and methods of selection of participants	4/95-102
Variables	7	Clearly define all outcomes, exposures, predictors, potential confounders, and effect modifiers. Give diagnostic criteria, if applicable	4-5/103-117
Data sources/ measurement	8*	For each variable of interest, give sources of data and details of methods of assessment (measurement). Describe comparability of assessment methods if there is more than one group	4/89-93; 5/113-117
Bias	9	Describe any efforts to address potential sources of bias	4/89-91; 8/200-2017
Study size	10	Explain how the study size was arrived at	4/89-91; Supplementary 10
Quantitative variables	11	Explain how quantitative variables were handled in the analyses. If applicable, describe which groupings were chosen and why	4-5/104-110; 5/113-117
Statistical methods	12	(*a*) Describe all statistical methods, including those used to control for confounding	5-7/118-189
		(*b*) Describe any methods used to examine subgroups and interactions	5/113-117; 7/186-189
		(*c*) Explain how missing data were addressed	8/204-207; Supplementary 7 & 8
		(*d*) If applicable, describe analytical methods taking account of sampling strategy	5/120-123; 7/172-174; 7/186-189

		(*e*) Describe any sensitivity analyses	8/204-205; Supplementary 4b, 8
**Results**			
Participants	13*	(a) Report numbers of individuals at each stage of study—eg numbers potentially eligible, examined for eligibility, confirmed eligible, included in the study, completing follow-up, and analysed	8/200-207; Supplementary 7
		(b) Give reasons for non-participation at each stage	8/200-207; Supplementary 7
		(c) Consider use of a flow diagram	Described using narrative text: 8/200-207; Table: Supplementary 7
Descriptive data	14*	(a) Give characteristics of study participants (eg demographic, clinical, social) and information on exposures and potential confounders	Table 1
		(b) Indicate number of participants with missing data for each variable of interest	8/200-207; Table 1; Supplementary 7
Outcome data	15*	Report numbers of outcome events or summary measures	Table 1-3
Main results	16	(*a*) Give unadjusted estimates and, if applicable, confounder-adjusted estimates and their precision (eg, 95% confidence interval). Make clear which confounders were adjusted for and why they were included	8/209-273; Table 1-3
		(*b*) Report category boundaries when continuous variables were categorized	4/104-110
		(*c*) If relevant, consider translating estimates of relative risk into absolute risk for a meaningful time period	N/A
Other analyses	17	Report other analyses done—eg analyses of subgroups and interactions, and sensitivity analyses	Supplementary 2,6,7,8,9,10
**Discussion**			
Key results	18	Summarise key results with reference to study objectives	10-11/275-283
Limitations	19	Discuss limitations of the study, taking into account sources of potential bias or imprecision. Discuss both direction and magnitude of any potential bias	14/377-386
Interpretation	20	Give a cautious overall interpretation of results considering objectives, limitations, multiplicity of analyses, results from similar studies, and other relevant evidence	11-15/296-417
Generalisability	21	Discuss the generalisability (external validity) of the study results	11-13/296-370
**Other information**			
Funding	22	Give the source of funding and the role of the funders for the present study and, if applicable, for the original study on which the present article is based	19/581-583

Supplementary 2: Spatial autocorrelation analyses for the 3 anthropometric outcomes (univariate and bivariate)

Pairwise correlation for anthropometric outcomes and bivariate spatial autocorrelation

We have performed additional supplementary analyses (suing GeoDa: Anselin L, Syabri I, Kho Y. GeoDa: an introduction to spatial data analysis. Geographical analysis. 2006 Jan;38(1):5-22) which assesses pairwise correlation/association between the 3 outcomes as well as bivariate Moran's I to assess if there was significant spatial autocorrelation between the outcomes. This analysis suggests that there is no significant association between stunting and thinness/wasting while there is weak positive but significant spatial autocorrelation between stunting and obesity prevalence as well as weak negative spatial correlation between thinness and obesity (please see detailed analyses below).

```
. spearman stunted_svy thin_svy

 Number of obs =     256
Spearman's rho =       0.0729

Test of Ho: stunted_svy and thin_svy are independent
    Prob > |t| =       0.2452

. gllamm stunted_svy thin_svy, i(id)

number of level 1 units = 256
number of level 2 units = 52

Condition Number = 14.594452

gllamm model

log likelihood = 283.93295

------------------------------------------------------------------------------
 stunted_svy |      Coef.   Std. Err.      z    P>|z|     [95% Conf. Interval]
-------------+----------------------------------------------------------------
    thin_svy |   .0385636   .0726234     0.53   0.595    -.1037757    .1809028
       _cons |   .1082981   .0061531    17.60   0.000     .0962381     .120358
------------------------------------------------------------------------------

Variance at level 1
------------------------------------------------------------------------------

  .00637033 (.00056306)

Variances and covariances of random effects
------------------------------------------------------------------------------

***level 2 (id)

    var(1): 2.643e-24 (5.133e-14)
------------------------------------------------------------------------------
```

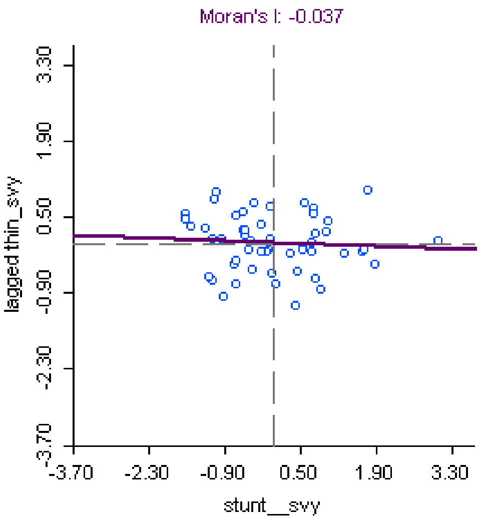

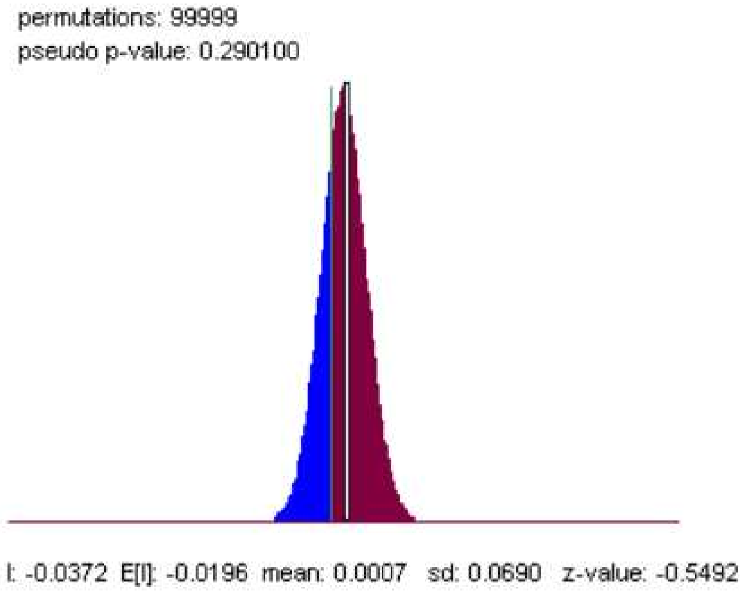


```
. spearman stunted_svy obese_svy

 Number of obs =     256
Spearman's rho =       0.2051

Test of Ho: stunted_svy and obese_svy are independent
    Prob > |t| =       0.0010

. gllamm stunted_svy obese_svy , i(id)

number of level 1 units = 256
number of level 2 units = 52

Condition Number = 10.565877

gllamm model

log likelihood = 292.58012

------------------------------------------------------------------------------
 stunted_svy |      Coef.   Std. Err.      z    P>|z|     [95% Conf. Interval]
-------------+----------------------------------------------------------------
```

```
   obese_svy |   .1980684   .0475478     4.17   0.000      .1048765     .2912604
       _cons |   .0791266   .0090305     8.76   0.000      .0614272     .0968261
------------------------------------------------------------------------------

Variance at level 1
------------------------------------------------------------------------------

  .00580379 (.00057983)

Variances and covariances of random effects
------------------------------------------------------------------------------

***level 2 (id)

    var(1): .00015837 (.00029997)
------------------------------------------------------------------------------
```

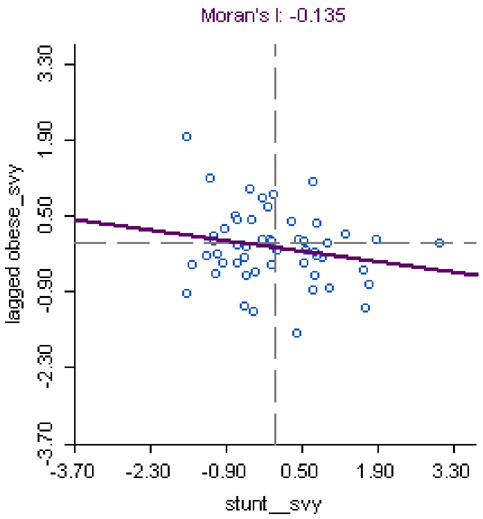

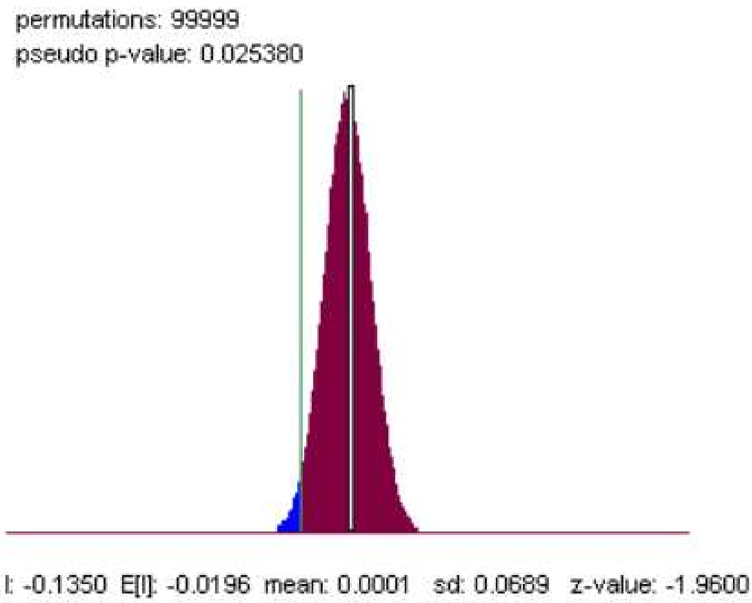


```
. spearman thin_svy obese_svy

 Number of obs =     256
Spearman's rho =      -0.1424

Test of Ho: thin_svy and obese_svy are independent
```

```
    Prob > |t| =       0.0227

. gllamm thin_svy obese_svy , i(id)

number of level 1 units = 256
number of level 2 units = 52

Condition Number = 10.976401

gllamm model

log likelihood = 324.36079

------------------------------------------------------------------------------
    thin_svy |      Coef.   Std. Err.      z    P>|z|     [95% Conf. Interval]
-------------+----------------------------------------------------------------
   obese_svy |   -.067802    .040258    -1.68   0.092    -.1467062    .0111022
       _cons |   .0602269   .0078037     7.72   0.000     .0449319    .0755218
------------------------------------------------------------------------------

Variance at level 1
------------------------------------------------------------------------------

  .00447574 (.00044278)

Variances and covariances of random effects
------------------------------------------------------------------------------

***level 2 (id)

    var(1): .00018259 (.00023176)
------------------------------------------------------------------------------
```

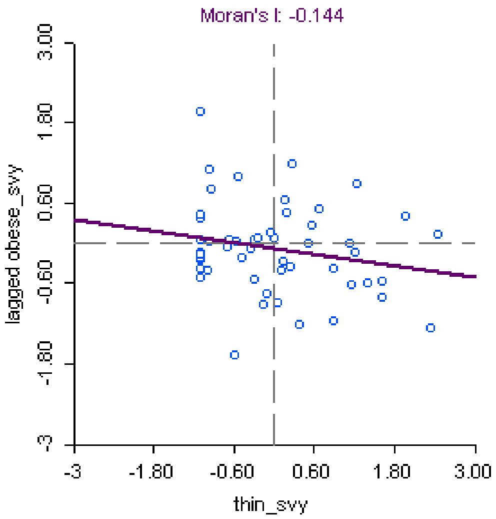

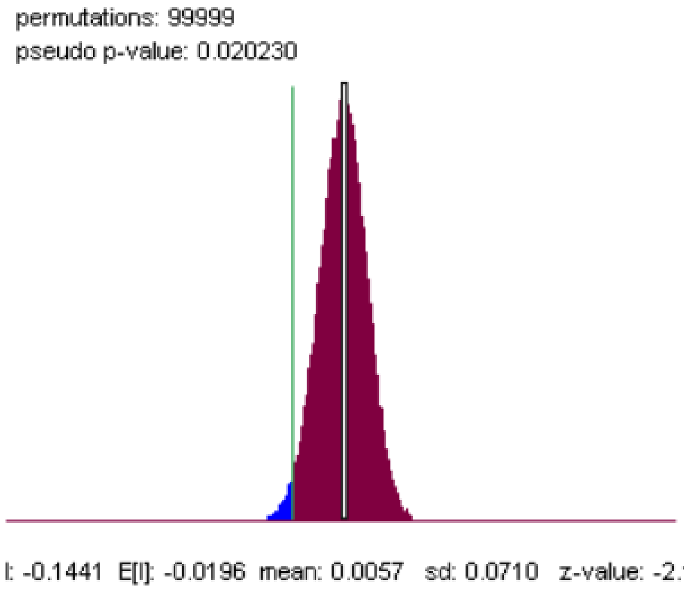

With regards to the shared temporal effect this we think can be retained as all 3 outcomes appear to have a negative coefficient associated with increasing panel or wave.

```
. gllamm stunted_svy year , i(id)

number of level 1 units = 256
number of level 2 units = 52

Condition Number = 31.724715

gllamm model

log likelihood = 293.64743

------------------------------------------------------------------------------
 stunted_svy |      Coef.   Std. Err.      z    P>|z|     [95% Conf. Interval]
-------------+----------------------------------------------------------------
        year |  -.0153423   .0033894    -4.53   0.000    -.0219855   -.0086992
       _cons |   .1563577   .0112694    13.87   0.000     .1342702    .1784453
------------------------------------------------------------------------------

Variance at level 1
------------------------------------------------------------------------------

  .00590475 (.00052191)

Variances and covariances of random effects
------------------------------------------------------------------------------


***level 2 (id)

    var(1): 8.887e-19 (4.854e-11)
------------------------------------------------------------------------------

. gllamm thin_svy year , i(id)

number of level 1 units = 256
number of level 2 units = 52

Condition Number = 37.175479

gllamm model

log likelihood = 327.11892

------------------------------------------------------------------------------
```

```
    thin_svy |      Coef.   Std. Err.      z    P>|z|     [95% Conf. Interval]
-------------+----------------------------------------------------------------
        year |  -.0084373   .0028941    -2.92   0.004    -.0141096    -.002765
       _cons |   .0749857   .0098979     7.58   0.000     .0555862    .0943852
------------------------------------------------------------------------------

Variance at level 1
------------------------------------------------------------------------------

  .00430301 (.00042507)

Variances and covariances of random effects
------------------------------------------------------------------------------


***level 2 (id)

    var(1): .00027197 (.0002388)
------------------------------------------------------------------------------

. gllamm obese_svy year , i(id)

number of level 1 units = 256
number of level 2 units = 52

Condition Number = 21.597249

gllamm model

log likelihood = 215.4003

------------------------------------------------------------------------------
   obese_svy |      Coef.   Std. Err.      z    P>|z|     [95% Conf. Interval]
-------------+----------------------------------------------------------------
        year |  -.0112194   .0043125    -2.60   0.009    -.0196717   -.0027671
       _cons |   .1905201   .0155017    12.29   0.000     .1601374    .2209029
------------------------------------------------------------------------------

Variance at level 1
------------------------------------------------------------------------------

  .00954712 (.00094327)

Variances and covariances of random effects
------------------------------------------------------------------------------

***level 2 (id)

    var(1): .00175973 (.00074487)
------------------------------------------------------------------------------
```

Univariate spatial autocorrelation

Based on the univariate Moran’s I statistics for each anthropometric outcome there appeared to be significant spatial heterogeneity present for all 3 outcomes.

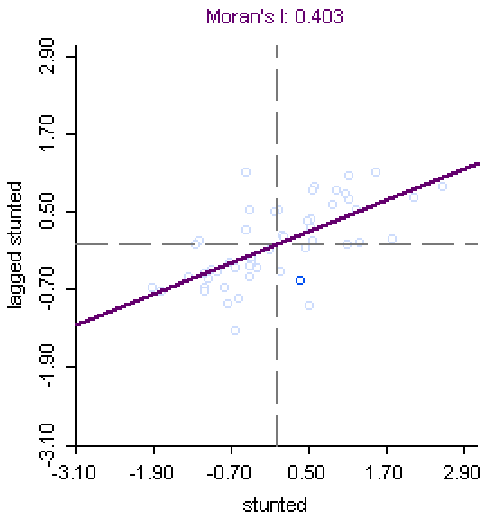
Moran's I: 0.403
lagged stunted
stunted
2.90
1.70
0.50
-0.70
-1.90
-3.10
-3.10 -1.90 -0.70 0.50 1.70 2.90

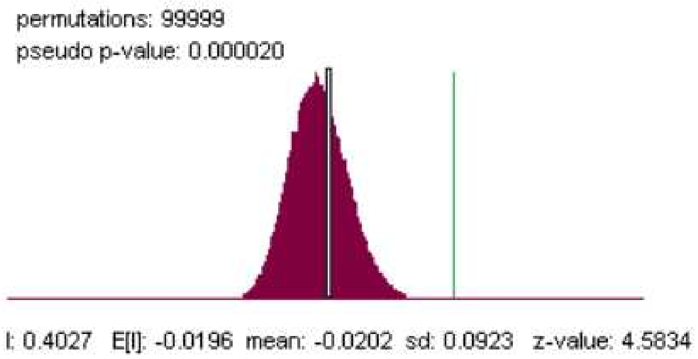
permutations: 99999
pseudo p-value: 0.000020
I: 0.4027 E[I]: -0.0196 mean: -0.0202 sd: 0.0923 z-value: 4.5834

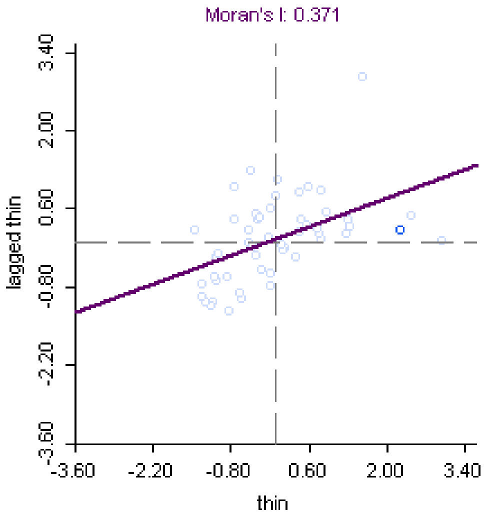
Moran's I: 0.371
lagged thin
thin
3.40
2.00
0.60
-0.80
-2.20
-3.60
-3.60 -2.20 -0.80 0.60 2.00 3.40

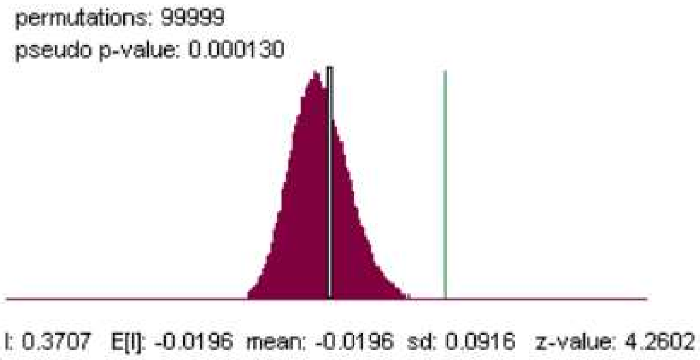
permutations: 99999
pseudo p-value: 0.000130
I: 0.3707 E[I]: -0.0196 mean: -0.0196 sd: 0.0916 z-value: 4.2602

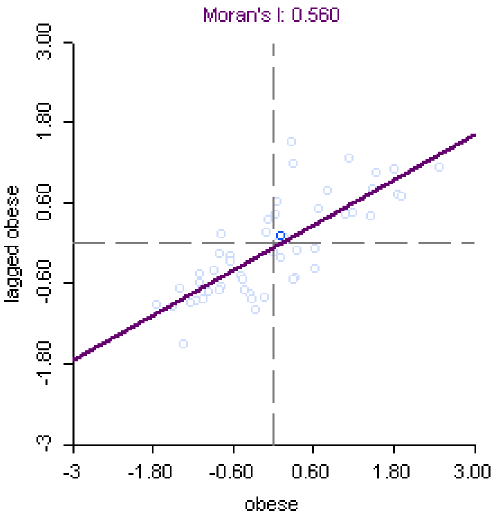
Moran's I: 0.560
lagged obese
obese
-3
-1.80
-0.60
0.60
1.80
3.00

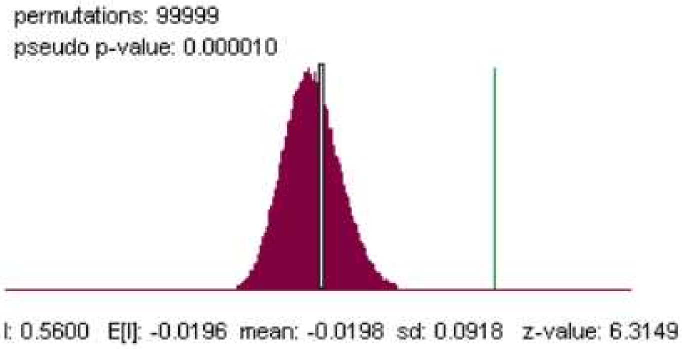
permutations: 99999
pseudo p-value: 0.000010
I: 0.5600 E[I]: -0.0196 mean: -0.0198 sd: 0.0918 z-value: 6.3149

Supplementary 3: Win BUGS code for Bayesian space-time binomial model

```
model
{
	for( i in 1 : N ) {
		for( j in 1 : T ) {
		#Likelihood

		stunted[i,j] ~ dbin(p1[i,j],child[i,j])
		logit(p1[i,j])<-alpha1+phi1[i]+gamma1[j]+nu1[i,j]

		thin[i,j] ~ dbin(p2[i,j],child[i,j])
		logit(p2[i,j])<-alpha2+phi2[i]+gamma2[j]+nu2[i,j]
		exceedance2[i,j]<-step(p2[i,j]-0.05)	# reduce and maintain wasting to <5%

		obese[i,j] ~ dbin(p3[i,j],child[i,j])
		logit(p3[i,j])<-alpha3+phi3[i]+gamma3[j]+nu3[i,j]
		}
	exceedance1[i,5]<-step((1-p1[i,5]/p1[i,3])-0.17) #17% is target reduction by 2017 from 2012
								# assuming target 40% reduction by 2025
	exceedance3[i,5]<-step(p3[i,5]/p3[i,3]-1)		# no increase in obesity from 2012 to 2017
	}

# - Space
phi1[1:52]~car.normal(adj[],weights[],num[],tau.phi[1])
phi2[1:52]~car.normal(adj[],weights[],num[],tau.phi[2])
phi3[1:52]~car.normal(adj[],weights[],num[],tau.phi[3])

for(k in 1:240) {weights[k]<-1}

# - Time:

gamma1[1:T]~car.normal(adj.t[],weights.t[],num.t[],tau.gamma[1])
gamma2[1:T]~car.normal(adj.t[],weights.t[],num.t[],tau.gamma[2])
gamma3[1:T]~car.normal(adj.t[],weights.t[],num.t[],tau.gamma[3])

for(t in 1:1) {
	weights.t[t] <- 1;
	adj.t[t] <- t+1;
	num.t[t] <- 1
	}
for(t in 2:(T-1)) {
	weights.t[2+(t-2)*2] <- 1;
	adj.t[2+(t-2)*2] <- t-1
	weights.t[3+(t-2)*2] <- 1;
	adj.t[3+(t-2)*2] <- t+1;
	num.t[t] <- 2
	}
for(t in T:T) {
	weights.t[(T-2)*2 + 2] <- 1;
	adj.t[(T-2)*2 + 2] <- t-1;
	num.t[t] <- 1
	}

#Space-time Interaction terms
for(i in 1:N){
	for(j in 1:T){
	nu1[i,j]~dnorm(0, tau.nu[1])
	nu2[i,j]~dnorm(0, tau.nu[2])
	nu3[i,j]~dnorm(0, tau.nu[3])
```

```
        }
}

#Hyperprior specification

for(i in 1:3){
tau.phi[i]~dgamma(0.5, 0.0005)
tau.gamma[i]~dgamma(0.5, 0.0005)
tau.nu[i]~dgamma(0.5, 0.0005)
}

alpha1~dflat()
alpha2~dflat()
alpha3~dflat()

}
}
```

Supplementary 4: a) Model random effects posteriors and b) sensitivity analysis of hyper parameter selection

a)

Spatial random effects (phi)

Stunting

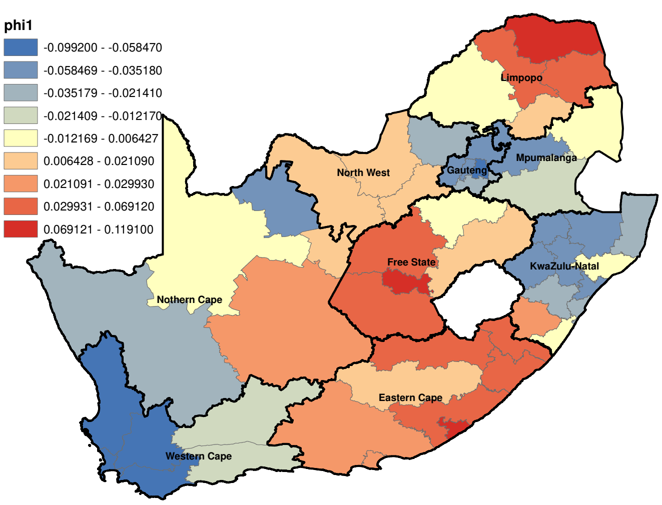

Thinness/wasting

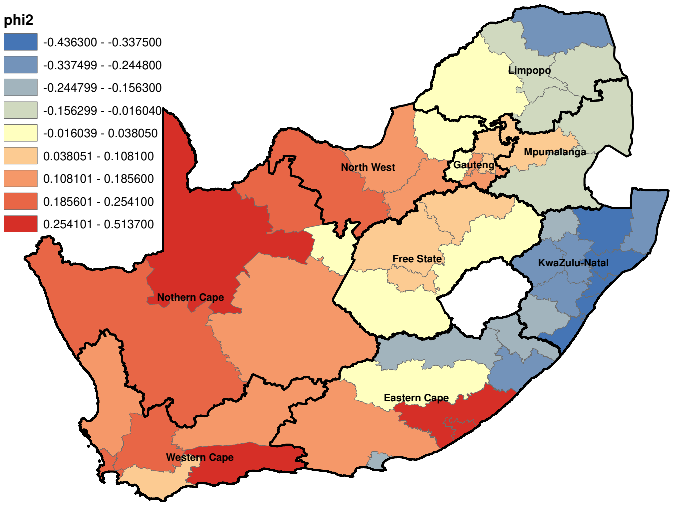

Obesity

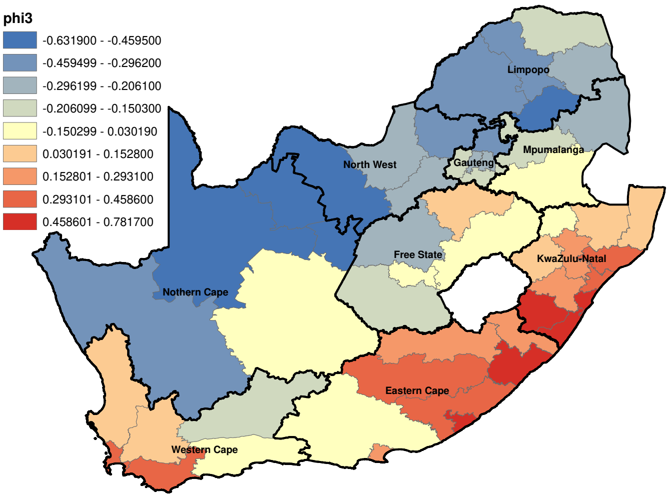

Unstructured effects (2017) (nu)

Stunting

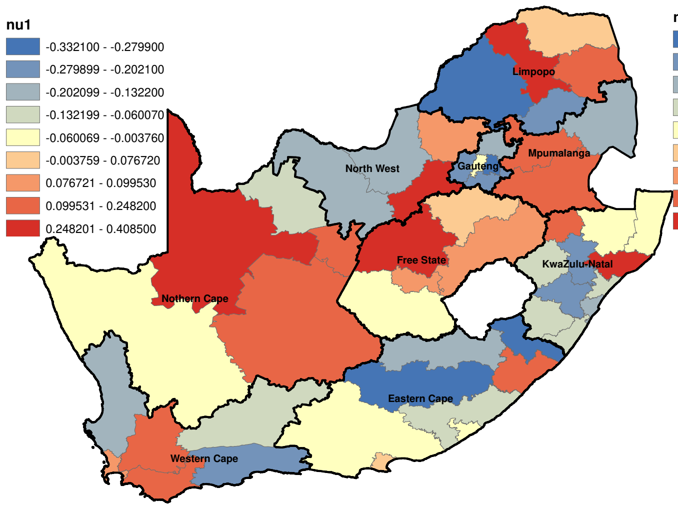

Thinness/wasting

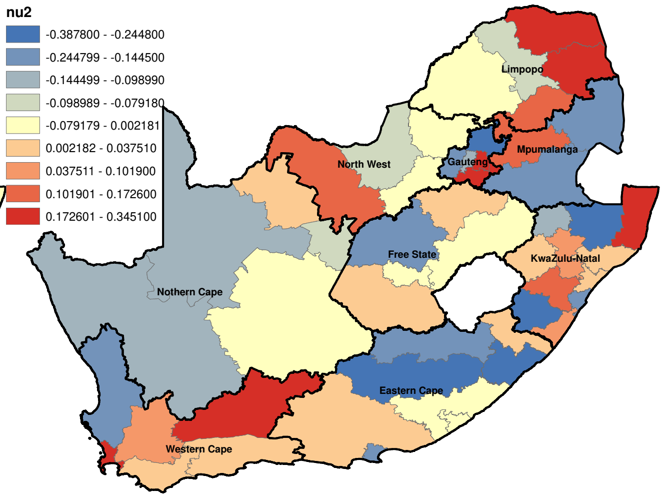

Obesity

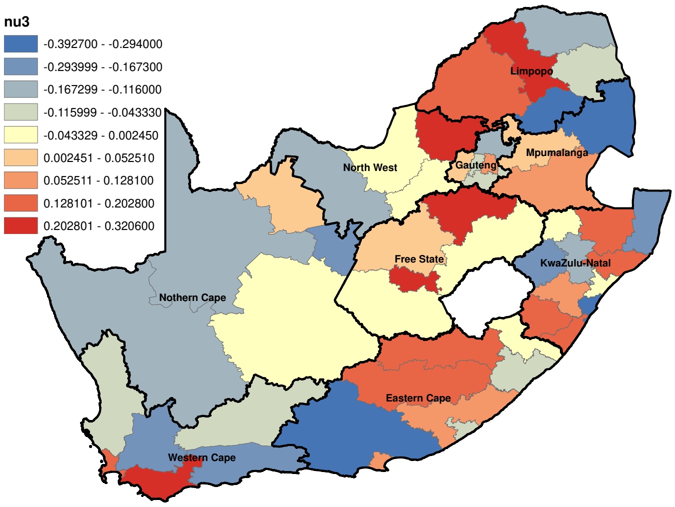

Temporal random effects (gamma)

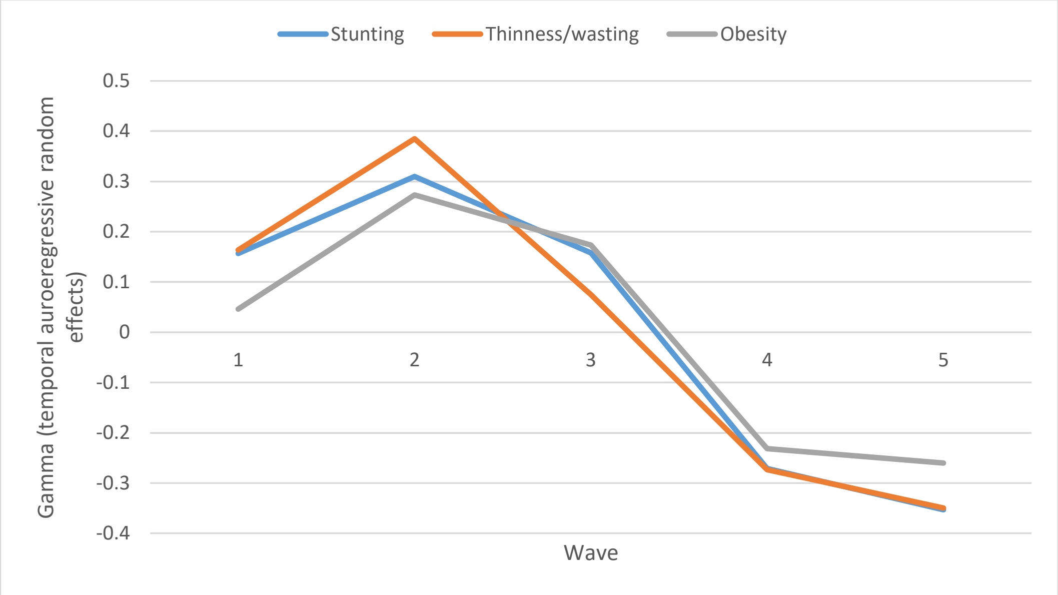

b)

We concluded an additional sensitivity analysis to confirm whether the choice of hyper parameter may have affected the prevalence estimates. For the variance parameters, namely σ^2_ν, σ^2_ϕ, σ^2_γ we assumed Gamma(0.5,0.0005) distributions as recommended by Wakefield (Wakefield J, Best N, Waller L. Bayesian approaches to disease mapping. Spatial epidemiology: methods and applications 2000:104-07.) for the Baysian prevalence/exceedance probability estimates presented in the main text. We also tested whether changes to this prior may have affected the estimates. Other choices for this prior (Lawson A, Browne W, Vidal Rodeiro C. Disease Mapping with WinBUGS and MLWin. Chichester: John Wiley & Sons; 2003) that are commonly used include.

Gamma (0.001, 0.001)

Gamma (0.01,0.01)

Pairwise scatterplots of the posterior prevalence for the various gamma distribution choices for the hyper parameters below suggest that the model estimates were largely insensitive to the choice of distribution assumed:

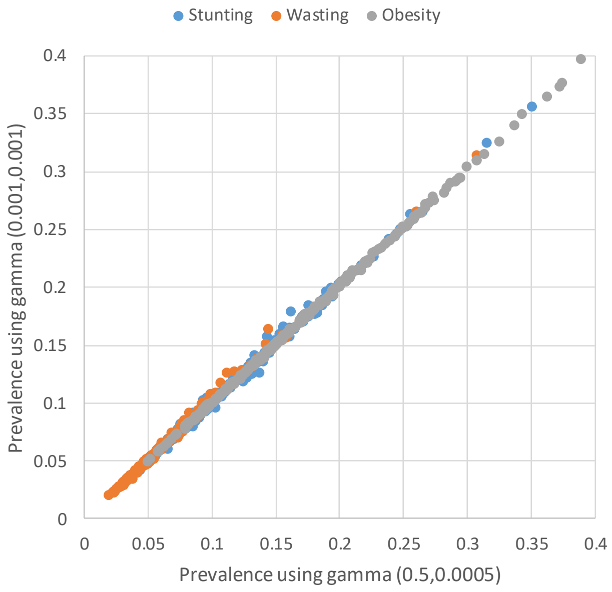
Stunting
Wasting
Obesity
Prevalence using gamma (0.001,0.001)
Prevalence using gamma (0.5,0.0005)
0
0.05
0.1
0.15
0.2
0.25
0.3
0.35
0.4

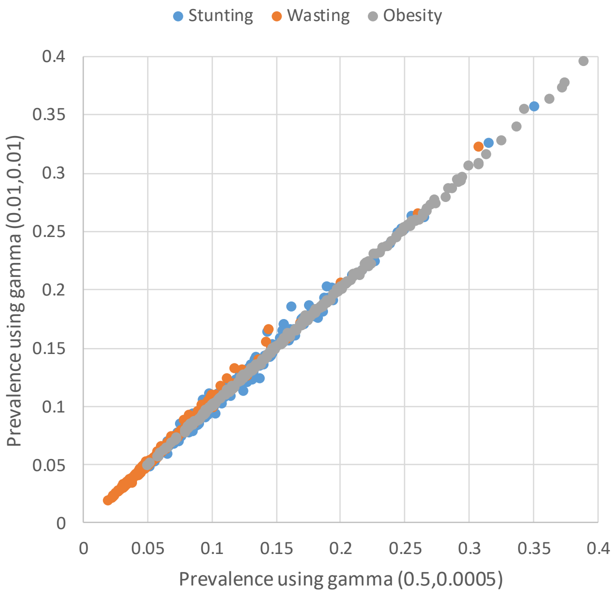
Stunting
Wasting
Obesity
Prevalence using gamma (0.01,0.01)
Prevalence using gamma (0.5,0.0005)
0
0.05
0.1
0.15
0.2
0.25
0.3
0.35
0.4

Supplementary 5: a) Model convergence [Gelman-Rubin statistics/plots]

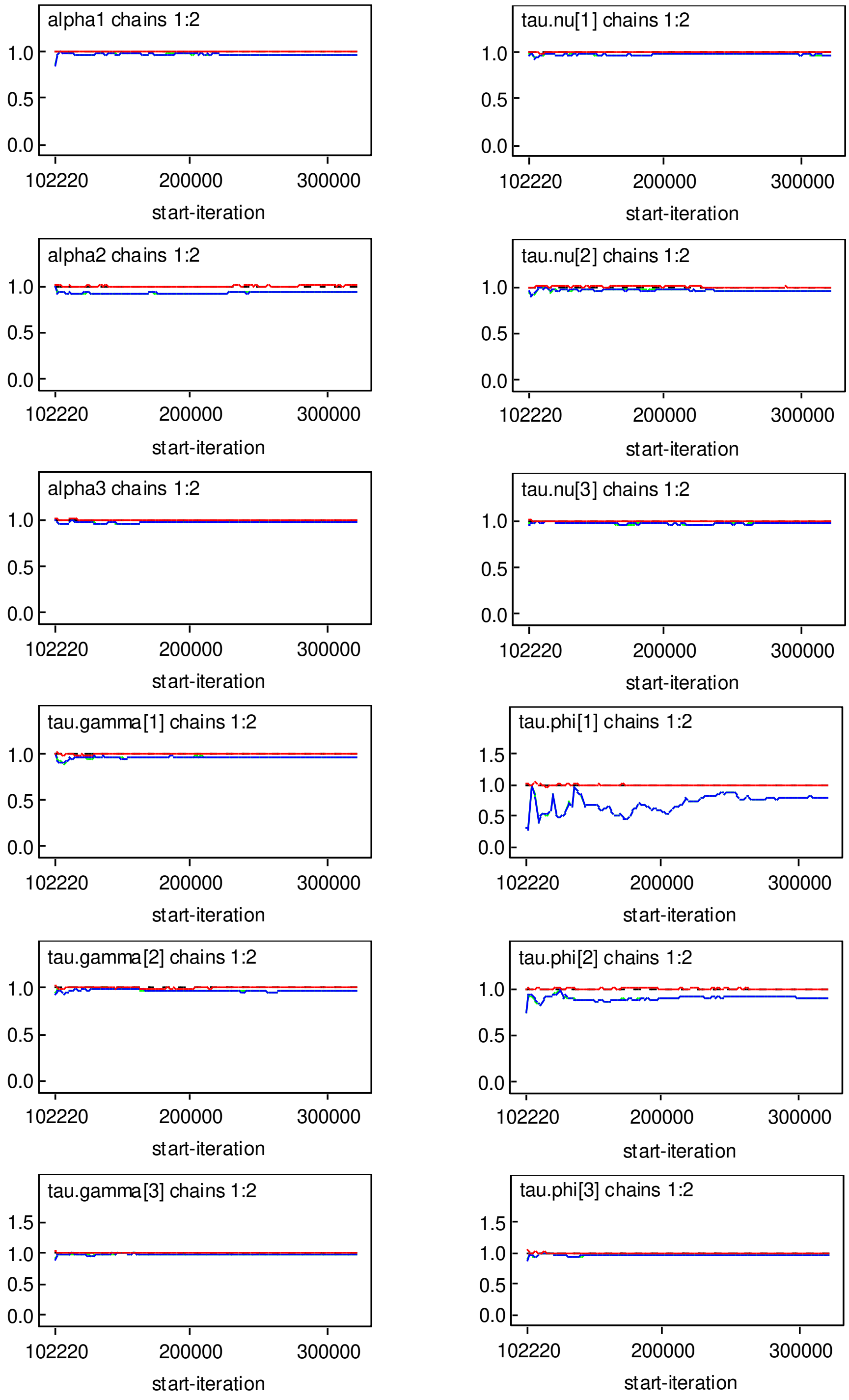

Supplementary 6: Model fit and out of sample validation

Overall model fit

Dbar = post.mean of -2logL; Dhat = -2LogL at post.mean of stochastic nodes

	Dbar	Dhat	pD	DIC
obese	1110.400	969.250	141.149	1251.550
stunted	1036.090	910.101	125.987	1162.080
thin	695.343	602.042	93.301	788.643
total	2841.830	2481.390	360.437	3202.270

Comparison of survey prevalence versus model fitted prevalence by anthropometric measure

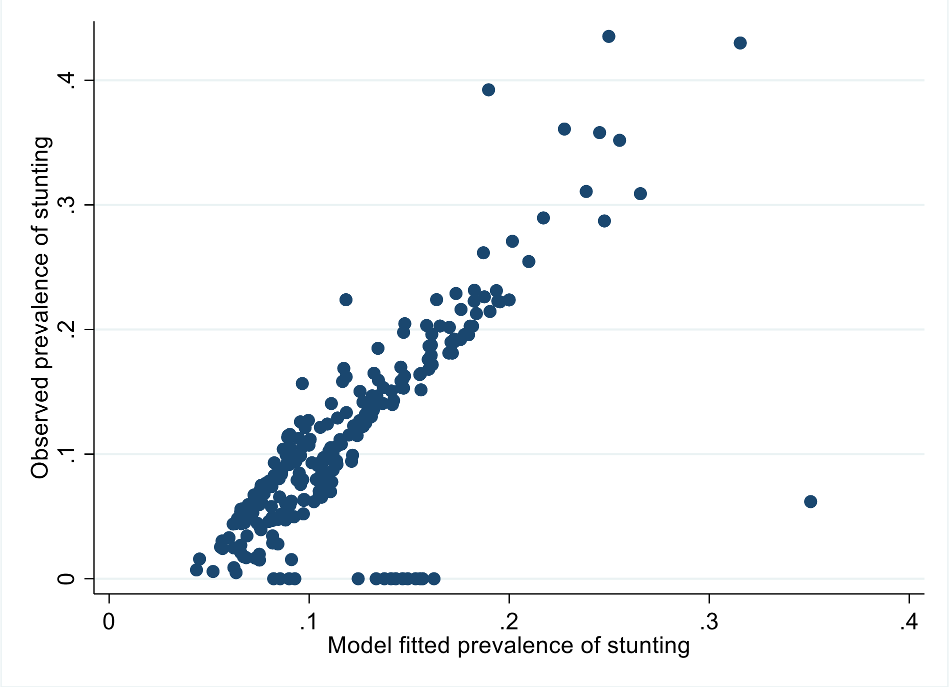


```
. spearman stunted_svy p1 if stunted_svy~=0

 Number of obs =      241
Spearman's rho =       0.9190

Test of Ho: stunted_svy and p1 are independent
    Prob > |t| =       0.0000

. spearman stunted_svy p1

 Number of obs =      256
Spearman's rho =       0.7729

Test of Ho: stunted_svy and p1 are independent
    Prob > |t| =       0.0000
```

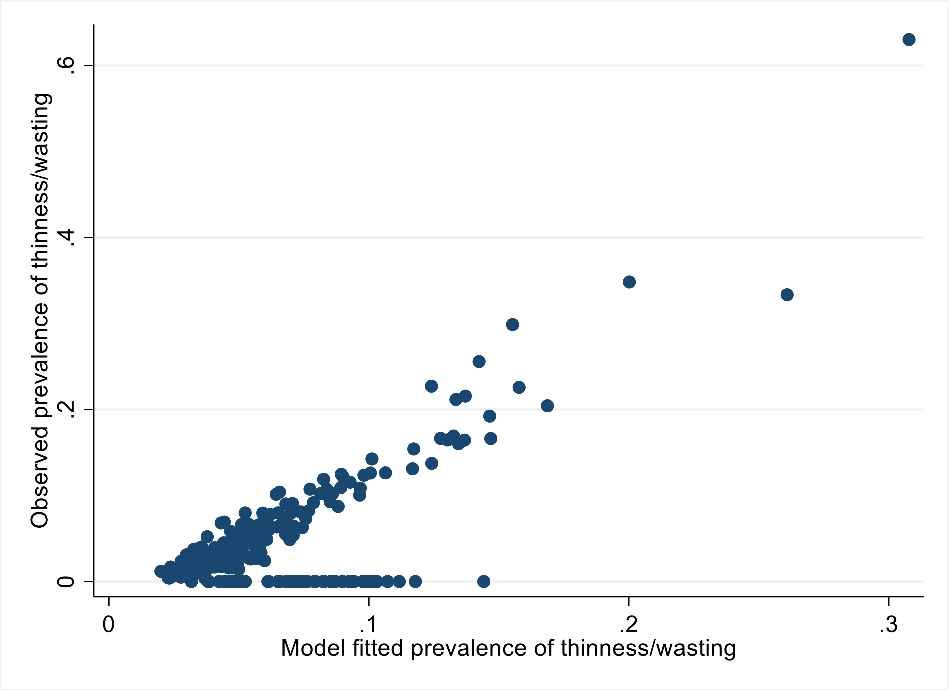


```
. spearman thin_svy p2 if thin_svy~=0

 Number of obs =      191
Spearman's rho =       0.9019

Test of Ho: thin_svy and p2 are independent
    Prob > |t| =       0.0000

. spearman thin_svy p2

 Number of obs =      256
Spearman's rho =       0.2972

Test of Ho: thin_svy and p2 are independent
    Prob > |t| =       0.0000
```

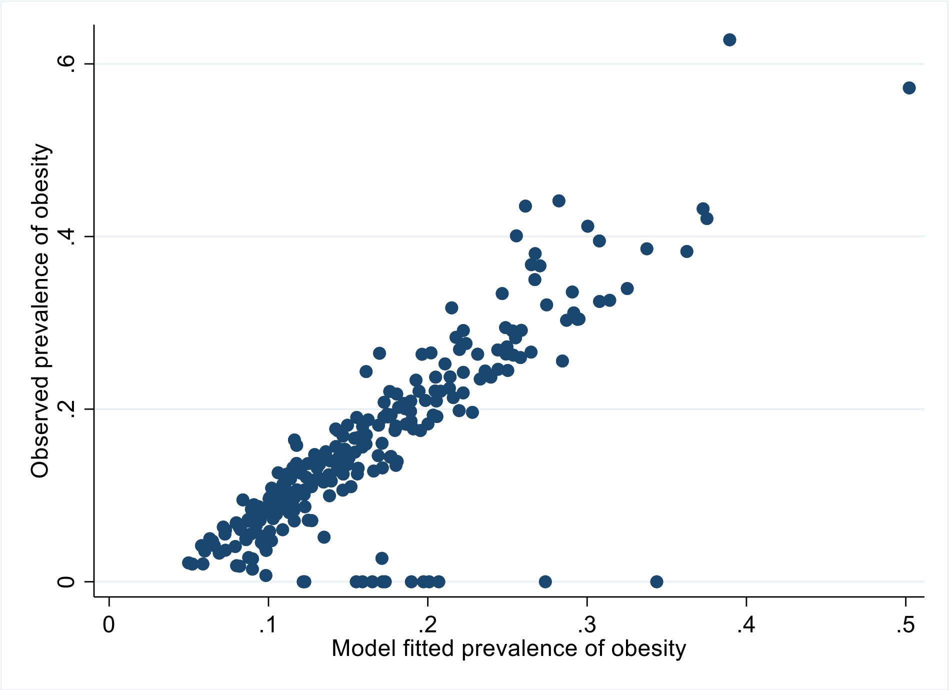


```
. spearman obese_svy p3 if obese_svy~=0

 Number of obs =      243
Spearman's rho =        0.9485

Test of Ho: obese_svy and p3 are independent
    Prob > |t| =        0.0000

. spearman obese_svy p3

 Number of obs =      256
Spearman's rho =        0.8179

Test of Ho: obese_svy and p3 are independent
    Prob > |t| =        0.0000
```

Out of sample validation/prediction (10% random sample)

Of the 37 out of sample validation points, 31 (or 84%) of the observed prevalence of stunting where within the 95% uncertainty interval for the predicted posterior prevalence, 28/37 (78%) for thinness/wasting and 31/37 (84%) for obesity.

id	wave	District	Prov	Stunted (observed)	Thin/wasted (observed)	Obese (observed)	Stunted (posterior)	95% BCI		Thin/ wasted (posterior)	95% BCI		Obese (posterior)	95% BCI	
3	4	City of Tshwane (TSH)	Gauteng	0.01584	0.028309	0.03565	0.09138	0.0344	0.1911	0.05339	0.01411	0.1343	0.1104	0.04251	0.226
4	1	City of Johannesburg (JHB)	Gauteng	0.075611	0.015782	0.187624	0.1285	0.05006	0.26	0.08426	0.0217	0.2094	0.1389	0.05482	0.2789
5	1	Buffalo City (BUF)	Eastern Cape	0.392359	0.255737	0.255737	0.1366	0.04961	0.2864	0.105	0.02015	0.3356	0.3173	0.1231	0.5788
8	3	Cacadu (DC10)	Eastern Cape	0.063566	0.063563	0.071285	0.1308	0.05085	0.261	0.07077	0.01873	0.1759	0.1926	0.07914	0.3611
9	5	Amathole (DC12)	Eastern Cape	0.034391	0.048998	0.263574	0.08987	0.03286	0.1895	0.0618	0.01346	0.1731	0.1882	0.07342	0.363
12	1	O.R.Tambo (DC15)	Eastern Cape	0.222196	0.015888	0.246151	0.138	0.05288	0.2758	0.06349	0.01635	0.1665	0.2552	0.111	0.4522
13	3	Xhariep (DC16)	Free State	0.052021	0.022786	0.086915	0.1352	0.0517	0.2722	0.06617	0.01758	0.1673	0.1631	0.06518	0.3172
14	4	Lejweleputswa (DC18)	Free State	0.164856	0.059159	0.08698	0.09708	0.0363	0.2027	0.0539	0.01409	0.1363	0.1095	0.04191	0.2254
15	3	Thabo Mofutsanyane (DC19)	Free State	0.228885	0.030611	0.106143	0.1284	0.04956	0.2567	0.06452	0.0173	0.1614	0.1861	0.07662	0.353

16	5	Cape Winelands (DC2)	Western Cape	0.125904	0.065507	0.089544	0.08173	0.02978	0.176	0.05496	0.01371	0.1417	0.1369	0.05382	0.2708
19	3	UMgungundlovu (DC22)	KwaZulu-Natal	0.133616	0.107423	0.213388	0.1224	0.0468	0.2492	0.05064	0.01301	0.136	0.2251	0.0955	0.4095
20	3	Uthukela (DC23)	KwaZulu-Natal	0.140663	0.029259	0.244155	0.1202	0.04537	0.2464	0.05216	0.01372	0.1382	0.1857	0.07645	0.3524
21	5	Umzinyathi (DC24)	KwaZulu-Natal	0.045311	0.037798	0.117828	0.08302	0.03081	0.1783	0.03554	0.008943	0.09847	0.1524	0.05855	0.3055
23	4	Zululand (DC26)	KwaZulu-Natal	0.082797	0.026059	0.193396	0.08843	0.03282	0.1885	0.03922	0.009809	0.1077	0.1404	0.0551	0.2804
25	3	Uthungulu (DC28)	KwaZulu-Natal	0.178442	0.022595	0.239129	0.1247	0.04783	0.251	0.0497	0.01282	0.1345	0.2337	0.09804	0.4257
26	3	iLembe (DC29)	KwaZulu-Natal	0.196082	0.045007	0.198271	0.1188	0.04489	0.2454	0.04939	0.01221	0.1343	0.2598	0.1132	0.4611
27	4	Overberg (DC3)	Western Cape	0.044303	0.006543	0.22102	0.08827	0.03194	0.1892	0.05854	0.01459	0.1527	0.1828	0.07273	0.3521
28	1	Gert Sibande (DC30)	Mpumalanga	0.039364	0.016922	0.087611	0.1301	0.05057	0.2615	0.06794	0.0188	0.1708	0.1692	0.06952	0.3252
28	3	Gert Sibande (DC30)	Mpumalanga	0.13142	0.169157	0.242636	0.126	0.04846	0.252	0.05976	0.01619	0.1517	0.1798	0.07408	0.3441
30	2	Ehlanzeni (DC32)	Mpumalanga	0.082747	0.049337	0.118953	0.1462	0.05729	0.2896	0.07806	0.02038	0.2002	0.1458	0.05572	0.2908
30	3	Ehlanzeni (DC32)	Mpumalanga	0.308996	0.102135	0.263661	0.1244	0.04733	0.252	0.05775	0.01508	0.1496	0.1336	0.05113	0.2685
32	4	Vhembe (DC34)	Limpopo	0.159305	0.005527	0.060438	0.1034	0.03696	0.2201	0.04615	0.01114	0.1254	0.127	0.04623	0.2623
33	4	Capricorn (DC35)	Limpopo	0.098606	0.024444	0.137144	0.09882	0.0363	0.2066	0.05008	0.0131	0.1289	0.09301	0.0339	0.198
35	1	Bojanala (DC37)	North West	0.061806	0.026407	0.041916	0.1308	0.05145	0.2631	0.07596	0.01993	0.1909	0.1472	0.05754	0.2922
35	3	Bojanala (DC37)	North West	0.051943	0.050775	0.07316	0.127	0.0494	0.2549	0.06689	0.01764	0.1705	0.1567	0.06121	0.3078
36	4	Ngaka Modiri Molema (DC38)	North West	0.098734	0.085696	0.112994	0.09498	0.03531	0.1977	0.05315	0.01375	0.1366	0.1163	0.04431	0.2389
37	4	Dr Ruth Segomotsi Mompati (DC39)	North West	0.074933	0.081794	0.020508	0.09534	0.03553	0.199	0.05775	0.01489	0.1462	0.09674	0.03614	0.2034
40	1	Sedibeng (DC42)	Gauteng	0.202795	0.225723	0.106432	0.1281	0.04987	0.26	0.07473	0.02019	0.1852	0.1554	0.06286	0.3048
40	2	Sedibeng (DC42)	Gauteng	0.152953	0.100409	0.132566	0.1455	0.05777	0.2879	0.08836	0.02332	0.2164	0.1801	0.07327	0.3438
41	4	Sisonke (DC43)	KwaZulu-Natal	0.058274	0.010655	0.058335	0.09652	0.03541	0.2031	0.04685	0.01187	0.123	0.2282	0.09509	0.4207
42	5	Alfred Nzo (DC44)	Eastern Cape	0.020967	0.040195	0.143771	0.09156	0.03377	0.1927	0.03907	0.009915	0.1059	0.1643	0.06519	0.3221
45	1	West Rand (DC48)	Gauteng	0.085192	0.124663	0.07049	0.1293	0.05015	0.2589	0.07178	0.01918	0.1799	0.1645	0.06532	0.3235
46	5	Central Karoo (DC5)	Western Cape	0.059525	0.10142	0.080496	0.08649	0.0322	0.1821	0.05009	0.01282	0.1309	0.1124	0.04168	0.2323
51	1	Ekurhuleni (EKU)	Gauteng	0.146754	0.026242	0.026557	0.1237	0.04661	0.2538	0.07674	0.02017	0.1912	0.1587	0.06215	0.3162
51	4	Ekurhuleni (EKU)	Gauteng	0.007073	0.079849	0.08599	0.08755	0.03179	0.1863	0.05287	0.01372	0.1358	0.1248	0.0475	0.2561
52	4	eThekwini (ETH)	KwaZulu-Natal	0.141696	0.011844	0.311557	0.08787	0.03228	0.1884	0.03885	0.009519	0.1088	0.196	0.07941	0.3735
52	5	eThekwini (ETH)	KwaZulu-Natal	0.055993	0.016832	0.124759	0.08245	0.03015	0.1764	0.0343	0.008309	0.09693	0.1901	0.07589	0.3647

```
. spearman stunted_svy stuntedvpost if validation_sample2==1

 Number of obs =      37
Spearman's rho =       0.4445

Test of Ho: stunted_svy and stuntedvpost are independent
    Prob > |t| =       0.0058
```

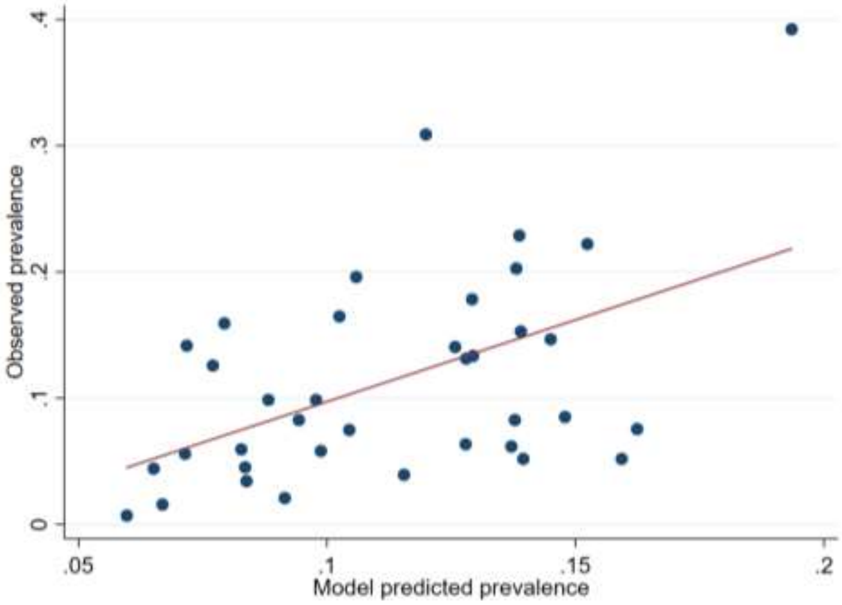


```
. spearman thin_svy thinvpost if validation_sample2==1

 Number of obs =      37
Spearman's rho =       0.2048

Test of Ho: thin_svy and thinvpost are independent
    Prob > |t| =       0.2239
```

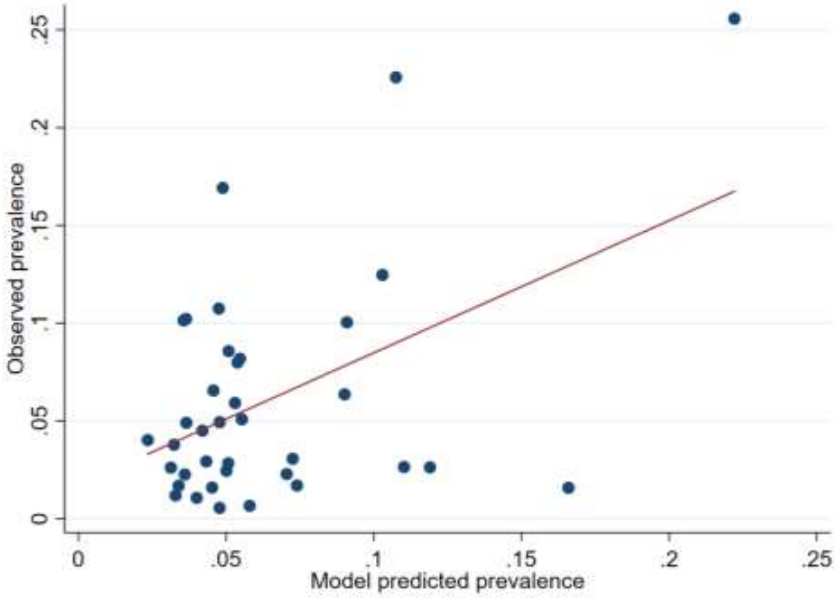


```
. spearman thin_svy thinvpost if validation_sample2==1 & thin_svy~=0

 Number of obs =      37
Spearman's rho =       0.2048

Test of Ho: thin_svy and thinvpost are independent
    Prob > |t| =       0.2239
```

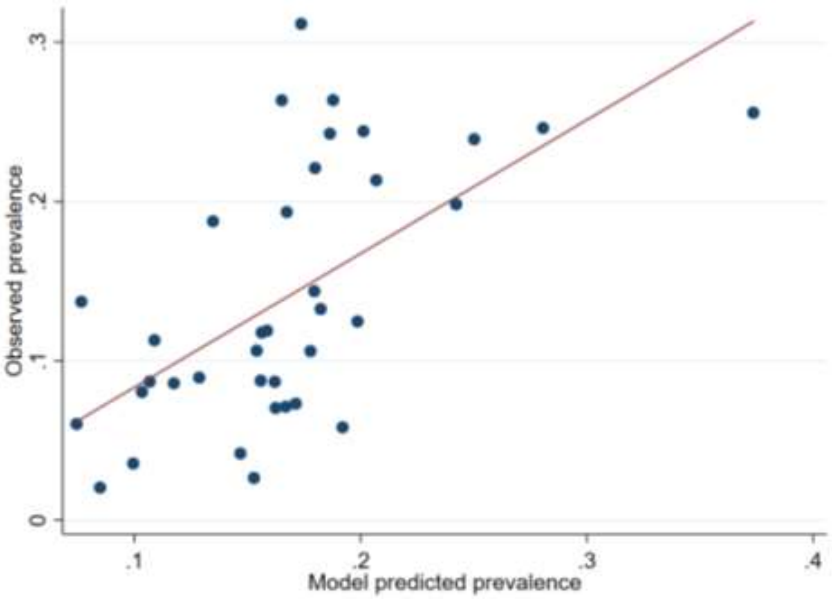
Observed prevalence
0
.1
.2
.3
.1
.2
.3
.4
Model predicted prevalence

Supplementary 7: Description of the study sample across survey rounds

Survey wave	Age (in years)	Sampled	Estimated population size using survey weights	95% CI		% sampled with height/weight measurement
**2008**	0	661	1092027	948199	1235854	35.9%
	1	661	1151665	1009086	1294244	67.9%
	2	670	1088458	960285	1216632	71.0%
	3	642	1034244	902011	1166477	81.0%
	4	620	1016227	882185	1150270	83.5%
	<5	3254	5382621	5005478	5759764	
**2010/11**	0	517	866786	720440	1013132	16.2%
	1	621	1032184	840129	1224239	42.5%
	2	751	1225419	1040085	1410753	49.3%
	3	840	1206389	1026681	1386097	53.3%
	4	820	1196800	1031500	1362101	53.3%
	<5	3549	5527578	4914106	6141050	
**2012**	0	652	902357	777704	1027010	45.1%
	1	691	1039354	887868	1190839	87.7%
	2	764	1183609	995508	1371711	87.6%
	3	826	1257820	1036042	1479598	89.6%
	4	909	1405034	1191438	1618631	87.3%
	<5	3842	5788174	5112765	6463583	
**2014/15**	0	886	1185863	1003941	1367786	50.3%
	1	875	1162949	985828	1340070	92.9%
	2	863	1060232	901257	1219207	92.7%
	3	914	1160946	985127	1336765	94.0%
	4	960	1298110	1098342	1497879	94.3%
	<5	4498	5868101	5200170	6536031	
**2017**	0	813	987763	841487	1134040	47.8%
	1	909	1215360	1045099	1385622	86.4%
	2	996	1293408	1105038	1481779	84.6%
	3	992	1264427	1088783	1440071	88.9%
	4	1000	1129184	973431	1284937	90.4%
	<5	4710	5890142	5261158	6519126	

Supplementary 8: Sensitivity analyses for missing weight and height

Summary: A comparison of missing weight/height proportions by various socio-demographic variables suggests that many were likely missing at random. Distributions of race, gender, household income, low birthweight, food security status, mother education category and father education category were not significantly different when comparing children with missing weight/height measurements to those with a valid weight/height measurement (please see analysis output below). However, age did significantly differ by missing status in that infants (<1 year of age) were significantly more likely to have a missing weight/height measurement compared to children aged 1-4 years. There also appeared to be significant differences in missing weight/height status by province of residence i.e. children in Mpumalanga, Western Cape fir example had higher proportions of missing weight/height measurements among children under 5 (p<0.001). Furthermore, missing weight/height measurements for children were more significantly more likely among those children with younger mothers (<25 years of age).

```
. svy: tab race_ missing_height_weight if race_~=0, row ci
(running tabulate on estimation sample)

Number of strata   =        53                 Number of obs     =      16,649
Number of PSUs     =     1,076                 Population size   =  25,331,414
                                               Design df         =       1,023

-------------------------------------------------------
          |           missing_height_weight
    race_ |            0             1         Total
----------+--------------------------------------------
  African |        .8129         .1871             1
          | [.8006,.8246]  [.1754,.1994]
          |
 Coloured |        .7803         .2197             1
          | [.7437,.8129]  [.1871,.2563]
          |
 Asian/In |        .7593         .2407             1
          |  [.5708,.882]   [.118,.4292]
          |
    White |          .74           .26             1
          |  [.643,.8182]   [.1818,.357]
          |
    Total |        .8066         .1934             1
          | [.7945,.8181]  [.1819,.2055]
-------------------------------------------------------
  Key:  row proportion
        [95% confidence interval for row proportion]

  Pearson:
    Uncorrected   chi2(3)         =   32.5162
    Design-based  F(2.49, 2551.53)=    1.7810     P = 0.1588

. svy: tab gender_ missing_height_weight if race_~=0, row ci
(running tabulate on estimation sample)

Number of strata   =        53                 Number of obs     =      19,138
Number of PSUs     =     1,218                 Population size   =  28,354,881
                                               Design df         =       1,165

-------------------------------------------------------
          |           missing_height_weight
  gender_ |            0             1         Total
----------+--------------------------------------------
     Male |        .8065         .1935             1
          | [.7926,.8196]  [.1804,.2074]
          |
   Female |        .8102         .1898             1
          | [.7951,.8245]  [.1755,.2049]
          |
    Total |        .8083         .1917             1
          |  [.7972,.819]   [.181,.2028]
-------------------------------------------------------
  Key:  row proportion
        [95% confidence interval for row proportion]

  Pearson:
    Uncorrected   chi2(1)         =    0.4400
    Design-based  F(1, 1165)      =    0.1697     P = 0.6805
```

```
. svy: tab age_ missing_height_weight, row ci
(running tabulate on estimation sample)

Number of strata   =        53                Number of obs     =      19,201
Number of PSUs     =     1,227                Population size   =  28,456,616
                                              Design df         =       1,174

-------------------------------------------------------
          |           missing_height_weight
     age_ |             0              1         Total
----------+--------------------------------------------
        0 |         .4596          .5404             1
          | [.4362,.4832]  [.5168,.5638]
          |
        1 |         .8581          .1419             1
          | [.8308,.8816]  [.1184,.1692]
          |
        2 |         .8764          .1236             1
          | [.8573,.8933]  [.1067,.1427]
          |
        3 |         .8952          .1048             1
          | [.8726,.9142]  [.0858,.1274]
          |
        4 |         .9015          .0985             1
          |  [.8847,.916]   [.084,.1153]
          |
    Total |         .8083          .1917             1
          | [.7972,.8189]  [.1811,.2028]
-------------------------------------------------------
  Key:  row proportion
        [95% confidence interval for row proportion]

  Pearson:
    Uncorrected   chi2(4)         = 3267.7805
    Design-based  F(3.41, 3999.27)=  238.9174     P = 0.0000

. svy: tab hh_inc missing_height_weight, row ci
(running tabulate on estimation sample)

Number of strata   =        53                Number of obs     =      18,289
Number of PSUs     =     1,195                Population size   =  26,887,499
                                              Design df         =       1,142

-------------------------------------------------------
          |           missing_height_weight
   hh_inc |             0              1         Total
----------+--------------------------------------------
        1 |         .8032          .1968             1
          | [.7792,.8251]  [.1749,.2208]
          |
        2 |         .8286          .1714             1
          |  [.8012,.853]   [.147,.1988]
          |
        3 |         .8289          .1711             1
          | [.8084,.8475]  [.1525,.1916]
          |
        4 |         .8076          .1924             1
          | [.7751,.8365]  [.1635,.2249]
          |
        5 |         .7862          .2138             1
          |  [.7578,.812]   [.188,.2422]
          |
    Total |         .8096          .1904             1
          | [.7982,.8205]  [.1795,.2018]
-------------------------------------------------------
  Key:  row proportion
        [95% confidence interval for row proportion]

  Pearson:
    Uncorrected   chi2(4)         =   32.2620
    Design-based  F(3.67, 4186.36)=    1.9756     P = 0.1017

. svy: tab province missing_height_weight, row ci
(running tabulate on estimation sample)

Number of strata   =        53                Number of obs     =      19,201
Number of PSUs     =     1,227                Population size   =  28,456,616
```

```
                                              Design df         =      1,174

-------------------------------------------------------
          |           missing_height_weight
 province |            0             1         Total
----------+--------------------------------------------
  Eastern |        .8421         .1579             1
          |  [.819,.8627]   [.1373,.181]
          |
 Free Sta |         .833          .167             1
          | [.7968,.8638]  [.1362,.2032]
          |
  Gauteng |        .7866         .2134             1
          | [.7637,.8078]  [.1922,.2363]
          |
 KwaZulu- |        .8448         .1552             1
          | [.8255,.8624]  [.1376,.1745]
          |
  Limpopo |        .8422         .1578             1
          | [.8184,.8634]  [.1366,.1816]
          |
 Mpumalan |        .7557         .2443             1
          | [.7187,.7892]  [.2108,.2813]
          |
 North We |        .8011         .1989             1
          |  [.7725,.827]   [.173,.2275]
          |
 Northern |        .7921         .2079             1
          | [.7674,.8149]  [.1851,.2326]
          |
  Western |        .7422         .2578             1
          |  [.7064,.775]   [.225,.2936]
          |
    Total |        .8083         .1917             1
          | [.7972,.8189]  [.1811,.2028]
-------------------------------------------------------
  Key:  row proportion
        [95% confidence interval for row proportion]

  Pearson:
    Uncorrected   chi2(8)         =  171.9467
    Design-based  F(6.89, 8090.45)=    9.8218     P = 0.0000

. svy: tab LBW missing_height_weight, row ci
(running tabulate on estimation sample)

Number of strata   =        53                Number of obs     =     16,606
Number of PSUs     =     1,128                Population size   =  24,829,511
                                              Design df         =      1,075

-------------------------------------------------------
          |           missing_height_weight
      LBW |            0             1         Total
----------+--------------------------------------------
        0 |        .8164         .1836             1
          | [.8044,.8278]  [.1722,.1956]
          |
        1 |        .8106         .1894             1
          | [.7788,.8388]  [.1612,.2212]
          |
    Total |        .8158         .1842             1
          | [.8045,.8266]  [.1734,.1955]
-------------------------------------------------------
  Key:  row proportion
        [95% confidence interval for row proportion]

  Pearson:
    Uncorrected   chi2(1)         =    0.3369
    Design-based  F(1, 1075)      =    0.1307     P = 0.7178

. svy: tab foodsecurity_proxy missing_height_weight, row ci
(running tabulate on estimation sample)

Number of strata   =        53                 Number of obs     =      5,017
Number of PSUs     =       438                 Population size   =  8,843,019
                                               Design df         =        385
```

```
------------------------------------------------------
foodsecur |            missing_height_weight
ity_proxy |             0              1         Total
----------+-------------------------------------------
        1 |         .7719          .2281             1
          | [.7467,.7952]  [.2048,.2533]
          |
        2 |         .8019          .1981             1
          | [.7352,.8551]  [.1449,.2648]
          |
        3 |         .7596          .2404             1
          | [.6983,.8119]  [.1881,.3017]
          |
        4 |         .8284          .1716             1
          | [.7561,.8825]  [.1175,.2439]
          |
        5 |         .7869          .2131             1
          | [.6923,.8583]  [.1417,.3077]
          |
    Total |         .7751          .2249             1
          |  [.753,.7959]   [.2041,.247]
------------------------------------------------------
  Key:  row proportion
        [95% confidence interval for row proportion]

  Pearson:
    Uncorrected   chi2(4)         =    6.4682
    Design-based  F(3.04, 1168.67)=    0.8267     P = 0.4803

. svy: tab mthagegrp missing_height_weight, row ci
(running tabulate on estimation sample)

Number of strata   =        53                Number of obs     =      17,335
Number of PSUs     =     1,192                Population size   =  26,432,345
                                              Design df         =       1,139

------------------------------------------------------
RECODE of |
mth_age_f |            missing_height_weight
inal      |             0              1         Total
----------+-------------------------------------------
        1 |         .6691          .3309             1
          |  [.628,.7077]   [.2923,.372]
          |
        2 |         .7787          .2213             1
          | [.7553,.8004]  [.1996,.2447]
          |
        3 |         .8128          .1872             1
          | [.7961,.8285]  [.1715,.2039]
          |
        4 |         .8329          .1671             1
          | [.8096,.8539]  [.1461,.1904]
          |
        5 |         .8629          .1371             1
          | [.7939,.9113]  [.0887,.2061]
          |
    Total |         .8006          .1994             1
          | [.7894,.8115]  [.1885,.2106]
------------------------------------------------------
  Key:  row proportion
        [95% confidence interval for row proportion]

  Pearson:
    Uncorrected   chi2(4)         =  169.4906
    Design-based  F(3.89, 4436.22)=   15.6564     P = 0.0000

. svy: tab mth_edu2 missing_height_weight, row ci
(running tabulate on estimation sample)

Number of strata   =        53                Number of obs     =      16,352
Number of PSUs     =     1,169                Population size   =  25,254,660
                                              Design df         =       1,116

------------------------------------------------------
          |            missing_height_weight
 mth_edu2 |             0              1         Total
----------+-------------------------------------------
```

```
        0 |         .8043          .1957              1
          | [.7537,.8466]  [.1534,.2463]
          |
        1 |         .7848          .2152              1
          | [.7529,.8136]  [.1864,.2471]
          |
        2 |         .7976          .2024              1
          | [.7848,.8098]  [.1902,.2152]
          |
        3 |         .8122          .1878              1
          | [.7734,.8457]  [.1543,.2266]
          |
    Total |         .7982          .2018              1
          | [.7868,.8092]  [.1908,.2132]
-------------------------------------------------------
  Key:  row proportion
        [95% confidence interval for row proportion]

  Pearson:
    Uncorrected   chi2(3)         =    3.7648
    Design-based  F(2.41, 2688.60)=    0.5454     P = 0.6124

. svy: tab fth_educat missing_height_weight, row ci
(running tabulate on estimation sample)

Number of strata   =        53                 Number of obs     =      4,574
Number of PSUs     =       755                 Population size   =  8,485,206
                                               Design df         =        702

-------------------------------------------------------
fth_educa |           missing_height_weight
t         |             0              1          Total
----------+--------------------------------------------
        0 |         .9417          .0583              1
          |  [.732,.9896]   [.0104,.268]
          |
        1 |         .7923          .2077              1
          | [.7634,.8185]  [.1815,.2366]
          |
        2 |          .803           .197              1
          |  [.759,.8406]   [.1594,.241]
          |
        3 |         .7618          .2382              1
          | [.6661,.8368]  [.1632,.3339]
          |
    Total |         .7948          .2052              1
          | [.7719,.8159]  [.1841,.2281]
-------------------------------------------------------
  Key:  row proportion
        [95% confidence interval for row proportion]

  Pearson:
    Uncorrected   chi2(3)         =    3.8826
    Design-based  F(2.17, 1522.03)=    0.5250     P = 0.6062
```

Supplementary 9: Full posterior prevalence estimates with 95% Bayesian uncertainty intervals (UIs) by district and year. Also includes exceedance probabilities for 17% reduction in stunting from wave 3 (2012) to wave 5 (2017) - to achieve 40% reduction from 2012 to 2025, 5% target threshold for wasting prevalence and no increase in obesity from wave 3 (2012) to wave 5 (2017) as per 2025 nutritional targets.

Province	District	wave	stunting	95% UI		Exceedance probability 17% reduction from wave 3 to 5	thinness	95% UI		Exceedance probability 5% target threshold	obesity	95% UI		Exceedance probability - no increase from wave 3 to 5
Eastern Cape	Alfred Nzo(DC44)	1	9.2%	4.4%	16.1%	N/A	6.1%	2.3%	12.1%	0.6223	16.9%	9.3%	26.2%	N/A
KwaZulu-Natal	Amajuba(DC25)	1	9.7%	5.1%	15.7%	N/A	5.1%	2.1%	9.8%	0.4572	16.1%	9.8%	24.0%	N/A
Eastern Cape	Amathole(DC12)	1	14.8%	6.4%	27.4%	N/A	12.4%	3.9%	26.8%	0.9399	28.2%	14.4%	46.2%	N/A
North West	Bojanala(DC37)	1	10.2%	4.6%	18.4%	N/A	5.7%	1.9%	12.3%	0.5349	9.7%	4.3%	17.7%	N/A
Eastern Cape	Buffalo City(BUF)	1	19.0%	8.3%	35.1%	N/A	14.2%	4.0%	33.4%	0.9435	28.5%	13.0%	48.8%	N/A
Eastern Cape	Cacadu(DC10)	1	21.7%	12.9%	32.5%	N/A	8.0%	2.2%	19.8%	0.7199	18.2%	10.3%	28.0%	N/A
Western Cape	Cape Winelands(DC2)	1	12.5%	4.7%	25.8%	N/A	9.7%	4.4%	17.1%	0.9475	18.7%	10.8%	28.3%	N/A
Limpopo	Capricorn(DC35)	1	12.4%	6.5%	20.6%	N/A	10.1%	4.6%	18.2%	0.9578	12.2%	4.6%	25.1%	N/A
Western Cape	Central Karoo(DC5)	1	16.0%	9.0%	24.9%	N/A	7.6%	3.2%	14.3%	0.8194	13.9%	7.6%	22.2%	N/A
Eastern Cape	Chris Hani(DC13)	1	9.7%	4.7%	17.0%	N/A	7.4%	2.1%	18.3%	0.6688	27.5%	17.5%	38.9%	N/A
Western Cape	City of Cape Town(CPT)	1	8.1%	4.0%	13.8%	N/A	9.0%	2.4%	21.6%	0.7817	15.6%	8.9%	24.0%	N/A
Gauteng	City of Johannesburg(JHB)	1	9.6%	4.8%	15.9%	N/A	4.6%	1.6%	9.4%	0.3591	16.3%	9.5%	24.7%	N/A
Gauteng	City of Tshwane(TSH)	1	18.3%	11.1%	27.2%	N/A	12.8%	6.5%	20.8%	0.9967	9.5%	4.9%	15.8%	N/A
North West	Dr Kenneth Kaunda(DC40)	1	13.4%	6.5%	23.0%	N/A	13.4%	5.8%	24.4%	0.9898	14.7%	7.2%	25.0%	N/A
North West	Dr Ruth Segomotsi Mompati(DC39)	1	11.2%	6.4%	17.5%	N/A	13.5%	7.5%	20.9%	0.9997	10.0%	5.5%	15.9%	N/A
Western Cape	Eden(DC4)	1	13.7%	6.8%	23.5%	N/A	9.8%	2.5%	25.0%	0.81	21.8%	12.0%	34.2%	N/A
Mpumalanga	Ehlanzeni(DC32)	1	10.7%	5.7%	17.3%	N/A	4.0%	1.5%	8.0%	0.2425	8.9%	4.6%	14.9%	N/A
Gauteng	Ekurhuleni(EKU)	1	13.2%	6.5%	22.0%	N/A	5.5%	1.9%	11.3%	0.5078	9.0%	4.0%	16.2%	N/A
Free State	Fezile Dabi(DC20)	1	12.7%	5.8%	23.0%	N/A	7.6%	2.0%	19.2%	0.692	25.6%	13.8%	40.1%	N/A
Northern Cape	Frances Baard(DC9)	1	13.2%	7.4%	20.7%	N/A	5.6%	2.3%	10.6%	0.5542	8.9%	4.5%	15.1%	N/A
Mpumalanga	Gert Sibande(DC30)	1	7.6%	3.8%	13.0%	N/A	4.1%	1.5%	8.2%	0.2558	11.5%	6.4%	18.1%	N/A
Limpopo	Greater Sekhukhune(DC47)	1	14.6%	8.1%	22.9%	N/A	7.9%	3.5%	14.3%	0.8598	7.3%	3.4%	12.9%	N/A
Eastern Cape	Joe Gqabi(DC14)	1	12.8%	6.8%	21.0%	N/A	4.6%	1.7%	9.6%	0.3627	18.6%	10.9%	28.4%	N/A
Northern Cape	John Taolo Gaetsewe(DC45)	1	8.4%	3.7%	15.3%	N/A	6.0%	2.1%	12.5%	0.5885	11.5%	5.6%	20.0%	N/A
Free State	Lejweleputswa(DC18)	1	11.1%	5.0%	19.7%	N/A	7.0%	2.5%	14.6%	0.714	9.9%	4.4%	17.8%	N/A
Free State	Mangaung(MAN)	1	35.1%	21.0%	51.4%	N/A	7.6%	2.0%	19.6%	0.6713	16.5%	6.3%	33.5%	N/A
Limpopo	Mopani(DC33)	1	8.2%	3.8%	14.5%	N/A	5.6%	2.1%	11.2%	0.5388	9.6%	4.7%	16.6%	N/A
Northern Cape	Namakwa(DC6)	1	14.6%	7.6%	24.1%	N/A	7.4%	2.8%	14.6%	0.7848	10.5%	4.9%	18.1%	N/A
Eastern Cape	Nelson Mandela Bay(NMA)	1	11.2%	5.6%	18.9%	N/A	5.5%	2.0%	11.2%	0.5236	25.3%	15.5%	37.0%	N/A
North West	Ngaka Modiri Molema(DC38)	1	11.1%	5.8%	18.2%	N/A	5.7%	2.2%	11.0%	0.5684	18.1%	10.6%	27.3%	N/A
Mpumalanga	Nkangala(DC31)	1	13.3%	7.3%	21.1%	N/A	11.7%	5.7%	20.0%	0.9902	15.4%	8.6%	23.7%	N/A
Eastern Cape	O.R.Tambo(DC15)	1	19.5%	12.6%	27.8%	N/A	3.5%	1.3%	7.0%	0.1417	24.4%	16.5%	33.4%	N/A
Western Cape	Overberg(DC3)	1	11.5%	5.8%	19.4%	N/A	5.4%	1.9%	10.9%	0.4959	17.7%	9.9%	27.8%	N/A
Northern Cape	Pixley ka Seme(DC7)	1	25.0%	13.7%	39.7%	N/A	8.3%	2.3%	20.3%	0.7481	26.1%	14.7%	40.7%	N/A
Gauteng	Sedibeng(DC42)	1	16.5%	9.2%	26.2%	N/A	15.8%	7.9%	26.3%	0.9993	12.3%	6.4%	20.3%	N/A
KwaZulu-Natal	Sisonke(DC43)	1	18.4%	11.3%	26.9%	N/A	8.1%	3.8%	14.5%	0.894	20.6%	13.1%	29.4%	N/A
Northern Cape	Siyanda(DC8)	1	13.3%	7.2%	21.2%	N/A	7.0%	2.7%	13.3%	0.7479	9.1%	4.4%	15.6%	N/A
Free State	Thabo Mofutsanyane(DC19)	1	12.8%	6.2%	22.1%	N/A	7.2%	2.0%	18.0%	0.653	17.5%	9.1%	28.2%	N/A
KwaZulu-Natal	UMgungundlovu(DC22)	1	8.5%	4.1%	14.5%	N/A	4.5%	1.6%	9.1%	0.3328	20.5%	12.6%	30.2%	N/A
KwaZulu-Natal	Ugu(DC21)	1	8.1%	4.4%	13.1%	N/A	2.9%	1.1%	5.8%	0.0602	19.1%	12.6%	26.7%	N/A

KwaZulu-Natal	Umkhanyakude(DC27)	1	11.2%	5.6%	18.9%	N/A	8.3%	3.5%	15.9%	0.8631	13.8%	7.2%	22.5%	N/A
KwaZulu-Natal	Umzinyathi(DC24)	1	12.8%	4.9%	26.1%	N/A	5.5%	1.4%	14.4%	0.4431	19.0%	7.7%	36.5%	N/A
KwaZulu-Natal	Uthukela(DC23)	1	5.6%	3.0%	9.1%	N/A	4.2%	2.0%	7.5%	0.2652	10.7%	6.7%	15.5%	N/A
KwaZulu-Natal	Uthungulu(DC28)	1	11.4%	6.4%	17.8%	N/A	3.2%	1.2%	6.6%	0.1071	19.0%	12.0%	27.4%	N/A
Limpopo	Vhembe(DC34)	1	25.5%	15.4%	38.0%	N/A	4.5%	1.5%	9.9%	0.3466	20.2%	11.3%	31.3%	N/A
Limpopo	Waterberg(DC36)	1	12.0%	6.3%	19.6%	N/A	6.5%	2.7%	12.2%	0.6979	12.1%	6.4%	19.5%	N/A
Western Cape	West Coast(DC1)	1	10.4%	4.6%	18.7%	N/A	8.5%	2.3%	21.0%	0.7495	26.7%	15.6%	40.5%	N/A
Gauteng	West Rand(DC48)	1	11.0%	5.0%	19.9%	N/A	8.9%	3.4%	18.0%	0.8797	11.6%	5.3%	20.5%	N/A
Free State	Xhariep(DC16)	1	20.2%	11.3%	31.3%	N/A	7.1%	2.0%	17.3%	0.6479	10.2%	4.8%	18.0%	N/A
KwaZulu-Natal	Zululand(DC26)	1	8.2%	4.0%	14.0%	N/A	3.6%	1.3%	7.5%	0.1747	13.9%	7.9%	21.8%	N/A
KwaZulu-Natal	eThekwini(ETH)	1	8.1%	4.1%	13.7%	N/A	3.5%	1.3%	7.2%	0.15	19.5%	12.3%	28.5%	N/A
KwaZulu-Natal	iLembe(DC29)	1	10.7%	5.7%	17.4%	N/A	5.1%	1.3%	13.2%	0.4016	37.3%	27.2%	48.2%	N/A
Eastern Cape	Alfred Nzo(DC44)	2	16.2%	8.8%	25.8%	N/A	7.3%	1.9%	18.1%	0.659	18.0%	10.0%	28.0%	N/A
KwaZulu-Natal	Amajuba(DC25)	2	7.6%	4.0%	12.5%	N/A	6.6%	3.1%	11.6%	0.7634	11.6%	6.7%	17.7%	N/A
Eastern Cape	Amathole(DC12)	2	15.6%	5.8%	31.2%	N/A	30.8%	14.7%	51.8%	1	27.4%	11.7%	48.8%	N/A
North West	Bojanala(DC37)	2	10.8%	5.4%	18.4%	N/A	9.0%	2.4%	22.1%	0.7872	15.6%	8.6%	24.9%	N/A
Eastern Cape	Buffalo City(BUF)	2	16.2%	6.0%	33.4%	N/A	14.4%	3.0%	40.0%	0.8966	34.4%	14.5%	60.0%	N/A
Eastern Cape	Cacadu(DC10)	2	17.3%	9.9%	26.7%	N/A	9.8%	2.7%	24.0%	0.8257	9.8%	4.7%	16.9%	N/A
Western Cape	Cape Winelands(DC2)	2	10.7%	5.5%	17.7%	N/A	16.9%	9.5%	26.4%	0.9999	24.4%	15.6%	34.5%	N/A
Limpopo	Capricorn(DC35)	2	17.8%	10.2%	27.3%	N/A	8.5%	3.8%	15.5%	0.8982	8.8%	4.1%	15.5%	N/A
Western Cape	Central Karoo(DC5)	2	11.2%	5.7%	18.5%	N/A	10.1%	2.7%	24.5%	0.842	17.2%	6.6%	33.5%	N/A
Eastern Cape	Chris Hani(DC13)	2	10.9%	5.7%	17.9%	N/A	9.0%	2.4%	22.0%	0.7881	24.0%	15.3%	34.0%	N/A
Western Cape	City of Cape Town(CPT)	2	18.1%	11.2%	26.3%	N/A	14.7%	8.3%	22.7%	0.9998	37.5%	27.7%	48.0%	N/A
Gauteng	City of Johannesburg(JHB)	2	15.6%	9.5%	23.2%	N/A	9.9%	2.7%	24.2%	0.8305	14.9%	8.9%	22.3%	N/A
Gauteng	City of Tshwane(TSH)	2	17.3%	10.7%	25.4%	N/A	9.2%	2.5%	22.2%	0.8056	19.5%	12.3%	28.0%	N/A
North West	Dr Kenneth Kaunda(DC40)	2	24.5%	13.9%	37.9%	N/A	10.1%	2.7%	24.2%	0.8362	16.1%	8.3%	26.7%	N/A
North West	Dr Ruth Segomotsi Mompati(DC39)	2	17.0%	10.5%	24.8%	N/A	12.4%	6.7%	19.7%	0.9974	13.1%	7.4%	20.2%	N/A
Western Cape	Eden(DC4)	2	22.8%	12.1%	36.7%	N/A	11.8%	3.1%	29.0%	0.8861	20.1%	8.1%	38.6%	N/A
Mpumalanga	Ehlanzeni(DC32)	2	10.6%	5.8%	17.0%	N/A	6.0%	2.6%	11.1%	0.6463	13.3%	7.6%	20.3%	N/A
Gauteng	Ekurhuleni(EKU)	2	10.8%	5.5%	18.2%	N/A	9.4%	2.5%	23.1%	0.8025	21.1%	12.8%	31.3%	N/A
Free State	Fezile Dabi(DC20)	2	14.9%	5.7%	29.8%	N/A	9.4%	2.5%	23.3%	0.8069	18.1%	9.0%	30.2%	N/A
Northern Cape	Frances Baard(DC9)	2	14.2%	8.1%	21.9%	N/A	4.7%	1.8%	9.3%	0.3905	10.3%	5.5%	16.7%	N/A
Mpumalanga	Gert Sibande(DC30)	2	14.2%	8.6%	21.0%	N/A	8.6%	2.3%	21.1%	0.769	21.4%	14.3%	29.5%	N/A
Limpopo	Greater Sekhukhune(DC47)	2	18.0%	11.2%	26.0%	N/A	5.8%	2.5%	10.8%	0.6066	9.4%	5.0%	15.1%	N/A
Eastern Cape	Joe Gqabi(DC14)	2	10.5%	5.3%	17.6%	N/A	7.6%	2.0%	19.1%	0.6798	20.0%	12.1%	29.7%	N/A
Northern Cape	John Taolo Gaetsewe(DC45)	2	9.1%	4.0%	16.7%	N/A	14.7%	6.6%	25.7%	0.9957	11.6%	5.6%	20.3%	N/A
Free State	Lejweleputswa(DC18)	2	19.4%	11.1%	29.9%	N/A	13.0%	6.2%	22.4%	0.9947	15.9%	6.3%	31.0%	N/A
Free State	Mangaung(MAN)	2	15.6%	7.4%	26.6%	N/A	9.4%	2.4%	23.5%	0.7897	19.7%	7.8%	38.7%	N/A
Limpopo	Mopani(DC33)	2	12.2%	6.3%	19.9%	N/A	6.8%	2.7%	13.0%	0.7201	20.5%	12.2%	30.6%	N/A
Northern Cape	Namakwa(DC6)	2	14.7%	5.7%	29.1%	N/A	10.7%	3.0%	26.0%	0.8658	15.5%	5.9%	30.8%	N/A
Eastern Cape	Nelson Mandela Bay(NMA)	2	11.4%	6.0%	18.3%	N/A	6.0%	2.4%	11.6%	0.6202	17.6%	10.5%	26.6%	N/A
North West	Ngaka Modiri Molema(DC38)	2	19.0%	11.9%	27.9%	N/A	11.7%	6.0%	19.3%	0.9936	9.6%	4.9%	15.9%	N/A
Mpumalanga	Nkangala(DC31)	2	9.7%	4.8%	16.3%	N/A	9.3%	2.6%	22.1%	0.8075	17.3%	7.1%	33.3%	N/A
Eastern Cape	O.R.Tambo(DC15)	2	24.8%	16.8%	33.8%	N/A	4.4%	1.7%	8.6%	0.3192	31.4%	22.3%	41.3%	N/A
Western Cape	Overberg(DC3)	2	14.1%	5.2%	28.3%	N/A	8.8%	3.7%	16.3%	0.9011	25.0%	15.7%	35.8%	N/A
Northern Cape	Pixley ka Seme(DC7)	2	15.3%	5.9%	30.5%	N/A	10.1%	2.7%	24.1%	0.8434	19.0%	7.6%	35.7%	N/A
Gauteng	Sedibeng(DC42)	2	14.7%	8.4%	22.9%	N/A	9.6%	4.5%	16.9%	0.9546	14.6%	8.3%	22.5%	N/A
KwaZulu-Natal	Sisonke(DC43)	2	20.0%	12.9%	28.6%	N/A	7.5%	1.9%	18.9%	0.6726	50.2%	39.8%	60.8%	N/A

Northern Cape	Siyanda(DC8)	2	10.5%	5.2%	17.5%	N/A	26.1%	16.1%	38.3%	1	8.6%	4.1%	15.0%	N/A
Free State	Thabo Mofutsanyane(DC19)	2	16.1%	8.2%	27.1%	N/A	13.7%	6.0%	25.5%	0.9908	27.0%	15.7%	41.1%	N/A
KwaZulu-Natal	UMgungundlovu(DC22)	2	19.4%	12.3%	28.3%	N/A	6.8%	1.8%	17.3%	0.6059	16.6%	9.9%	24.6%	N/A
KwaZulu-Natal	Ugu(DC21)	2	17.2%	11.3%	23.9%	N/A	5.0%	2.3%	8.8%	0.4501	36.3%	28.2%	44.7%	N/A
KwaZulu-Natal	Umkhanyakude(DC27)	2	18.3%	9.6%	29.7%	N/A	7.1%	1.7%	18.6%	0.6238	30.8%	18.6%	44.7%	N/A
KwaZulu-Natal	Umzinyathi(DC24)	2	14.5%	5.5%	29.2%	N/A	6.6%	1.7%	17.0%	0.5943	22.6%	9.3%	42.2%	N/A
KwaZulu-Natal	Uthukela(DC23)	2	11.1%	7.3%	15.6%	N/A	3.0%	1.3%	5.5%	0.0477	25.4%	19.6%	31.7%	N/A
KwaZulu-Natal	Uthungulu(DC28)	2	16.0%	9.5%	24.2%	N/A	6.0%	2.5%	11.2%	0.6329	28.7%	19.9%	38.6%	N/A
Limpopo	Vhembe(DC34)	2	31.6%	20.1%	44.8%	N/A	7.2%	1.8%	19.1%	0.6245	30.0%	18.9%	43.1%	N/A
Limpopo	Waterberg(DC36)	2	18.2%	11.1%	26.6%	N/A	7.1%	3.1%	12.8%	0.7899	14.8%	8.6%	22.5%	N/A
Western Cape	West Coast(DC1)	2	13.8%	5.0%	28.6%	N/A	10.3%	2.6%	25.9%	0.8387	20.7%	8.2%	39.2%	N/A
Gauteng	West Rand(DC48)	2	11.1%	5.4%	19.3%	N/A	7.1%	2.7%	14.2%	0.7375	22.4%	13.0%	33.8%	N/A
Free State	Xhariep(DC16)	2	15.7%	6.0%	31.4%	N/A	8.7%	2.4%	21.7%	0.7647	24.7%	14.4%	37.2%	N/A
KwaZulu-Natal	Zululand(DC26)	2	11.4%	6.0%	18.4%	N/A	6.1%	2.5%	11.9%	0.6421	12.7%	6.8%	20.2%	N/A
KwaZulu-Natal	eThekwini(ETH)	2	15.5%	9.6%	22.6%	N/A	4.9%	2.1%	9.2%	0.4327	32.5%	23.8%	42.1%	N/A
KwaZulu-Natal	iLembe(DC29)	2	8.9%	4.4%	14.9%	N/A	5.2%	2.1%	10.3%	0.4818	29.4%	20.1%	39.7%	N/A
Eastern Cape	Alfred Nzo(DC44)	3	17.0%	9.7%	26.5%	N/A	6.2%	2.5%	12.3%	0.6337	25.0%	15.7%	36.2%	N/A
KwaZulu-Natal	Amajuba(DC25)	3	12.2%	6.8%	19.4%	N/A	4.5%	1.8%	8.8%	0.341	14.3%	8.3%	21.9%	N/A
Eastern Cape	Amathole(DC12)	3	12.1%	5.4%	22.2%	N/A	10.1%	3.5%	20.9%	0.901	17.1%	7.8%	29.7%	N/A
North West	Bojanala(DC37)	3	8.6%	4.1%	14.7%	N/A	5.7%	2.2%	11.1%	0.5831	10.3%	5.2%	17.2%	N/A
Eastern Cape	Buffalo City(BUF)	3	14.3%	5.2%	30.4%	N/A	11.2%	2.2%	33.1%	0.7951	39.0%	20.0%	61.1%	N/A
Eastern Cape	Cacadu(DC10)	3	9.8%	4.7%	16.9%	N/A	6.6%	2.6%	13.0%	0.7016	12.5%	6.3%	20.5%	N/A
Western Cape	Cape Winelands(DC2)	3	9.0%	4.3%	15.7%	N/A	5.0%	1.7%	10.3%	0.4359	17.1%	9.6%	26.4%	N/A
Limpopo	Capricorn(DC35)	3	17.1%	10.5%	25.3%	N/A	4.6%	1.8%	8.9%	0.3588	11.2%	6.1%	17.9%	N/A
Western Cape	Central Karoo(DC5)	3	11.6%	6.0%	19.0%	N/A	4.9%	1.8%	10.0%	0.4155	13.8%	7.5%	22.1%	N/A
Eastern Cape	Chris Hani(DC13)	3	12.4%	6.9%	19.6%	N/A	8.9%	4.2%	15.6%	0.9323	22.2%	14.2%	31.4%	N/A
Western Cape	City of Cape Town(CPT)	3	10.1%	5.7%	15.8%	N/A	4.8%	2.0%	9.0%	0.403	23.3%	16.2%	31.5%	N/A
Gauteng	City of Johannesburg(JHB)	3	14.7%	8.9%	21.8%	N/A	10.6%	5.6%	17.3%	0.9889	14.5%	8.6%	21.6%	N/A
Gauteng	City of Tshwane(TSH)	3	9.4%	5.2%	15.1%	N/A	6.1%	2.8%	10.9%	0.675	14.6%	9.0%	21.8%	N/A
North West	Dr Kenneth Kaunda(DC40)	3	15.9%	8.0%	26.5%	N/A	7.6%	2.1%	19.0%	0.6966	17.6%	9.1%	29.4%	N/A
North West	Dr Ruth Segomotsi Mompati(DC39)	3	21.0%	13.3%	30.0%	N/A	4.6%	1.7%	9.1%	0.3583	13.5%	7.7%	21.1%	N/A
Western Cape	Eden(DC4)	3	10.6%	4.6%	19.4%	N/A	20.0%	9.4%	34.8%	0.9997	13.5%	6.2%	23.4%	N/A
Mpumalanga	Ehlanzeni(DC32)	3	26.6%	19.0%	35.2%	N/A	8.6%	4.5%	14.0%	0.9527	23.1%	16.1%	31.0%	N/A
Gauteng	Ekurhuleni(EKU)	3	8.0%	3.9%	13.7%	N/A	4.3%	1.6%	8.8%	0.3087	14.5%	8.2%	22.6%	N/A
Free State	Fezile Dabi(DC20)	3	13.4%	6.3%	23.9%	N/A	7.1%	1.9%	17.8%	0.6345	17.2%	8.2%	29.2%	N/A
Northern Cape	Frances Baard(DC9)	3	9.1%	4.7%	15.3%	N/A	5.8%	2.4%	11.0%	0.6016	10.2%	5.4%	16.6%	N/A
Mpumalanga	Gert Sibande(DC30)	3	12.9%	7.6%	19.4%	N/A	13.3%	7.5%	20.6%	0.9999	22.2%	14.9%	30.6%	N/A
Limpopo	Greater Sekhukhune(DC47)	3	11.0%	6.6%	16.6%	N/A	4.1%	1.8%	7.6%	0.2448	14.2%	8.9%	20.8%	N/A
Eastern Cape	Joe Gqabi(DC14)	3	18.8%	11.2%	28.5%	N/A	3.7%	1.3%	7.8%	0.2	29.1%	19.5%	40.1%	N/A
Northern Cape	John Taolo Gaetsewe(DC45)	3	8.8%	4.1%	15.6%	N/A	5.9%	2.1%	11.9%	0.5754	14.4%	7.6%	23.5%	N/A
Free State	Lejweleputswa(DC18)	3	14.8%	7.9%	23.7%	N/A	7.4%	3.1%	13.8%	0.798	12.2%	6.3%	20.1%	N/A
Free State	Mangaung(MAN)	3	17.6%	9.3%	28.5%	N/A	7.0%	1.7%	18.0%	0.6214	15.6%	7.8%	26.0%	N/A
Limpopo	Mopani(DC33)	3	23.9%	14.7%	34.6%	N/A	9.0%	3.9%	16.5%	0.92	26.7%	17.0%	37.8%	N/A
Northern Cape	Namakwa(DC6)	3	13.3%	6.6%	22.3%	N/A	9.8%	4.1%	18.2%	0.9336	11.6%	5.5%	20.2%	N/A
Eastern Cape	Nelson Mandela Bay(NMA)	3	16.1%	9.1%	25.0%	N/A	4.9%	1.7%	10.2%	0.4129	20.3%	11.8%	30.9%	N/A
North West	Ngaka Modiri Molema(DC38)	3	16.0%	9.8%	23.5%	N/A	6.6%	2.9%	11.8%	0.7373	24.9%	16.9%	33.9%	N/A
Mpumalanga	Nkangala(DC31)	3	7.3%	3.3%	13.2%	N/A	5.7%	2.1%	11.3%	0.5696	16.9%	9.5%	26.4%	N/A
Eastern Cape	O.R.Tambo(DC15)	3	13.1%	7.4%	20.3%	N/A	3.2%	1.1%	6.5%	0.1062	30.8%	21.9%	40.7%	N/A

Western Cape	Overberg(DC3)	3	8.8%	4.3%	15.0%	N/A	7.3%	1.9%	18.6%	0.6533	33.8%	23.3%	45.2%	N/A
Northern Cape	Pixley ka Seme(DC7)	3	13.4%	5.0%	26.8%	N/A	15.5%	6.7%	28.7%	0.9958	15.2%	7.3%	26.6%	N/A
Gauteng	Sedibeng(DC42)	3	10.6%	5.6%	17.2%	N/A	5.9%	2.4%	11.0%	0.609	13.2%	7.6%	20.7%	N/A
KwaZulu-Natal	Sisonke(DC43)	3	14.6%	9.2%	21.3%	N/A	9.3%	5.0%	15.0%	0.9734	29.5%	21.7%	38.0%	N/A
Northern Cape	Siyanda(DC8)	3	14.1%	8.2%	21.8%	N/A	13.7%	7.5%	21.7%	0.9988	8.2%	4.1%	13.6%	N/A
Free State	Thabo Mofutsanyane(DC19)	3	17.3%	9.2%	28.2%	N/A	5.2%	1.7%	11.3%	0.4635	14.7%	7.3%	24.4%	N/A
KwaZulu-Natal	UMgungundlovu(DC22)	3	12.9%	7.3%	20.2%	N/A	7.7%	3.5%	13.9%	0.8559	21.6%	13.8%	30.9%	N/A
KwaZulu-Natal	Ugu(DC21)	3	17.6%	11.8%	24.3%	N/A	5.3%	2.5%	9.3%	0.5197	25.8%	18.7%	33.9%	N/A
KwaZulu-Natal	Umkhanyakude(DC27)	3	10.4%	5.6%	16.8%	N/A	3.2%	1.1%	6.7%	0.1092	19.0%	11.9%	27.3%	N/A
KwaZulu-Natal	Umzinyathi(DC24)	3	12.7%	4.9%	25.5%	N/A	5.0%	1.3%	12.9%	0.3875	21.1%	8.4%	39.7%	N/A
KwaZulu-Natal	Uthukela(DC23)	3	13.7%	9.5%	18.4%	N/A	3.4%	1.7%	5.9%	0.091	23.6%	18.2%	29.5%	N/A
KwaZulu-Natal	Uthungulu(DC28)	3	16.1%	9.9%	23.6%	N/A	3.3%	1.2%	6.6%	0.1242	23.7%	16.0%	32.3%	N/A
Limpopo	Vhembe(DC34)	3	7.5%	3.4%	13.7%	N/A	3.4%	1.1%	7.4%	0.1458	9.7%	4.7%	16.4%	N/A
Limpopo	Waterberg(DC36)	3	12.5%	7.3%	19.5%	N/A	6.5%	1.8%	15.9%	0.5857	9.2%	4.9%	15.2%	N/A
Western Cape	West Coast(DC1)	3	7.5%	3.3%	13.6%	N/A	7.7%	3.1%	14.6%	0.8136	19.9%	11.2%	30.5%	N/A
Gauteng	West Rand(DC48)	3	18.7%	10.2%	29.8%	N/A	4.9%	1.6%	10.4%	0.3999	19.3%	10.5%	30.5%	N/A
Free State	Xhariep(DC16)	3	9.7%	4.5%	17.1%	N/A	4.8%	1.6%	10.1%	0.3945	12.3%	6.0%	20.5%	N/A
KwaZulu-Natal	Zululand(DC26)	3	12.8%	7.1%	20.0%	N/A	3.6%	1.3%	7.4%	0.1669	25.9%	17.4%	36.1%	N/A
KwaZulu-Natal	eThekwini(ETH)	3	9.5%	5.7%	14.5%	N/A	4.9%	2.3%	8.6%	0.4256	26.5%	19.6%	33.9%	N/A
KwaZulu-Natal	iLembe(DC29)	3	16.1%	9.0%	25.4%	N/A	4.4%	1.5%	9.4%	0.3189	22.0%	13.0%	32.6%	N/A
Eastern Cape	Alfred Nzo(DC44)	4	14.7%	8.2%	23.1%	N/A	2.8%	0.9%	6.3%	0.0755	13.5%	7.3%	21.5%	N/A
KwaZulu-Natal	Amajuba(DC25)	4	8.0%	4.5%	12.6%	N/A	3.9%	1.0%	10.1%	0.2334	10.7%	6.4%	16.0%	N/A
Eastern Cape	Amathole(DC12)	4	9.3%	3.9%	17.8%	N/A	6.9%	1.6%	17.7%	0.5961	22.2%	11.3%	36.2%	N/A
North West	Bojanala(DC37)	4	8.9%	4.5%	15.0%	N/A	4.9%	1.3%	12.6%	0.3855	8.9%	4.5%	14.9%	N/A
Eastern Cape	Buffalo City(BUF)	4	10.0%	3.9%	19.9%	N/A	8.2%	1.6%	25.1%	0.6461	25.5%	12.5%	42.7%	N/A
Eastern Cape	Cacadu(DC10)	4	9.0%	4.4%	15.7%	N/A	4.2%	1.4%	8.7%	0.2758	26.5%	16.8%	38.0%	N/A
Western Cape	Cape Winelands(DC2)	4	6.2%	3.0%	10.7%	N/A	6.2%	1.6%	15.6%	0.5444	10.6%	5.9%	16.5%	N/A
Limpopo	Capricorn(DC35)	4	9.6%	5.1%	15.5%	N/A	3.4%	1.3%	6.9%	0.1341	11.8%	6.7%	18.5%	N/A
Western Cape	Central Karoo(DC5)	4	8.6%	4.1%	15.1%	N/A	4.6%	1.6%	9.4%	0.3614	7.9%	3.6%	14.2%	N/A
Eastern Cape	Chris Hani(DC13)	4	9.1%	4.7%	14.9%	N/A	3.8%	1.5%	7.6%	0.1996	15.9%	9.5%	23.7%	N/A
Western Cape	City of Cape Town(CPT)	4	8.5%	3.0%	18.8%	N/A	7.0%	3.3%	12.2%	0.8015	15.4%	9.5%	22.5%	N/A
Gauteng	City of Johannesburg(JHB)	4	6.6%	3.5%	10.7%	N/A	8.4%	4.6%	13.4%	0.9533	8.0%	4.5%	12.6%	N/A
Gauteng	City of Tshwane(TSH)	4	4.5%	2.1%	7.9%	N/A	3.7%	1.5%	7.0%	0.1582	6.0%	3.1%	10.1%	N/A
North West	Dr Kenneth Kaunda(DC40)	4	6.9%	2.8%	13.1%	N/A	5.4%	1.8%	11.6%	0.4939	8.0%	3.5%	15.0%	N/A
North West	Dr Ruth Segomotsi Mompati(DC39)	4	8.0%	4.2%	13.3%	N/A	7.0%	3.3%	12.2%	0.8128	5.2%	2.5%	9.2%	N/A
Western Cape	Eden(DC4)	4	9.0%	3.3%	19.0%	N/A	6.6%	1.6%	17.3%	0.5772	13.2%	6.1%	23.0%	N/A
Mpumalanga	Ehlanzeni(DC32)	4	6.6%	3.5%	10.8%	N/A	3.4%	1.4%	6.4%	0.1089	12.5%	7.6%	18.3%	N/A
Gauteng	Ekurhuleni(EKU)	4	4.4%	2.0%	7.8%	N/A	6.5%	3.0%	11.5%	0.7371	9.3%	5.0%	15.0%	N/A
Free State	Fezile Dabi(DC20)	4	6.7%	2.8%	12.8%	N/A	5.2%	1.3%	13.4%	0.4179	21.5%	11.8%	33.9%	N/A
Northern Cape	Frances Baard(DC9)	4	10.9%	6.1%	17.1%	N/A	3.4%	1.3%	7.0%	0.139	7.2%	3.6%	12.2%	N/A
Mpumalanga	Gert Sibande(DC30)	4	12.5%	7.3%	19.1%	N/A	4.7%	2.0%	9.0%	0.3821	13.1%	7.7%	19.9%	N/A
Limpopo	Greater Sekhukhune(DC47)	4	11.9%	7.2%	17.6%	N/A	5.4%	2.5%	9.6%	0.5501	5.8%	3.0%	9.6%	N/A
Eastern Cape	Joe Gqabi(DC14)	4	16.4%	9.4%	25.3%	N/A	3.2%	1.1%	6.9%	0.1167	13.4%	7.3%	21.2%	N/A
Northern Cape	John Taolo Gaetsewe(DC45)	4	9.7%	5.2%	16.0%	N/A	8.4%	3.9%	14.6%	0.9098	6.5%	3.1%	11.4%	N/A
Free State	Lejweleputswa(DC18)	4	13.2%	7.4%	20.8%	N/A	5.4%	2.2%	10.4%	0.5142	9.4%	4.8%	15.4%	N/A
Free State	Mangaung(MAN)	4	8.5%	3.7%	15.8%	N/A	5.4%	1.8%	11.8%	0.4869	15.0%	7.7%	24.9%	N/A
Limpopo	Mopani(DC33)	4	6.9%	3.3%	12.0%	N/A	5.3%	2.1%	10.3%	0.5005	8.7%	4.4%	14.5%	N/A
Northern Cape	Namakwa(DC6)	4	7.6%	3.4%	13.9%	N/A	7.1%	2.8%	13.8%	0.7477	8.7%	4.1%	15.4%	N/A

Eastern Cape	Nelson Mandela Bay(NMA)	4	10.6%	5.5%	17.6%	N/A	4.2%	1.0%	11.9%	0.282	18.9%	11.3%	28.4%	N/A
North West	Ngaka Modiri Molema(DC38)	4	9.4%	5.2%	15.0%	N/A	7.1%	3.4%	12.3%	0.8173	10.9%	6.3%	17.0%	N/A
Mpumalanga	Nkangala(DC31)	4	5.7%	2.6%	10.2%	N/A	5.1%	1.4%	12.8%	0.3983	15.5%	9.0%	23.7%	N/A
Eastern Cape	O.R.Tambo(DC15)	4	8.6%	4.9%	13.3%	N/A	3.8%	0.9%	10.4%	0.2255	24.9%	18.4%	32.3%	N/A
Western Cape	Overberg(DC3)	4	6.3%	2.9%	11.2%	N/A	3.1%	1.0%	6.6%	0.1012	20.5%	12.8%	29.5%	N/A
Northern Cape	Pixley ka Seme(DC7)	4	7.6%	3.2%	14.2%	N/A	4.9%	1.7%	10.6%	0.4143	14.2%	7.0%	24.3%	N/A
Gauteng	Sedibeng(DC42)	4	8.7%	4.4%	14.6%	N/A	3.4%	1.2%	7.1%	0.1509	12.9%	7.2%	20.4%	N/A
KwaZulu-Natal	Sisonke(DC43)	4	7.0%	3.7%	11.5%	N/A	2.5%	0.9%	5.2%	0.0337	10.1%	5.6%	15.7%	N/A
Northern Cape	Siyanda(DC8)	4	9.0%	4.8%	14.9%	N/A	6.8%	3.0%	12.3%	0.761	5.0%	2.2%	9.0%	N/A
Free State	Thabo Mofutsanyane(DC19)	4	6.9%	3.0%	12.8%	N/A	4.8%	1.3%	12.3%	0.3625	11.8%	5.9%	20.0%	N/A
KwaZulu-Natal	UMgungundlovu(DC22)	4	6.4%	3.2%	10.9%	N/A	2.3%	0.8%	4.9%	0.0216	14.8%	9.1%	21.7%	N/A
KwaZulu-Natal	Ugu(DC21)	4	7.3%	4.1%	11.4%	N/A	2.9%	1.2%	5.6%	0.0537	18.0%	12.4%	24.6%	N/A
KwaZulu-Natal	Umkhanyakude(DC27)	4	7.4%	3.6%	12.4%	N/A	3.8%	1.0%	10.2%	0.23	14.1%	8.2%	21.7%	N/A
KwaZulu-Natal	Umzinyathi(DC24)	4	8.8%	3.3%	18.9%	N/A	3.6%	0.9%	9.8%	0.1991	15.2%	5.8%	30.8%	N/A
KwaZulu-Natal	Uthukela(DC23)	4	9.6%	6.4%	13.3%	N/A	5.0%	2.8%	7.8%	0.4454	14.7%	10.8%	19.4%	N/A
KwaZulu-Natal	Uthungulu(DC28)	4	11.4%	6.9%	17.2%	N/A	3.2%	1.3%	6.4%	0.093	18.9%	12.6%	26.1%	N/A
Limpopo	Vhembe(DC34)	4	13.5%	7.8%	20.7%	N/A	2.4%	0.7%	5.2%	0.0304	8.3%	4.2%	13.8%	N/A
Limpopo	Waterberg(DC36)	4	8.2%	4.4%	13.1%	N/A	3.5%	1.4%	6.7%	0.1388	7.3%	3.8%	12.0%	N/A
Western Cape	West Coast(DC1)	4	5.2%	2.2%	9.9%	N/A	4.5%	1.6%	9.5%	0.34	8.2%	3.7%	14.7%	N/A
Gauteng	West Rand(DC48)	4	6.2%	2.6%	11.9%	N/A	4.1%	1.3%	8.9%	0.2656	11.4%	5.4%	19.8%	N/A
Free State	Xhariep(DC16)	4	13.4%	6.9%	22.1%	N/A	4.8%	1.3%	12.4%	0.3584	8.6%	3.9%	15.1%	N/A
KwaZulu-Natal	Zululand(DC26)	4	8.3%	4.5%	13.2%	N/A	2.9%	1.1%	5.9%	0.0707	17.7%	11.5%	25.0%	N/A
KwaZulu-Natal	eThekwini(ETH)	4	12.7%	8.2%	17.9%	N/A	2.0%	0.8%	4.1%	0.0055	29.2%	22.4%	36.5%	N/A
KwaZulu-Natal	iLembe(DC29)	4	7.0%	3.6%	11.6%	N/A	2.8%	1.0%	5.7%	0.0561	15.0%	9.3%	22.0%	N/A
Eastern Cape	Alfred Nzo(DC44)	5	6.6%	2.7%	12.7%	0.958	3.6%	1.1%	8.1%	0.1844	15.1%	7.7%	25.1%	0.0655
KwaZulu-Natal	Amajuba(DC25)	5	8.9%	4.4%	15.0%	0.6443	3.1%	1.1%	6.7%	0.1028	11.1%	5.9%	18.0%	0.2338
Eastern Cape	Amathole(DC12)	5	8.2%	3.1%	17.0%	0.669	6.1%	1.6%	15.4%	0.5297	19.6%	8.5%	35.6%	0.6116
North West	Bojanala(DC37)	5	8.7%	3.9%	16.4%	0.3584	4.6%	1.2%	12.2%	0.3387	11.6%	5.4%	20.5%	0.6028
Eastern Cape	Buffalo City(BUF)	5	9.3%	3.2%	20.6%	0.6684	7.9%	1.4%	25.4%	0.608	22.8%	9.8%	41.2%	0.0726
Eastern Cape	Cacadu(DC10)	5	8.1%	3.5%	15.3%	0.5055	4.9%	1.3%	12.7%	0.3818	9.0%	4.0%	16.5%	0.2162
Western Cape	Cape Winelands(DC2)	5	9.6%	4.4%	17.0%	0.3039	5.8%	2.2%	11.7%	0.5642	11.2%	5.5%	19.0%	0.1291
Limpopo	Capricorn(DC35)	5	11.9%	5.9%	20.2%	0.6993	3.7%	1.2%	8.1%	0.1959	11.8%	5.8%	20.2%	0.5339
Western Cape	Central Karoo(DC5)	5	7.5%	3.2%	14.4%	0.7188	6.4%	2.3%	13.8%	0.6329	9.9%	4.4%	18.2%	0.2008
Eastern Cape	Chris Hani(DC13)	5	6.2%	2.6%	11.8%	0.8805	3.5%	1.1%	7.7%	0.1712	18.5%	10.3%	28.9%	0.2741
Western Cape	City of Cape Town(CPT)	5	8.3%	3.8%	14.8%	0.5419	6.8%	2.7%	13.4%	0.7178	18.5%	10.4%	28.3%	0.2048
Gauteng	City of Johannesburg(JHB)	5	7.6%	3.7%	13.3%	0.8987	4.3%	1.5%	8.7%	0.2993	9.3%	4.7%	15.7%	0.1068
Gauteng	City of Tshwane(TSH)	5	6.6%	3.2%	11.5%	0.6727	3.5%	1.2%	7.1%	0.1517	8.0%	4.0%	13.4%	0.0405
North West	Dr Kenneth Kaunda(DC40)	5	11.8%	5.3%	21.7%	0.6061	5.0%	1.5%	11.5%	0.4061	10.3%	4.5%	19.2%	0.108
North West	Dr Ruth Segomotsi Mompati(DC39)	5	7.2%	3.2%	13.2%	0.9883	6.0%	2.3%	11.9%	0.5997	7.3%	3.3%	13.4%	0.0628
Western Cape	Eden(DC4)	5	6.8%	2.7%	13.4%	0.7027	6.1%	1.4%	15.7%	0.5276	10.9%	4.8%	20.1%	0.3119
Mpumalanga	Ehlanzeni(DC32)	5	7.2%	3.4%	12.7%	0.9998	3.4%	1.2%	7.1%	0.1361	6.9%	3.2%	12.3%	0.0002
Gauteng	Ekurhuleni(EKU)	5	5.6%	2.4%	10.5%	0.6546	5.9%	2.3%	11.7%	0.6005	11.1%	5.7%	18.6%	0.2311
Free State	Fezile Dabi(DC20)	5	8.9%	3.7%	17.5%	0.6807	4.8%	1.3%	12.4%	0.3609	17.0%	8.0%	29.8%	0.4831
Northern Cape	Frances Baard(DC9)	5	9.5%	4.6%	16.6%	0.3076	4.0%	1.4%	8.6%	0.2516	6.6%	3.0%	12.1%	0.1522
Mpumalanga	Gert Sibande(DC30)	5	9.0%	4.3%	15.9%	0.6795	3.4%	1.1%	7.5%	0.1421	13.6%	7.2%	22.2%	0.0581
Limpopo	Greater Sekhukhune(DC47)	5	6.8%	3.0%	12.3%	0.7849	4.7%	1.7%	9.8%	0.3635	5.9%	2.6%	10.8%	0.0085
Eastern Cape	Joe Gqabi(DC14)	5	7.4%	3.0%	14.0%	0.9632	3.2%	0.9%	7.5%	0.1286	17.3%	8.8%	28.8%	0.0514
Northern Cape	John Taolo Gaetsewe(DC45)	5	7.1%	3.0%	13.7%	0.5346	5.5%	1.8%	11.8%	0.4975	8.4%	3.7%	15.7%	0.0982

Free State	Lejweleputswa(DC18)	5	11.7%	5.7%	20.3%	0.5537	4.1%	1.3%	9.0%	0.2609	10.2%	4.8%	18.0%	0.3243
Free State	Mangaung(MAN)	5	9.8%	4.1%	18.4%	0.8263	4.7%	1.2%	12.4%	0.3582	16.1%	7.5%	28.5%	0.5143
Limpopo	Mopani(DC33)	5	10.0%	4.6%	17.9%	0.9661	5.2%	1.8%	11.2%	0.4653	9.6%	4.4%	17.2%	0.0022
Northern Cape	Namakwa(DC6)	5	8.2%	3.0%	17.9%	0.731	4.9%	1.5%	11.2%	0.4009	8.6%	3.6%	16.5%	0.2484
Eastern Cape	Nelson Mandela Bay(NMA)	5	8.9%	4.0%	16.1%	0.8491	3.2%	0.9%	7.5%	0.1316	17.2%	9.0%	27.6%	0.3095
North West	Ngaka Modiri Molema(DC38)	5	7.0%	3.1%	12.9%	0.95	4.4%	1.5%	9.1%	0.3177	10.1%	4.9%	17.1%	0.0032
Mpumalanga	Nkangala(DC31)	5	8.9%	4.0%	16.6%	0.2224	5.1%	1.8%	11.0%	0.4396	11.1%	5.3%	19.5%	0.1345
Eastern Cape	O.R.Tambo(DC15)	5	10.1%	5.4%	16.4%	0.5924	2.3%	0.7%	5.1%	0.0279	17.9%	11.0%	26.1%	0.0172
Western Cape	Overberg(DC3)	5	9.0%	4.1%	16.1%	0.3381	4.9%	1.2%	12.9%	0.3739	22.0%	12.8%	33.7%	0.0586
Northern Cape	Pixley ka Seme(DC7)	5	9.7%	4.0%	18.7%	0.5992	5.2%	1.4%	13.3%	0.4273	12.3%	4.8%	24.8%	0.311
Gauteng	Sedibeng(DC42)	5	6.4%	2.6%	12.1%	0.7712	6.6%	2.4%	13.3%	0.6657	10.0%	4.6%	17.8%	0.2336
KwaZulu-Natal	Sisonke(DC43)	5	7.7%	3.5%	14.2%	0.887	2.8%	0.8%	6.3%	0.0772	21.4%	12.6%	32.4%	0.1028
Northern Cape	Siyanda(DC8)	5	11.7%	5.9%	19.6%	0.5127	5.2%	1.9%	10.6%	0.4716	6.3%	2.7%	11.8%	0.2676
Free State	Thabo Mofutsanyane(DC19)	5	9.0%	3.7%	17.3%	0.8498	4.4%	1.2%	11.4%	0.3145	12.4%	5.5%	22.6%	0.3373
KwaZulu-Natal	UMgungundlovu(DC22)	5	6.6%	2.9%	12.2%	0.8795	3.8%	1.3%	8.2%	0.2098	17.5%	9.8%	27.3%	0.2441
KwaZulu-Natal	Ugu(DC21)	5	7.6%	3.8%	12.9%	0.9731	3.3%	1.2%	6.7%	0.1131	20.8%	13.3%	29.7%	0.1827
KwaZulu-Natal	Umkhanyakude(DC27)	5	7.8%	3.6%	14.2%	0.6034	4.4%	1.5%	9.7%	0.3244	11.3%	5.5%	19.3%	0.0658
KwaZulu-Natal	Umzinyathi(DC24)	5	6.4%	2.9%	11.5%	0.8143	3.4%	1.2%	7.3%	0.1398	12.9%	6.8%	20.7%	0.1568
KwaZulu-Natal	Uthukela(DC23)	5	7.2%	3.5%	12.5%	0.9174	3.2%	1.1%	6.8%	0.1074	11.4%	6.2%	18.1%	0.0034
KwaZulu-Natal	Uthungulu(DC28)	5	11.1%	5.8%	18.0%	0.7098	3.0%	1.0%	6.5%	0.0927	18.6%	11.3%	27.6%	0.1876
Limpopo	Vhembe(DC34)	5	9.7%	4.5%	17.3%	0.1707	4.3%	1.4%	9.5%	0.3067	9.8%	4.6%	17.2%	0.5039
Limpopo	Waterberg(DC36)	5	6.0%	2.7%	10.9%	0.913	4.1%	1.5%	8.4%	0.2555	10.6%	5.4%	17.8%	0.634
Western Cape	West Coast(DC1)	5	6.6%	2.6%	13.2%	0.4791	4.5%	1.3%	10.6%	0.329	12.7%	5.6%	23.0%	0.1249
Gauteng	West Rand(DC48)	5	6.3%	2.5%	12.9%	0.9742	3.7%	1.1%	8.6%	0.2027	11.4%	5.0%	20.8%	0.0971
Free State	Xhariep(DC16)	5	8.5%	3.6%	16.2%	0.4699	4.4%	1.2%	11.5%	0.3171	10.6%	4.8%	19.2%	0.3661
KwaZulu-Natal	Zululand(DC26)	5	7.6%	3.6%	13.5%	0.7981	2.3%	0.7%	5.2%	0.033	15.9%	8.9%	24.9%	0.0506
KwaZulu-Natal	eThekwini(ETH)	5	6.7%	3.2%	11.5%	0.6792	2.4%	0.8%	5.2%	0.0314	14.7%	8.6%	22.4%	0.0119
KwaZulu-Natal	iLembe(DC29)	5	7.0%	3.2%	12.9%	0.9372	3.2%	0.8%	8.7%	0.1436	18.0%	10.3%	27.9%	0.2679

Supplementary 10: post hoc power analysis

We performed a post hoc power analysis to assess the minimum effect size detectable among infants which has the smallest number of observations. The post hoc power analysis suggests that the sample size in the smallest age group has the power to detect a small effect size (w~0.1 based on Cohens rules of thumb [Cohen, 1988]) when using a chi-square test with 2x9 cells (maximum number of cells tested in our analyses i.e. binary nutritional classification versus province of residence) with 80% power and 5% alpha or type I error.

χ^2 tests - Goodness-of-fit tests: Contingency tables

**Analysis:**	Post hoc: Compute achieved power		
**Input:**	Effect size w	=	0.11
	α err prob	=	0.05
	Total sample size	=	1277
	Df	=	8
**Output:**	Noncentrality parameter λ	=	15.4517000
	Critical χ^2	=	15.5073131
	Power (1-β err prob)	=	0.8133607

Cohen, J (1988) Statistical power analysis for the behavioral sciences (2nd ed.). Hillsdale, NJ: Erlbaum.

A summary guideline for effect size determinations is also provided in Kotrlik, JW and Williams, HA (2003) The incorporation of effect size in information technology, learning, and performance research. *Information Techology, Learning, and Performance Journal* **21(1)** 1-7.

Effect Size	Use	Small	Medium	Large
Correlation inc Phi		0.1	0.3	0.5
Cramer's V	r x c frequency tables	0.1	0.3	0.5
Difference in arcsines	Comparing two proportions	0.2	0.5	0.8
η^2	Anova	0.01	0.06	0.14
omega-squared	Anova; See Field (2013)	0.01	0.06	0.14
Multivariate eta-squared	one-way MANOVA	0.01	0.06	0.14
Cohen's f	one-way an(c)ova (regression)	0.1	0.25	0.4
η^2	Multiple regression	0.02	0.13	0.26
κ^2	Mediation analysis	0.01	0.09	0.25
Cohen's f	Multiple Regression	0.14	0.39	0.59
Cohen's d	t-tests	0.2	0.5	0.8
Cohen's ω	chi-square	0.1	0.3	0.5
Odds Ratios	2 by 2 tables	1.5	3.5	9
Odds Ratios	p vs 0.5	0.55	0.65	0.75
Average Spearman rho	Friedman test	0.1	0.3	0.5
