# Supplementary material for: Spatial-temporal trends and risk factors for undernutrition and obesity among children (<5 years) in South Africa, 2008–2017: findings from a nationally representative longitudinal panel survey
Source: BMJ Open. 2020 Apr 8;10(4):e034476. doi: 10.1136/bmjopen-2019-034476 (PMC7245449; doi:10.1136/bmjopen-2019-034476)
Supplement: Author's manuscript [file bmjopen-2019-034476.draft_revisions.pdf]

BMJ Open

BMJ Open is committed to open peer review. As part of this commitment we make the peer review history of every article we publish publicly available.

When an article is published we post the peer reviewers' comments and the authors' responses online. We also post the versions of the paper that were used during peer review. These are the versions that the peer review comments apply to.

The versions of the paper that follow are the versions that were submitted during the peer review process. They are not the versions of record or the final published versions. They should not be cited or distributed as the published version of this manuscript.

BMJ Open is an open access journal and the full, final, typeset and author-corrected version of record of the manuscript is available on our site with no access controls, subscription charges or pay-per-view fees (http://bmjopen.bmj.com).

If you have any questions on BMJ Open's open peer review process please email info.bmjopen@bmj.com

Spatial-temporal trends and risk factors for under-nutrition and obesity among children (<5 years) in South Africa, 2008-2017: findings from a nationally representative longitudinal panel survey

Journal:	*BMJ Open*
Manuscript ID	bmjopen-2019-034476
Article Type:	Original research
Date Submitted by the Author:	26-Sep-2019

Complete List of Authors:	Sartorius, Benn; London School of Hygiene and Tropical Medicine Faculty of Infectious and Tropical Diseases, Disease Control<br>Sartorius, Kurt; University of KwaZulu-Natal, Public Health Medicine, School of Nursing and Public Health<br>Green, Rosemary; London School of Hygiene and Tropical Medicine, 2 Department of Population Health<br>Lutge, Elizabeth; South African Government Department, KwaZulu Natal Department of Health<br>Scheekbeek, Pauline; London School of Hygiene & Tropical Medicine, Epidemiology and Population Health<br>Tanser, Frank; Africa Centre for Health and Population Studies; University of KwaZulu-Natal<br>Dangour, Alan; London School of Hygiene & Tropical Medicine, Dept of Population Health<br>Slotow, Rob; University of KwaZulu-Natal

Keywords:	PUBLIC HEALTH, Community child health < PAEDIATRICS, NUTRITION & DIETETICS

SCHOLARONE™
Manuscripts

I, the Submitting Author has the right to grant and does grant on behalf of all authors of the Work (as defined in the below author licence), an exclusive licence and/or a non-exclusive licence for contributions from authors who are: i) UK Crown employees; ii) where BMJ has agreed a CC-BY licence shall apply, and/or iii) in accordance with the terms applicable for US Federal Government officers or employees acting as part of their official duties; on a worldwide, perpetual, irrevocable, royalty-free basis to BMJ Publishing Group Ltd ("BMJ") its licensees and where the relevant Journal is co-owned by BMJ to the co-owners of the Journal, to publish the Work in this journal and any other BMJ products and to exploit all rights, as set out in our licence.

The Submitting Author accepts and understands that any supply made under these terms is made by BMJ to the Submitting Author unless you are acting as an employee on behalf of your employer or a postgraduate student of an affiliated institution which is paying any applicable article publishing charge ("APC") for Open Access articles. Where the Submitting Author wishes to make the Work available on an Open Access basis (and intends to pay the relevant APC), the terms of reuse of such Open Access shall be governed by a Creative Commons licence – details of these licences and which Creative Commons licence will apply to this Work are set out in our licence referred to above.

Other than as permitted in any relevant BMJ Author's Self Archiving Policies, I confirm this Work has not been accepted for publication elsewhere, is not being considered for publication elsewhere and does not duplicate material already published. I confirm all authors consent to publication of this Work and authorise the granting of this licence.

For peer review only

Title page

Spatial-temporal trends and risk factors for under-nutrition and obesity among children (<5 years) in South Africa, 2008-2017: findings from a nationally representative longitudinal panel survey

Running title: under-nutrition and obesity among children in South Africa

Sartorius B[1-3]*, Sartorius K[2,4], Green R[5], Lutge E[2,6], Scheelbeek P[5], Tanser F[2,7-9], Dangour AD[5], Slotow R[10,11]

1. Department of Disease Control, London School of Hygiene & Tropical Medicine, London, UK
2. Department of Public Health Medicine, School of Nursing and Public Health, University of KwaZulu-Natal, Durban, South Africa
3. Department of Health Metrics Sciences, School of Medicine, University of Washington, Seattle, USA
4. Faculty of Commerce, Law and Management, University of the Witwatersrand, Johannesburg, South Africa
5. Department of Population Health, London School of Hygiene & Tropical Medicine, London, UK
6. Health Research and Knowledge Management, KwaZulu-Natal Department of Health, KwaZulu-Natal, South Africa
7. Africa Health Research Institute (AHRI), KwaZulu-Natal, South Africa
8. Centre for the AIDS Programme of Research in South Africa (CAPRISA), University of KwaZulu-Natal, Durban, South Africa
9. Institute of Epidemiology and Health Care, University College London, London, United Kingdom
10. School of Life Sciences, University of Kwazulu-Natal, Pietermaritzburg Campus, South Africa
11. Department of Genetics, Evolution and Environment, University College, London, UK

*Corresponding author: Dr Benn Sartorius; Address: Department of Disease Control, London School of Hygiene and Tropical Medicine, Keppel Street, Bloomsbury, London, WC1E7HT, United Kingdom; Tel.: +44 (0)207 927 2164; E-mail: benn.sartorius1@lshtm.ac.uk

Word count: 4095

For peer review only

Summary

Objectives: To assess space-time trends in the burden of malnutrition and associated risk factors among children under 5 years of age in South Africa

Design: national panel survey

Setting: national, community based

Participants: Community based sample of children and adults. Sample size: 3,254 children in wave 1 (2008) to 4,710 children in wave 5 (2017).

Primary outcomes: Stunting, wasting/thinness and obesity among children (<5). Classification were based on anthropometric (height and weight) z-scores using WHO growth standards.

Results: Between 2008 and 2017 there was a significant decline nationally in stunting prevalence among children under 5 years of age from 11.0% to 7.6% (p=0.007), while thinness/wasting (5.2% to 3.8%, p=0.131) and obesity (14.5% to 12.9%, p=0.312) decreased insignificantly. Stunting prevalence appears relatively evenly spread across South Africa, while obesity is more pronounced in the east of the country and thinness/wasting more pronounced in the west. Only 16/ 52 districts had an estimated wasting prevalence below the 2025 target threshold of 5% in 2017.African ethnicity, male gender, low birth weight, lower socio-economic and maternal/paternal education status and residence in a rural area were significantly associated with stunting. Children living in a lower income and food insecure household with young malnourished mothers were significantly more likely to be thin/wasted while African children, with higher birth weights, living in lower income households in KwaZulu-Natal and Eastern Cape were significantly more likely to be obese.

Conclusions: While improvements in stunting have been observed, thinness/wasting and obesity prevalence remain largely unchanged. The geographic and socio-demographic heterogeneity in childhood malnutrition has implications for equitable attainment of global nutritional targets for 2025. Many districts appeared to have dual epidemics of under and over nutrition (high within district heterogeneity and inequality). Effective public health planning and tailored interventions are required at the sub-national level to address this challenge.

Keywords: nutritional status, nutritional transition, undernutrition, obesity, children, South Africa

Strengths and limitations of this study

For peer review only

- Utilises data from a nationally representative repeated panel data at individual/household level over a 10-year period (5 survey waves).
- Employed a fully Bayesian space-time shared component model to produce more stable estimates of malnutrition burden at provincial and district level among children under five years of age in South Africa.
- Panel design allows assessment of change in malnutrition burden within the same individuals/households observed at multiple time points.
- Missing or invalid weight/height measurements may have introduced selection bias if not missing at random, and may thus have affected both the internal validity and the representativeness the findings.
- As primary panel study was not designed/powered for provincial and lower geographic level analysis, we cannot discount the resultant impact on precision/random variability when analysing at provincial/district level (administrative tier just below province) and further stratification by socio-demographic correlates.

Background

Despite reductions in malnutrition 150.8 million children (22.2%) under five are stunted and a further 50.5 million children are wasted [1]. Furthermore rapidly rising trend in overweight and obesity in children and adults [2-4] [5] has emerged as one of the most serious global public health issues of the 21st century [6]. Sub-Saharan Africa (SSA) has among the highest levels of child malnutrition[1] globally. This problem is particularly illustrated by South Africa [7], a middle income country with high levels of wealth/economic inequality that is undergoing rapid socioeconomic and lifestyle changes that have precipitated a nutritional transition, high prevalence of overweight/obesity in children [8]. The dual burdens of undernutrition and overweight/obesity are not distributed in a spatially homogenous manner [9], and the health risks associated with malnutrition vary by age, gender, ethnicity and geographical location [10].

Progress to tackle all forms of child malnutrition remain much too slow [1]. In order to support the delivery of public health interventions that will be most effective at reducing malnutrition, an understanding of the geographical distribution of malnutrition is required. Limited data are collected at lower administrative unit level making it difficult to identify specific groups of high-risk individuals and thus, determine the most suitable and

---

[1] Child malnutrition is defined as a pathological state as a result of inadequate nutrition, including undernutrition due to insufficient intake of dietary energy and other key nutrients resulting in stunting (low height for age) or wasting (low weight-for-length) and overweight and obesity due to excessive consumption of dietary energy and reduced levels of physical activity.

For peer review only

cost-effective opportunities and solutions. Previous studies of nutritional status of the South African population have mostly focused on adults [11 12]. Here we use a large, nationally-representative data from multiple rounds of the National Income Dynamics Study over the period 2008 to 2017 to assess space-time trends in the burden of malnutrition and associated risk factors among children under 5 years of age in South Africa.

Methods

Data

Data were taken from the five panel (cross-sectional) waves of the South African National Income Dynamics Study (SA-NIDS)[13], the first national panel study in South Africa. SA-NIDS was undertaken by the South African Labour and Development Research Unit based at the School of Economics at the University of Cape Town. The surveys took place in 2008, 2010-11, 2012, 2014-15 and 2017. These are named waves 1-5 respectively. A detailed description of the data collection methods can be found elsewhere [26]. In short, a stratified, two-stage random cluster sample design was employed to sample households for inclusion at baseline using proportionally allocated stratification, based on the 52 district councils (DCs) in South Africa. Within each DC (primary sampling unit [PSU]), clusters of dwelling units were systematically drawn [14]. The household level response rate was 69% and the individual response rate within households was 93%. Survey enumerators attempt to collect weight and height measurements of all individuals (including children) in selected households.

Study population

We restricted our analysis to children <5 years of age.

Outcomes

We calculated height for age (HA) and BMI-for-age (BA) z-scores using the WHO 2007 growth standards [15 16]. We generated z-scores by transformation of child anthropometric data using the “lambda mu sigma” method (‘zanthro’ function in Stata 15). As recommended, weight-for-length was used in children 0 to <2 years of age, and BMI-for-age in children 2 years of age and older [17]. We defined obesity as weight-for-length z-score ≥+2 for children under 2 years of age and BMI for age z-score of >2+ for children age 2 and older [17]. We defined wasting as weight-for-length z-score < -2 for children under 2 years of age and thinness as BMI for age z-score < -2 for children 2 years and older. Stunting was defined as HA z-score of < -2.

Geographic and socio-demographic variables

For peer review only

To identify relevant inequalities under-nutrition and obesity indicators were stratified temporally (survey year), geographically (province and residence location type: urban informal settlements, urban formal, tribal/rural) and by important socio-demographic categories (Gender: Female/Male; ethnicity: Black/African, Coloured, Indian/Asian, White/Caucasian; Maternal: age; education status; body mass index; household socio-economic status (income) classified into quantiles [1=lowest, 5=highest].

Data analysis

Analyses were performed using Stata software version 15 [StataCorp. 2017. Stata Statistical Software: Release 15. College Station, TX: StataCorp LLC]. Clustering, as well as survey design effects, were accounted for using sample weights to estimate standard error and 95% confidence intervals (CIs) around mean anthropometric z-score point estimates, both overall and stratified by other socio-demographic variables such ethnicity and gender, socio-economic status, and residence location type. Extrapolated population totals of malnourished children (< 5) by yearly age were estimated using the survey weights.

Space-time Bayesian modelling: Furthermore, we employed a Bayesian joint (shared component) space-time binomial model [18] to estimate stable malnutrition prevalence rates at provincial and district levels across the 5 waves. The model splits the risk of malnutrition into three spatio-temporal components: a shared component for all three malnutrition types (stunting, thinness/wasting and obesity) and two additional components that capture that unshared differences between the three types. The model formulation contains an additive decomposition for the shared part, space–time interaction terms common to the three malnutrition types and additional heterogeneity terms. This methodology was employed in an attempt to stabilise estimates at district level given that the primary sampling design was not developed to provide point estimates at this level of geographic disaggregation. Survey weighted prevalence's were applied to sample size totals by district and panel to obtain a survey weighted numerator count by malnutrition type in the binomial distribution. The joint space-time was fitted in WINBUGS using Markov chain Monte Carlo (MCMC) simulation and non-informative priors. The full model code is provided in the Supplementary Material (1). The model was run until the Monte Carlo error for each parameter of interest was less than 5% of the sample standard deviation. Posterior prevalence estimates (and 95% Bayesian credibility intervals) of undernutrition and obesity levels at provincial and district level were mapped using ArcGIS 10.6.1 [ESRI 2011. ArcGIS Desktop: Release 10. Redlands, CA: Environmental Systems Research Institute].

For peer review only

Risk factors analysis: Two-way tabulations of key socio-demographic covariates, year and child nutritional status were performed using the 'svy: tab' function to produce survey weighted prevalence estimates. Tests of independence for complex survey data survey (weighted Pearson's chi-square test) was utilised to assess the significance of bivariate associations between malnutrition burden and year as well as socio-demographic covariates.

Ethical approval: Approval for the primary study was granted by the Ethics Committee of the University of Cape Town. The current analysis is a secondary data analysis of an open access dataset and does not require further ethical approval.

Patient and Public Involvement: As this was a data analysis utilising secondary data from a national community based panel survey, the development of the research question was not informed by the study subjects. Likewise, we could not involve study participants in the design of this study. Study participants were not involved in conduct of the primary study. Results will be disseminated in the form of peer reviewed article as well as through presentation to senior members of our National Department of Health and KwaZulu-Natal Department of Health.

Results

Study population

The sample of children <5 years of age in the 7,301 households included in the SA-NIDS survey increased from 3,254 children at baseline (2008) to 4,710 children in wave 5 (2017) (Supplementary Material 2). With the exception of children under 1 year of age and survey wave 2 in 2010/11, valid weight and height measurements were taken from 85-90% of children sampled between the age of 1 and 5 on average.

Temporal changes in burden of malnutrition from 2008 to 2017)

Between 2008 and 2017, the prevalence of stunting among children aged under 5 years decreased significantly from 11.0% to 7.6% (p=0.007) (Table 1). Over the same period, both the prevalence of wasting/thinness (and the prevalence of obesity decreased non-significantly (from 5.2 to 3.8%, p= 0.131 and 14.5% to 12.9%, p= 0.312 respectively). The prevalence of thinness was significantly (p<0.001) higher in children under 2 years of age (8% in 2008; 6% in 2017) compared to 4% in 2008 and 3% in 2017 among children 2 years and older. The prevalence of obesity was also significantly (p<0.001) higher among children under 2 years of age and increased over the study period (18.4% in 2008 vs 21.7 in 2017, p=0.331).

For peer review only

Space-time burden of malnutrition at provincial and district level

Under nutrition: In 2008 and 2017, the highest prevalence of stunting was estimated in the Free State (17.1% and 10.5% respectively) followed by Eastern Cape (14.7% and 8.5% respectively) and Limpopo (14.0% and 9.3% respectively) (Figure 1 – panel a1). One district respectively in Free State (Mangaung Metropolitan), Limpopo (Vhembe) and Northern Cape (Pixley ka Seme) had an estimated stunting prevalence in excess of 15% in 2017 (Figure 1– panel a2). Gauteng had the highest burden of thinness/wasting in 2008 (9.6%) followed by North West province (9.3%) and Free State (7.6%) (Figure 2a). By 2017 2017 the highest burden was observed in Western Cape (at 5.6%) followed by Northern Cape (4.9%) and North West (4.6%) (Figure 2b). The estimates suggest that at provincial level 7 of 9 provinces were above the 5% target threshold for wasting in 2017 (only Eastern Cape and KwaZulu-Natal were the exceptions). There appeared to be a general gradient of higher burden of thinness/wasting in the western half of country in 2017 (lower burden in KwaZulu-Natal and northern districts of Eastern Cape) (Figure 2b). The three highest wasting prevalence districts in 2017 were Amathole [EC] (12.6%), Siyanda [NC] (11.4%) and Eden [WC] (10.9%) (Figure 2b). Similarly, to the provincial level finding above, only 16 of 52 districts had an estimated wasting prevalence below 5% in 2017.

Obesity: In 2008, the highest prevalence of obesity was estimated in Eastern Cape (22.5%) followed by Western Cape (18.4%) and KwaZulu-Natal (17.6%) (Figure 3a). A decade later in 2017, the highest prevalence of childhood obesity was still estimated to be in the Eastern Cape (15.6%), followed by KwaZulu-Natal (15.1%)and Western Cape (15.0%). In contrast to the wasting gradient highlighted above (higher burden in the western half of the country), the burden of obesity in 2017 appeared to be much higher in the eastern half of the country (particularly KwaZulu-Natal and Eastern Cape) (Figure 3b). The 4 highest obesity prevalence districts in 2017 were located in KwaZulu-Natal (Sisonke [26.2%] and eThekwini Metropolitan [25.7%) and Eastern Cape (Buffalo City Metropolitan [29.1%] and O.R Tambo [25.9%]).

Figure 1: Bayesian posterior prevalence by province (and wave) and district level prevalence (equal intervals, 2017) of stunting among children <5 years

Figure 2: Bayesian posterior prevalence by province (and wave) and district level prevalence (equal intervals, 2017) thinness/wasting among children <5 years

Figure 3: Bayesian posterior prevalence by province (and wave) and district level prevalence (equal intervals, 2017) of obesity among children <5 years

For peer review only

Factors associated with child nutritional status

A bivariate analysis of demographic, maternal, socio-economic and household factors at individual nutritional status level suggests that African ethnicity (p<0.001), male gender (p=0.002), low birth weight (<0.001), residing in lower socio-economic status household (p<0.001), province of residence (p=0.012), lower maternal/paternal education status (p<0.001, 0.020 respectively) and residence in a rural/tribal authority area (p<0.001) were significantly associated with stunting (Table 2). Children living in lower income households (p=0.053), lower food security (as measured through child hunger in last year) (p<0.001), province of residence (p=0.002), having a younger mother (<20) (p=0.012) and mother having a lower BMI classification (p=0.005) was significantly associated with thinness/wasting status. Children of African ethnicity (p<0.001), higher birth weight (p=0.006), living in lower income households (p=0.001) in KwaZulu-Natal and Eastern Cape (p<0.001) as well as paternal educational attainment (p=0.033) were significantly associated with obesity status (Table 2).

Discussion

Main findings*:* The present study illustrates that while stunting has declined among South African children over the last 10 years, wasting and obesity appear largely unchanged, suggesting that development and public health interventions have had a variable impact. Stunting prevalence appears relatively evenly spread across South Africa, but obesity burden is more pronounced in the east of the country, whereas thinness/wasting is more pronounced in the west, with only 16 of 52 districts with estimated wasting prevalence below the 5% (WHO 2025 target threshold) in 2017. A concerning pattern observed was the increase prevalence of obesity in children under the age of two years. Key socio-demographic factors associated with malnutrition status were identified which likely underpins the spatial patterns (and heterogeneity) observed across the country. African children with lower birth weights residing in lower income households in rural areas with less educated mothers and fathers were particular more likely to be stunted. Children in lower income, food insecure households with malnourished young mothers appeared particularly more likely to be thin/wasted while African children, with higher birth weights, living in lower income households in KwaZulu-Natal and Eastern Cape were also more likely to be obese.. Furthermore, low household income appeared to be positively associated with all 3 nutritional types. Declining childhood stunting rates from 2008-2017 may well have resulted from government initiatives to support food security and child health (among other things), but our findings of distinct geographic and socio-demographic variability in undernutrition and obesity rates suggest that tacking malnutrition in South Africa is complex.

For peer review only

Models and targets for nationally-driven intervention need to be carefully specified according to local environments and socio-economic profiles.

Contribution to existing literature*:* Two previous studies in South Africa among primary school aged children dating back 25+ years (1993and 1994 respectively) utilised cross sectional data[19 20], thus limiting insight into temporal trends. Furthermore, the study by Jinabhai et al. [19] was restricted to KwaZulu-Natal limiting national representativeness. Another cross sectional study in South African in 2001-2003 among primary school children in five South African Provinces suggested that relative to 1993 prevalence of undernutrition had decreased while obesity had increased[20 21]. Thus these previous data are now outdated, were largely focused on primary school aged children as well as cross sectional in nature and geographically restricted.

This is also the first spatial-temporal Bayesian shared component analysis of malnutrition trends among children in South Africa utilising geographically representative repeated panel data over a 10-year period. The current study focusing on children under 5 year of age suggests that there is prominent geographic heterogeneity in malnutrition burden in South Africa in this youngest age group. This is in line with findings from other settings in Africa that have documented similar spatial heterogeneity [22] and persistence of these malnutrition inequalities has been demonstrated in an 80 country study further highlighting this ongoing public health conundrum [23 24]. Our results demonstrate a strong west to east gradient of higher underweight burden on the western side of South Africa and greater obesity on the eastern seaboard (Eastern Cape and KwaZulu-Natal). A map of poverty and inequality in South Africa [2] illustrates the co-existence of high levels of poverty and inequality in many parts of KwaZulu-Natal and the Eastern Cape with high levels of overweight/obesity. This is further confirmed by our individual child level analysis which suggested a significantly higher obesity prevalence in lower income households. Metropolitan areas displayed high levels of nutritional inequality that complement national studies of poverty and inequality [25].

Under and over nutrition status appeared positively associated with lower household income classification. This finding of stunting and wasting disproportionately affecting the poor has been often demonstrated [26]. Other studies in Africa in particular have documented similar patterns i.e. children living in low SES households, children who live in peripheral areas and whose mothers had little or no schooling were at significantly higher risk of malnutrition [27]. The inconsistent challenges facing health authorities are occurring in the face of rapid urbanization

[2] https://southafrica-info.com/people/mapping-poverty-in-south-africa/

For peer review only - http://bmjopen.bmj.com/site/about/guidelines.xhtml

and industrialization that simultaneously attract both the rich and the poor to live in the same geographic districts [28].The heterogeneous geographic relationship between household income and undernutrition is also affected by the allocation of household income that is a function of maternal education, access to markets, infrastructure and sanitation [29]. Additionally, these data suggest that there is a strong and highly significant association between higher food insecurity (child hunger frequency in the preceding year) and increased thinness/wasting. Community and government based packages of support need to be highly targeted to the poorest and most food insecure households to further reduce inequality in this regard and maximise reductions in malnutrition.

Our findings suggest that children with low birth weight (due to pre-term delivery, fetal/intrauterine growth restriction or a combination of the two) were significantly more likely to be stunted than normal weight babies and this has been demonstrated in many other low and middle income settings (for example [30]). Socioeconomic status/factors are known risk factors for LBW [31]and may in part explain the significant association found between stunting and lower household income. South Africa has the higher number of incident and prevalent HIV infections globally [32]. A further important contextual risk factor for LBW is maternal HIV status. A systematic review and large observational studies focussing on low and middle incoming countries, suggest a strong and significant association between maternal HIV infection and LBW [33 34]. Evidence from South Africa also suggests the anthropometric z-score of HIV-infected children appear to be consistently lower when compared to HIV-exposed but uninfected children [35]. We also observed a significantly higher prevalence of stunting among male children which has been demonstrated previously in a meta-analysis for sub-Saharan Africa [36], the suggested cause of which might be that male children are more vulnerable to health inequalities relative to female children of the same age. Strengthening community-based packages of care and community health worker (CHW) performance/skills in rural and high burden geographies are key strategies to improve primary health care delivery through better identification of women at higher risk of poor birth outcomes (e.g. HIV positive, history of previous poor birth outcomes and/or currently malnourished), higher referral rates for facility births, and improved linkage to other health as well as social services [37]. Lastly given the high adolescent fertility rates in many parts of South Africa [38], there is also much scope to improve CHW identification of households with higher risk malnourished adolescent girls prior to pregnancy to ensure more optimal linkage to government and social support to ensure adequate nutrition as well as improved awareness regarding family planning practices e.g. ensuring adequate birth spacing [39].

Obesity in children has a complex aetiology that includes a wide range of socioeconomic, demographic, environmental and cultural variables [40]such as household composition, mother's education, household income,

For peer review only

household size, environmental factors, rural versus urban location, and sanitation [9 41]. The high burden of obesity is likely associated with a progressive increases in the per capita food supply and consumption of high calorific foods (e.g. fat, sugar, fast and/or processed foods) in South Africa[42]. This rapidly changing dietary pattern has, in part, been attributed to urbanisation, growing and expanding supermarkets /formal food retailers, and the availability of fast/processed foods [43]. An interesting finding in these data was the significant positive association between child obesity status and residing in a lower income household. This association has been demonstrated previously [44-46] and this evidence base is growing. This conforms with the idea that lower and higher income households/families often have a higher obesity risk than middle income households i.e. so called U-shaped association. Lower income or economically deprived families often replace health fresh food options with cheaper and more calorific processed foods [45]**.** Multiple studies have demonstrated that the majority of low-income South Africans have a low dietary diversity, and, therefore, consume a limited food range consisting predominantly of a starchy staple such as bread and maize, with low intakes of vegetables and fruit [42]. Future work will characterise food purchasing patterns (and changes over time) among households in South Africa which will be compared with paired longitudinal anthropometric measurements to identify specific dietary patterns associated with child nutritional status.

Lastly and contextually, body mass is culturally influenced in South Africa, and the high level of obesity in KwaZulu-Natal and Eastern Cape may at least in part be a result of cultural beliefs that associate overweight with wealth and good health [47]. Geographic patterns of higher obesity in South Africa appeared to overlap areas of high poverty particular on the eastern side of the country[3] and thus not solely concentrated among higher socio-economic households.

Strengths: To our knowledge this is the first spatial-temporal analysis of malnutrition trends among children under five years of age in South Africa. We used standardised anthropometric measurements of children and their mothers from a nationally representative repeated panel data over a 10-year period. The panel nature of the design allows assessment of change in malnutrition burden within the same individuals/households observed at multiple time points. A further strength was the implementation of a fully Bayesian space-time shared component model to produce more stable joint estimates of malnutrition by province, district and year.

---

[3] https://southafrica-info.com/people/mapping-poverty-in-south-africa/

For peer review only

Weaknesses: The study has several limitations. Firstly, missing or invalid weight/height measurements (especially in wave 2, and among infants – Supplementary Material 2) may have introduced selection bias (if not missing at random), and may thus have affected both the internal validity and the representativeness the findings in the broader South African context. Secondly as the primary panel study was not designed/powered for provincial [48]and lower geographic level analysis, we cannot discount the resultant impact on precision/random variability when analysing at provincial/district level (administrative tier just below province) and further stratification by socio-demographic correlates. Thirdly, we cannot discount the effect of inter-observer variability across different study districts, despite extensive interviewer training and standardization of study protocols. All anthropometric measurements (e.g. weight, height) were taken in duplicate in NIDS [26] which would have ensured better reliability.

Cost of malnutrition, policy and research needs: Estimating the cost of child malnutrition in South Africa is extremely complicated and no locally-determined cost data exist. Data from the United States, suggest that the incremental lifetime direct medical cost for a 10-year-old obese child relative to a 10-year-old normal weight child ranges from USD 12 660 to USD 19 630 [49]. Estimates of the cost of treating wasted children are approximately USD 200 per child [50] while stunting has been consistently linked to worse economic outcomes in adulthood [51] and estimates suggest that, on average, the future per capita income penalty for a stunted individual could be as large as 9-10% in SSA [52]. Urgent investments are needed to accelerate the reduction of all forms of malnutrition, as well as to curb the obesity epidemic among young children in South Africa. There is also considerable evidence indicates that childhood wasting and stunting can be reduced by 60% and 20% respectively using ten nutrition-specific interventions [53], with an estimated return on investment (ROI) of 18:1, i.e. for USD 1 spent on implementing effective programmes there would be USD 18 return in future economic benefits [54]. Very few obesity prevention interventions targeting children have been effective and a comprehensive multifaceted strategy tackling diet, physical inactivity, coupled with psychosocial support and local food environment change may prove more effective. Nutrition policies tackling child obesity must promote household nutrition security and healthy growth, decrease overconsumption of nutrient-poor foods, better shield children from increasingly pervasive marketing of energy-dense, nutrient-poor foods and sugar sweetened beverages as well as reduction of growing physical inactivity [55].

Our findings suggest the need to implement evidence-based child health strategies and policy (e.g. further social grant support to vulnerable and impoverished households) that is tailored to specific geographies and socially

For peer review only

disadvantaged sub-populations. Integrated nutrition programs in low and middle income countries (LMIC) have had a substantial impact on child nutrition and health via a combination of multisector targeted interventions [56]. Furthermore implementation and/or strengthening of school-based food program can provide a launching pad for preventive programs including education and awareness, provision of healthier/more nutrition food options and micronutrient supplementation, deworming, increased immunization coverage and improved growth monitoring as well as counselling [56]. This may be especially true of obese children where the highest prevalence was observed in higher income households with higher food purchasing power and where local food environments are likely is likely to be an important contextual determinant. A higher prevalence of child thinness/wasting among younger mothers (<25) in poorer, food insecure household, highlights the importance of policies that enable younger mothers to adequately care for their children in all settings.

Conclusions

The heterogeneity of malnutrition is a feature of spatial inequality and rapid urbanization that has manifested in widening levels of inequality in South Africa's districts and a need to reassess where nutrition programmes need to be further decentralised to the highest risk municipalities and local communities to maximise effectiveness. This work provides the first district level ranking of childhood overweight, thinness/wasting and stunting and allows a differentiated pro-active tailored intervention to be developed for each municipal district. The dual epidemic of undernutrition and overweight/obesity requires differential geographical policy inputs in metropolitan areas and districts across the rural-urban divide. The current and future health cost of malnutrition among South African children cannot be overstated. There is an urgent need to address nutrition problems among preschool aged children in South Africa and other low and middle income countries. Effective public health planning and geographically/contextually tailored interventions are required at sub-national level to address this challenge. The analytical framework employed in this study we believe will have definite utility in other settings.

References

1. Development Initiatives. 2018 Global Nutrition Report: Shining a light to spur action on nutrition. Bristol, UK: Development Initiatives, 2018.
2. Finucane MM, Stevens G, Cowan M, et al. Global Burden of Metabolic Risk Factors of Chronic Diseases Collaborating Group (Body Mass Index) National, regional, and global trends in body-mass index since 1980: systematic analysis of health examination surveys and epidemiological studies with 960 country-years and 9.1 million participants. *Lancet* 2011;377(9765):557-67.
3. Collaborators TGO. Health Effects of Overweight and Obesity in 195 Countries over 25 Years. *New England Journal of Medicine*;0(0):null. doi: 10.1056/NEJMoa1614362
4. Collaboration NRF. Trends in adult body-mass index in 200 countries from 1975 to 2014: a pooled analysis of 1698 population-based measurement studies with 19· 2 million participants. *The Lancet* 2016;387(10026):1377-96.

For peer review only

5. Bleich SN, Vercammen KA, Zatz LY, et al. Interventions to prevent global childhood overweight and obesity: a systematic review. *The Lancet Diabetes & Endocrinology* 2017
6. Malik VS, Willett WC, Hu FB. Global obesity: trends, risk factors and policy implications. *Nature Reviews Endocrinology* 2013;9(1):13-27.
7. Bain LE, Awah PK, Geraldine N, et al. Malnutrition in Sub–Saharan Africa: burden, causes and prospects. *Pan African Medical Journal* 2013;15(1)
8. Rossouw HA, Grant CC, Viljoen M. Overweight and obesity in children and adolescents: The South African problem. *South African journal of science* 2012;108(5-6):31-37.
9. Keino S, Plasqui G, Ettyang G, et al. Determinants of stunting and overweight among young children and adolescents in sub-Saharan Africa. *Food and nutrition bulletin* 2014;35(2):167-78.
10. Osgood-Zimmerman A, Millear AI, Stubbs RW, et al. Mapping child growth failure in Africa between 2000 and 2015. *Nature* 2018;555(7694):41.
11. Cois A, Day C. Obesity trends and risk factors in the South African adult population. *BMC obesity* 2015;2(1):42.
12. Sartorius B, Veerman LJ, Manyema M, et al. Determinants of obesity and associated population attributability, South Africa: Empirical evidence from a national panel survey, 2008-2012. *PloS one* 2015;10(6):e0130218.
13. COOVADIA H, BLAND R, TOLLMAN SM, et al. Implications of mortality transition for primary health care in rural South Africa: a population-based surveillance study. Commentary. *Lancet* 2008;372(9642)
14. Sartorius BK, Chersich MF, Mwaura M, et al. Maternal anaemia and duration of zidovudine in antiretroviral regimens for preventing mother-to-child transmission: a randomized trial in three African countries. *BMC infectious diseases* 2013;13(1):1.
15. Health WHONf. WHO child growth standards: growth velocity based on weight, length and head circumference: methods and development: World Health Organization 2009.
16. Organization WH, Health WHONf. WHO child growth standards: head circumference-for-age, arm circumference-for-age, triceps skinfold-for-age and subscapular skinfold-for-age: methods and development: World Health Organization 2007.
17. Gibson RS. Principles of nutritional assessment: Oxford university press, USA 2005.
18. Richardson S, Abellan JJ, Best N. Bayesian spatio-temporal analysis of joint patterns of male and female lung cancer risks in Yorkshire (UK). *Statistical methods in medical research* 2006;15(4):385-407.
19. Zere E, McIntyre D. Inequities in under-five child malnutrition in South Africa. *International journal for equity in health* 2003;2(1):7.
20. Jinabhai C, Taylor M, Sullivan K. Implications of the prevalence of stunting, overweight and obesity amongst South African primary school children: a possible nutritional transition? *European journal of clinical nutrition* 2003;57(2):358.
21. Armstrong ME, Lambert MI, Lambert EV. Secular trends in the prevalence of stunting, overweight and obesity among South African children (1994–2004). *European journal of clinical nutrition* 2011;65(7):835.
22. Kandala N-B, Madungu TP, Emina JB, et al. Malnutrition among children under the age of five in the Democratic Republic of Congo (DRC): does geographic location matter? *BMC public health* 2011;11(1):261.
23. Bredenkamp C, Buisman LR, Van de Poel E. Persistent inequalities in child undernutrition: evidence from 80 countries, from 1990 to today. *International Journal of Epidemiology* 2014;43(4):1328-35.
24. Di Cesare M, Bhatti Z, Soofi SB, et al. Geographical and socioeconomic inequalities in women and children's nutritional status in Pakistan in 2011: an analysis of data from a nationally representative survey. *The Lancet Global Health* 2015;3(4):e229-e39.
25. Ataguba JE, Akazili J, McIntyre D. Socioeconomic-related health inequality in South Africa: evidence from General Household Surveys. *International journal for equity in health* 2011;10(1):48.
26. Van de Poel E, Hosseinpoor AR, Speybroeck N, et al. Socioeconomic inequality in malnutrition in developing countries. *Bulletin of the World Health Organization* 2008;86:282-91.
27. Delpeuch F, Traissac P, Martin-Prével Y, et al. Economic crisis and malnutrition: socioeconomic determinants of anthropometric status of preschool children and their mothers in an African urban area. *Public health nutrition* 2000;3(1):39-47.
28. Oyvat C. Agrarian structures, urbanization, and inequality. *World Development* 2016;83:207-30.
29. Hoddinott J, Haddad L. Does female income share influence household expenditures? Evidence from Côte d'Ivoire. *oxford Bulletin of Economics and Statistics* 1995;57(1):77-96.
30. Aryastami NK, Shankar A, Kusumawardani N, et al. Low birth weight was the most dominant predictor associated with stunting among children aged 12–23 months in Indonesia. *BMC Nutrition* 2017;3(1):16.
31. de Bernabé JV, Soriano T, Albaladejo R, et al. Risk factors for low birth weight: a review. *European Journal of Obstetrics & Gynecology and Reproductive Biology* 2004;116(1):3-15.

For peer review only

32. Frank TD, Carter A, Jahagirdar D, et al. Global, regional, and national incidence, prevalence, and mortality of HIV, 1980–2017, and forecasts to 2030, for 195 countries and territories: a systematic analysis for the Global Burden of Diseases, Injuries, and Risk Factors Study 2017. *The Lancet HIV* 2019
33. Xiao P-L, Zhou Y-B, Chen Y, et al. Association between maternal HIV infection and low birth weight and prematurity: a meta-analysis of cohort studies. *BMC pregnancy and childbirth* 2015;15(1):246.
34. Althabe F, Moore JL, Gibbons L, et al. Adverse maternal and perinatal outcomes in adolescent pregnancies: The Global Network's Maternal Newborn Health Registry study. *Reproductive health* 2015;12(2):S8.
35. Patel D, Bland R, Coovadia H, et al. Breastfeeding, HIV status and weights in South African children: a comparison of HIV-exposed and unexposed children. *Aids* 2010;24(3):437-45.
36. Wamani H, Åstrøm AN, Peterson S, et al. Boys are more stunted than girls in sub-Saharan Africa: a meta-analysis of 16 demographic and health surveys. *BMC pediatrics* 2007;7(1):17.
37. Redding S, Conrey E, Porter K, et al. Pathways community care coordination in low birth weight prevention. *Maternal and child health journal* 2015;19(3):643-50.
38. Statistics South Africa. Recorded live births 2012. www.statssa.gov.za/publications/P0305/P03052011.pdf. 2012
39. Organization WH. WHA Global nutrition targets 2025: low birth weight policy brief. Geneva: WHO, 2014.[Accessed on: June 1st, 2017].
40. Monasta L, Batty G, Cattaneo A, et al. Early-life determinants of overweight and obesity: a review of systematic reviews. *Obesity reviews* 2010;11(10):695-708.
41. Kruger R, Kruger H, Macintyre U. The determinants of overweight and obesity among 10-to 15-year-old schoolchildren in the North West Province, South Africa–the THUSA BANA (Transition and Health during Urbanisation of South Africans; BANA, children) study. *Public health nutrition* 2006;9(3):351-58.
42. Pereira LM. The future of South Africa's food system: What is research telling us. *SA Food Lab South Africa* 2014
43. McLachlan M, Landman A. Nutrition-sensitive agriculture–a South African perspective. *Food security* 2013;5(6):857-71.
44. Brophy S, Cooksey R, Gravenor MB, et al. Risk factors for childhood obesity at age 5: analysis of the millennium cohort study. *BMC public health* 2009;9(1):467.
45. Brunt H, Lester N, Davies G, et al. Childhood overweight and obesity: is the gap closing the wrong way? *Journal of public health* 2008;30(2):145-52.
46. Kim TJ, von dem Knesebeck O. Income and obesity: what is the direction of the relationship? A systematic review and meta-analysis. *BMJ open* 2018;8(1):e019862.
47. Rossouw HA, Grant CC, Viljoen M. Overweight and obesity in children and adolescents: the South African problem: review article. *South African Journal of Science* 2012;108(5-6):1-7.
48. Leibbrandt M, Woolard I, de Villiers L. Methodology: Report on NIDS wave 1. *Technical paper* 2009;1
49. Finkelstein EA, Graham WCK, Malhotra R. Lifetime direct medical costs of childhood obesity. *Pediatrics* 2014:peds. 2014-0063.
50. Horton S, Shekar M, Ajay M. Scaling up nutrition: What will it cost?: The World Bank 2009.
51. McGovern ME, Krishna A, Aguayo VM, et al. A review of the evidence linking child stunting to economic outcomes. *International journal of epidemiology* 2017;46(4):1171-91.
52. Sartorius K, Sartorius B, Winkler C, et al. 1. Abstract 2. Introduction 3. miRNA biogenesis, mRNA silencing and export into the extracellular space 3.1. Intracellular mRNA silencing 3.2. Extracellular mRNA silencing.
53. Vaivada T, Gaffey MF, Das JK, et al. Evidence-based interventions for improvement of maternal and child nutrition in low-income settings: what's new? *Current Opinion in Clinical Nutrition & Metabolic Care* 2017;20(3):204-10.
54. Hoddinott J, Alderman H, Behrman JR, et al. The economic rationale for investing in stunting reduction. *Maternal & Child Nutrition* 2013;9:69-82.
55. Lobstein T, Jackson-Leach R, Moodie ML, et al. Child and adolescent obesity: part of a bigger picture. *The lancet* 2015;385(9986):2510-20.
56. UNICEF. Progress for children: a report card on nutrition: Unicef 2006.

For peer review only

Authors' contributions

All authors contributed to the conception and design of the study. BS performed the analysis and initial interpretation of the findings. BS drafted the manuscript. All authors reviewed and provided input to revise the manuscript. All authors gave final approval for submission.

Funding

This study forms part of the Sustainable and Healthy Food Systems (SHEFS) project supported by the Wellcome Trust's Our Planet, Our Health programme (grant number 205200/Z/16/Z). The funders of the study had no role in study design, data collection, data analysis, data interpretation or writing of the report.

Competing interests

None declared.

Patient consent for publication

Not required.

Ethics approval

This study utilised open access data and hence ethical approval was not necessary.

Data availability statement

Data are publically available at https://www.datafirst.uct.ac.za/dataportal/index.php/catalog/NIDS/about

For peer review only

Tables

Table 1: Burden of stunting, thinness/wasting and obesity among children by age and survey round

Survey wave	Age (in years)	N (valid HAZ)	n (stunted)	Prop: Stunted i	Estimated Population stunted	N (valid BMIZ)	n (thin /wasted)	Prop: Thinness ii	Estimated Population thinness	n (obese)	Prop: obese iii	Estimated Population obese
2008	0	220	31	0.14 (0.09, 0.22)	153648 (81545, 273371)	180	21	0.12 (0.07, 0.2)	133882 (66374, 251867)	32	0.1 (0.06, 0.15)	107783 (59737, 185749)
	1	419	29	0.08 (0.05, 0.13)	91903 (48436, 164369)	386	24	0.06 (0.03, 0.11)	66566 (29263, 143661)	76	0.22 (0.16, 0.3)	253021 (159436, 383096)
	2	453	62	0.15 (0.1, 0.21)	159241 (96989, 250626)	419	10	0.03 (0.01, 0.07)	34613 (12484, 87598)	70	0.14 (0.1, 0.19)	148357 (93148, 227510)
	3	489	55	0.11 (0.08, 0.15)	111595 (69906, 172639)	470	19	0.04 (0.02, 0.07)	39715 (20205, 75821)	67	0.17 (0.12, 0.24)	176235 (104092, 284620)
	4	498	48	0.09 (0.06, 0.13)	93391 (54519, 154136)	461	25	0.05 (0.03, 0.08)	52031 (27083, 96623)	34	0.08 (0.05, 0.12)	80282 (45874, 135732)
	**0-5**	**2079**	**225**	**0.11 (0.09, 0.13) iv**	**591550 (451494, 766049)**	**1916**	**99**	**0.05 (0.04, 0.07) iv**	**277743 (196715, 385904)**	**279**	**0.14 (0.12, 0.17) iv**	**778865 (599156, 996439)**
2010/11	0	75	24	0.33 (0.16, 0.57)	289420 (114550, 577181)	69	7	0.1 (0.04, 0.23)	88499 (30258, 228461)	22	0.39 (0.21, 0.61)	340820 (153454, 615984)
	1	236	20	0.06 (0.03, 0.11)	63995 (25204, 132218)	215	11	0.07 (0.03, 0.14)	69776 (25204, 173842)	52	0.29 (0.19, 0.41)	299127 (159624, 499489)
	2	340	61	0.22 (0.16, 0.29)	267019 (166414, 407708)	314	17	0.06 (0.03, 0.11)	76344 (35363, 155183)	72	0.22 (0.16, 0.29)	270818 (167454, 414761)
	3	427	52	0.11 (0.07, 0.16)	130531 (73921, 220389)	402	20	0.03 (0.02, 0.06)	39208 (16427, 85938)	78	0.16 (0.11, 0.23)	195314 (114988, 313258)
	4	422	62	0.17 (0.12, 0.24)	205730 (122130, 329629)	394	19	0.03 (0.02, 0.06)	39494 (17639, 84450)	65	0.17 (0.12, 0.24)	208842 (126152, 329629)
	**0-5**	**1500**	**219**	**0.16 (0.13, 0.19)**	**862302 (633920, 1148376)**	**1394**	**74**	**0.05 (0.03, 0.07)**	**265877 (167080, 405309)**	**289**	**0.21 (0.17, 0.26)**	**1159133 (835398, 1565968)**
2012	0	271	59	0.2 (0.14, 0.28)	181464 (108101, 288795)	250	38	0.2 (0.12, 0.3)	179118 (95658, 311389)	55	0.19 (0.12, 0.28)	169192 (94880, 284482)
	1	544	78	0.13 (0.09, 0.17)	132310 (80796, 207206)	538	27	0.08 (0.05, 0.13)	80862 (40842, 150046)	138	0.23 (0.18, 0.28)	234062 (157153, 334626)
	2	629	72	0.1 (0.07, 0.14)	116230 (68690, 187924)	629	49	0.05 (0.03, 0.07)	55866 (30861, 97391)	147	0.23 (0.18, 0.29)	269508 (176205, 392309)
	3	710	82	0.11 (0.08, 0.16)	142259 (82987, 232297)	692	29	0.03 (0.02, 0.06)	43898 (20928, 87296)	102	0.15 (0.11, 0.2)	191943 (117798, 297399)
	4	771	112	0.16 (0.12, 0.2)	221293 (142258, 330201)	762	30	0.03 (0.02, 0.05)	43556 (20731, 87406)	118	0.18 (0.14, 0.22)	250658 (167278, 362573)
	**0-5**	**2925**	**403**	**0.13 (0.11, 0.16)**	**762303 (567517, 1001855)**	**2871**	**173**	**0.06 (0.05, 0.07)**	**328768 (230074, 458914)**	**560**	**0.19 (0.17, 0.22)**	**1112487 (853832, 1415525)**
2014/15	0	434	74	0.12 (0.08, 0.18)	144201 (81319, 240730)	421	37	0.1 (0.06, 0.18)	123211 (59233, 240730)	78	0.17 (0.12, 0.23)	197209 (117461, 313223)
	1	801	53	0.06 (0.04, 0.08)	67916 (39433, 112566)	801	24	0.03 (0.01, 0.08)	39657 (9858, 101845)	169	0.23 (0.18, 0.28)	266780 (179421, 379240)
	2	785	65	0.08 (0.05, 0.12)	85985 (48668, 146305)	781	16	0.02 (0.01, 0.03)	16222 (6309, 39015)	128	0.16 (0.12, 0.22)	170803 (106348, 263349)
	3	853	82	0.08 (0.06, 0.11)	89857 (54478, 143034)	845	24	0.04 (0.02, 0.07)	40865 (18323, 86890)	79	0.12 (0.08, 0.15)	133857 (83637, 205862)
	4	899	67	0.06 (0.04, 0.09)	77887 (45801, 127320)	897	19	0.02 (0.01, 0.05)	30376 (12301, 71898)	56	0.06 (0.04, 0.11)	82300 (38662, 166265)
	**0-5**	**3772**	**341**	**0.08 (0.06, 0.09)**	**441281 (327611, 581707)**	**3745**	**120**	**0.04 (0.03, 0.05)**	**213012 (130004, 333338)**	**510**	**0.14 (0.12, 0.17)**	**834444 (618820, 1098053)**
2017	0	372	50	0.13 (0.08, 0.19)	125347 (68160, 218303)	357	32	0.12 (0.07, 0.2)	121396 (62270, 221478)	70	0.18 (0.12, 0.25)	174538 (104344, 278066)
	1	760	55	0.08 (0.05, 0.11)	95527 (56435, 153804)	742	23	0.03 (0.02, 0.07)	42416 (17767, 94222)	146	0.23 (0.19, 0.29)	285123 (194388, 403216)
	2	833	63	0.07 (0.05, 0.11)	94807 (54147, 158550)	830	20	0.03 (0.02, 0.07)	43976 (18786, 99279)	130	0.15 (0.12, 0.19)	191812 (127079, 280056)
	3	875	77	0.08 (0.05, 0.12)	99890 (54439, 175689)	872	14	0.02 (0.01, 0.06)	30726 (10888, 79204)	77	0.07 (0.05, 0.1)	88889 (54439, 138247)
	4	900	59	0.05 (0.04, 0.07)	57363 (34849, 91231)	899	23	0.03 (0.01, 0.05)	29923 (13628, 62962)	47	0.06 (0.04, 0.08)	63912 (36990, 105365)
	**0-5**	**3740**	**304**	**0.08 (0.06, 0.09) iv**	**445295 (326192, 593240)**	**3700**	**112**	**0.04 (0.03, 0.05) iv**	**223236 (136790, 345514)**	**470**	**0.13 (0.11, 0.15) iv**	**758650 (583989, 964831)**
**At last observation**	**0-5**	**10711**	**1049**	**0.09 (0.08, 0.10)**	**1 397 020 (1 177 247, 1 616 793)**	**10467**	**391**	**0.04 (0.03, 0.05)**	**560 806 (448 656, 672 957)**	**1,438**	**0.14 (0.13, 0.16)**	**2 048 650 (1 722 242, 2 375 058)**

i: HAZ ≤ -2 SD; ii BMI for age z-score ≤ -2SD; iii BMI for age z-score ≥ +2SD

iv: Significance tests (survey weighted logistic regression) among children 0-5: stunting (2017 vs 2008) p=0.007; thinness/wasting (2017 vs 2008) p=0.131; obesity (2017 vs 2008) p=0.312

For peer review only

Table 2: Demographic, socio-economic and maternal factors associated with nutritional status among children under 5 years, 2008-2017

**Variable**	**Category**	**Stunted**		**p-value**	**Thin/wasted**		**p-value**	**Obese**		**p-value**
		**Yes (% col)**	**No (% col)**		**Yes (% col)**	**No (% col)**		**Yes (% col)**	**No (% col)**	
	African	**0.939**	0.871		0.885	0.876		**0.929**	0.866	
	Coloured	0.053	**0.074**		0.076	0.072		0.051	**0.076**	
	Asian/Indian	0.003	**0.012**		0.015	0.011		0.003	**0.013**	
Ethnicity	White	0.006	**0.039**	**<0.001**	0.025	0.037	0.858	0.014	**0.041**	**<0.001**
	Male	**0.562**	0.496		0.514	0.501		0.523	0.498	
Gender	Female	0.438	0.504	**0.002**	0.486	0.499	0.686	0.477	0.502	0.178
	LBW (<2.5 kgs)	**0.153**	0.093		**0.130**	0.098		0.072	**0.104**	
	NBW (≥2.5 kgs)	0.847	0.907	**<0.001**	0.870	0.902	0.163	0.928	0.896	**0.006**
	HBW (≥4 kgs)							**0.056**	0.040	
Birthweight	Non-HBW (<4kgs)	N/A						0.944	0.960	**0.037**
	Lowest	**0.294**	0.199		**0.234**	0.203		0.226	0.200	
	Low	0.205	0.187		**0.214**	0.188		0.203	0.186	
	Middle	0.183	0.200		0.169	0.201		0.180	0.204	
	High	0.197	0.186		0.184	0.191		0.182	0.192	
Income quantile	Highest	0.122	**0.229**	**<0.001**	0.200	0.218	0.481	0.209	0.218	0.422
Low monthly household income	<R2500	**0.566**	0.417		**0.488**	0.423		**0.481**	0.416	
	≥R2500	0.434	**0.583**	**<0.001**	0.512	0.577	**0.053**	0.519	0.584	**0.001**
	Never	0.689	0.697		0.512	**0.704**		0.707	0.693	
	Seldom	0.127	0.096		0.111	0.097		0.076	0.102	
	Sometimes	0.126	0.155		**0.317**	0.148		0.154	0.155	
	Often	0.054	0.043		0.052	0.042		0.052	0.041	
Child hungry in last year (food security) i	Always	0.004	0.009	0.505	0.007	0.009	**<0.001**	0.011	0.009	0.645
	Eastern Cape	**0.165**	0.132		0.075	0.137		**0.190**	0.124	
	Free State	0.066	0.050		0.032	0.052		0.045	0.052	
	Gauteng	0.188	0.236		**0.298**	0.231		0.173	0.246	
	KwaZulu-Natal	0.218	0.227		0.161	0.228		**0.293**	0.212	
	Limpopo	**0.143**	0.109		0.129	0.113		0.074	0.121	
	Mpumalanga	0.085	0.083		0.096	0.082		0.074	0.085	
	North West	0.055	0.050		0.060	0.050		0.038	0.053	
	Northern Cape	0.022	0.023		0.033	0.022		0.011	0.025	
Province	Western Cape	0.060	0.091	**0.012**	0.116	0.086	**0.002**	0.103	0.084	**<0.001**
	Rural/Tribal authority	**0.519**	0.451		0.429	0.460		0.466	0.457	
	Urban Informal	0.122	0.101		0.100	0.102		**0.133**	0.097	
Environment	Urban Formal	0.359	**0.448**	**<0.001**	0.470	0.437	0.647	0.402	0.446	0.111
	Underweight	**0.041**	0.022		**0.051**	0.023		0.019	0.025	
	Normal	**0.397**	0.344		**0.418**	0.348		0.327	0.356	
	Overweight	0.268	0.273		0.249	0.272		0.260	0.273	
Mother BMI	Obese	0.294	**0.361**	**0.003**	0.282	**0.357**	**0.005**	**0.395**	0.346	0.135
Mother age	<20	0.073	0.048	0.118	**0.112**	0.047	**0.012**	**0.057**	0.049	0.311

For peer review only

	20-24	0.271	0.292		0.292	0.291		**0.322**	0.285	
	25-34	0.470	0.461		0.396	0.465		0.441	**0.465**	
	35-44	0.171	0.183		0.185	0.182		0.168	**0.185**	
	45+	0.015	0.016		0.015	0.016		0.012	**0.017**	
Mother education	None	0.004	0.002	**<0.001**	0.004	0.002	0.540	0.001	0.002	0.111
	Primary	**0.762**	0.621		0.645	0.630		0.652	0.626	
	Secondary	0.207	**0.354**		0.326	0.344		0.314	0.349	
	Tertiary	0.028	0.024		0.025	0.024		0.033	0.023	
Father education	None	0.003	0.003	**0.020**	0.005	0.003	0.960	0.002	0.003	**0.033**
	Primary	**0.646**	0.560		0.565	0.556		0.584	0.551	
	Secondary	0.275	0.389		0.382	0.387		0.318	0.398	
	Tertiary	0.077	0.048		0.048	0.055		**0.097**	0.047	

i: only included in wave 1 questionnaire

For peer review only

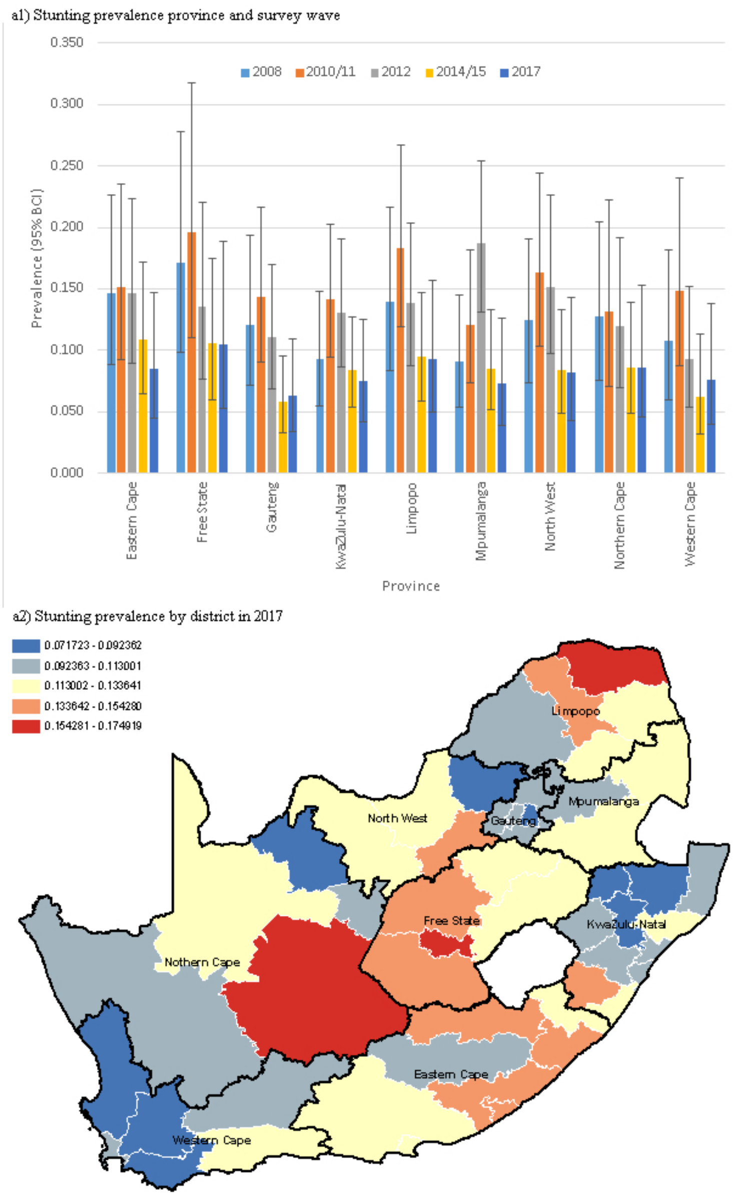
a1) Stunting prevalence province and survey wave
2008
2010/11
2012
2014/15
2017
Prevalence (95% BCI)
0.350
0.300
0.250
0.200
0.150
0.100
0.050
0.000
Eastern Cape
Free State
Gauteng
KwaZulu-Natal
Limpopo
Mpumalanga
North West
Northern Cape
Western Cape
Province
a2) Stunting prevalence by district in 2017
0.071723 - 0.092362
0.092363 - 0.113001
0.113002 - 0.133641
0.133642 - 0.154280
0.154281 - 0.174919
Limpopo
Mpumalanga
North West
Gauteng
Free State
KwaZulu-Natal
Nothern Cape
Eastern Cape
Western Cape

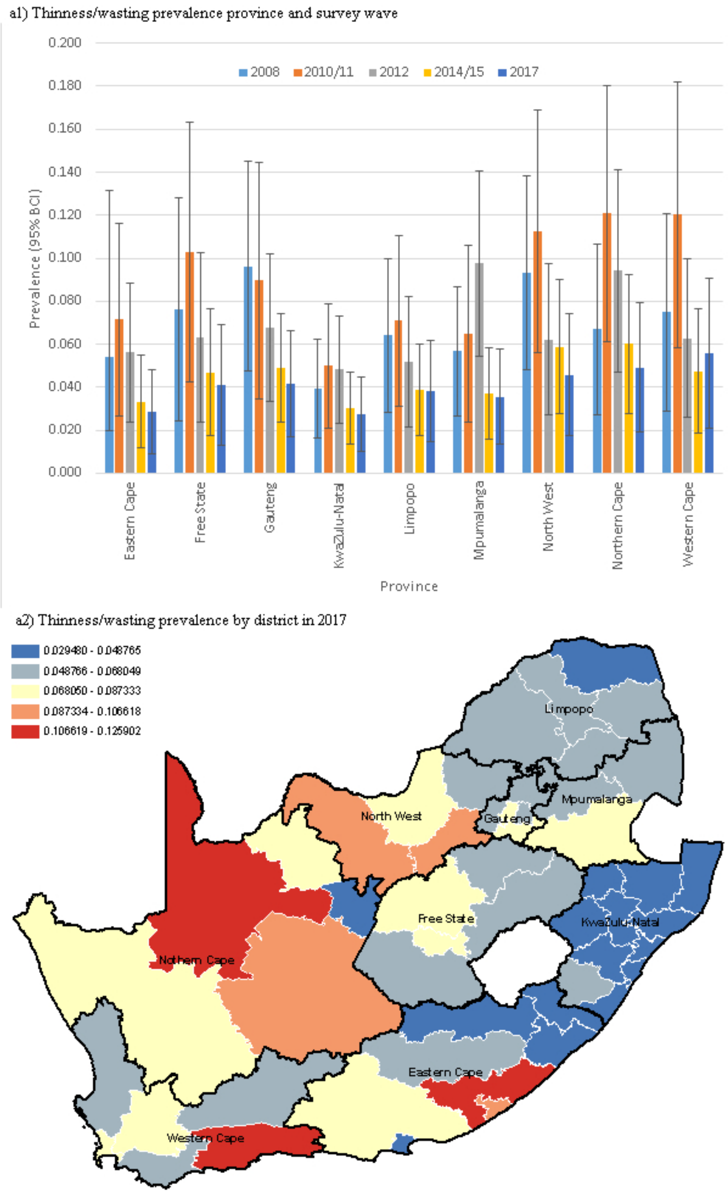
a1) Thinness/wasting prevalence province and survey wave
2008
2010/11
2012
2014/15
2017
Prevalence (95% BCI)
0.200
0.180
0.160
0.140
0.120
0.100
0.080
0.060
0.040
0.020
0.000
Eastern Cape
Free State
Gauteng
Kwa2ulu-Natal
Limpopo
Mpumalanga
North West
Northern Cape
Western Cape
Province
a2) Thinness/wasting prevalence by district in 2017
0.029480 - 0.048765
0.048766 - 0.068049
0.068050 - 0.087333
0.087334 - 0.106618
0.106619 - 0.125902
Limpopo
Mpumalanga
North West
Gauteng
Free State
Kwa2ulu-Natal
Nothern Cape
Eastern Cape
Western Cape

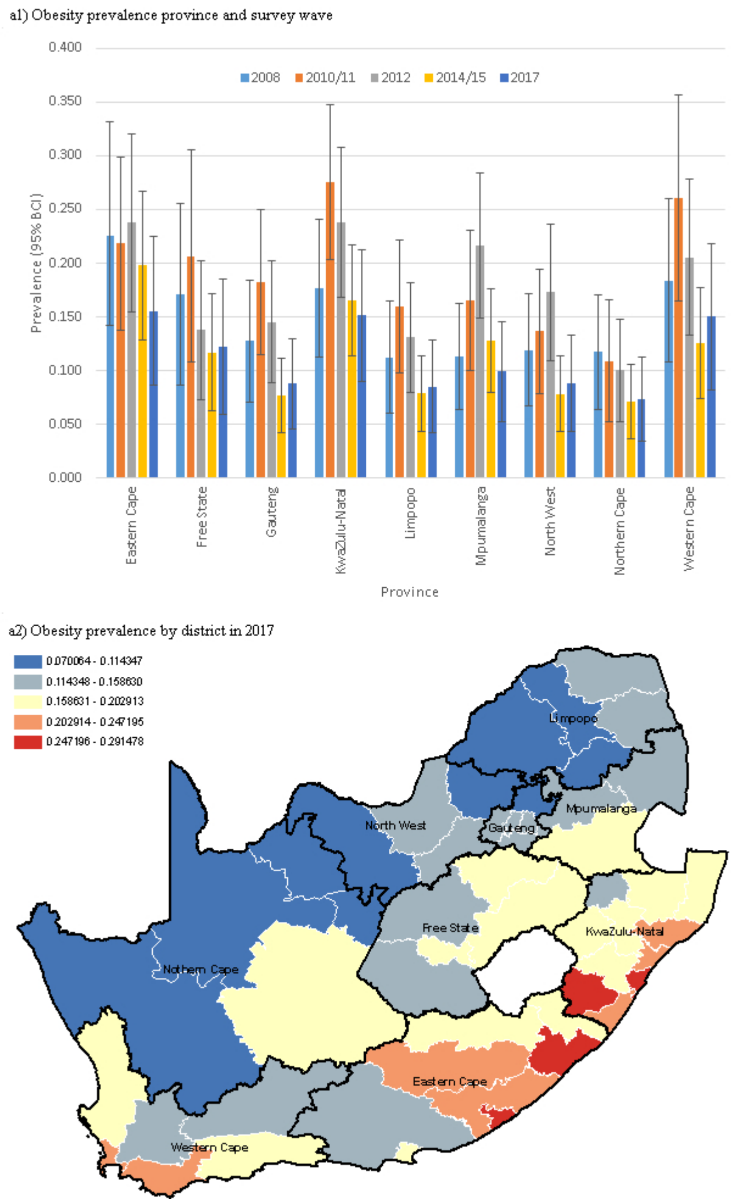
a1) Obesity prevalence province and survey wave
2008
2010/11
2012
2014/15
2017
Prevalence (95% BCI)
0.400
0.350
0.300
0.250
0.200
0.150
0.100
0.050
0.000
Eastern Cape
Free State
Gauteng
KwaZulu-Natal
Limpopo
Mpumalanga
North West
Northern Cape
Western Cape
Province
a2) Obesity prevalence by district in 2017
0.070064 - 0.114347
0.114348 - 0.158630
0.158631 - 0.202913
0.202914 - 0.247195
0.247196 - 0.291478
Limpopo
Mpumalanga
Gauteng
North West
Free State
KwaZulu-Natal
Nothern Cape
Eastern Cape
Western Cape

Supplementary Material

Supplementary 1: Win BUGS code for Bayesian joint (shared component) space-time binomial model

```
model

{
( i in 1 : N ) {
            for( j in 1 : T ) {
            #Likelihood

            stunted[i,j] ~ dbin(p1[i,j],tot[i,j])
            logit(p1[i,j])<-alpha1+mu[i,j,1]

            thin[i,j] ~ dbin(p2[i,j],tot[i,j])
            logit(p2[i,j])<-alpha2+mu[i,j,2]

            obese[i,j] ~ dbin(p3[i,j],tot[i,j])
            logit(p3[i,j])<-alpha3+mu[i,j,3]

            mu[i,j,1:3]~dmnorm(eta[i,j,],Sigma.inv[,])

            #Joint modelling
            eta[i,j,1]<-lambda[i]*delta+xi[j]*kappa+nu[i,j]
            eta[i,j,2]<-lambda[i]/delta+xi[j]/kappa+nu[i,j]+beta1[i]+gamma1[j]
            eta[i,j,3]<-lambda[i]/delta+xi[j]/kappa+nu[i,j]+beta2[i]+gamma2[j]

            }
}

# - Space
lambda[1:52]~car.normal(adj[],weights[],num[],tau.lambda)
beta1[1:52]~car.normal(adj[],weights[],num[],tau.beta1)
beta2[1:52]~car.normal(adj[],weights[],num[],tau.beta2)

for(k in 1:240) {weights[k]<-1}

# - Time:

xi[1:T]~car.normal(adj.t[],weights.t[],num.t[],tau.xi)
gamma1[1:T]~car.normal(adj.t[],weights.t[],num.t[],tau.gamma1)
gamma2[1:T]~car.normal(adj.t[],weights.t[],num.t[],tau.gamma2)

for(t in 1:1) {
           weights.t[t] <- 1;
           adj.t[t] <- t+1;
           num.t[t] <- 1
           }
for(t in 2:(T-1)) {
           weights.t[2+(t-2)*2] <- 1;
           adj.t[2+(t-2)*2] <- t-1
           weights.t[3+(t-2)*2] <- 1;
           adj.t[3+(t-2)*2] <- t+1;
           num.t[t] <- 2
           }
for(t in T:T) {
           weights.t[(T-2)*2 + 2] <- 1;
           adj.t[(T-2)*2 + 2] <- t-1;
           num.t[t] <- 1
           }
#Space-time Interaction Modelling (priors)
for(i in 1:N){
            for(j in 1:T){
            nu[i,j]~dnorm(0, tau.nu)
            RRnu[i,j]<-exp(nu[i,j])
            prob.nu[i,j]<-step(nu[i,j])
            }
}

#Hyperprior specification

tau.lambda~dgamma(0.5, 0.0005)
tau.xi~dgamma(0.5, 0.0005)
tau.beta1~dgamma(0.5, 0.0005)
tau.beta2~dgamma(0.5, 0.0005)
tau.gamma1~dgamma(0.5, 0.0005)
tau.gamma2~dgamma(0.5, 0.0005)
tau.nu~dgamma(0.5, 0.0005)

Sigma.inv[1:3,1:3]~dwish(B[,],3)
log(delta)<-logdelta
log(kappa)<-logkappa
logdelta~dnorm(0,0.2)
logkappa~dnorm(0,0.2)
B[1,1]<-0.01
B[2,2]<-0.01
B[3,3]<-0.01
B[1,2]<-0
B[1,3]<-0
B[2,1]<-0
B[2,3]<-0
B[3,1]<-0
B[3,2]<-0

alpha1~dflat()
alpha2~dflat()
alpha3~dflat()
```

For peer review only - http://bmjopen.bmj.com/site/about/guidelines.xhtml

Supplementary 2: Description of the study sample across survey rounds

Survey wave	Age (in years)	Sampled	Estimated population size using survey weights	95% CI		% sampled with height/weight measurement
**2008**	0	661	1092027	948199	1235854	35.9%
	1	661	1151665	1009086	1294244	67.9%
	2	670	1088458	960285	1216632	71.0%
	3	642	1034244	902011	1166477	81.0%
	4	620	1016227	882185	1150270	83.5%
	<5	3254	5382621	5005478	5759764	
**2010/11**	0	517	866786	720440	1013132	16.2%
	1	621	1032184	840129	1224239	42.5%
	2	751	1225419	1040085	1410753	49.3%
	3	840	1206389	1026681	1386097	53.3%
	4	820	1196800	1031500	1362101	53.3%
	<5	3549	5527578	4914106	6141050	
**2012**	0	652	902357	777704	1027010	45.1%
	1	691	1039354	887868	1190839	87.7%
	2	764	1183609	995508	1371711	87.6%
	3	826	1257820	1036042	1479598	89.6%
	4	909	1405034	1191438	1618631	87.3%
	<5	3842	5788174	5112765	6463583	
**2014/15**	0	886	1185863	1003941	1367786	50.3%
	1	875	1162949	985828	1340070	92.9%
	2	863	1060232	901257	1219207	92.7%
	3	914	1160946	985127	1336765	94.0%
	4	960	1298110	1098342	1497879	94.3%
	<5	4498	5868101	5200170	6536031	
**2017**	0	813	987763	841487	1134040	47.8%
	1	909	1215360	1045099	1385622	86.4%
	2	996	1293408	1105038	1481779	84.6%
	3	992	1264427	1088783	1440071	88.9%
	4	1000	1129184	973431	1284937	90.4%
	<5	4710	5890142	5261158	6519126	

For peer review only

BMJ Open

Spatial-temporal trends and risk factors for under-nutrition and obesity among children (<5 years) in South Africa, 2008-2017: findings from a nationally representative longitudinal panel survey

Journal:	*BMJ Open*
Manuscript ID	bmjopen-2019-034476.R1
Article Type:	Original research
Date Submitted by the Author:	23-Dec-2019
Complete List of Authors:	Sartorius, Benn; London School of Hygiene and Tropical Medicine Faculty of Infectious and Tropical Diseases, Disease Control<br>Sartorius, Kurt; University of KwaZulu-Natal, Public Health Medicine, School of Nursing and Public Health<br>Green, Rosemary; London School of Hygiene and Tropical Medicine, 2 Department of Population Health<br>Lutge, Elizabeth; South African Government Department, KwaZulu Natal Department of Health<br>Scheelbeek, Pauline; London School of Hygiene & Tropical Medicine, Epidemiology and Population Health<br>Tanser, Frank; Africa Centre for Health and Population Studies; University of KwaZulu-Natal<br>Dangour, Alan; London School of Hygiene & Tropical Medicine, Dept of Population Health<br>Slotow, Rob; University of KwaZulu-Natal
**Primary Subject Heading**:	Nutrition and metabolism
Secondary Subject Heading:	Epidemiology, Public health
Keywords:	PUBLIC HEALTH, Community child health < PAEDIATRICS, NUTRITION & DIETETICS

SCHOLARONE™
Manuscripts

BMJ

I, the Submitting Author has the right to grant and does grant on behalf of all authors of the Work (as defined in the below author licence), an exclusive licence and/or a non-exclusive licence for contributions from authors who are: i) UK Crown employees; ii) where BMJ has agreed a CC-BY licence shall apply, and/or iii) in accordance with the terms applicable for US Federal Government officers or employees acting as part of their official duties; on a worldwide, perpetual, irrevocable, royalty-free basis to BMJ Publishing Group Ltd ("BMJ") its licensees and where the relevant Journal is co-owned by BMJ to the co-owners of the Journal, to publish the Work in this journal and any other BMJ products and to exploit all rights, as set out in our licence.

The Submitting Author accepts and understands that any supply made under these terms is made by BMJ to the Submitting Author unless you are acting as an employee on behalf of your employer or a postgraduate student of an affiliated institution which is paying any applicable article publishing charge ("APC") for Open Access articles. Where the Submitting Author wishes to make the Work available on an Open Access basis (and intends to pay the relevant APC), the terms of reuse of such Open Access shall be governed by a Creative Commons licence – details of these licences and which Creative Commons licence will apply to this Work are set out in our licence referred to above.

Other than as permitted in any relevant BMJ Author's Self Archiving Policies, I confirm this Work has not been accepted for publication elsewhere, is not being considered for publication elsewhere and does not duplicate material already published. I confirm all authors consent to publication of this Work and authorise the granting of this licence.

For peer review only

Title page

Spatial-temporal trends and risk factors for under-nutrition and obesity among children (<5 years) in South Africa, 2008-2017: findings from a nationally representative longitudinal panel survey

Running title: under-nutrition and obesity among children in South Africa

Sartorius B[1-3]*, Sartorius K[2,4], Green R[5], Lutge E[2,6], Scheelbeek P[5], Tanser F[2,7-9], Dangour AD[5], Slotow R[10,11]

1. Department of Disease Control, London School of Hygiene & Tropical Medicine, London, UK
2. Department of Public Health Medicine, School of Nursing and Public Health, University of KwaZulu-Natal, Durban, South Africa
3. Department of Health Metrics Sciences, School of Medicine, University of Washington, Seattle, USA
4. Faculty of Commerce, Law and Management, University of the Witwatersrand, Johannesburg, South Africa
5. Department of Population Health, London School of Hygiene & Tropical Medicine, London, UK
6. Health Research and Knowledge Management, KwaZulu-Natal Department of Health, KwaZulu-Natal, South Africa
7. Africa Health Research Institute (AHRI), KwaZulu-Natal, South Africa
8. Centre for the AIDS Programme of Research in South Africa (CAPRISA), University of KwaZulu-Natal, Durban, South Africa
9. Institute of Epidemiology and Health Care, University College London, London, United Kingdom
10. School of Life Sciences, University of Kwazulu-Natal, Pietermaritzburg Campus, South Africa
11. Department of Genetics, Evolution and Environment, University College, London, UK

*Corresponding author: Dr Benn Sartorius; Address: Department of Disease Control, London School of Hygiene and Tropical Medicine, Keppel Street, Bloomsbury, London, WC1E7HT, United Kingdom; Tel.: +44 (0)207 927 2164; E-mail: benn.sartorius1@lshtm.ac.uk

Word count: 4095

For peer review only

Summary

Objectives: To assess space-time trends in the burden of malnutrition and associated risk factors among children under 5 years of age in South Africa

Design: national panel survey

Setting: national, community based

Participants: Community based sample of children and adults. Sample size: 3,254 children in wave 1 (2008) to 4,710 children in wave 5 (2017).

Primary outcomes: Stunting, wasting/thinness and obesity among children (<5). Classification were based on anthropometric (height and weight) z-scores using WHO growth standards.

Results: Between 2008 and 2017 there was a larger decline nationally in stunting prevalence among children under 5 years of age from 11.0% to 7.6% (p=0.007), compared to thinness/wasting (5.2% to 3.8%, p=0.131) and obesity (14.5% to 12.9%, p=0.312). Stunting prevalence appears relatively evenly spread across South Africa, while obesity is more pronounced in the east of the country and thinness/wasting more pronounced in the west. Approximately 80% (41/ 52) of districts had an estimated wasting prevalence below the 2025 target threshold of 5% in 2017.African ethnicity, male gender, low birth weight, lower socio-economic and maternal/paternal education status and residence in a rural area were significantly associated with stunting. Children living in a lower income and food insecure household with young malnourished mothers were significantly more likely to be thin/wasted while African children, with higher birth weights, living in lower income households in KwaZulu-Natal and Eastern Cape were significantly more likely to be obese.

Conclusions: While improvements in stunting have been observed, thinness/wasting and obesity prevalence remain largely unchanged. The geographic and socio-demographic heterogeneity in childhood malnutrition has implications for equitable attainment of global nutritional targets for 2025. Many districts appeared to have dual epidemics of under and over nutrition (high within district heterogeneity and inequality). Effective public health planning and tailored interventions are required at the sub-national level to address this challenge.

Keywords: nutritional status, nutritional transition, undernutrition, obesity, children, South Africa

Strengths and limitations of this study

For peer review only - http://bmjopen.bmj.com/site/about/guidelines.xhtml

- Utilises data from a nationally representative repeated panel data at individual/household level over a 10-year period (5 survey waves).
- Employed a fully Bayesian space-time shared component model to produce more stable estimates of malnutrition burden at provincial and district level among children under five years of age in South Africa.
- Panel design allows assessment of change in malnutrition burden within the same individuals/households observed at multiple time points.
- Missing or invalid weight/height measurements may have introduced selection bias if not missing at random, and may thus have affected both the internal validity and the representativeness the findings.
- As primary panel study was not designed/powered for provincial and lower geographic level analysis, we cannot discount the resultant impact on precision/random variability when analysing at provincial/district level (administrative tier just below province) and further stratification by socio-demographic correlates.

Background

Despite reductions in malnutrition 150.8 million children (22.2%) under five are stunted and a further 50.5 million children are wasted [1]. Furthermore rapidly rising trend in overweight and obesity in children and adults [2-4] [5] has emerged as one of the most serious global public health issues of the 21st century [6]. Sub-Saharan Africa (SSA) has among the highest levels of child malnutrition[1] globally. This problem is particularly illustrated by South Africa [7], a middle income country with high levels of wealth/economic inequality that is undergoing rapid socioeconomic and lifestyle changes that have precipitated a nutritional transition, high prevalence of overweight/obesity in children [8]. The dual burdens of undernutrition and overweight/obesity are not distributed in a spatially homogenous manner [9], and the health risks associated with malnutrition vary by age, gender, ethnicity and geographical location [10].

Progress to tackle all forms of child malnutrition remain much too slow [1]. In order to support the delivery of public health interventions that will be most effective at reducing malnutrition, an understanding of the geographical distribution of malnutrition is required. Limited data are collected at lower administrative unit level making it difficult to identify specific groups of high-risk individuals and thus, determine the most suitable and

[1] Child malnutrition is defined as a pathological state as a result of inadequate nutrition, including undernutrition due to insufficient intake of dietary energy and other key nutrients resulting in stunting (low height for age) or wasting (low weight-for-length) and overweight and obesity due to excessive consumption of dietary energy and reduced levels of physical activity.

For peer review only - http://bmjopen.bmj.com/site/about/guidelines.xhtml

cost-effective opportunities and solutions. Previous studies of nutritional status of the South African population have mostly focused on adults [11 12]. Here we use a large, nationally-representative data from multiple rounds of the National Income Dynamics Study over the period 2008 to 2017 to assess space-time trends in the burden of malnutrition and associated risk factors among children under 5 years of age in South Africa.

Methods

Data

Data were taken from the five panel (cross-sectional) waves of the South African National Income Dynamics Study (SA-NIDS)[13 14] (http://www.nids.uct.ac.za/nids-data/data-access; https://www.datafirst.uct.ac.za/dataportal/index.php/catalog/NIDS/), the first national panel study in South Africa. SA-NIDS was undertaken by the South African Labour and Development Research Unit based at the School of Economics at the University of Cape Town. The surveys took place in 2008, 2010-11, 2012, 2014-15 and 2017. These are named waves 1-5 respectively. A detailed description of the data collection methods can be found elsewhere [26]. In short, a stratified, two-stage random cluster sample design was employed to sample households for inclusion at baseline using proportionally allocated stratification, based on the 52 district councils (DCs) in South Africa[13]. Within each DC (primary sampling unit [PSU]), clusters of dwelling units were systematically drawn. The household level response rate was 69% and the individual response rate within households was 93%. Survey enumerators attempt to collect weight and height measurements of all individuals (including children) in selected households.

Study population

We restricted our analysis to children <5 years of age.

Outcomes

We calculated height for age (HA) and BMI-for-age (BA) z-scores using the WHO 2007 growth standards [15 16]. We generated z-scores by transformation of child anthropometric data using the "lambda mu sigma" method ('zanthro' function in Stata 15). As recommended, weight-for-length was used in children 0 to <2 years of age, and BMI-for-age in children 2 years of age and older [17]. We defined obesity as weight-for-length z-score ≥+2 for children under 2 years of age and BMI for age z-score of >2+ for children age 2 and older [17]. We defined wasting as weight-for-length z-score < -2 for children under 2 years of age and thinness as BMI for age z-score < -2 for children 2 years and older. Stunting was defined as HA z-score of < -2.

For peer review only - http://bmjopen.bmj.com/site/about/guidelines.xhtml

Geographic and socio-demographic variables

To identify relevant inequalities under-nutrition and obesity indicators were stratified temporally (survey year), geographically (province and residence location type: urban informal settlements, urban formal, tribal/rural) and by important socio-demographic categories (Gender: Female/Male; ethnicity: Black/African, Coloured, Indian/Asian, White/Caucasian; Maternal: age; education status; body mass index; household socio-economic status (income) classified into quantiles [1=lowest, 5=highest].

Data analysis

Analyses were performed using Stata software version 15 [StataCorp. 2017. Stata Statistical Software: Release 15. College Station, TX: StataCorp LLC]. Given the multistage random sampling design of the primary study, clustering and survey design effects were accounted for using sample weights to estimate standard error and 95% confidence intervals (CIs) around mean anthropometric z-score point estimates, both overall and stratified by other socio-demographic variables such ethnicity and gender, socio-economic status, and residence location type. Extrapolated population totals of malnourished children (< 5) by yearly age were estimated using the survey weights.

Space-time Bayesian modelling: We assessed for the presence of univariate and bivariate spatial autocorrelation for the three anthropometric classifications using Moran's I statistics. This analysis was performed using GeoDa [18]. Based on these tests it appeared that there was no prominent bivariate spatial autocorrelation between the three measures but that each measure was significant heterogeneous across space to warrant the use of a spatial model (Supplementary Material 1).

We employed Bayesian spatial-temporal modelling approach in an attempt to stabilise estimates at district level given that the primary sampling design was not developed to provide point estimates at this level of geographic disaggregation and resultant zero prevalence estimates for particular districts and waves. We choose a Bayesian spatial-temporal formulation to model each of the anthropometric outcomes independently using an autoregressive approach, suggested by a recent methodological comparison [19], which fuses ideas from autoregressive time series to link information in time and by spatial modelling to link information in space. We also opted for an autoregressive model which only included the spatial term for every period and did not include a heterogeneous term which resulted in a more parsimonious description of risk [20].

For peer review only

Let Y_{ij} be the number of stunted, thin or obese children for the ith area and jth period, i =1,...,I, j =1,...,J, and ni j the total number of children sampled in a given area and period. We assumed that $Y_{i\,j}$ follows a binomial distribution i.e. Y_{ij} ~ binomial (nij, πij), i =1,...,53, j =1,..., 5, where π it is the risk (prevalence) of stunting, thinness or obesity in region i in period j. As per Martínez-Beneito et al. [20] we define the logit of the prevalence for a given anthropometric outcome for the first wave (or period) as the sum of an intercept and two random effects, namely:

$$\pi_{i1} = \mu + \alpha_1 + (1-\rho^2)^{-1/2} \cdot (\theta_{i1} + \phi_{i1}),\ i = 1,\ldots,I$$
$$\theta_{i1} \sim \text{Normal}(0,\ \sigma^2_\theta),\ i = 1, \ldots, I$$
$$\phi_1 = (\phi_{11}, \ldots, \phi_{I1}) \sim \text{CAR.normal}\ (\sigma^2_\phi)$$

and subsequent time periods 2,…,J as:

$$\pi_{ij} = \mu + \alpha_j + \rho \cdot (\pi_{i(j-1)} - \mu - \alpha_{j-1}) + \theta_{ij} + \phi_{ij},\ \text{for } i = 1, \ldots, I \text{ and } j=2,\ldots,J$$
$$\theta_{ij} \sim \text{Normal}(0,\ \sigma^2_\theta),\ \text{for } i = 1, \ldots, I \text{ and } j=2,\ldots,J$$
$$\phi_j \sim \text{CAR.normal}\ (\sigma^2_\phi),\ \text{for } j=2,\ldots,J$$
$$\alpha = (\alpha_1, \alpha_2, \ldots, \alpha_J) \sim \text{CAR.normal}(\sigma^2_\alpha)$$

where ϕ, the spatial random effect, assumes an intrinsic Gaussian conditionally autoregressive distribution [21] (abbreviated above as CAR.normal), whereby the spatially correlated random effect of the ith region (φ_i) is based on the sum of its weighted neighbourhood values. We used an adjacency matrix of common boundaries (neighbours) of a given region when modelling this parameter. The heterogeneous or unstructured random effect is represented by θ and is included to ensure sufficient flexibility for estimates in close regions that is not captured by the spatially structured term. The spatial and heterogeneous random effect terms are both independent in time and mutually independent in every period. Furthermore, ρ corresponds to the temporal correlation term, μ models the mean level of risks for all the periods and regions and α_1 models the mean deviation of the risks in the first period from the mean level for all of them. A first-order random walk CAR.normal was also used as prior distribution for α.

The following prior distributions were assumed for the parameters defined above:

$$\sigma^2_\theta, \sigma^2_\phi, \sigma^2_\alpha \sim \text{Gamma}(0.5, 0.0005)$$
$$\rho \sim \text{Uniform}(-1,1)$$
$$\mu \sim \text{Normal}(0,C)$$

For peer review only

For peer review only - http://bmjopen.bmj.com/site/about/guidelines.xhtml

The prior distribution on the temporal correlation parameter (ρ) was chosen to ensures the stationarity of the time series, considering that it has an order 1 autoregressive structure. We chose inverse gamma distributions for the variance parameters with values of 0.5 and 0.0005 as suggested by Wakefield et al [22].

Survey weighted prevalences were applied to sample size totals by district and panel to obtain a survey weighted numerator counts (Y_{ij} above) by malnutrition type in the binomial distribution. The space-time models were fitted in WINBUGS using Markov chain Monte Carlo (MCMC) simulation and non-informative priors. The full WINBUGS model code is provided in the Supplementary Material (2. We used two-chain MCMC simulation for parameter estimation and Gelman-Rubin statistics/plots [23] were used to assess model convergence/stability and where the Monte Carlo error for each parameter of interest was less than 5% of the sample standard deviation (Supplementary Material 3). For model validation, we firstly compared the observed and fitted prevalence values to assess overall model adequacy and fit (using model Deviance Information Criterion [DIC] and comparison of observed vs fitted prevalence estimate) and secondly, performed an out of sample validation using a random 10% sample with observed data. These additional analyses can be found in the Supplementary Material 4. The model was run until the Monte Carlo error for each parameter of interest was less than 5% of the sample standard deviation. Posterior prevalence estimates and 95% Bayesian credibility intervals for stunting, thinness/wasting and obesity t provincial and district level were mapped using ArcGIS 10.6.1 [ESRI 2011. ArcGIS Desktop: Release 10. Redlands, CA: Environmental Systems Research Institute].

Risk factors analysis: Survey weighed two-way tabulations of key socio-demographic covariates, year and child nutritional status were performed to produce correctly weighted prevalence estimates. Tests of independence for complex survey data survey (weighted Pearson's chi-square test) was utilised to assess the significance of bivariate associations between malnutrition burden and year as well as socio-demographic covariates.

Ethical approval: Approval for the primary study was granted by the Ethics Committee of the University of Cape Town. The current analysis is a secondary data analysis of an open access dataset and does not require further ethical approval.

Patient and Public Involvement: As this was a data analysis utilising secondary data from a national community based panel survey, the development of the research question was not informed by the study subjects. Likewise, we could not involve study participants in the design of this study. Study participants were not involved in conduct of the primary study. Results will be disseminated in the form of peer reviewed article as well as through presentation to senior members of our National Department of Health and KwaZulu-Natal Department of Health.

For peer review only - http://bmjopen.bmj.com/site/about/guidelines.xhtml

194 ## Results

195 ### Study population

196 The sample of children <5 years of age in the 7,301 households included in the SA-NIDS survey increased from
197 3,254 children at baseline (2008) to 4,710 children in wave 5 (2017) (Supplementary Material 5). With the
198 exception of children under 1 year of age and survey wave 2 in 2010/11, valid weight and height measurements
199 were taken from 85-90% of children sampled between the age of 1 and 5 on average (Supplementary Material 5).
200 An additional sensitivity analysis comparing distributions of various socio-demographic characteristics by
201 missing weight/height status was also performed (Supplementary Section 6). These findings suggest that
202 children with missing weight/height were largely missing at random, with the exception of age and province.

203 ### Temporal changes in burden of malnutrition from 2008 to 2017)

204 Between 2008 and 2017, the prevalence of stunting among children aged under 5 years decreased from 11.0% to
205 7.6% (p=0.007) (Table 1). Over the same period, both the prevalence of wasting/thinness (and the prevalence of
206 obesity decreased (from 5.2 to 3.8%, p= 0.131 and 14.5% to 12.9%, p= 0.312 respectively). The prevalence of
207 thinness was higher (p<0.001) in children under 2 years of age (8% [95%CI: 5.0-11.8%] in 2008; 6% [95%CI:
208 4.1-9.1%] in 2017) compared to 4% (95%CI: 3.2-6.2%) in 2008 and 3% (95%CI: 2.0-4.5%) in 2017 among
209 children 2 years and older . The prevalence of obesity was also higher among children under 2 years of age and
210 increased over the study period (18.4% [95%CI: 13.7-24.1%] in 2008 vs 21.7% [95%CI: 19.3-24.2%]in 2017,
211 p=0.091).

212

213 ### Space-time burden of malnutrition at provincial and district level

214 *Under nutrition:* In 2008 and 2017, the highest prevalence of stunting was estimated in the Free State (17.1% and
215 10.5% respectively) followed by Eastern Cape (14.7% and 8.5% respectively) and Limpopo (14.0% and 9.3%
216 respectively) (Figure 1 – panel a1). One district each in Free State (Mangaung Metropolitan), Limpopo (Vhembe)
217 and Northern Cape (Pixley ka Seme) had a posterior median smoothed prevalence of stunting in excess of 15% in
218 2017 (Figure 1– panel a2). Gauteng had the highest burden of thinness/wasting in 2008 (9.6%) followed by North
219 West province (9.3%) and Free State (7.6%) (Figure 2a). By 2017 2017 the highest burden was observed in
220 Western Cape (at 5.6%) followed by Northern Cape (4.9%) and North West (4.6%) (Figure 2b). The estimates
221 suggest that at provincial level 7 of 9 provinces were above the 5% target threshold for wasting in 2017 (only
222 Eastern Cape and KwaZulu-Natal were the exceptions). There appeared to be a general gradient of higher burden

For peer review only

of thinness/wasting in the western half of country in 2017 (lower burden in KwaZulu-Natal and northern districts of Eastern Cape) (Figure 2b). The three districts with the highest posterior median smoothed prevalence of wasting in 2017 were Amathole [EC] (12.6%), Siyanda [NC] (11.4%) and Eden [WC] (10.9%) (Figure 2b). Similarly, to the provincial level finding above, only 16 of 52 districts had an estimated wasting prevalence below 5% in 2017.

Obesity: In 2008, the highest posterior median smoothed prevalence of obesity was estimated in Eastern Cape (22.5%) followed by Western Cape (18.4%) and KwaZulu-Natal (17.6%) (Figure 3a). A decade later in 2017, the highest prevalence of childhood obesity was still estimated to be in the Eastern Cape (15.6%), followed by KwaZulu-Natal (15.1%)and Western Cape (15.0%). In contrast to the wasting gradient highlighted above (higher burden in the western half of the country), the burden of obesity in 2017 appeared to be much higher in the eastern half of the country (particularly KwaZulu-Natal and Eastern Cape) (Figure 3b). The 4 highest obesity prevalence districts in 2017 were located in KwaZulu-Natal (Sisonke [26.2%] and eThekwini Metropolitan [25.7%) and Eastern Cape (Buffalo City Metropolitan [29.1%] and O.R Tambo [25.9%]).

Figure 1: Bayesian posterior median smoothed prevalence of stunting by province (and wave) and district level prevalence (equal intervals, 2017) among children <5 years

Figure 2: Bayesian posterior median smoothed prevalence of thinness/wasting by province (and wave) and district level prevalence (equal intervals, 2017) among children <5 years

Figure 3: Bayesian posterior median smoothed prevalence of obesity by province (and wave) and district level prevalence (equal intervals, 2017) among children <5 years

Factors associated with child nutritional status

A bivariate analysis of demographic, maternal, socio-economic and household factors at individual nutritional status level suggests that African ethnicity ($p<0.001$), male gender ($p=0.002$), low birth weight (<0.001), residing in lower socio-economic status household ($p<0.001$), province of residence ($p=0.012$), lower maternal/paternal education status ($p<0.001$, 0.020 respectively) and residence in a rural/tribal authority area ($p<0.001$) were significantly associated with stunting (Table 2). Children living in lower income households ($p=0.053$), lower food security (as measured through child hunger in last year) ($p<0.001$), province of residence ($p=0.002$), having a younger mother (<20) ($p=0.012$) and mother having a lower BMI classification ($p=0.005$) was significantly

For peer review only - http://bmjopen.bmj.com/site/about/guidelines.xhtml

associated with thinness/wasting status. Children of African ethnicity ($p<0.001$), higher birth weight ($p=0.006$), living in lower income households ($p=0.001$) in KwaZulu-Natal and Eastern Cape ($p<0.001$) as well as paternal educational attainment ($p=0.033$) were significantly associated with obesity status (Table 2).

Discussion

***Main findings**:* The present study illustrates that while stunting has declined among South African children over the last 10 years, wasting and obesity appear largely unchanged, suggesting that development and public health interventions have had a variable impact. Stunting prevalence appears relatively evenly spread across South Africa, but obesity burden is more pronounced in the east of the country, whereas thinness/wasting is more pronounced in the west, with only 16 of 52 districts with estimated wasting prevalence below the 5% (WHO 2025 target threshold) in 2017. A concerning pattern observed was the increase prevalence of obesity in children under the age of two years. Key socio-demographic factors associated with malnutrition status were identified which likely underpins the spatial patterns (and heterogeneity) observed across the country. African children with lower birth weights residing in lower income households in rural areas with less educated mothers and fathers were particular more likely to be stunted. Children in lower income, food insecure households with malnourished young mothers appeared particularly more likely to be thin/wasted while African children, with higher birth weights, living in lower income households in KwaZulu-Natal and Eastern Cape were also more likely to be obese. Furthermore, low household income appeared to be positively associated with all 3 nutritional types. Declining childhood stunting rates from 2008-2017 may well have resulted from government initiatives to support food security and child health (among other things), but our findings of distinct geographic and socio-demographic variability in undernutrition and obesity rates suggest that tackling malnutrition in South Africa is complex. Models and targets for nationally-driven intervention need to be carefully specified according to local environments and socio-economic profiles.

***Contribution to existing literature**:* Two previous studies in South Africa among primary school aged children dating back 25+ years (1993and 1994 respectively) utilised cross sectional data[24 25], thus limiting insight into temporal trends. Furthermore, the study by Jinabhai et al. [19] was restricted to KwaZulu-Natal limiting national representativeness. Another cross sectional study in South African in 2001-2003 among primary school children in five South African Provinces suggested that relative to 1993 prevalence of undernutrition had decreased while obesity had increased[25 26]. Thus these previous data are now outdated, were largely focused on primary school aged children as well as cross sectional in nature and geographically restricted.

For peer review only - http://bmjopen.bmj.com/site/about/guidelines.xhtml

This is also the first spatial-temporal Bayesian shared component analysis of malnutrition trends among children in South Africa utilising geographically representative repeated panel data over a 10-year period. The current study focusing on children under 5 year of age suggests that there is prominent geographic heterogeneity in malnutrition burden in South Africa in this youngest age group. This is in line with findings from other settings in Africa that have documented similar spatial heterogeneity [27] and persistence of these malnutrition inequalities has been demonstrated in an 80 country study further highlighting this ongoing public health conundrum [28 29]. Our results demonstrate a strong west to east gradient of higher underweight burden on the western side of South Africa and greater obesity on the eastern seaboard (Eastern Cape and KwaZulu-Natal). A map of poverty and inequality in South Africa [2] illustrates the co-existence of high levels of poverty and inequality in many parts of KwaZulu-Natal and the Eastern Cape with high levels of overweight/obesity. This is further confirmed by our individual child level analysis which suggested a significantly higher obesity prevalence in lower income households. Metropolitan areas displayed high levels of nutritional inequality that complement national studies of poverty and inequality [30].

Under and over nutrition status appeared positively associated with lower household income classification. This finding of stunting and wasting disproportionately affecting the poor has been often demonstrated [31]. Other studies in Africa in particular have documented similar patterns i.e. children living in low SES households, children who live in peripheral areas and whose mothers had little or no schooling were at significantly higher risk of malnutrition [32]. The inconsistent challenges facing health authorities are occurring in the face of rapid urbanization and industrialization that simultaneously attract both the rich and the poor to live in the same geographic districts [33].The heterogeneous geographic relationship between household income and undernutrition is also affected by the allocation of household income that is a function of maternal education, access to markets, infrastructure and sanitation [34]. Additionally, these data suggest that there is a strong and highly significant association between higher food insecurity (child hunger frequency in the preceding year) and increased thinness/wasting. Community and government based packages of support need to be highly targeted to the poorest and most food insecure households to further reduce inequality in this regard and maximise reductions in malnutrition.

Our findings suggest that children with low birth weight (due to pre-term delivery, fetal/intrauterine growth restriction or a combination of the two) were significantly more likely to be stunted than normal weight babies and

[2] https://southafrica-info.com/people/mapping-poverty-in-south-africa/

For peer review only - http://bmjopen.bmj.com/site/about/guidelines.xhtml

this has been demonstrated in many other low and middle income settings (for example [35]). Socioeconomic status/factors are known risk factors for LBW [36]and may in part explain the significant association found between stunting and lower household income. South Africa has the higher number of incident and prevalent HIV infections globally [37]. A further important contextual risk factor for LBW is maternal HIV status. A systematic review and large observational studies focussing on low and middle incoming countries, suggest a strong and significant association between maternal HIV infection and LBW [38 39]. Evidence from South Africa also suggests the anthropometric z-score of HIV-infected children appear to be consistently lower when compared to HIV-exposed but uninfected children [40]. We also observed a significantly higher prevalence of stunting among male children which has been demonstrated previously in a meta-analysis for sub-Saharan Africa [41], the suggested cause of which might be that male children are more vulnerable to health inequalities relative to female children of the same age. Strengthening community-based packages of care and community health worker (CHW) performance/skills in rural and high burden geographies are key strategies to improve primary health care delivery through better identification of women at higher risk of poor birth outcomes (e.g. HIV positive, history of previous poor birth outcomes and/or currently malnourished), higher referral rates for facility births, and improved linkage to other health as well as social services [42]. Lastly given the high adolescent fertility rates in many parts of South Africa [43], there is also much scope to improve CHW identification of households with higher risk malnourished adolescent girls prior to pregnancy to ensure more optimal linkage to government and social support to ensure adequate nutrition as well as improved awareness regarding family planning practices e.g. ensuring adequate birth spacing [44].

Obesity in children has a complex aetiology that includes a wide range of socioeconomic, demographic, environmental and cultural variables [45]such as household composition, mother's education, household income, household size, environmental factors, rural versus urban location, and sanitation [9 46]. The high burden of obesity is likely associated with a progressive increases in the per capita food supply and consumption of high calorific foods (e.g. fat, sugar, fast and/or processed foods) in South Africa[47]. This rapidly changing dietary pattern has, in part, been attributed to urbanisation, growing and expanding supermarkets /formal food retailers, and the availability of fast/processed foods [48]. An interesting finding in these data was the significant positive association between child obesity status and residing in a lower income household. This association has been demonstrated previously [49-51] and this evidence base is growing. This conforms with the idea that lower and higher income households/families often have a higher obesity risk than middle income households i.e. so called U-shaped association. Lower income or economically deprived families often replace health fresh food options with cheaper

For peer review only - http://bmjopen.bmj.com/site/about/guidelines.xhtml

339 and more calorific processed foods [50]. Multiple studies have demonstrated that the majority of low-income South
340 Africans have a low dietary diversity, and, therefore, consume a limited food range consisting predominantly of a
341 starchy staple such as bread and maize, with low intakes of vegetables and fruit [47]. Future work will characterise
342 food purchasing patterns (and changes over time) among households in South Africa which will be compared with
343 paired longitudinal anthropometric measurements to identify specific dietary patterns associated with child
344 nutritional status.

345 Lastly and contextually, body mass is culturally influenced in South Africa, and the high level of obesity in
346 KwaZulu-Natal and Eastern Cape may at least in part be a result of cultural beliefs that associate overweight with
347 wealth and good health [52]. Geographic patterns of higher obesity in South Africa appeared to overlap areas of high
348 poverty particular on the eastern side of the country[3] and thus not solely concentrated among higher socio-
349 economic households.

350 ***Strengths:*** To our knowledge this is the first spatial-temporal analysis of malnutrition trends among children
351 under five years of age in South Africa. We used standardised anthropometric measurements of children and their
352 mothers from a nationally representative repeated panel data over a 10-year period. The panel nature of the design
353 allows assessment of change in malnutrition burden within the same individuals/households observed at multiple
354 time points. A further strength was the implementation of a fully Bayesian space-time shared component model to
355 produce more stable joint estimates of malnutrition by province, district and year.

356 ***Weaknesses***: The study has several limitations. Firstly, missing or invalid weight/height measurements (especially
357 in wave 2, and among infants – Supplementary Material 2) may have introduced selection bias (if not missing at
358 random), and may thus have affected both the internal validity and the representativeness the findings in the
359 broader South African context. Secondly as the primary panel study was not designed/powered for provincial
360 [13]and lower geographic level analysis, we cannot discount the resultant impact on precision/random variability
361 when analysing at provincial/district level (administrative tier just below province) and further stratification by
362 socio-demographic correlates. Thirdly, we cannot discount the effect of inter-observer variability across different
363 study districts, despite extensive interviewer training and standardization of study protocols. All anthropometric
364 measurements (e.g. weight, height) were taken in duplicate in NIDS [26] which would have ensured better
365 reliability.

[3] https://southafrica-info.com/people/mapping-poverty-in-south-africa/

For peer review only - http://bmjopen.bmj.com/site/about/guidelines.xhtml

Cost of malnutrition, policy and research needs: Estimating the cost of child malnutrition in South Africa is extremely complicated and no locally-determined cost data exist. Data from the United States, suggest that the incremental lifetime direct medical cost for a 10-year-old obese child relative to a 10-year-old normal weight child ranges from USD 12 660 to USD 19 630 [53]. Estimates of the cost of treating wasted children are approximately USD 200 per child [54] while stunting has been consistently linked to worse economic outcomes in adulthood [55] and estimates suggest that, on average, the future per capita income penalty for a stunted individual could be as large as 9-10% in SSA [56]. Urgent investments are needed to accelerate the reduction of all forms of malnutrition, as well as to curb the obesity epidemic among young children in South Africa. There is also considerable evidence indicates that childhood wasting and stunting can be reduced by 60% and 20% respectively using ten nutrition-specific interventions [57], with an estimated return on investment (ROI) of 18:1, i.e. for USD 1 spent on implementing effective programmes there would be USD 18 return in future economic benefits [58]. Very few obesity prevention interventions targeting children have been effective and a comprehensive multifaceted strategy tackling diet, physical inactivity, coupled with psychosocial support and local food environment change may prove more effective. Nutrition policies tackling child obesity must promote household nutrition security and healthy growth, decrease overconsumption of nutrient-poor foods, better shield children from increasingly pervasive marketing of energy-dense, nutrient-poor foods and sugar sweetened beverages as well as reduction of growing physical inactivity [59].

Our findings suggest the need to implement evidence-based child health strategies and policy (e.g. further social grant support to vulnerable and impoverished households) that is tailored to specific geographies and socially disadvantaged sub-populations. A higher prevalence of child thinness/wasting among younger mothers (<25) in poorer, food insecure household, highlights the importance of policies that enable younger mothers to adequately care for their children in all settings. Integrated nutrition programs in low and middle income countries (LMIC) have had a substantial impact on child nutrition and health via a combination of multisector targeted interventions [60]. Furthermore implementation and/or strengthening of school-based food program can provide a launching pad for preventive programs including education and awareness, provision of healthier/more nutrition food options and micronutrient supplementation, deworming, increased immunization coverage and improved growth monitoring as well as counselling [60]. This may be especially true of obese children where high prevalence was observed in higher income households with higher food purchasing power and where local food environments are likely is likely to be an important contextual determinant. A further contextual trend which may further compound

For peer review only - http://bmjopen.bmj.com/site/about/guidelines.xhtml

this problem is the rapidly rising median household income observed over the period (from ZAR1400 in 2008 to ZAR 3640 by 2017).

Conclusions

The heterogeneity of malnutrition is a feature of spatial inequality and rapid urbanization that has manifested in widening levels of inequality in South Africa's districts and a need to reassess where nutrition programmes need to be further decentralised to the highest risk municipalities and local communities to maximise effectiveness. This work provides the first district level ranking of childhood overweight, thinness/wasting and stunting and allows a differentiated pro-active tailored intervention to be developed for each municipal district. The dual epidemic of undernutrition and overweight/obesity requires differential geographical policy inputs in metropolitan areas and districts across the rural-urban divide. The current and future health cost of malnutrition among South African children is likely substantial based on previous costing estimates. There is an urgent need to address nutrition problems among preschool aged children in South Africa and other low and middle income countries. Effective public health planning and geographically/contextually tailored interventions are required at sub-national level to address this challenge. The analytical framework employed in this study we believe will have definite utility in other settings.

References

1. Development Initiatives. 2018 Global Nutrition Report: Shining a light to spur action on nutrition. Bristol, UK: Development Initiatives, 2018.
2. Finucane MM, Stevens G, Cowan M, et al. Global Burden of Metabolic Risk Factors of Chronic Diseases Collaborating Group (Body Mass Index) National, regional, and global trends in body-mass index since 1980: systematic analysis of health examination surveys and epidemiological studies with 960 country-years and 9.1 million participants. *Lancet* 2011;377(9765):557-67.
3. Collaborators TGO. Health Effects of Overweight and Obesity in 195 Countries over 25 Years. *New England Journal of Medicine*;0(0):null. doi: 10.1056/NEJMoa1614362
4. Collaboration NRF. Trends in adult body-mass index in 200 countries from 1975 to 2014: a pooled analysis of 1698 population-based measurement studies with 19· 2 million participants. *The Lancet* 2016;387(10026):1377-96.
5. Bleich SN, Vercammen KA, Zatz LY, et al. Interventions to prevent global childhood overweight and obesity: a systematic review. *The Lancet Diabetes & Endocrinology* 2017
6. Malik VS, Willett WC, Hu FB. Global obesity: trends, risk factors and policy implications. *Nature Reviews Endocrinology* 2013;9(1):13-27.
7. Bain LE, Awah PK, Geraldine N, et al. Malnutrition in Sub–Saharan Africa: burden, causes and prospects. *Pan African Medical Journal* 2013;15(1)
8. Rossouw HA, Grant CC, Viljoen M. Overweight and obesity in children and adolescents: The South African problem. *South African journal of science* 2012;108(5-6):31-37.
9. Keino S, Plasqui G, Ettyang G, et al. Determinants of stunting and overweight among young children and adolescents in sub-Saharan Africa. *Food and nutrition bulletin* 2014;35(2):167-78.

For peer review only - http://bmjopen.bmj.com/site/about/guidelines.xhtml

10. Osgood-Zimmerman A, Millear AI, Stubbs RW, et al. Mapping child growth failure in Africa between 2000 and 2015. *Nature* 2018;555(7694):41.
11. Cois A, Day C. Obesity trends and risk factors in the South African adult population. *BMC obesity* 2015;2(1):42.
12. Sartorius B, Veerman LJ, Manyema M, et al. Determinants of obesity and associated population attributability, South Africa: Empirical evidence from a national panel survey, 2008-2012. *PloS one* 2015;10(6):e0130218.
13. Leibbrandt M, Woolard I, de Villiers L. Methodology: Report on NIDS wave 1. *Technical paper* 2009;1
14. Southern Africa Labour and Development Research Unit. National Income Dynamics Study 2017, Wave 5 [dataset]. Version 1.0.0 In: Pretoria: Department of Planning M, and Evaluation [funding agency]. Cape Town: Southern Africa Labour and Development Research Unit [implementer], 2018. , ed.: Cape Town: DataFirst [distributor], 2018., 2018.
15. Health WHONf. WHO child growth standards: growth velocity based on weight, length and head circumference: methods and development: World Health Organization 2009.
16. Organization WH, Health WHONf. WHO child growth standards: head circumference-for-age, arm circumference-for-age, triceps skinfold-for-age and subscapular skinfold-for-age: methods and development: World Health Organization 2007.
17. Gibson RS. Principles of nutritional assessment: Oxford university press, USA 2005.
18. Anselin L, Syabri I, Kho Y. GeoDa: an introduction to spatial data analysis. *Geographical analysis* 2006;38(1):5-22.
19. Anderson C, Ryan L. A comparison of spatio-temporal disease mapping approaches including an application to ischaemic heart disease in New South Wales, Australia. *International journal of environmental research and public health* 2017;14(2):146.
20. Martínez-Beneito MA, López-Quilez A, Botella-Rocamora P. An autoregressive approach to spatio-temporal disease mapping. *Statistics in medicine* 2008;27(15):2874-89.
21. Besag J, York J, Mollié A. Bayesian image restoration, with two applications in spatial statistics. *Annals of the institute of statistical mathematics* 1991;43(1):1-20.
22. Wakefield J, Best N, Waller L. Bayesian approaches to disease mapping. *Spatial epidemiology: methods and applications* 2000:104-07.
23. Gelman A, Rubin DB. Inference from iterative simulation using multiple sequences. *Statistical science* 1992;7(4):457-72.
24. Zere E, McIntyre D. Inequities in under-five child malnutrition in South Africa. *International journal for equity in health* 2003;2(1):7.
25. Jinabhai C, Taylor M, Sullivan K. Implications of the prevalence of stunting, overweight and obesity amongst South African primary school children: a possible nutritional transition? *European journal of clinical nutrition* 2003;57(2):358.
26. Armstrong ME, Lambert MI, Lambert EV. Secular trends in the prevalence of stunting, overweight and obesity among South African children (1994–2004). *European journal of clinical nutrition* 2011;65(7):835.
27. Kandala N-B, Madungu TP, Emina JB, et al. Malnutrition among children under the age of five in the Democratic Republic of Congo (DRC): does geographic location matter? *BMC public health* 2011;11(1):261.
28. Bredenkamp C, Buisman LR, Van de Poel E. Persistent inequalities in child undernutrition: evidence from 80 countries, from 1990 to today. *International Journal of Epidemiology* 2014;43(4):1328-35.
29. Di Cesare M, Bhatti Z, Soofi SB, et al. Geographical and socioeconomic inequalities in women and children's nutritional status in Pakistan in 2011: an analysis of data from a nationally representative survey. *The Lancet Global Health* 2015;3(4):e229-e39.
30. Ataguba JE, Akazili J, McIntyre D. Socioeconomic-related health inequality in South Africa: evidence from General Household Surveys. *International journal for equity in health* 2011;10(1):48.

For peer review only - http://bmjopen.bmj.com/site/about/guidelines.xhtml

31. Van de Poel E, Hosseinpoor AR, Speybroeck N, et al. Socioeconomic inequality in malnutrition in developing countries. *Bulletin of the World Health Organization* 2008;86:282-91.
32. Delpeuch F, Traissac P, Martin-Prével Y, et al. Economic crisis and malnutrition: socioeconomic determinants of anthropometric status of preschool children and their mothers in an African urban area. *Public health nutrition* 2000;3(1):39-47.
33. Oyvat C. Agrarian structures, urbanization, and inequality. *World Development* 2016;83:207-30.
34. Hoddinott J, Haddad L. Does female income share influence household expenditures? Evidence from Côte d'Ivoire. *oxford Bulletin of Economics and Statistics* 1995;57(1):77-96.
35. Aryastami NK, Shankar A, Kusumawardani N, et al. Low birth weight was the most dominant predictor associated with stunting among children aged 12–23 months in Indonesia. *BMC Nutrition* 2017;3(1):16.
36. de Bernabé JV, Soriano T, Albaladejo R, et al. Risk factors for low birth weight: a review. *European Journal of Obstetrics & Gynecology and Reproductive Biology* 2004;116(1):3-15.
37. Frank TD, Carter A, Jahagirdar D, et al. Global, regional, and national incidence, prevalence, and mortality of HIV, 1980–2017, and forecasts to 2030, for 195 countries and territories: a systematic analysis for the Global Burden of Diseases, Injuries, and Risk Factors Study 2017. *The Lancet HIV* 2019
38. Xiao P-L, Zhou Y-B, Chen Y, et al. Association between maternal HIV infection and low birth weight and prematurity: a meta-analysis of cohort studies. *BMC pregnancy and childbirth* 2015;15(1):246.
39. Althabe F, Moore JL, Gibbons L, et al. Adverse maternal and perinatal outcomes in adolescent pregnancies: The Global Network's Maternal Newborn Health Registry study. *Reproductive health* 2015;12(2):S8.
40. Patel D, Bland R, Coovadia H, et al. Breastfeeding, HIV status and weights in South African children: a comparison of HIV-exposed and unexposed children. *Aids* 2010;24(3):437-45.
41. Wamani H, Åstrøm AN, Peterson S, et al. Boys are more stunted than girls in sub-Saharan Africa: a meta-analysis of 16 demographic and health surveys. *BMC pediatrics* 2007;7(1):17.
42. Redding S, Conrey E, Porter K, et al. Pathways community care coordination in low birth weight prevention. *Maternal and child health journal* 2015;19(3):643-50.
43. Statistics South Africa. Recorded live births 2012. www.statssa.gov.za/publications/P0305/P03052011.pdf. 2012
44. Organization WH. WHA Global nutrition targets 2025: low birth weight policy brief. Geneva: WHO, 2014.[Accessed on: June 1st, 2017].
45. Monasta L, Batty G, Cattaneo A, et al. Early-life determinants of overweight and obesity: a review of systematic reviews. *Obesity reviews* 2010;11(10):695-708.
46. Kruger R, Kruger H, Macintyre U. The determinants of overweight and obesity among 10-to 15-year-old schoolchildren in the North West Province, South Africa–the THUSA BANA (Transition and Health during Urbanisation of South Africans; BANA, children) study. *Public health nutrition* 2006;9(3):351-58.
47. Pereira LM. The future of South Africa's food system: What is research telling us. *SA Food Lab South Africa* 2014
48. McLachlan M, Landman A. Nutrition-sensitive agriculture–a South African perspective. *Food security* 2013;5(6):857-71.
49. Brophy S, Cooksey R, Gravenor MB, et al. Risk factors for childhood obesity at age 5: analysis of the millennium cohort study. *BMC public health* 2009;9(1):467.
50. Brunt H, Lester N, Davies G, et al. Childhood overweight and obesity: is the gap closing the wrong way? *Journal of public health* 2008;30(2):145-52.
51. Kim TJ, von dem Knesebeck O. Income and obesity: what is the direction of the relationship? A systematic review and meta-analysis. *BMJ open* 2018;8(1):e019862.
52. Rossouw HA, Grant CC, Viljoen M. Overweight and obesity in children and adolescents: the South African problem: review article. *South African Journal of Science* 2012;108(5-6):1-7.

For peer review only - http://bmjopen.bmj.com/site/about/guidelines.xhtml

53. Finkelstein EA, Graham WCK, Malhotra R. Lifetime direct medical costs of childhood obesity. *Pediatrics* 2014:peds. 2014-0063.
54. Horton S, Shekar M, Ajay M. Scaling up nutrition: What will it cost?: The World Bank 2009.
55. McGovern ME, Krishna A, Aguayo VM, et al. A review of the evidence linking child stunting to economic outcomes. *International journal of epidemiology* 2017;46(4):1171-91.
56. Sartorius K, Sartorius B, Winkler C, et al. 1. Abstract 2. Introduction 3. miRNA biogenesis, mRNA silencing and export into the extracellular space 3.1. Intracellular mRNA silencing 3.2. Extracellular mRNA silencing.
57. Vaivada T, Gaffey MF, Das JK, et al. Evidence-based interventions for improvement of maternal and child nutrition in low-income settings: what's new? *Current Opinion in Clinical Nutrition & Metabolic Care* 2017;20(3):204-10.
58. Hoddinott J, Alderman H, Behrman JR, et al. The economic rationale for investing in stunting reduction. *Maternal & Child Nutrition* 2013;9:69-82.
59. Lobstein T, Jackson-Leach R, Moodie ML, et al. Child and adolescent obesity: part of a bigger picture. *The lancet* 2015;385(9986):2510-20.
60. UNICEF. Progress for children: a report card on nutrition: Unicef 2006.

For peer review only

Authors' contributions

All authors contributed to the conception and design of the study. BS performed the analysis and initial interpretation of the findings. BS drafted the manuscript. All authors reviewed and provided input to revise the manuscript. All authors gave final approval for submission.

Funding

This study forms part of the Sustainable and Healthy Food Systems (SHEFS) project supported by the Wellcome Trust's Our Planet, Our Health programme (grant number 205200/Z/16/Z). The funders of the study had no role in study design, data collection, data analysis, data interpretation or writing of the report.

Competing interests

None declared.

Patient consent for publication

Not required.

Ethics approval

This study utilised open access data and hence ethical approval was not necessary.

Data availability statement

For peer review only - http://bmjopen.bmj.com/site/about/guidelines.xhtml

566 Data are publically available at https://www.datafirst.uct.ac.za/dataportal/index.php/catalog/NIDS/about

567

For peer review only - http://bmjopen.bmj.com/site/about/guidelines.xhtml

For peer review only

Tables

Table 1: Burden of stunting, thinness/wasting and obesity among children by age and survey round

Survey wave	Age (in years)	N (valid HAZ)	n (stunted)	Prop: Stunted i	Estimated Population stunted	N (valid BMIZ)	n (thin /wasted)	Prop: Thinness ii	Estimated Population thinness	n (obese)	Prop: obese iii	Estimated Population obese
2008	0	220	31	0.14 (0.09, 0.22)	153648 (81545, 273371)	180	21	0.12 (0.07, 0.2)	133882 (66374, 251867)	32	0.1 (0.06, 0.15)	107783 (59737, 185749)
	1	419	29	0.08 (0.05, 0.13)	91903 (48436, 164369)	386	24	0.06 (0.03, 0.11)	66566 (29263, 143661)	76	0.22 (0.16, 0.3)	253021 (159436, 383096)
	2	453	62	0.15 (0.1, 0.21)	159241 (96989, 250626)	419	10	0.03 (0.01, 0.07)	34613 (12484, 87598)	70	0.14 (0.1, 0.19)	148357 (93148, 227510)
	3	489	55	0.11 (0.08, 0.15)	111595 (69906, 172639)	470	19	0.04 (0.02, 0.07)	39715 (20205, 75821)	67	0.17 (0.12, 0.24)	176235 (104092, 284620)
	4	498	48	0.09 (0.06, 0.13)	93391 (54519, 154136)	461	25	0.05 (0.03, 0.08)	52031 (27083, 96623)	34	0.08 (0.05, 0.12)	80282 (45874, 135732)
	**0-5**	**2079**	**225**	**0.11 (0.09, 0.13) iv**	**591550 (451494, 766049)**	**1916**	**99**	**0.05 (0.04, 0.07) iv**	**277743 (196715, 385904)**	**279**	**0.14 (0.12, 0.17) iv**	**778865 (599156, 996439)**
2010/11	0	75	24	0.33 (0.16, 0.57)	289420 (114550, 577181)	69	7	0.1 (0.04, 0.23)	88499 (30258, 228461)	22	0.39 (0.21, 0.61)	340820 (153454, 615984)
	1	236	20	0.06 (0.03, 0.11)	63995 (25204, 132218)	215	11	0.07 (0.03, 0.14)	69776 (25204, 173842)	52	0.29 (0.19, 0.41)	299127 (159624, 499489)
	2	340	61	0.22 (0.16, 0.29)	267019 (166414, 407708)	314	17	0.06 (0.03, 0.11)	76344 (35363, 155183)	72	0.22 (0.16, 0.29)	270818 (167454, 414761)
	3	427	52	0.11 (0.07, 0.16)	130531 (73921, 220389)	402	20	0.03 (0.02, 0.06)	39208 (16427, 85938)	78	0.16 (0.11, 0.23)	195314 (114988, 313258)
	4	422	62	0.17 (0.12, 0.24)	205730 (122130, 329629)	394	19	0.03 (0.02, 0.06)	39494 (17639, 84450)	65	0.17 (0.12, 0.24)	208842 (126152, 329629)
	**0-5**	**1500**	**219**	**0.16 (0.13, 0.19)**	**862302 (633920, 1148376)**	**1394**	**74**	**0.05 (0.03, 0.07)**	**265877 (167080, 405309)**	**289**	**0.21 (0.17, 0.26)**	**1159133 (835398, 1565968)**
2012	0	271	59	0.2 (0.14, 0.28)	181464 (108101, 288795)	250	38	0.2 (0.12, 0.3)	179118 (95658, 311389)	55	0.19 (0.12, 0.28)	169192 (94880, 284482)
	1	544	78	0.13 (0.09, 0.17)	132310 (80796, 207206)	538	27	0.08 (0.05, 0.13)	80862 (40842, 150046)	138	0.23 (0.18, 0.28)	234062 (157153, 334626)
	2	629	72	0.1 (0.07, 0.14)	116230 (68690, 187924)	629	49	0.05 (0.03, 0.07)	55866 (30861, 97391)	147	0.23 (0.18, 0.29)	269508 (176205, 392309)
	3	710	82	0.11 (0.08, 0.16)	142259 (82987, 232297)	692	29	0.03 (0.02, 0.06)	43898 (20928, 87296)	102	0.15 (0.11, 0.2)	191943 (117798, 297399)
	4	771	112	0.16 (0.12, 0.2)	221293 (142258, 330201)	762	30	0.03 (0.02, 0.05)	43556 (20731, 87406)	118	0.18 (0.14, 0.22)	250658 (167278, 362573)
	**0-5**	**2925**	**403**	**0.13 (0.11, 0.16)**	**762303 (567517, 1001855)**	**2871**	**173**	**0.06 (0.05, 0.07)**	**328768 (230074, 458914)**	**560**	**0.19 (0.17, 0.22)**	**1112487 (853832, 1415525)**
2014/15	0	434	74	0.12 (0.08, 0.18)	144201 (81319, 240730)	421	37	0.1 (0.06, 0.18)	123211 (59233, 240730)	78	0.17 (0.12, 0.23)	197209 (117461, 313223)
	1	801	53	0.06 (0.04, 0.08)	67916 (39433, 112566)	801	24	0.03 (0.01, 0.08)	39657 (9858, 101845)	169	0.23 (0.18, 0.28)	266780 (179421, 379240)
	2	785	65	0.08 (0.05, 0.12)	85985 (48668, 146305)	781	16	0.02 (0.01, 0.03)	16222 (6309, 39015)	128	0.16 (0.12, 0.22)	170803 (106348, 263349)
	3	853	82	0.08 (0.06, 0.11)	89857 (54478, 143034)	845	24	0.04 (0.02, 0.07)	40865 (18323, 86890)	79	0.12 (0.08, 0.15)	133857 (83637, 205862)
	4	899	67	0.06 (0.04, 0.09)	77887 (45801, 127320)	897	19	0.02 (0.01, 0.05)	30376 (12301, 71898)	56	0.06 (0.04, 0.11)	82300 (38662, 166265)
	**0-5**	**3772**	**341**	**0.08 (0.06, 0.09)**	**441281 (327611, 581707)**	**3745**	**120**	**0.04 (0.03, 0.05)**	**213012 (130004, 333338)**	**510**	**0.14 (0.12, 0.17)**	**834444 (618820, 1098053)**
2017	0	372	50	0.13 (0.08, 0.19)	125347 (68160, 218303)	357	32	0.12 (0.07, 0.2)	121396 (62270, 221478)	70	0.18 (0.12, 0.25)	174538 (104344, 278066)
	1	760	55	0.08 (0.05, 0.11)	95527 (56435, 153804)	742	23	0.03 (0.02, 0.07)	42416 (17767, 94222)	146	0.23 (0.19, 0.29)	285123 (194388, 403216)
	2	833	63	0.07 (0.05, 0.11)	94807 (54147, 158550)	830	20	0.03 (0.02, 0.07)	43976 (18786, 99279)	130	0.15 (0.12, 0.19)	191812 (127079, 280056)
	3	875	77	0.08 (0.05, 0.12)	99890 (54439, 175689)	872	14	0.02 (0.01, 0.06)	30726 (10888, 79204)	77	0.07 (0.05, 0.1)	88889 (54439, 138247)
	4	900	59	0.05 (0.04, 0.07)	57363 (34849, 91231)	899	23	0.03 (0.01, 0.05)	29923 (13628, 62962)	47	0.06 (0.04, 0.08)	63912 (36990, 105365)
	**0-5**	**3740**	**304**	**0.08 (0.06, 0.09) iv**	**445295 (326192, 593240)**	**3700**	**112**	**0.04 (0.03, 0.05) iv**	**223236 (136790, 345514)**	**470**	**0.13 (0.11, 0.15) iv**	**758650 (583989, 964831)**
**At last observation**	**0-5**	**10711**	**1049**	**0.09 (0.08, 0.10)**	**1 397 020 (1 177 247, 1 616 793)**	**10467**	**391**	**0.04 (0.03, 0.05)**	**560 806 (448 656, 672 957)**	**1,438**	**0.14 (0.13, 0.16)**	**2 048 650 (1 722 242, 2 375 058)**

i: HAZ ≤ -2 SD; ii BMI for age z-score ≤ -2SD; iii BMI for age z-score ≥ +2SD

iv: Significance tests (survey weighted logistic regression) among children 0-5: stunting (2017 vs 2008) p=0.007; thinness/wasting (2017 vs 2008) p=0.131; obesity (2017 vs 2008) p=0.312

For peer review only - http://bmjopen.bmj.com/site/about/guidelines.xhtml

Table 2: Demographic, socio-economic and maternal factors associated with nutritional status among children under 5 years, 2008-2017

Variable	Category	Stunted		p-value	Thin/wasted		p-value	Obese		p-value
		Yes (% col)	No (% col)		Yes (% col)	No (% col)		Yes (% col)	No (% col)	
Ethnicity	African	0.939 [.9027,.9619]	0.871 [.8284,.9039]		0.885 [.8155,.9306]	0.879 [.8383,.9108]		0.931 [.9017,.9522]	0.870 [.8255,.9044]	
	Coloured	0.053 [.0311,.0879]	0.074 [.0486,.1116]		0.076 [.0415,.1344]	0.072 [.0474,.1081]		0.052 [.0333,.0789]	0.076 [.0495,.1152]	
	Asian/Indian	0.003 [4.0e-04,.0202]	0.012 [.0049,.0294]		0.015 [.0026,.077]	0.011 [.0046,.0278]		0.004 [8.4e-04,.0141]	0.013 [.0053,.0317]	
	White	0.006 [.0017,.0179]	0.039 [.0238,.0627]	**<0.001**	0.025 [.0083,.0711]	0.037 [.0229,.0605]	0.823	0.014 [.0066,.0287]	0.041 [.0248,.067]	**<0.001**
Gender	Male	0.562 [.5204,.603]	0.496 [.4797,.5121]		0.514 [.4543,.5742]	0.501 [.4846,.5182]		0.523 [.488,.5575]	0.498 [.481,.5151]	
	Female	0.438 [.397,.4796]	0.504 [.4879,.5203]	**0.002**	0.486 [.4258,.5457]	0.499 [.4818,.5154]	0.686	0.477 [.4425,.512]	0.502 [.4849,.519]	0.178
Birthweight	LBW (<2.5 kgs)	0.148 [.1143,.1891]	0.098 [.0849,.1117]		0.13 [.0891,.1867]	0.098 [.0858,.1111]		0.072 [.0554,.0938]	0.104 [.0919,.118]	
	NBW (≥2.5 kgs)	0.852 [.8109,.8857]	0.903 [.8883,.9151]	**<0.001**	0.87 [.8133,.9109]	0.902 [.8889,.9142]	0.163	0.928 [.9062,.9446]	0.896 [.882,.9081]	**0.006**
	HBW (≥4 kgs)	Not applicable			Not applicable			0.056 [.0419,.0751]	0.04 [.0323,.0496]	
	Non-HBW (<4kgs)							0.944 [.9249,.9581]	0.96 [.9504,.9677]	**0.037**
Income quantile	Lowest	0.294 [.2567,.3334]	0.199 [.1824,.2156]		0.234 [.1805,.2973]	0.203 [.1872,.2195]		0.226 [.1936,.2617]	0.2 [.1834,.2181]	
	Low	0.205 [.1714,.2423]	0.187 [.1714,.2028]		0.214 [.1698,.2656]	0.188 [.173,.2029]		0.203 [.1725,.2377]	0.186 [.1723,.2005]	
	Middle	0.183 [.1555,.2148]	0.200 [.1853,.2154]		0.169 [.1305,.2167]	0.201 [.1871,.2162]		0.18 [.1501,.2135]	0.204 [.1891,.2189]	
	High	0.197 [.1579,.243]	0.186 [.1714,.2021]		0.184 [.1394,.2377]	0.191 [.1751,.2074]		0.182 [.1445,.2269]	0.192 [.1769,.2079]	
	Highest	0.122 [.0924,.1583]	0.229 [.2015,.2585]	**<0.001**	0.2 [.1494,.2612]	0.218 [.1906,.2476]	0.481	0.209 [.1673,.2586]	0.218 [.1915,.2478]	0.422
Low monthly household income	<R2500	0.566 [.5213,.6101]	0.417 [.3929,.4409]		0.488 [.4228,.5544]	0.423 [.3994,.4469]		0.481 [.4406,.5214]	0.416 [.392,.4396]	
	≥R2500	0.434 [.3899,.4787]	0.583 [.5591,.6071]	**<0.001**	0.512 [.4456,.5772]	0.577 [.5531,.6006]	**0.053**	0.519 [.4786,.5594]	0.584 [.5604,.608]	**0.001**
Child hungry in last year (food security) i	Never	0.689 [.595,.7701]	0.697 [.6568,.7346]		0.512 [.3895,.6337]	0.704 [.6643,.7401]		0.707 [.6302,.773]	0.693 [.6522,.7318]	
	Seldom	0.127 [.0669,.2286]	0.096 [.0766,.1193]		0.111 [.056,.2074]	0.097 [.0765,.1219]		0.076 [.0499,.1138]	0.102 [.0787,.13]	
	Sometimes	0.126 [.0807,.1919]	0.155 [.1303,.184]		0.317 [.219,.4354]	0.148 [.1243,.1752]		0.154 [.0994,.231]	0.155 [.1316,.1822]	
	Often	0.054 [.0265,.1049]	0.043 [.0276,.0653]		0.052 [.0222,.1181]	0.042 [.0272,.0655]		0.052 [.0272,.0981]	0.041 [.0269,.0621]	
	Always	0.004 [.0011,.0144]	0.009 [.0048,.0173]	0.505	0.007 [.001,.0504]	0.009 [.0049,.0171]	**<0.001**	0.011 [.0039,.0313]	0.009 [.0048,.016]	0.645
Province	Eastern Cape	0.165 [.1137,.2336]	0.132 [.0978,.1765]		0.075 [.0492,.1137]	0.137 [.1007,.1838]		0.19 [.1321,.2643]	0.124 [.0916,.1652]	
	Free State	0.066 [.0441,.0961]	0.050 [.036,.0678]		0.032 [.0169,.0611]	0.052 [.0376,.0709]		0.045 [.0298,.068]	0.052 [.0379,.071]	
	Gauteng	0.188 [.132,.2606]	0.236 [.1819,.2996]		0.298 [.1952,.4272]	0.231 [.1784,.2937]		0.173 [.1234,.2365]	0.246 [.1891,.3128]	
	KwaZulu-Natal	0.218 [.1619,.2857]	0.227 [.1801,.2819]		0.161 [.1151,.2195]	0.228 [.1804,.2835]		0.293 [.217,.3834]	0.212 [.1691,.262]	
	Limpopo	0.143 [.0947,.2088]	0.109 [.0816,.1444]		0.129 [.0823,.195]	0.113 [.0842,.1491]		0.074 [.0514,.105]	0.121 [.0902,.1599]	
	Mpumalanga	0.085 [.0541,.1318]	0.083 [.0621,.1102]		0.096 [.0611,.1487]	0.082 [.0611,.1098]		0.074 [.0506,.1079]	0.085 [.0626,.1131]	
	North West	0.055 [.0355,.0833]	0.05 [.035,.0709]		0.06 [.0376,.0943]	0.05 [.0346,.0712]		0.038 [.0252,.056]	0.053 [.0362,.076]	
	Northern Cape	0.022 [.0141,.0333]	0.023 [.0163,.031]		0.033 [.0217,.0489]	0.022 [.0159,.0303]		0.011 [.0072,.0156]	0.025 [.0178,.0341]	
	Western Cape	0.06 [.0321,.1089]	0.091 [.0606,.134]	**0.012**	0.116 [.0638,.2016]	0.086 [.0572,.1262]	**0.002**	0.103 [.0626,.1641]	0.084 [.0554,.1254]	**<0.001**
Environment	Rural/Tribal authority	0.519 [.4417,.5963]	0.451 [.3933,.5091]	**<0.001**	0.429 [.3428,.5201]	0.46 [.4021,.5193]	0.647	0.466 [.3857,.5479]	0.457 [.4002,.5158]	0.111

For peer review only

For peer review only - http://bmjopen.bmj.com/site/about/guidelines.xhtml

	Urban Informal	0.122 [.0737,.1943]	0.101 [.0628,.1592]		0.1 [.0557,.1743]	0.102 [.0636,.161]		0.133 [.0691,.239]	0.097 [.0618,.148]	
	Urban Formal	0.359 [.292,.4319]	0.448 [.389,.509]		0.47 [.3734,.5696]	0.437 [.3787,.4979]		0.402 [.3261,.4821]	0.446 [.3868,.5066]	
	Underweight	0.041 [.0271,.0604]	0.022 [.0178,.0282]		0.051 [.0298,.0867]	0.023 [.018,.0281]		0.019 [.01,.0351]	0.025 [.0198,.0311]	
	Normal	0.397 [.3521,.444]	0.344 [.3213,.3683]		0.418 [.3455,.4946]	0.348 [.3251,.3724]		0.327 [.2853,.3708]	0.356 [.332,.3815]	
	Overweight	0.268 [.2311,.3092]	0.273 [.2565,.289]		0.249 [.199,.3064]	0.272 [.2565,.2881]		0.26 [.23,.2922]	0.273 [.2567,.2899]	
Mother BMI	Obese	0.294 [.2452,.3479]	0.361 [.3342,.3882]	**0.003**	0.282 [.2137,.3615]	0.357 [.3298,.3853]	**0.005**	0.395 [.3514,.4396]	0.346 [.3175,.3753]	0.135
	<20	0.073 [.0562,.0947]	0.048 [.0419,.0555]		0.112 [.0574,.206]	0.047 [.041,.0532]		0.057 [.0456,.0701]	0.049 [.0418,.0562]	
	20-24	0.219 [.1852,.2571]	0.230 [.2152,.2459]		0.258 [.201,.3252]	0.23 [.2138,.2461]		0.265 [.2272,.3069]	0.224 [.2091,.2405]	
	25-34	0.468 [.4191,.5175]	0.491 [.4705,.5107]		0.398 [.3311,.4691]	0.492 [.4713,.5118]		0.472 [.425,.5189]	0.49 [.4691,.511]	
	35-44	0.215 [.1731,.2638]	0.210 [.191,.2297]		0.213 [.1682,.2667]	0.211 [.1923,.2301]		0.191 [.1536,.236]	0.214 [.1952,.2348]	
Mother age	45+	0.025 [.0161,.0381]	0.021 [.0177,.0256]	0. 156	0.019 [.0089,.0388]	0.022 [.018,.0261]	**0.007**	0.015 [.0095,.024]	0.023 [.0186,.0278]	0.121
	None	0.023 [.0136,.0397]	0.018 [.0144,.0226]		0.025 [.0127,.0479]	0.019 [.0148,.0239]		0.025 [.0157,.0406]	0.018 [.014,.023]	
	Primary	0.121 [.0921,.1576]	0.072 [.0625,.0835]		0.132 [.095,.1804]	0.071 [.061,.0825]		0.067 [.0488,.0925]	0.075 [.0647,.0869]	
	Secondary	0.799 [.7529,.8385]	0.796 [.7777,.8134]		0.715 [.6506,.7712]	0.802 [.7832,.8203]		0.803 [.7595,.8398]	0.798 [.7785,.8152]	
Mother education	Tertiary	0.057 [.0364,.0868]	0.114 [.0985,.1307]	**<0.001**	0.129 [.0862,.1874]	0.108 [.0925,.1251]	**0.001**	0.105 [.077,.1405]	0.11 [.0938,.1275]	0.568
	None	0.003 [8.0e-04,.0082]	0.003 [.0017,.0051]		0.005 [6.7e-04,.0333]	0.003 [.0017,.0051]		0.002 [6.8e-04,.0053]	0.003 [.0018,.0057]	
	Primary	0.646 [.5533,.7282]	0.56 [.5162,.6028]		0.565 [.4542,.6703]	0.556 [.5118,.5984]		0.584 [.499,.6637]	0.551 [.505,.5971]	
	Secondary	0.275 [.2008,.3629]	0.389 [.3468,.4334]		0.382 [.2783,.4965]	0.387 [.3448,.431]		0.318 [.2475,.3976]	0.398 [.3529,.445]	
Father education	Tertiary	0.077 [.0413,.1403]	0.048 [.035,.0651]	**0.020**	0.048 [.0206,.1099]	0.055 [.0389,.0761]	0.960	0.097 [.0502,.1779]	0.047 [.0338,.0658]	**0.033**

i: only included in wave 1 questionnaire

For peer review only

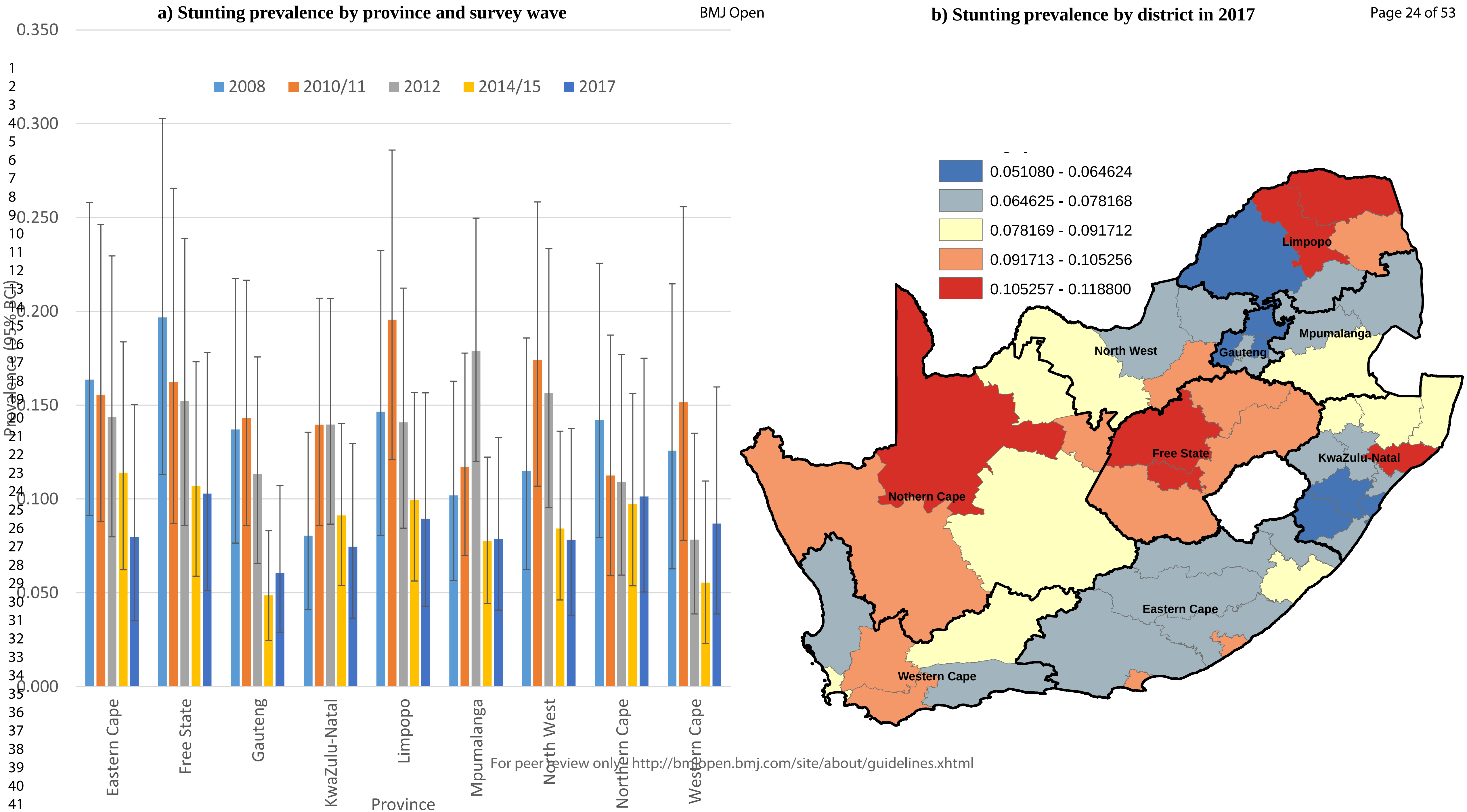
a) Stunting prevalence by province and survey wave
2008
2010/11
2012
2014/15
2017
0.350
0.300
0.250
0.200
0.150
0.100
0.050
0.000
Prevalence (95% BCI)
Eastern Cape
Free State
Gauteng
KwaZulu-Natal
Limpopo
Mpumalanga
North West
Northern Cape
Western Cape
Province
b) Stunting prevalence by district in 2017
0.051080 - 0.064624
0.064625 - 0.078168
0.078169 - 0.091712
0.091713 - 0.105256
0.105257 - 0.118800
Limpopo
Mpumalanga
Gauteng
North West
Free State
KwaZulu-Natal
Nothern Cape
Eastern Cape
Western Cape

a) Thinness/wasting prevalence by province and survey wave

b) Thinness/wasting prevalence by district in 2017

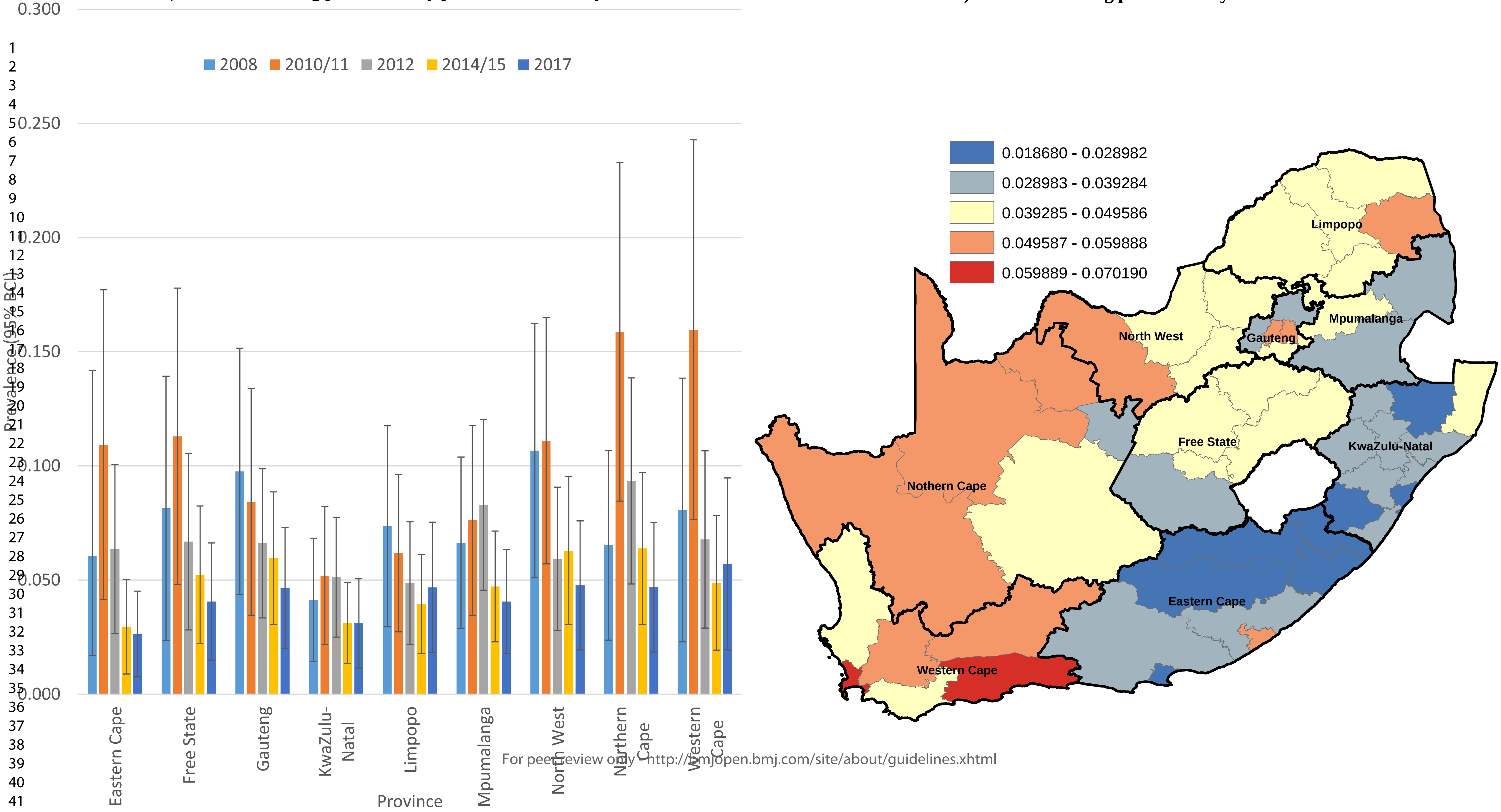

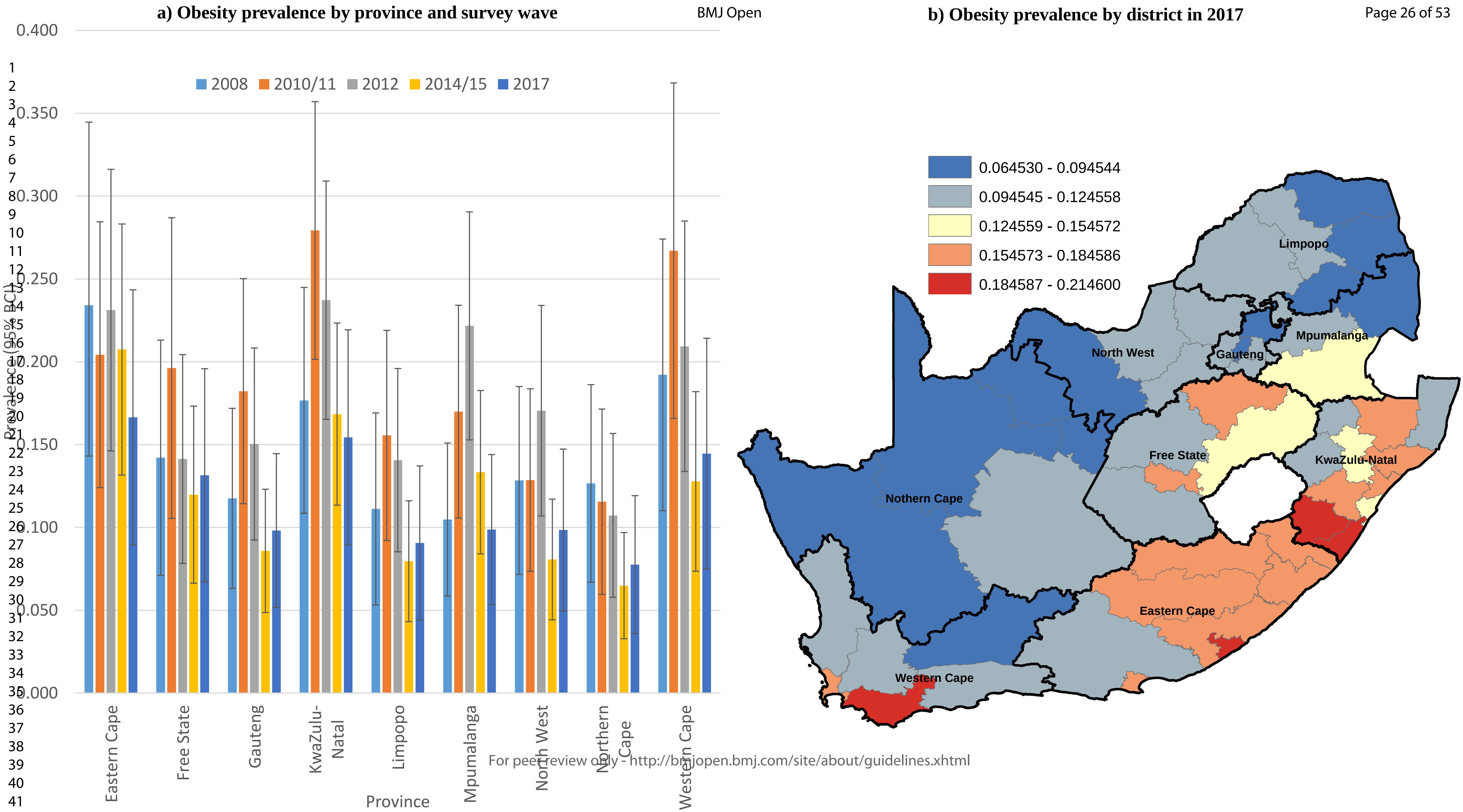

For peer review only - http://bmjopen.bmj.com/site/about/guidelines.xhtml

Supplementary Material

Supplementary 1: Spatial autocorrelation analyses for the 3 anthropometric outcomes (univariate and bivariate)

Pairwise correlation for anthropometric outcomes and bivariate spatial autocorrelation

We have performed additional supplementary analyses (suing GeoDa: Anselin L, Syabri I, Kho Y. GeoDa: an introduction to spatial data analysis. Geographical analysis. 2006 Jan;38(1):5-22) which assesses pairwise correlation/association between the 3 outcomes as well as bivariate Moran's I to assess if there was significant spatial autocorrelation between the outcomes. This analysis suggests that there is no significant association between stunting and thinness/wasting while there is weak positive but significant spatial autocorrelation between stunting and obesity prevalence as well as weak negative spatial correlation between thinness and obesity (please see detailed analyses below).

```
. spearman stunted_svy thin_svy

 Number of obs =      256
Spearman's rho =       0.0729

Test of Ho: stunted_svy and thin_svy are independent
    Prob > |t| =       0.2452

. gllamm stunted_svy thin_svy, i(id)

number of level 1 units = 256
number of level 2 units = 52

Condition Number = 14.594452

gllamm model

log likelihood = 283.93295

------------------------------------------------------------------------------
 stunted_svy |      Coef.   Std. Err.      z    P>|z|     [95% Conf. Interval]
-------------+----------------------------------------------------------------
    thin_svy |   .0385636   .0726234     0.53   0.595    -.1037757    .1809028
       _cons |   .1082981   .0061531    17.60   0.000     .0962381     .120358
------------------------------------------------------------------------------

Variance at level 1
------------------------------------------------------------------------------

  .00637033 (.00056306)

Variances and covariances of random effects
------------------------------------------------------------------------------

***level 2 (id)

    var(1): 2.643e-24 (5.133e-14)
------------------------------------------------------------------------------
```

For peer review only

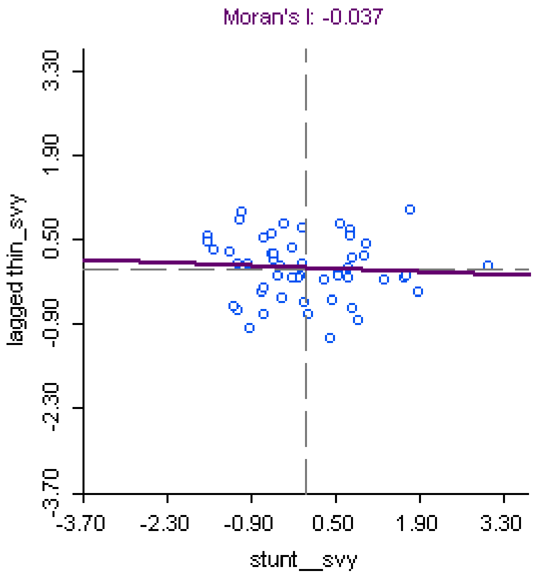

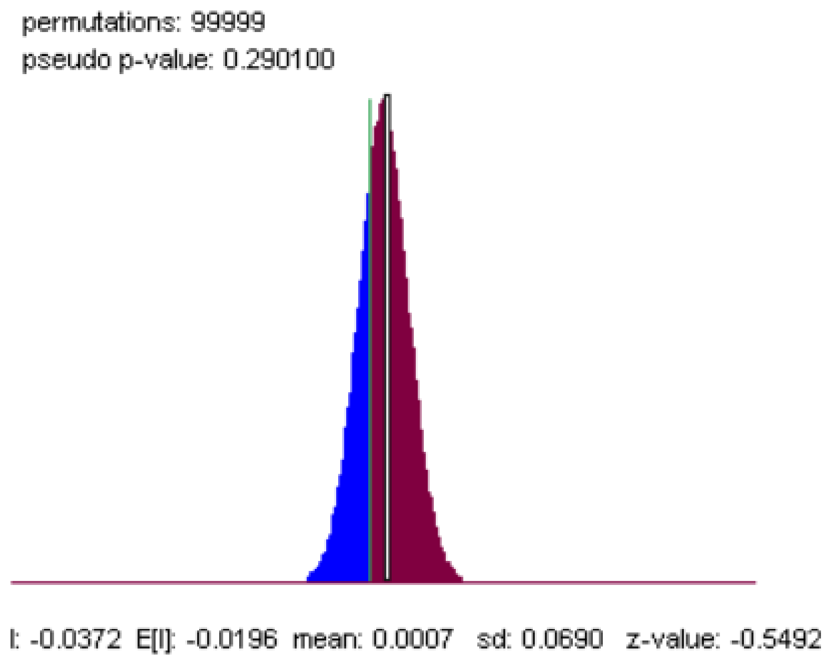


```
. spearman stunted_svy obese_svy

 Number of obs =     256
Spearman's rho =       0.2051

Test of Ho: stunted_svy and obese_svy are independent
    Prob > |t| =       0.0010

. gllamm stunted_svy obese_svy , i(id)

number of level 1 units = 256
number of level 2 units = 52

Condition Number = 10.565877

gllamm model

log likelihood = 292.58012

------------------------------------------------------------------------------
 stunted_svy |      Coef.   Std. Err.      z    P>|z|     [95% Conf. Interval]
-------------+----------------------------------------------------------------
```

```
    obese_svy |   .1980684   .0475478     4.17   0.000     .1048765    .2912604
        _cons |   .0791266   .0090305     8.76   0.000     .0614272    .0968261
------------------------------------------------------------------------------

Variance at level 1
------------------------------------------------------------------------------

  .00580379 (.00057983)

Variances and covariances of random effects
------------------------------------------------------------------------------

***level 2 (id)

    var(1): .00015837 (.00029997)
------------------------------------------------------------------------------
```

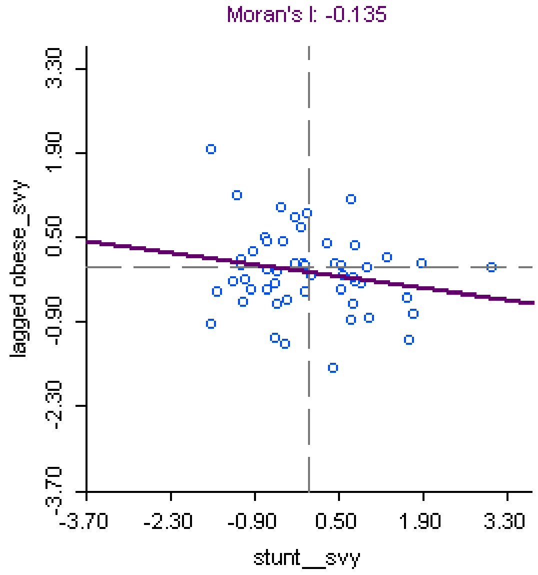

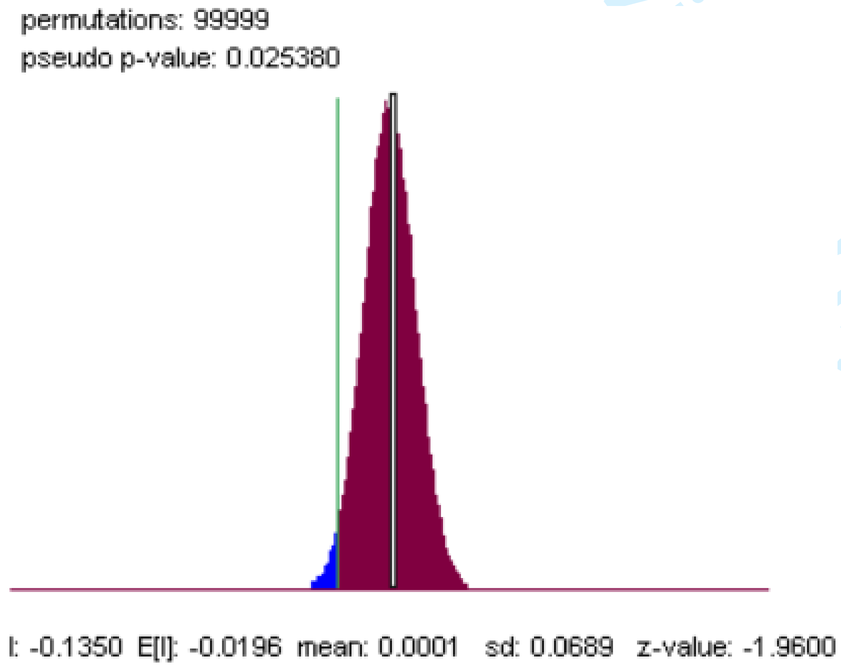


```
. spearman thin_svy obese_svy

 Number of obs =     256
Spearman's rho =      -0.1424

Test of Ho: thin_svy and obese_svy are independent
```

```
    Prob > |t| =       0.0227

. gllamm thin_svy obese_svy , i(id)

number of level 1 units = 256
number of level 2 units = 52

Condition Number = 10.976401

gllamm model

log likelihood = 324.36079

------------------------------------------------------------------------------
    thin_svy |      Coef.   Std. Err.      z    P>|z|     [95% Conf. Interval]
-------------+----------------------------------------------------------------
   obese_svy |   -.067802    .040258    -1.68   0.092    -.1467062    .0111022
       _cons |   .0602269   .0078037     7.72   0.000     .0449319    .0755218
------------------------------------------------------------------------------

Variance at level 1
------------------------------------------------------------------------------

  .00447574 (.00044278)

Variances and covariances of random effects
------------------------------------------------------------------------------

***level 2 (id)

    var(1): .00018259 (.00023176)
------------------------------------------------------------------------------
```

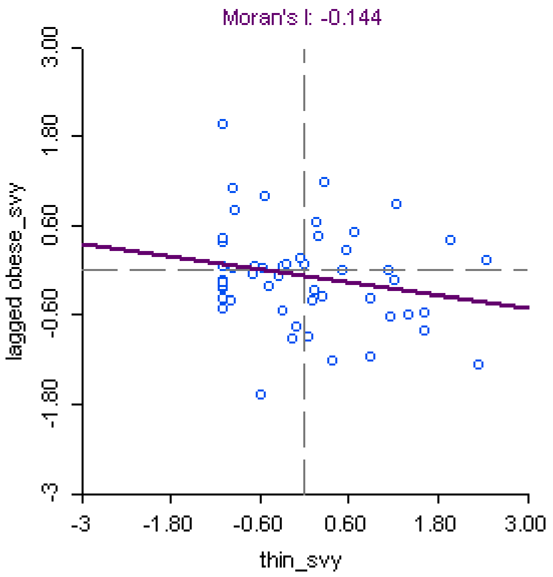

For peer review only

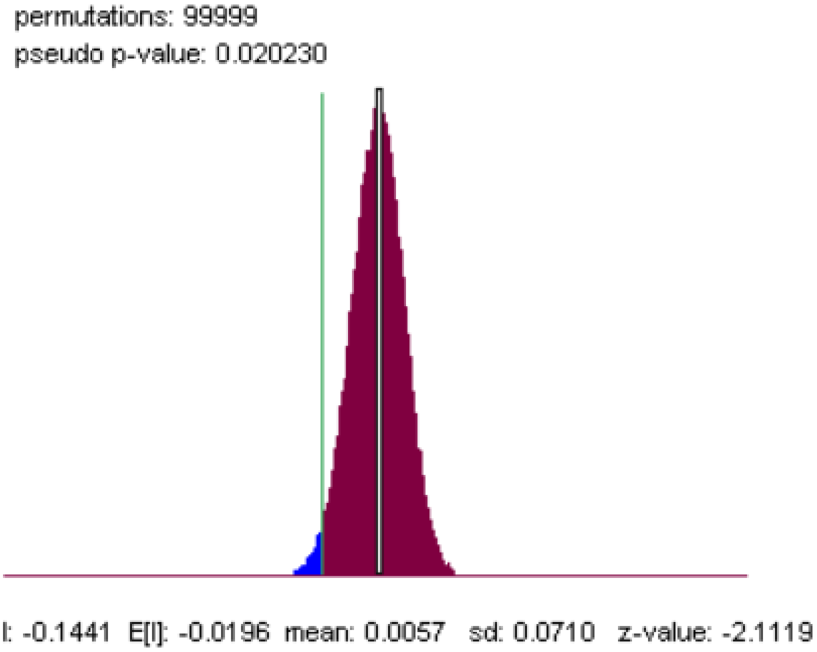

With regards to the shared temporal effect this we think can be retained as all 3 outcomes appear to have a negative coefficient associated with increasing panel or wave.

```
. gllamm stunted_svy year , i(id)

number of level 1 units = 256
number of level 2 units = 52

Condition Number = 31.724715

gllamm model

log likelihood = 293.64743

------------------------------------------------------------------------------
 stunted_svy |      Coef.   Std. Err.      z    P>|z|     [95% Conf. Interval]
-------------+----------------------------------------------------------------
        year |  -.0153423   .0033894    -4.53   0.000    -.0219855   -.0086992
       _cons |   .1563577   .0112694    13.87   0.000     .1342702    .1784453
------------------------------------------------------------------------------

Variance at level 1
------------------------------------------------------------------------------

  .00590475 (.00052191)

Variances and covariances of random effects
------------------------------------------------------------------------------


***level 2 (id)

    var(1): 8.887e-19 (4.854e-11)
------------------------------------------------------------------------------

. gllamm thin_svy year , i(id)

number of level 1 units = 256
number of level 2 units = 52

Condition Number = 37.175479

gllamm model

log likelihood = 327.11892

------------------------------------------------------------------------------
```

For peer review only

```
    thin_svy |      Coef.   Std. Err.      z    P>|z|     [95% Conf. Interval]
-------------+----------------------------------------------------------------
        year |  -.0084373   .0028941    -2.92   0.004    -.0141096   -.002765
       _cons |   .0749857   .0098979     7.58   0.000     .0555862    .0943852
------------------------------------------------------------------------------

Variance at level 1
------------------------------------------------------------------------------

  .00430301 (.00042507)

Variances and covariances of random effects
------------------------------------------------------------------------------


***level 2 (id)

    var(1): .00027197 (.0002388)
------------------------------------------------------------------------------

. gllamm obese_svy year , i(id)

number of level 1 units = 256
number of level 2 units = 52

Condition Number = 21.597249

gllamm model

log likelihood = 215.4003

------------------------------------------------------------------------------
   obese_svy |      Coef.   Std. Err.      z    P>|z|     [95% Conf. Interval]
-------------+----------------------------------------------------------------
        year |  -.0112194   .0043125    -2.60   0.009    -.0196717   -.0027671
       _cons |   .1905201   .0155017    12.29   0.000     .1601374    .2209029
------------------------------------------------------------------------------

Variance at level 1
------------------------------------------------------------------------------

  .00954712 (.00094327)

Variances and covariances of random effects
------------------------------------------------------------------------------

***level 2 (id)

    var(1): .00175973 (.00074487)
------------------------------------------------------------------------------
```

Univariate spatial autocorrelation

Based on the univariate Moran's I statistics for each anthropometric outcome there appeared to be significant spatial heterogeneity present for all 3 outcomes.

For peer review only

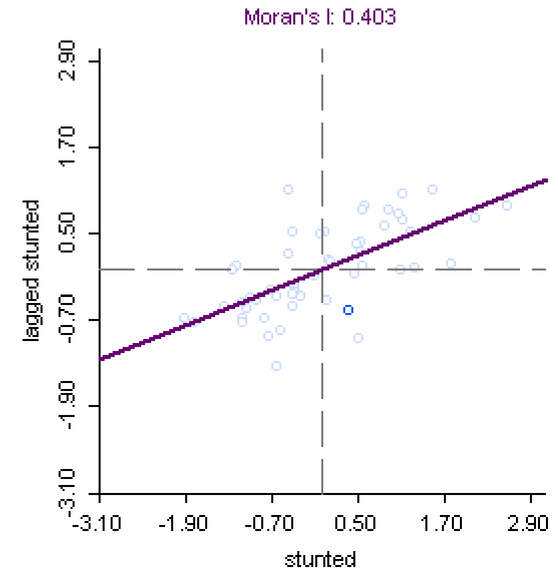
Moran's I: 0.403
lagged stunted
2.90
1.70
0.50
-0.70
-1.90
-3.10
-3.10
-1.90
-0.70
0.50
1.70
2.90
stunted

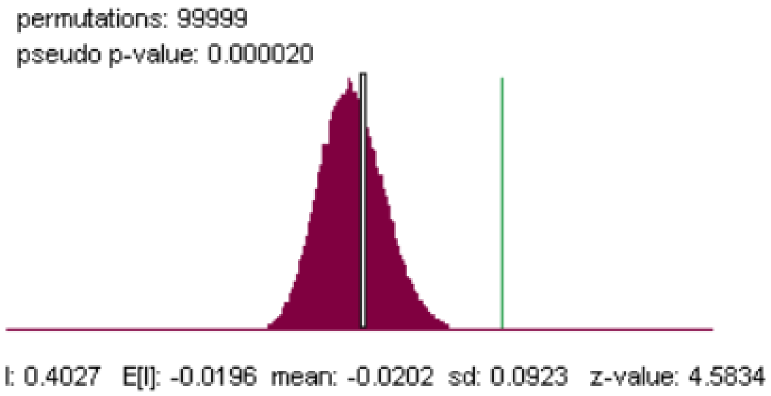
permutations: 99999
pseudo p-value: 0.000020
I: 0.4027 E[I]: -0.0196 mean: -0.0202 sd: 0.0923 z-value: 4.5834

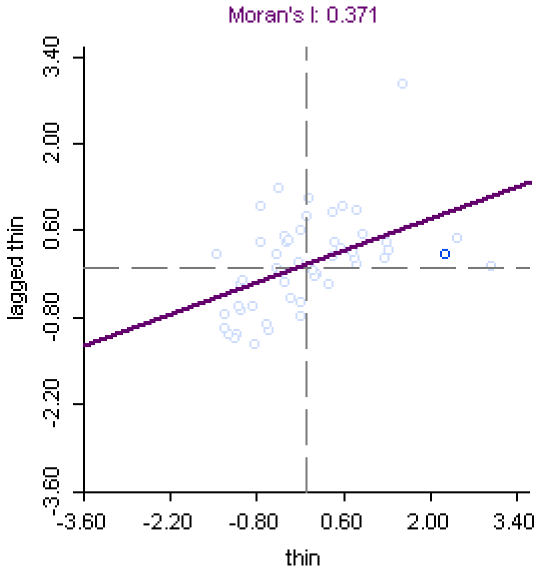
Moran's I: 0.371
lagged thin
3.40
2.00
0.60
-0.80
-2.20
-3.60
-3.60
-2.20
-0.80
0.60
2.00
3.40
thin

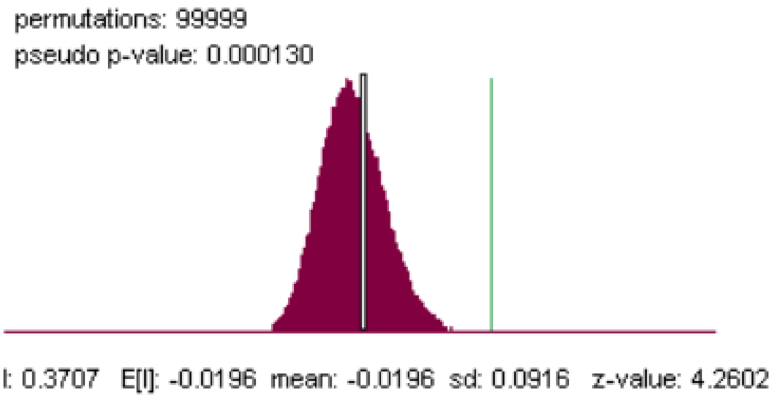

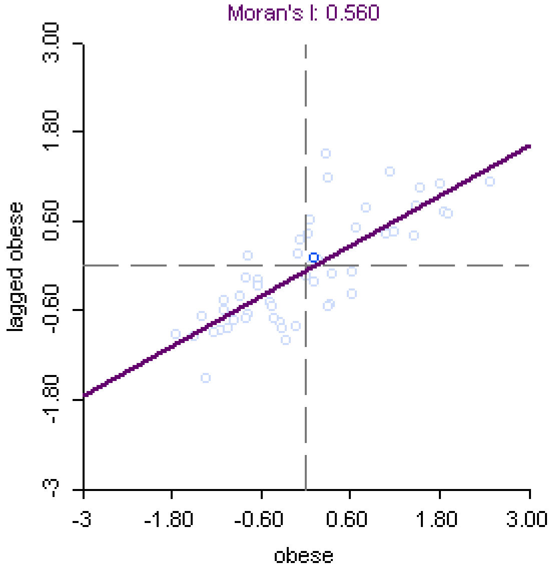

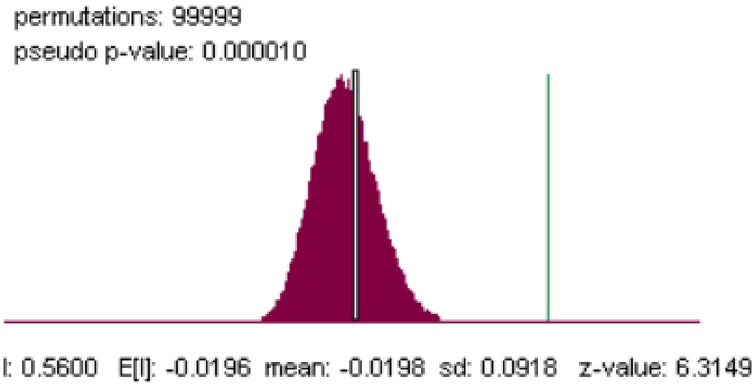

Supplementary 2: Win BUGS code for Bayesian space-time binomial model

```
model {
        for (i in 1:N ) {
                for (j in 1:T ) {
                #Likelihood

                stunted[j,i] ~ dbin(p1[j,i],child[j,i])
                logit(p1[j,i])<-mediainter1+inter1[j]+theta.ST1[j,i]
```

```
                thin[j,i] ~ dbin(p2[j,i],child[j,i])
                logit(p2[j,i])<-mediainter2+inter2[j]+theta.ST2[j,i]

                obese[j,i] ~ dbin(p3[j,i],child[j,i])
                logit(p3[j,i])<-mediainter3+inter3[j]+theta.ST3[j,i]
                }
        }

#Spatio-temporal effect for the first wave
  theta.S1[1,1:N]~car.normal(adj[],weights[],num[],prec.spat[1])
  theta.S2[1,1:N]~car.normal(adj[],weights[],num[],prec.spat[2])
  theta.S3[1,1:N]~car.normal(adj[],weights[],num[],prec.spat[3])

  for(i in 1:N){
        theta.ST1[1,i]<-pow(1-ro1*ro1,-0.5)*theta.S1[1,i]
        theta.ST2[1,i]<-pow(1-ro2*ro2,-0.5)*theta.S2[1,i]
        theta.ST3[1,i]<-pow(1-ro3*ro3,-0.5)*theta.S3[1,i]
        }

#Spatio-temporal effect for the subsequent waves
  for(j in 2:T){
    for(i in 1:N){
                theta.ST1[j,i]<-ro1*theta.ST1[j-1,i]+theta.S1[j,i]
                theta.ST2[j,i]<-ro2*theta.ST2[j-1,i]+theta.S2[j,i]
                theta.ST3[j,i]<-ro3*theta.ST3[j-1,i]+theta.S3[j,i]
                }
        theta.S1[j,1:N]~car.normal(adj[],weights[],num[],prec.spat[1])
        theta.S2[j,1:N]~car.normal(adj[],weights[],num[],prec.spat[1])
        theta.S3[j,1:N]~car.normal(adj[],weights[],num[],prec.spat[1])
        }

#Weights for CAR
for(k in 1:240) {
        weights[k]<-1
        }

#Prior distributions for the global time trends

inter1[1:T]~car.normal(adj.t[],weights.t[],num.t[],prec.inter[1])
inter2[1:T]~car.normal(adj.t[],weights.t[],num.t[],prec.inter[2])
inter3[1:T]~car.normal(adj.t[],weights.t[],num.t[],prec.inter[3])

for (t in 1:1) {
        weights.t[t] <- 1;
        adj.t[t] <- t+1;
        num.t[t] <- 1
        }
for (t in 2:(T-1)) {
        weights.t[2+(t-2)*2] <- 1;
        adj.t[2+(t-2)*2] <- t-1
        weights.t[3+(t-2)*2] <- 1;
        adj.t[3+(t-2)*2] <- t+1;
        num.t[t] <- 2
        }
for (t in T:T) {
        weights.t[(T-2)*2 + 2] <- 1;
        adj.t[(T-2)*2 + 2] <- t-1;
        num.t[t] <- 1
        }
```

```
#Prior distributions for the precision parameters in the model
for(i in 1:3){
            prec.spat[i]~dgamma(0.5, 0.005)
            prec.inter[i]~dgamma(0.5, 0.005)
            }

#Prior distributions for the mean risk for each outcome for every district and period
mediainter1~dflat()
mediainter2~dflat()
mediainter3~dflat()

#Prior distributions for the temporal dependence parameters for each outcome
    ro1~dunif(-1,1)
    ro2~dunif(-1,1)
    ro3~dunif(-1,1)

}
}
```

Supplementary 3: Model convergence

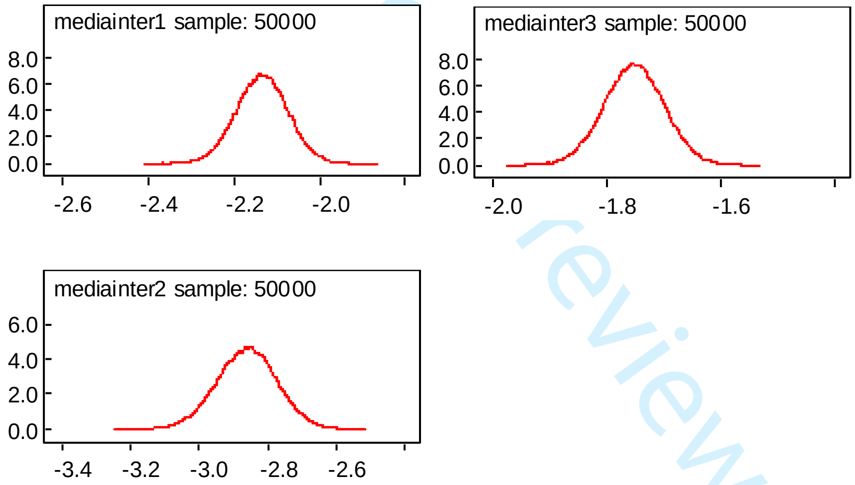

For peer review only

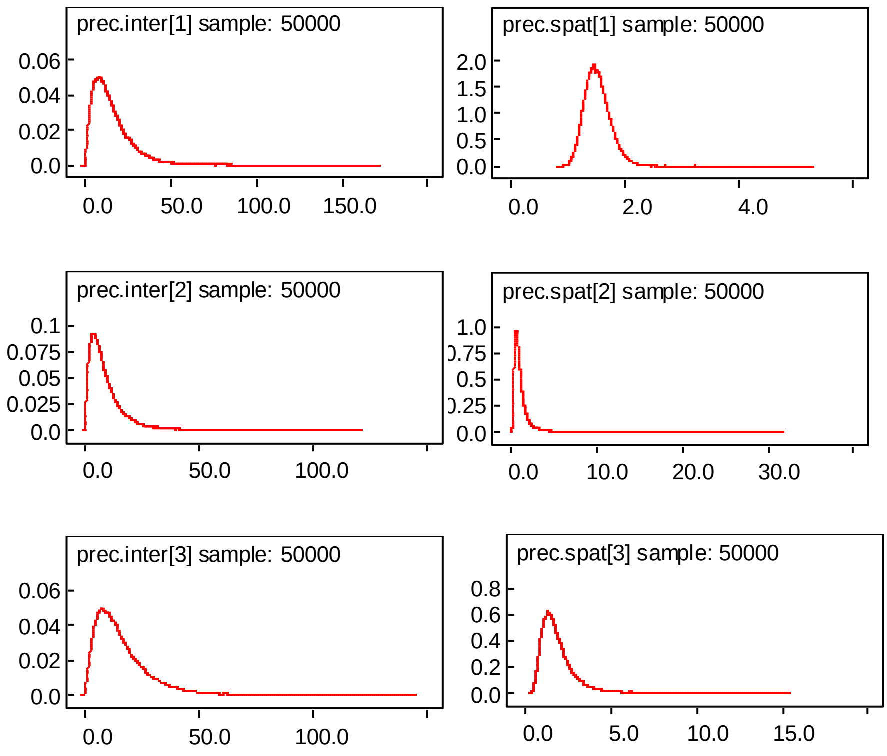
prec.inter[1] sample: 50000
prec.spat[1] sample: 50000
prec.inter[2] sample: 50000
prec.spat[2] sample: 50000
prec.inter[3] sample: 50000
prec.spat[3] sample: 50000

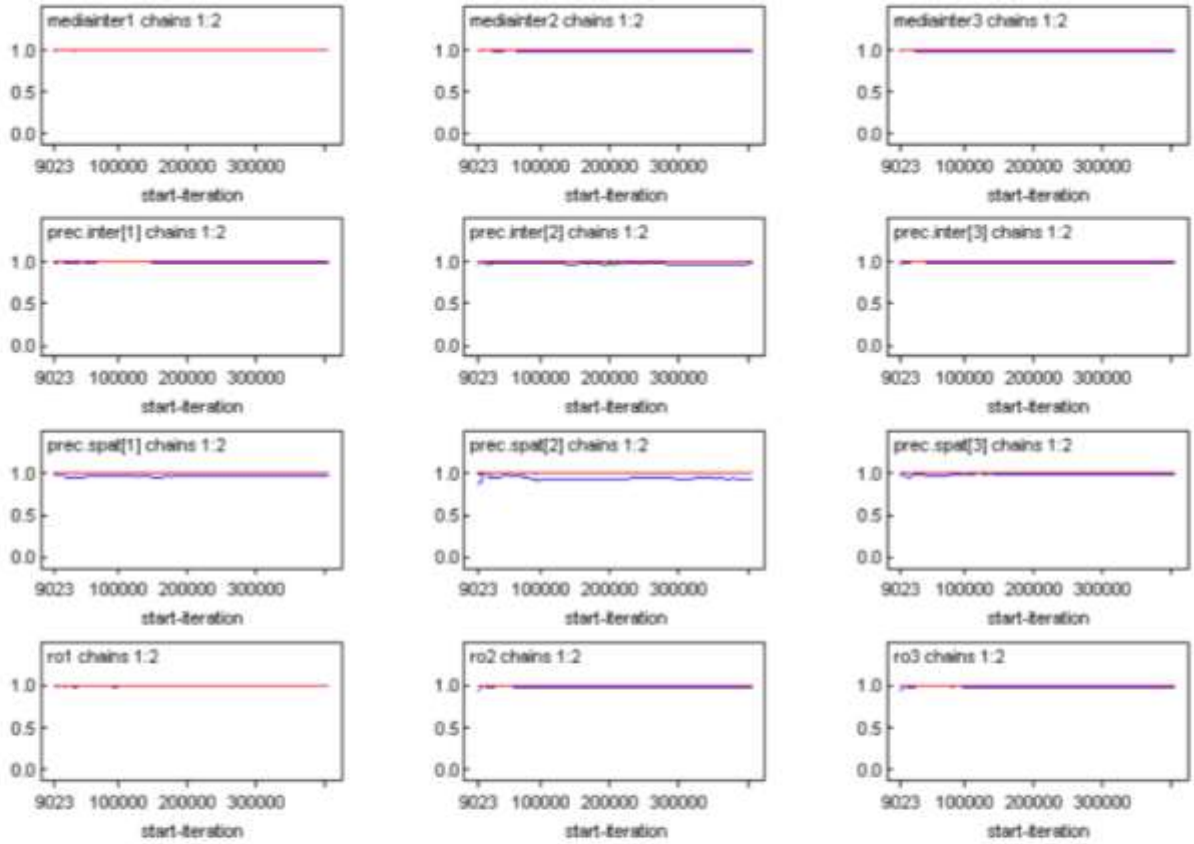
mediainter1 chains 1:2
mediainter2 chains 1:2
mediainter3 chains 1:2
prec.inter[1] chains 1:2
prec.inter[2] chains 1:2
prec.inter[3] chains 1:2
prec.spat[1] chains 1:2
prec.spat[2] chains 1:2
prec.spat[3] chains 1:2
ro1 chains 1:2
ro2 chains 1:2
ro3 chains 1:2
start-iteration

Supplementary 4: Model fit and out of sample validation

Overall model fit

Dbar = post.mean of -2logL; Dhat = -2LogL at post.mean of stochastic nodes

	Dbar	Dhat	pD	DIC
obese	1140.710	1008.690	132.013	1272.720
stunted	1053.560	936.365	117.194	1170.750
thin	719.774	636.974	82.800	802.574
total	2914.040	2582.030	332.007	3246.050

Comparison of survey prevalence versus model fitted prevalence by anthropometric measure

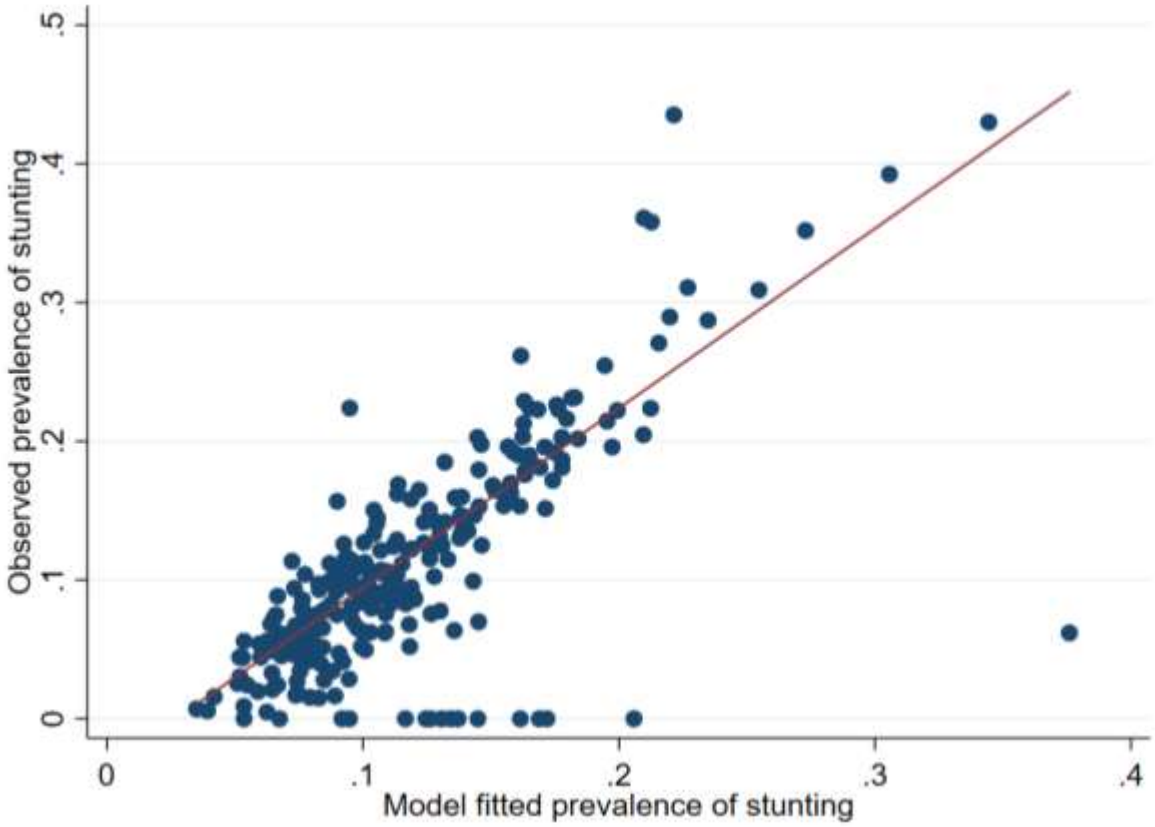


```
. spearman stunted_svy stunting_post if stunted_svy~=0

 Number of obs =     241
Spearman's rho =       0.8601

Test of Ho: stunted_svy and stunting_post are independent
    Prob > |t| =       0.0000

. spearman stunted_svy stunting_post

 Number of obs =     256
Spearman's rho =       0.7287

Test of Ho: stunted_svy and stunting_post are independent
    Prob > |t| =       0.0000
```

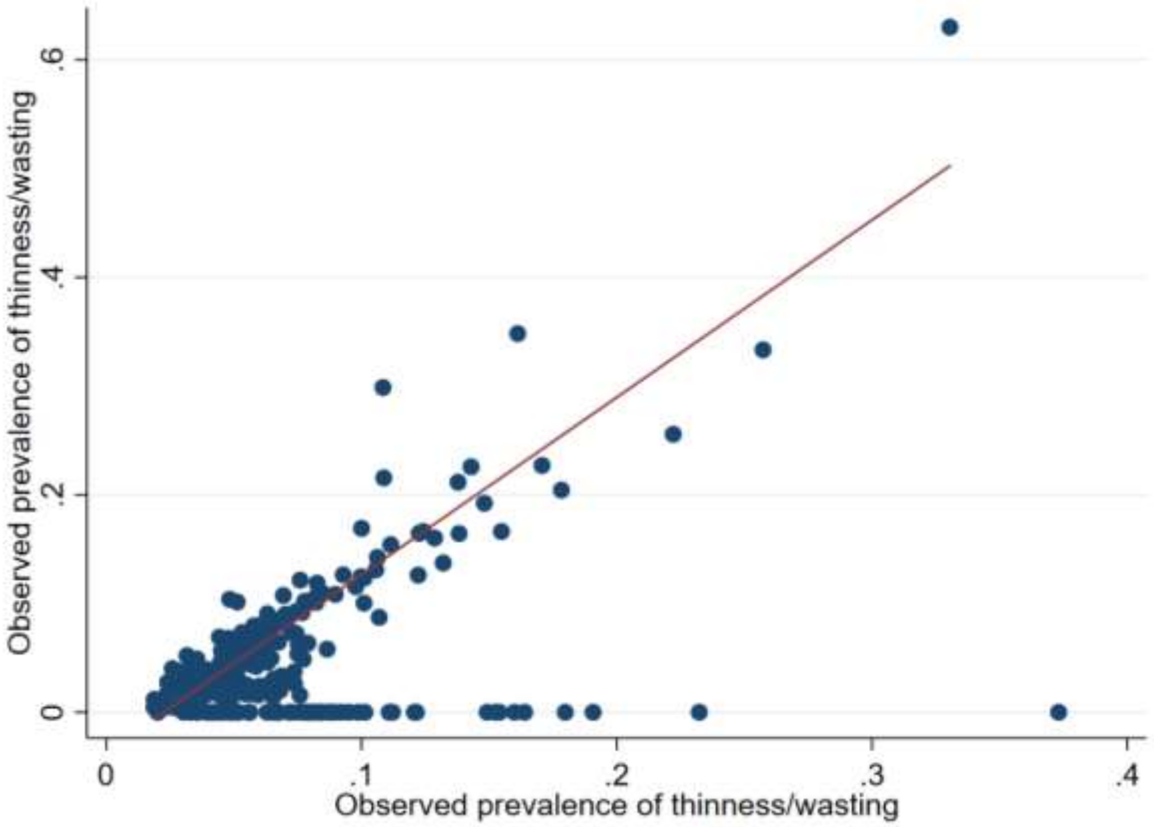


```
. spearman thin_svy thin_post if thin_svy~=0

 Number of obs =     191
Spearman's rho =       0.8047

Test of Ho: thin_svy and thin_post are independent
    Prob > |t| =       0.0000

. spearman thin_svy thin_post

 Number of obs =     256
Spearman's rho =       0.2857

Test of Ho: thin_svy and thin_post are independent
    Prob > |t| =       0.0000
```

review only

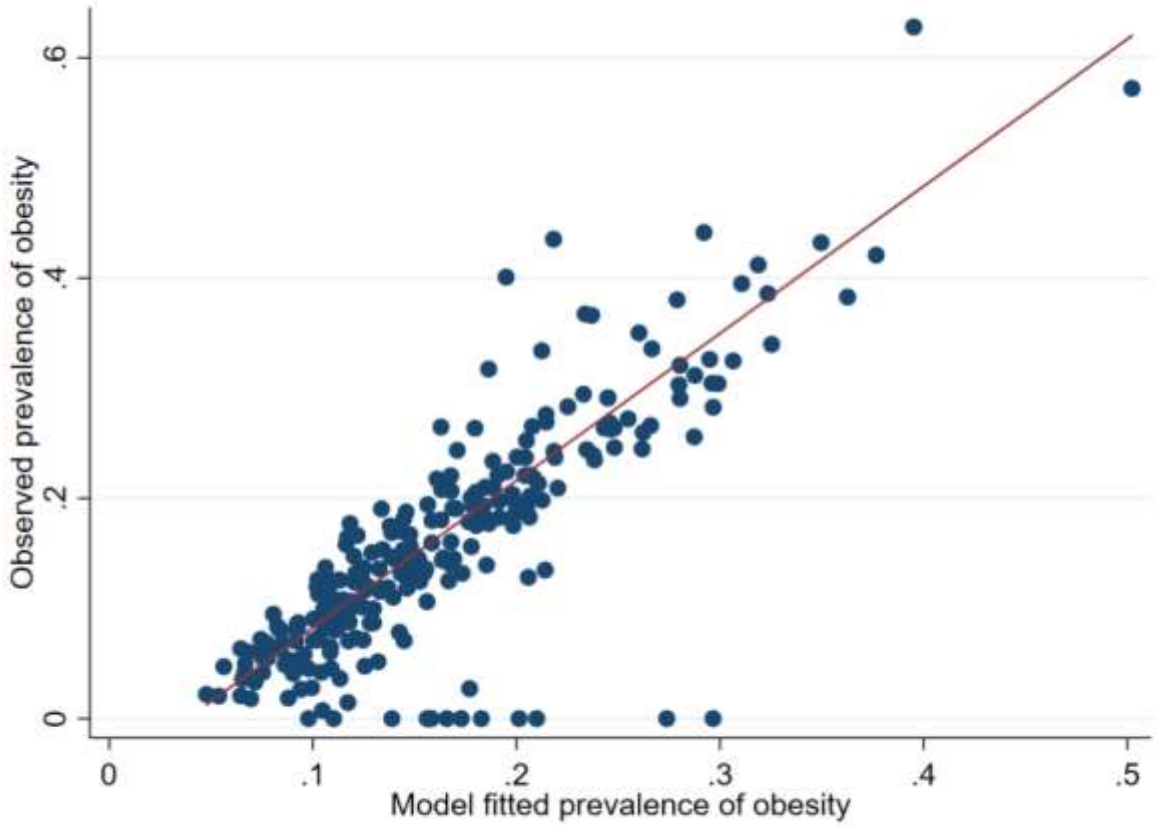


```
. spearman obese_svy obesity_post if obese_svy~=0

 Number of obs =     243
Spearman's rho =       0.9140

Test of Ho: obese_svy and obesity_post are independent
    Prob > |t| =       0.0000

. spearman obese_svy obesity_post

 Number of obs =     256
Spearman's rho =       0.8002

Test of Ho: obese_svy and obesity_post are independent
    Prob > |t| =       0.0000
```

Out of sample validation/prediction (10% random sample)

Of the 37 out of sample validation points, 28 of the observed prevalence of stunting where within the 95% uncertainty interval for the predicted posterior prevalence, 25/37 for thin/wasted and 30/37 for obesity.

```
. spearman stunted_svy stuntedvpost if validation_sample2==1

 Number of obs =      37
Spearman's rho =       0.4445

Test of Ho: stunted_svy and stuntedvpost are independent
    Prob > |t| =       0.0058
```

review only

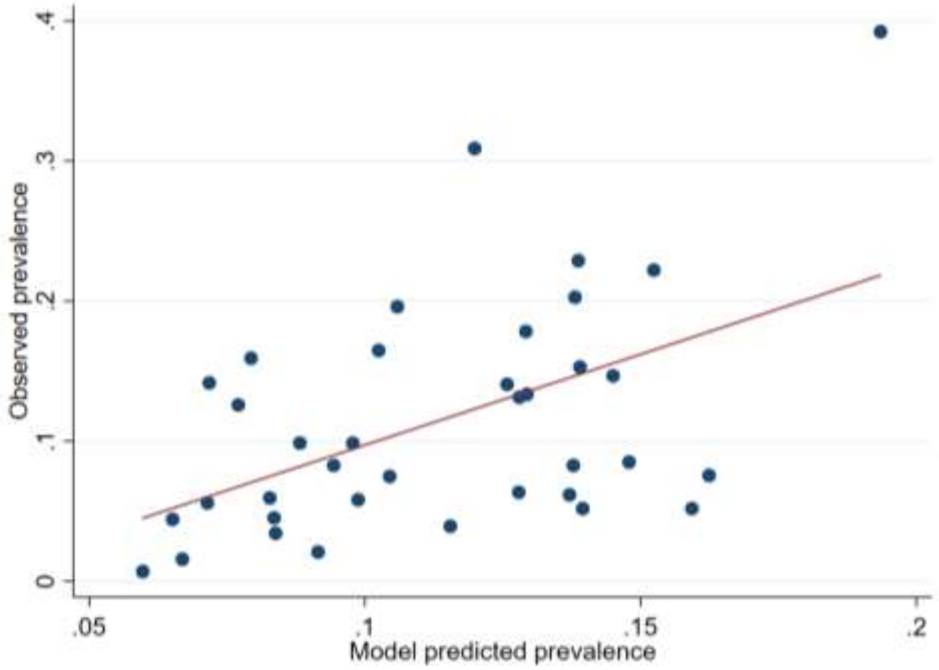


```
. spearman thin_svy thinvpost if validation_sample2==1

 Number of obs =      37
Spearman's rho =       0.2048

Test of Ho: thin_svy and thinvpost are independent
    Prob > |t| =       0.2239
```

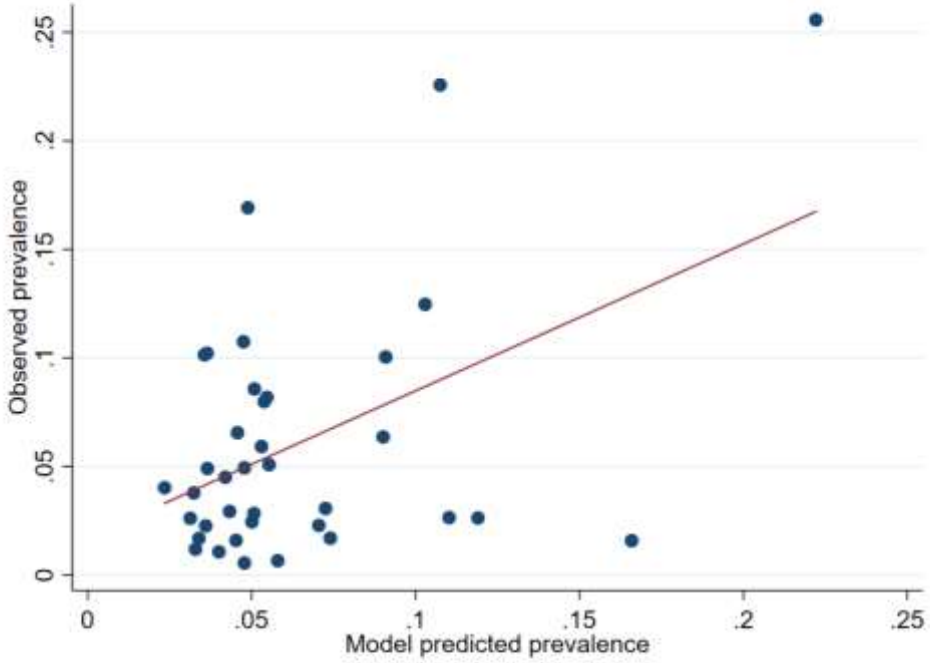


```
. spearman thin_svy thinvpost if validation_sample2==1 & thin_svy~=0

 Number of obs =      37
Spearman's rho =       0.2048

Test of Ho: thin_svy and thinvpost are independent
    Prob > |t| =       0.2239
```

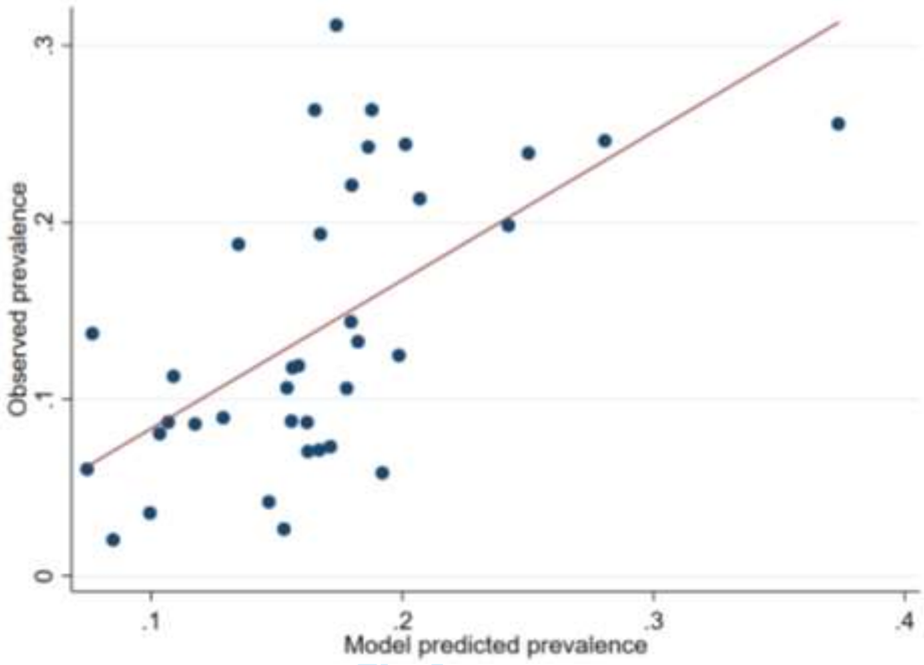
Observed prevalence
0
.1
.2
.3
.1
.2
.3
.4
Model predicted prevalence

Supplementary 5: Description of the study sample across survey rounds

Survey wave	Age (in years)	Sampled	Estimated population size using survey weights	95% CI		% sampled with height/weight measurement
**2008**	0	661	1092027	948199	1235854	35.9%
	1	661	1151665	1009086	1294244	67.9%
	2	670	1088458	960285	1216632	71.0%
	3	642	1034244	902011	1166477	81.0%
	4	620	1016227	882185	1150270	83.5%
	<5	3254	5382621	5005478	5759764	
**2010/11**	0	517	866786	720440	1013132	16.2%
	1	621	1032184	840129	1224239	42.5%
	2	751	1225419	1040085	1410753	49.3%
	3	840	1206389	1026681	1386097	53.3%
	4	820	1196800	1031500	1362101	53.3%
	<5	3549	5527578	4914106	6141050	
**2012**	0	652	902357	777704	1027010	45.1%
	1	691	1039354	887868	1190839	87.7%
	2	764	1183609	995508	1371711	87.6%
	3	826	1257820	1036042	1479598	89.6%
	4	909	1405034	1191438	1618631	87.3%
	<5	3842	5788174	5112765	6463583	
**2014/15**	0	886	1185863	1003941	1367786	50.3%
	1	875	1162949	985828	1340070	92.9%
	2	863	1060232	901257	1219207	92.7%
	3	914	1160946	985127	1336765	94.0%
	4	960	1298110	1098342	1497879	94.3%
	<5	4498	5868101	5200170	6536031	
**2017**	0	813	987763	841487	1134040	47.8%
	1	909	1215360	1045099	1385622	86.4%
	2	996	1293408	1105038	1481779	84.6%
	3	992	1264427	1088783	1440071	88.9%
	4	1000	1129184	973431	1284937	90.4%
	<5	4710	5890142	5261158	6519126	

For peer review only

Supplementary 6: Sensitivity analyses for missing weight and height

Summary: A comparison of missing weight/height proportions by various socio-demographic variables suggests that many were likely missing at random. Distributions of race, gender, household income, low birthweight, food security status, mother education category and father education category were not significantly different when comparing children with missing weight/height measurements to those with a valid weight/height measurement (please see analysis output below). However, age did significantly differ by missing status in that infants (<1 year of age) were significantly more likely to have a missing weight/height measurement compared to children aged 1-4 years. There also appeared to be significant differences in missing weight/height status by province of residence i.e. children in Mpumalanga, Western Cape fir example had higher proportions of missing weight/height measurements among children under 5 (p<0.001). Furthermore, missing weight/height measurements for children were more significantly more likely among those children with younger mothers (<25 years of age).

```
. svy: tab race_ missing_height_weight if race_~=0, row ci
(running tabulate on estimation sample)

Number of strata   =        53                Number of obs     =     16,649
Number of PSUs     =     1,076                Population size   =  25,331,414
                                              Design df         =      1,023
```

```
------------------------------------------------------
          |            missing_height_weight
    race_ |             0              1         Total
----------+-------------------------------------------
  African |         .8129          .1871             1
          | [.8006,.8246]  [.1754,.1994]
          |
 Coloured |         .7803          .2197             1
          | [.7437,.8129]  [.1871,.2563]
          |
 Asian/In |         .7593          .2407             1
          |  [.5708,.882]   [.118,.4292]
          |
    White |           .74            .26             1
          |  [.643,.8182]   [.1818,.357]
          |
    Total |         .8066          .1934             1
          | [.7945,.8181]  [.1819,.2055]
------------------------------------------------------
  Key:  row proportion
        [95% confidence interval for row proportion]

  Pearson:
    Uncorrected   chi2(3)         =   32.5162
    Design-based  F(2.49, 2551.53)=    1.7810     P = 0.1588

. svy: tab gender_ missing_height_weight if race_~=0, row ci
(running tabulate on estimation sample)

Number of strata   =        53                Number of obs     =     19,138
Number of PSUs     =     1,218                Population size   =  28,354,881
                                              Design df         =      1,165

------------------------------------------------------
          |            missing_height_weight
  gender_ |             0              1         Total
----------+-------------------------------------------
     Male |         .8065          .1935             1
          | [.7926,.8196]  [.1804,.2074]
          |
   Female |         .8102          .1898             1
          | [.7951,.8245]  [.1755,.2049]
          |
    Total |         .8083          .1917             1
          |  [.7972,.819]   [.181,.2028]
------------------------------------------------------
  Key:  row proportion
        [95% confidence interval for row proportion]

  Pearson:
    Uncorrected   chi2(1)         =    0.4400
    Design-based  F(1, 1165)      =    0.1697     P = 0.6805

. svy: tab age_ missing_height_weight, row ci
(running tabulate on estimation sample)

Number of strata   =        53                Number of obs     =     19,201
Number of PSUs     =     1,227                Population size   =  28,456,616
                                              Design df         =      1,174

------------------------------------------------------
          |            missing_height_weight
     age_ |             0              1         Total
----------+-------------------------------------------
        0 |         .4596          .5404             1
          | [.4362,.4832]  [.5168,.5638]
          |
        1 |         .8581          .1419             1
          | [.8308,.8816]  [.1184,.1692]
          |
        2 |         .8764          .1236             1
          | [.8573,.8933]  [.1067,.1427]
          |
        3 |         .8952          .1048             1
          | [.8726,.9142]  [.0858,.1274]
          |
```

For peer review only

```
        4 |         .9015          .0985              1
          |  [.8847,.916]   [.084,.1153]
          |
    Total |         .8083          .1917              1
          | [.7972,.8189]  [.1811,.2028]
-------------------------------------------------------
  Key:  row proportion
        [95% confidence interval for row proportion]

  Pearson:
    Uncorrected   chi2(4)          = 3267.7805
    Design-based  F(3.41, 3999.27)=  238.9174     P = 0.0000

. svy: tab hh_inc missing_height_weight, row ci
(running tabulate on estimation sample)

Number of strata   =        53                Number of obs     =      18,289
Number of PSUs     =     1,195                Population size   =  26,887,499
                                              Design df         =       1,142

-------------------------------------------------------
          |            missing_height_weight
   hh_inc |             0              1          Total
----------+--------------------------------------------
        1 |         .8032          .1968              1
          | [.7792,.8251]  [.1749,.2208]
          |
        2 |         .8286          .1714              1
          |  [.8012,.853]   [.147,.1988]
          |
        3 |         .8289          .1711              1
          | [.8084,.8475]  [.1525,.1916]
          |
        4 |         .8076          .1924              1
          | [.7751,.8365]  [.1635,.2249]
          |
        5 |         .7862          .2138              1
          |  [.7578,.812]   [.188,.2422]
          |
    Total |         .8096          .1904              1
          | [.7982,.8205]  [.1795,.2018]
-------------------------------------------------------
  Key:  row proportion
        [95% confidence interval for row proportion]

  Pearson:
    Uncorrected   chi2(4)          =   32.2620
    Design-based  F(3.67, 4186.36)=    1.9756     P = 0.1017

. svy: tab province missing_height_weight, row ci
(running tabulate on estimation sample)

Number of strata   =        53                Number of obs     =      19,201
Number of PSUs     =     1,227                Population size   =  28,456,616
                                              Design df         =       1,174

-------------------------------------------------------
          |            missing_height_weight
 province |             0              1          Total
----------+--------------------------------------------
  Eastern |         .8421          .1579              1
          |  [.819,.8627]   [.1373,.181]
          |
 Free Sta |          .833           .167              1
          | [.7968,.8638]  [.1362,.2032]
          |
  Gauteng |         .7866          .2134              1
          | [.7637,.8078]  [.1922,.2363]
          |
 KwaZulu- |         .8448          .1552              1
          | [.8255,.8624]  [.1376,.1745]
          |
  Limpopo |         .8422          .1578              1
          | [.8184,.8634]  [.1366,.1816]
          |
 Mpumalan |         .7557          .2443              1
          | [.7187,.7892]  [.2108,.2813]
```

For peer review only

```
           |
 North We |          .8011          .1989               1
          |  [.7725,.827]   [.173,.2275]
          |
 Northern |          .7921          .2079               1
          | [.7674,.8149]  [.1851,.2326]
          |
  Western |          .7422          .2578               1
          |  [.7064,.775]   [.225,.2936]
          |
    Total |          .8083          .1917               1
          | [.7972,.8189]  [.1811,.2028]
-------------------------------------------------------
  Key:  row proportion
        [95% confidence interval for row proportion]

  Pearson:
    Uncorrected   chi2(8)         =  171.9467
    Design-based  F(6.89, 8090.45)=    9.8218     P = 0.0000

. svy: tab LBW missing_height_weight, row ci
(running tabulate on estimation sample)

Number of strata   =        53                 Number of obs     =      16,606
Number of PSUs     =     1,128                 Population size   =  24,829,511
                                               Design df         =       1,075

-------------------------------------------------------
          |            missing_height_weight
      LBW |             0              1          Total
----------+--------------------------------------------
        0 |          .8164          .1836               1
          | [.8044,.8278]  [.1722,.1956]
          |
        1 |          .8106          .1894               1
          | [.7788,.8388]  [.1612,.2212]
          |
    Total |          .8158          .1842               1
          | [.8045,.8266]  [.1734,.1955]
-------------------------------------------------------
  Key:  row proportion
        [95% confidence interval for row proportion]

  Pearson:
    Uncorrected   chi2(1)         =    0.3369
    Design-based  F(1, 1075)      =    0.1307     P = 0.7178

. svy: tab foodsecurity_proxy missing_height_weight, row ci
(running tabulate on estimation sample)

Number of strata   =        53                  Number of obs     =      5,017
Number of PSUs     =       438                  Population size   =  8,843,019
                                                Design df         =        385

-------------------------------------------------------
foodsecur |            missing_height_weight
ity_proxy |             0              1          Total
----------+--------------------------------------------
        1 |          .7719          .2281               1
          | [.7467,.7952]  [.2048,.2533]
          |
        2 |          .8019          .1981               1
          | [.7352,.8551]  [.1449,.2648]
          |
        3 |          .7596          .2404               1
          | [.6983,.8119]  [.1881,.3017]
          |
        4 |          .8284          .1716               1
          | [.7561,.8825]  [.1175,.2439]
          |
        5 |          .7869          .2131               1
          | [.6923,.8583]  [.1417,.3077]
          |
    Total |          .7751          .2249               1
          |  [.753,.7959]   [.2041,.247]
-------------------------------------------------------
  Key:  row proportion
```

For peer review only

```
          [95% confidence interval for row proportion]

  Pearson:
    Uncorrected   chi2(4)         =    6.4682
    Design-based  F(3.04, 1168.67)=    0.8267     P = 0.4803

. svy: tab mthagegrp missing_height_weight, row ci
(running tabulate on estimation sample)

Number of strata   =        53                  Number of obs     =     17,335
Number of PSUs     =     1,192                  Population size   =  26,432,345
                                                Design df         =      1,139

-------------------------------------------------------
RECODE of |
mth_age_f |            missing_height_weight
inal      |              0              1         Total
----------+--------------------------------------------
        1 |          .6691          .3309             1
          |  [.628,.7077]   [.2923,.372]
          |
        2 |          .7787          .2213             1
          | [.7553,.8004]  [.1996,.2447]
          |
        3 |          .8128          .1872             1
          | [.7961,.8285]  [.1715,.2039]
          |
        4 |          .8329          .1671             1
          | [.8096,.8539]  [.1461,.1904]
          |
        5 |          .8629          .1371             1
          | [.7939,.9113]  [.0887,.2061]
          |
    Total |          .8006          .1994             1
          | [.7894,.8115]  [.1885,.2106]
-------------------------------------------------------
  Key:  row proportion
        [95% confidence interval for row proportion]

  Pearson:
    Uncorrected   chi2(4)         =  169.4906
    Design-based  F(3.89, 4436.22)=   15.6564     P = 0.0000

. svy: tab mth_edu2 missing_height_weight, row ci
(running tabulate on estimation sample)

Number of strata   =        53                  Number of obs     =     16,352
Number of PSUs     =     1,169                  Population size   =  25,254,660
                                                Design df         =      1,116

-------------------------------------------------------
          |            missing_height_weight
 mth_edu2 |              0              1         Total
----------+--------------------------------------------
        0 |          .8043          .1957             1
          | [.7537,.8466]  [.1534,.2463]
          |
        1 |          .7848          .2152             1
          | [.7529,.8136]  [.1864,.2471]
          |
        2 |          .7976          .2024             1
          | [.7848,.8098]  [.1902,.2152]
          |
        3 |          .8122          .1878             1
          | [.7734,.8457]  [.1543,.2266]
          |
    Total |          .7982          .2018             1
          | [.7868,.8092]  [.1908,.2132]
-------------------------------------------------------
  Key:  row proportion
        [95% confidence interval for row proportion]

  Pearson:
    Uncorrected   chi2(3)         =    3.7648
    Design-based  F(2.41, 2688.60)=    0.5454     P = 0.6124

. svy: tab fth_educat missing_height_weight, row ci
```

For peer review only

```
(running tabulate on estimation sample)

Number of strata   =        53                  Number of obs      =     4,574
Number of PSUs     =       755                  Population size    = 8,485,206
                                                Design df          =       702

-------------------------------------------------------
fth_educa |            missing_height_weight
t         |             0              1          Total
----------+--------------------------------------------
        0 |         .9417          .0583              1
          |  [.732,.9896]   [.0104,.268]
          |
        1 |         .7923          .2077              1
          | [.7634,.8185]  [.1815,.2366]
          |
        2 |          .803           .197              1
          |  [.759,.8406]   [.1594,.241]
          |
        3 |         .7618          .2382              1
          | [.6661,.8368]  [.1632,.3339]
          |
    Total |         .7948          .2052              1
          | [.7719,.8159]  [.1841,.2281]
-------------------------------------------------------
  Key:  row proportion
        [95% confidence interval for row proportion]

  Pearson:
    Uncorrected   chi2(3)         =    3.8826
    Design-based  F(2.17, 1522.03)=    0.5250     P = 0.6062
```

Supplementary 7: Full posterior prevalence estimates with 95% Bayesian uncertainty intervals (UIs) by district and year. Also includes exceedance probabilities for the 5% target threshold for wasting prevalence.

Province	District	wave	stunting	95% UI		thinness	95% UI		Exceedance probability 5% target threshold	obesity	95% UI	
Free State	Mangaung(MAN)	1	0.376	0.2267	0.5446	0.08848	0.01452	0.2771	0.6775	0.1388	0.04626	0.2953
Eastern Cape	Nelson Mandela Bay(NMA)	1	0.1189	0.04999	0.2169	0.04979	0.01135	0.1198	0.416	0.2803	0.1662	0.4144
Gauteng	City of Tshwane(TSH)	1	0.1683	0.1008	0.254	0.1242	0.06282	0.2126	0.9958	0.1023	0.05515	0.164
Gauteng	City of Johannesburg(JHB)	1	0.1089	0.05726	0.1785	0.05937	0.02058	0.1172	0.6032	0.1458	0.08299	0.229
Eastern Cape	Buffalo City(BUF)	1	0.3057	0.1065	0.5683	0.2221	0.04924	0.5067	0.9734	0.2873	0.09798	0.5453
Western Cape	City of Cape Town(CPT)	1	0.08183	0.03667	0.1476	0.08437	0.01581	0.2479	0.6765	0.1667	0.09279	0.2583
Western Cape	West Coast(DC1)	1	0.1033	0.04348	0.1936	0.09203	0.0153	0.2846	0.6962	0.2789	0.1598	0.4302
Eastern Cape	Cacadu(DC10)	1	0.2199	0.1344	0.3257	0.08308	0.02311	0.2087	0.7507	0.2068	0.1249	0.3062
Eastern Cape	Amathole(DC12)	1	0.2096	0.099	0.3623	0.1707	0.0536	0.3787	0.9811	0.2921	0.153	0.477
Eastern Cape	Chris Hani(DC13)	1	0.1357	0.07341	0.2189	0.0779	0.01946	0.2061	0.6891	0.2803	0.1811	0.4016
Eastern Cape	Joe Gqabi(DC14)	1	0.1464	0.08195	0.231	0.04274	0.01315	0.09161	0.3041	0.1916	0.1149	0.2862
Eastern Cape	O.R.Tambo(DC15)	1	0.1994	0.1267	0.2865	0.03065	0.008741	0.06674	0.1054	0.2481	0.1677	0.3417
Free State	Xhariep(DC16)	1	0.2156	0.1287	0.3248	0.06445	0.0169	0.1599	0.5905	0.1255	0.06464	0.2034
Free State	Lejweleputswa(DC18)	1	0.1452	0.0797	0.2328	0.08649	0.03458	0.1694	0.8808	0.1133	0.05684	0.1874
Free State	Thabo Mofutsanyane(DC19)	1	0.131	0.07164	0.2114	0.07928	0.02582	0.1879	0.7601	0.1564	0.0887	0.2497
Western Cape	Cape Winelands(DC2)	1	0.1264	0.0578	0.2302	0.0897	0.03798	0.169	0.9073	0.198	0.1221	0.2919
Free State	Fezile Dabi(DC20)	1	0.126	0.06157	0.219	0.1013	0.02768	0.2575	0.8474	0.1949	0.1041	0.3311
KwaZulu-Natal	Ugu(DC21)	1	0.07495	0.03921	0.1244	0.02685	0.008281	0.05741	0.0514	0.1864	0.1208	0.2644
KwaZulu-Natal	UMgungundlovu(DC22)	1	0.07298	0.03734	0.1239	0.03899	0.01355	0.08126	0.2228	0.1886	0.1164	0.2778
KwaZulu-Natal	Uthukela(DC23)	1	0.05198	0.02714	0.08637	0.03509	0.01446	0.06594	0.1308	0.1134	0.0695	0.1666
KwaZulu-Natal	Umzinyathi(DC24)	1	0.07512	0.0338	0.1411	0.03915	0.009919	0.09834	0.2381	0.1778	0.08775	0.3065

For peer review only

KwaZulu-Natal	Amajuba(DC25)	1	0.07637	0.03943	0.1284	0.04711	0.01877	0.09163	0.3801	0.1437	0.08594	0.2183
KwaZulu-Natal	Zululand(DC26)	1	0.07006	0.03489	0.1207	0.03588	0.0118	0.07437	0.1742	0.1256	0.07	0.1962
KwaZulu-Natal	Umkhanyakude(DC27)	1	0.09456	0.04001	0.175	0.08258	0.02853	0.1714	0.8135	0.1298	0.06118	0.2217
KwaZulu-Natal	Uthungulu(DC28)	1	0.09427	0.04948	0.1555	0.03186	0.009784	0.06811	0.1188	0.1858	0.1162	0.2708
KwaZulu-Natal	iLembe(DC29)	1	0.08274	0.0415	0.1414	0.03548	0.007382	0.09664	0.1972	0.3496	0.2412	0.4721
Western Cape	Overberg(DC3)	1	0.1153	0.05468	0.2007	0.0475	0.01269	0.1077	0.3856	0.1692	0.09083	0.2686
Mpumalanga	Gert Sibande(DC30)	1	0.08364	0.04684	0.1334	0.05459	0.02229	0.1009	0.5467	0.1179	0.07005	0.1778
Mpumalanga	Nkangala(DC31)	1	0.1283	0.07398	0.2	0.1114	0.05548	0.1954	0.9876	0.1218	0.07052	0.1913
Mpumalanga	Ehlanzeni(DC32)	1	0.1007	0.05339	0.1647	0.04363	0.01466	0.08845	0.3217	0.07789	0.03733	0.1324
Limpopo	Mopani(DC33)	1	0.0946	0.04588	0.1636	0.05868	0.02062	0.1191	0.5802	0.09186	0.0423	0.1598
Limpopo	Vhembe(DC34)	1	0.2729	0.1553	0.4137	0.0452	0.009503	0.1105	0.349	0.2078	0.1067	0.3407
Limpopo	Capricorn(DC35)	1	0.1332	0.06996	0.2186	0.1062	0.04626	0.1982	0.9617	0.09787	0.0345	0.1996
Limpopo	Waterberg(DC36)	1	0.1261	0.0715	0.1983	0.07607	0.03322	0.1386	0.8402	0.1134	0.06302	0.1807
North West	Bojanala(DC37)	1	0.1086	0.05322	0.1879	0.07376	0.02457	0.1508	0.7525	0.1042	0.04922	0.1792
North West	Ngaka Modiri Molema(DC38)	1	0.1114	0.05798	0.1846	0.07361	0.02921	0.1387	0.7964	0.1609	0.09222	0.2531
North West	Dr Ruth Segomotsi Mompati(DC39)	1	0.1133	0.06476	0.1768	0.1286	0.06945	0.21	0.9986	0.1112	0.06321	0.172
Western Cape	Eden(DC4)	1	0.1613	0.08269	0.2684	0.1121	0.0251	0.3225	0.8304	0.2252	0.1283	0.3511
North West	Dr Kenneth Kaunda(DC40)	1	0.1322	0.07233	0.2136	0.1378	0.06359	0.2547	0.9952	0.1387	0.0778	0.2225
Gauteng	Sedibeng(DC42)	1	0.1448	0.08442	0.2242	0.1429	0.07209	0.2485	0.9988	0.1185	0.06673	0.1861
KwaZulu-Natal	Sisonke(DC43)	1	0.1628	0.0938	0.2512	0.07959	0.03336	0.1513	0.8455	0.205	0.1269	0.2995
Eastern Cape	Alfred Nzo(DC44)	1	0.101	0.05069	0.1714	0.05384	0.01883	0.1131	0.4939	0.1642	0.09067	0.2552
Northern Cape	John Taolo Gaetsewe(DC45)	1	0.08483	0.03592	0.1593	0.06003	0.01797	0.1293	0.5806	0.1211	0.05718	0.2104
Limpopo	Greater Sekhukhune(DC47)	1	0.1366	0.07765	0.2139	0.07672	0.03378	0.1412	0.8441	0.0718	0.03253	0.1246
Gauteng	West Rand(DC48)	1	0.1172	0.05904	0.1994	0.09976	0.04016	0.1944	0.9326	0.1175	0.05831	0.1984
Western Cape	Central Karoo(DC5)	1	0.1778	0.1044	0.2711	0.07236	0.02868	0.1401	0.774	0.1676	0.097	0.255
Northern Cape	Namakwa(DC6)	1	0.1577	0.08655	0.2519	0.07517	0.028	0.1502	0.7827	0.1425	0.07457	0.2277
Northern Cape	Pixley ka Seme(DC7)	1	0.2215	0.1349	0.3311	0.07768	0.02588	0.178	0.7614	0.2182	0.133	0.3377
Northern Cape	Siyanda(DC8)	1	0.1399	0.07658	0.2232	0.07717	0.03067	0.1474	0.8224	0.1098	0.05505	0.1807
Northern Cape	Frances Baard(DC9)	1	0.1411	0.08378	0.2142	0.04756	0.01745	0.09265	0.3972	0.1034	0.05587	0.1634
Gauteng	Ekurhuleni(EKU)	1	0.1382	0.07203	0.2283	0.06411	0.02062	0.1316	0.6496	0.09407	0.04302	0.1632
KwaZulu-Natal	eThekwini(ETH)	1	0.06433	0.02926	0.1163	0.02767	0.007316	0.06364	0.0782	0.1984	0.1209	0.291
Free State	Mangaung(MAN)	2	0.1713	0.07974	0.2963	0.1215	0.03342	0.2957	0.902	0.2099	0.08075	0.4079
Eastern Cape	Nelson Mandela Bay(NMA)	2	0.1029	0.04638	0.1823	0.06417	0.02285	0.1291	0.6567	0.1404	0.07152	0.2302
Gauteng	City of Tshwane(TSH)	2	0.1607	0.1016	0.234	0.08938	0.03626	0.1775	0.8871	0.1908	0.1251	0.2707
Gauteng	City of Johannesburg(JHB)	2	0.1516	0.09194	0.2258	0.08218	0.02812	0.18	0.7932	0.1646	0.1024	0.2409
Eastern Cape	Buffalo City(BUF)	2	0.2059	0.02986	0.5744	0.3731	0.07256	0.7907	0.991	0.2738	0.04665	0.6668
Western Cape	City of Cape Town(CPT)	2	0.1777	0.106	0.2658	0.1548	0.08685	0.2417	0.9999	0.3767	0.2749	0.4855
Western Cape	West Coast(DC1)	2	0.1343	0.04711	0.2845	0.1798	0.05736	0.3891	0.9855	0.2965	0.1278	0.5219
Eastern Cape	Cacadu(DC10)	2	0.1588	0.09395	0.2417	0.154	0.06192	0.303	0.9921	0.1047	0.05618	0.1699
Eastern Cape	Amathole(DC12)	2	0.1719	0.06688	0.3391	0.3305	0.1636	0.5332	1	0.2013	0.08035	0.3844
Eastern Cape	Chris Hani(DC13)	2	0.1168	0.06562	0.1848	0.1492	0.05631	0.3041	0.9866	0.2189	0.141	0.3115
Eastern Cape	Joe Gqabi(DC14)	2	0.1181	0.06511	0.1878	0.08033	0.02881	0.1705	0.799	0.2063	0.1299	0.2985
Eastern Cape	O.R.Tambo(DC15)	2	0.2348	0.1557	0.3276	0.05506	0.02331	0.1031	0.5448	0.2949	0.2071	0.393
Free State	Xhariep(DC16)	2	0.1449	0.07114	0.2515	0.09865	0.03815	0.1994	0.915	0.2124	0.1259	0.3207
Free State	Lejweleputswa(DC18)	2	0.1816	0.1113	0.2705	0.1226	0.06554	0.2012	0.9971	0.1569	0.08066	0.2654
Free State	Thabo Mofutsanyane(DC19)	2	0.1453	0.08288	0.2286	0.1087	0.05234	0.1922	0.9804	0.2369	0.1468	0.3476

For peer review only

Western Cape	Cape Winelands(DC2)	2	0.1203	0.06752	0.1894	0.1783	0.1046	0.2718	1	0.2458	0.1623	0.3432
Free State	Fezile Dabi(DC20)	2	0.1615	0.0772	0.2855	0.1108	0.04032	0.237	0.9349	0.1853	0.1018	0.2942
KwaZulu-Natal	Ugu(DC21)	2	0.1778	0.1186	0.2481	0.04676	0.02163	0.08407	0.3704	0.3626	0.2788	0.4518
KwaZulu-Natal	UMgungundlovu(DC22)	2	0.1764	0.1122	0.2555	0.05619	0.02232	0.1151	0.5322	0.2058	0.1353	0.2888
KwaZulu-Natal	Uthukela(DC23)	2	0.1069	0.06915	0.1536	0.03332	0.01643	0.05773	0.0719	0.2463	0.1856	0.314
KwaZulu-Natal	Umzinyathi(DC24)	2	0.1182	0.05791	0.2076	0.05054	0.01868	0.1086	0.4242	0.2115	0.11	0.3491
KwaZulu-Natal	Amajuba(DC25)	2	0.07926	0.04422	0.1262	0.06104	0.03099	0.1039	0.6937	0.1281	0.07894	0.1891
KwaZulu-Natal	Zululand(DC26)	2	0.11	0.06136	0.1748	0.05228	0.023	0.09729	0.4865	0.1448	0.08545	0.2194
KwaZulu-Natal	Umkhanyakude(DC27)	2	0.1828	0.08925	0.3076	0.07254	0.01724	0.1923	0.6246	0.3106	0.1822	0.4597
KwaZulu-Natal	Uthungulu(DC28)	2	0.1508	0.08907	0.2284	0.04962	0.02121	0.09368	0.4297	0.2798	0.1918	0.3792
KwaZulu-Natal	iLembe(DC29)	2	0.09978	0.05305	0.1626	0.04743	0.01932	0.09164	0.382	0.299	0.2061	0.403
Western Cape	Overberg(DC3)	2	0.1689	0.06326	0.34	0.1069	0.04838	0.1905	0.9697	0.2617	0.1625	0.3779
Mpumalanga	Gert Sibande(DC30)	2	0.1272	0.08051	0.1858	0.0872	0.04003	0.1613	0.9131	0.1952	0.1337	0.2682
Mpumalanga	Nkangala(DC31)	2	0.1089	0.06387	0.168	0.08483	0.03725	0.1619	0.8879	0.1729	0.09377	0.2813
Mpumalanga	Ehlanzeni(DC32)	2	0.1109	0.06154	0.1756	0.05599	0.02578	0.1013	0.5748	0.1369	0.08076	0.2082
Limpopo	Mopani(DC33)	2	0.1431	0.07957	0.2256	0.0617	0.02606	0.1172	0.653	0.2044	0.1242	0.3026
Limpopo	Vhembe(DC34)	2	0.3445	0.2164	0.4879	0.04543	0.009324	0.1301	0.3269	0.3187	0.1959	0.4596
Limpopo	Capricorn(DC35)	2	0.1974	0.1192	0.2941	0.07428	0.03325	0.1356	0.8247	0.09946	0.05098	0.1659
Limpopo	Waterberg(DC36)	2	0.1778	0.1137	0.2566	0.06741	0.0335	0.1164	0.7848	0.1342	0.08162	0.2012
North West	Bojanala(DC37)	2	0.1266	0.0697	0.2024	0.07792	0.02931	0.1618	0.7908	0.1476	0.08438	0.23
North West	Ngaka Modiri Molema(DC38)	2	0.1954	0.1226	0.2846	0.1056	0.05521	0.1743	0.9881	0.109	0.05876	0.1752
North West	Dr Ruth Segomotsi Mompati(DC39)	2	0.1692	0.106	0.2468	0.132	0.07636	0.2046	0.9996	0.1239	0.07322	0.1886
Western Cape	Eden(DC4)	2	0.2097	0.1112	0.3394	0.2323	0.08294	0.4752	0.9983	0.1827	0.0791	0.3372
North West	Dr Kenneth Kaunda(DC40)	2	0.2127	0.1307	0.3157	0.1207	0.05429	0.2259	0.985	0.1584	0.09257	0.2445
Gauteng	Sedibeng(DC42)	2	0.1454	0.08982	0.2158	0.101	0.05312	0.1667	0.9842	0.1549	0.09703	0.2269
KwaZulu-Natal	Sisonke(DC43)	2	0.2124	0.1354	0.3036	0.09505	0.02861	0.2192	0.836	0.5023	0.3943	0.6106
Eastern Cape	Alfred Nzo(DC44)	2	0.1742	0.09988	0.2692	0.07957	0.0269	0.1764	0.7721	0.2141	0.1286	0.318
Northern Cape	John Taolo Gaetsewe(DC45)	2	0.07937	0.03288	0.1509	0.1481	0.07062	0.2554	0.9974	0.11	0.05066	0.1953
Limpopo	Greater Sekhukhune(DC47)	2	0.1711	0.1089	0.2471	0.0565	0.02749	0.09913	0.5941	0.1002	0.05735	0.156
Gauteng	West Rand(DC48)	2	0.1302	0.07144	0.209	0.07501	0.03295	0.1391	0.8238	0.2145	0.1305	0.318
Western Cape	Central Karoo(DC5)	2	0.1202	0.06622	0.1919	0.1525	0.06198	0.2952	0.9924	0.1561	0.07059	0.2849
Northern Cape	Namakwa(DC6)	2	0.1246	0.05504	0.2328	0.1907	0.08144	0.3554	0.9989	0.1582	0.07077	0.2903
Northern Cape	Pixley ka Seme(DC7)	2	0.1307	0.06888	0.2186	0.1639	0.07476	0.3043	0.9986	0.1658	0.08984	0.2696
Northern Cape	Siyanda(DC8)	2	0.09598	0.04842	0.1621	0.2573	0.1599	0.3732	1	0.08701	0.04297	0.1491
Northern Cape	Frances Baard(DC9)	2	0.1295	0.07766	0.1958	0.07582	0.03871	0.1274	0.8855	0.1067	0.06185	0.1654
Gauteng	Ekurhuleni(EKU)	2	0.1112	0.05864	0.1828	0.06712	0.02181	0.151	0.6442	0.2049	0.1248	0.3035
KwaZulu-Natal	eThekwini(ETH)	2	0.1541	0.09327	0.2296	0.04473	0.01898	0.08459	0.3271	0.3253	0.2353	0.4239
Free State	Mangaung(MAN)	3	0.1796	0.09535	0.2888	0.0718	0.02092	0.1736	0.6634	0.1433	0.07081	0.2421
Eastern Cape	Nelson Mandela Bay(NMA)	3	0.165	0.08202	0.2734	0.04625	0.01246	0.1066	0.36	0.1786	0.0921	0.2888
Gauteng	City of Tshwane(TSH)	3	0.09553	0.05602	0.1474	0.05805	0.02975	0.09837	0.6406	0.1444	0.09192	0.2094
Gauteng	City of Johannesburg(JHB)	3	0.1386	0.08344	0.2082	0.09279	0.04909	0.1526	0.972	0.1436	0.088	0.2134
Eastern Cape	Buffalo City(BUF)	3	0.1372	0.01922	0.4191	0.1599	0.01787	0.5186	0.8231	0.3951	0.1597	0.6695
Western Cape	City of Cape Town(CPT)	3	0.08289	0.04357	0.1361	0.05161	0.0232	0.09386	0.4803	0.2383	0.1639	0.3231
Western Cape	West Coast(DC1)	3	0.05897	0.02383	0.1142	0.07063	0.02809	0.1372	0.7527	0.1846	0.1021	0.2899
Eastern Cape	Cacadu(DC10)	3	0.09913	0.05348	0.162	0.07902	0.03841	0.1384	0.8888	0.1247	0.0704	0.1959
Eastern Cape	Amathole(DC12)	3	0.1125	0.0483	0.21	0.1222	0.04755	0.2395	0.9682	0.177	0.08792	0.297

For peer review only

Eastern Cape	Chris Hani(DC13)	3	0.1191	0.06821	0.1853	0.08486	0.04285	0.1444	0.9347	0.2082	0.1348	0.2952
Eastern Cape	Joe Gqabi(DC14)	3	0.1757	0.1063	0.2627	0.04569	0.01966	0.08636	0.3454	0.2665	0.178	0.3695
Eastern Cape	O.R.Tambo(DC15)	3	0.1378	0.08116	0.2099	0.03496	0.0137	0.069	0.1386	0.3064	0.2167	0.4054
Free State	Xhariep(DC16)	3	0.1183	0.06517	0.1899	0.05539	0.02437	0.1038	0.5477	0.1298	0.07328	0.204
Free State	Lejweleputswa(DC18)	3	0.1578	0.09485	0.2381	0.06957	0.03491	0.1203	0.8155	0.1247	0.07255	0.1934
Free State	Thabo Mofutsanyane(DC19)	3	0.163	0.09673	0.2486	0.06561	0.03092	0.1188	0.7382	0.156	0.09155	0.2386
Western Cape	Cape Winelands(DC2)	3	0.07268	0.03731	0.1233	0.06535	0.03095	0.1164	0.7413	0.1678	0.1021	0.2506
Free State	Fezile Dabi(DC20)	3	0.1435	0.07546	0.2375	0.07561	0.02844	0.161	0.7675	0.1733	0.09298	0.2798
KwaZulu-Natal	Ugu(DC21)	3	0.1749	0.117	0.2444	0.06093	0.03166	0.1021	0.7033	0.2622	0.1911	0.3414
KwaZulu-Natal	UMgungundlovu(DC22)	3	0.1384	0.08452	0.2071	0.06935	0.03519	0.1188	0.8178	0.2108	0.1402	0.295
KwaZulu-Natal	Uthukela(DC23)	3	0.1392	0.09633	0.1903	0.03777	0.01977	0.06308	0.1357	0.2345	0.1779	0.2975
KwaZulu-Natal	Umzinyathi(DC24)	3	0.1432	0.07187	0.2471	0.04596	0.01806	0.09536	0.3416	0.2133	0.111	0.3508
KwaZulu-Natal	Amajuba(DC25)	3	0.1271	0.0746	0.1953	0.04906	0.02308	0.08819	0.4175	0.1494	0.09112	0.2224
KwaZulu-Natal	Zululand(DC26)	3	0.13	0.07621	0.1989	0.03693	0.01626	0.06926	0.1535	0.245	0.1649	0.3383
KwaZulu-Natal	Umkhanyakude(DC27)	3	0.1065	0.05418	0.1766	0.02944	0.009015	0.06572	0.0913	0.1993	0.1229	0.2916
KwaZulu-Natal	Uthungulu(DC28)	3	0.1633	0.1007	0.2407	0.03355	0.01402	0.06467	0.1066	0.2377	0.1608	0.3265
KwaZulu-Natal	iLembe(DC29)	3	0.1567	0.08731	0.247	0.04675	0.01943	0.09049	0.3659	0.2126	0.1297	0.3134
Western Cape	Overberg(DC3)	3	0.0704	0.0317	0.1275	0.05608	0.01646	0.1344	0.4869	0.3234	0.2199	0.4389
Mpumalanga	Gert Sibande(DC30)	3	0.1385	0.08868	0.2001	0.1	0.058	0.1568	0.9935	0.2184	0.1525	0.2946
Mpumalanga	Nkangala(DC31)	3	0.08912	0.04967	0.1423	0.06322	0.03152	0.1102	0.723	0.1816	0.1142	0.2646
Mpumalanga	Ehlanzeni(DC32)	3	0.2547	0.179	0.3411	0.07772	0.04149	0.1277	0.9132	0.2431	0.1712	0.325
Limpopo	Mopani(DC33)	3	0.2269	0.1402	0.3321	0.07594	0.03503	0.1374	0.8468	0.2602	0.1678	0.3688
Limpopo	Vhembe(DC34)	3	0.08287	0.03497	0.1527	0.03008	0.008862	0.0689	0.1045	0.09792	0.04537	0.1711
Limpopo	Capricorn(DC35)	3	0.165	0.101	0.2445	0.04567	0.02002	0.08496	0.3423	0.1152	0.06539	0.1801
Limpopo	Waterberg(DC36)	3	0.1238	0.07581	0.1848	0.0486	0.02109	0.09374	0.3999	0.1087	0.06519	0.1645
North West	Bojanala(DC37)	3	0.09938	0.05396	0.1605	0.05226	0.02302	0.09714	0.4889	0.1211	0.06903	0.1891
North West	Ngaka Modiri Molema(DC38)	3	0.1633	0.1012	0.2402	0.06389	0.03132	0.1109	0.7345	0.2329	0.1564	0.3223
North West	Dr Ruth Segomotsi Mompati(DC39)	3	0.1945	0.1238	0.281	0.05648	0.02712	0.0996	0.5941	0.1384	0.08252	0.2097
Western Cape	Eden(DC4)	3	0.0843	0.03756	0.1559	0.1612	0.07344	0.2884	0.9981	0.132	0.06445	0.2259
North West	Dr Kenneth Kaunda(DC40)	3	0.1625	0.09505	0.2509	0.0677	0.02986	0.1307	0.7358	0.1677	0.09896	0.2572
Gauteng	Sedibeng(DC42)	3	0.1142	0.06875	0.1729	0.06461	0.0341	0.1078	0.7708	0.1466	0.0927	0.2138
KwaZulu-Natal	Sisonke(DC43)	3	0.155	0.0975	0.2252	0.09775	0.05317	0.1579	0.9841	0.296	0.2152	0.3851
Eastern Cape	Alfred Nzo(DC44)	3	0.1841	0.1084	0.2793	0.06464	0.02845	0.1201	0.7047	0.2548	0.1629	0.3633
Northern Cape	John Taolo Gaetsewe(DC45)	3	0.09068	0.04187	0.1614	0.06867	0.02895	0.1298	0.7468	0.1379	0.07142	0.2282
Limpopo	Greater Sekhukhune(DC47)	3	0.1279	0.08001	0.1869	0.04788	0.02424	0.08181	0.3928	0.1478	0.09599	0.2112
Gauteng	West Rand(DC48)	3	0.1617	0.09056	0.2555	0.0502	0.02172	0.09522	0.4424	0.1884	0.1098	0.2885
Western Cape	Central Karoo(DC5)	3	0.09703	0.05147	0.1598	0.06807	0.03197	0.1213	0.7748	0.1204	0.06643	0.1921
Northern Cape	Namakwa(DC6)	3	0.1057	0.05441	0.1782	0.1011	0.04923	0.1768	0.9714	0.1123	0.05835	0.1869
Northern Cape	Pixley ka Seme(DC7)	3	0.1166	0.06284	0.1935	0.1084	0.05628	0.1856	0.9895	0.1394	0.08035	0.2178
Northern Cape	Siyanda(DC8)	3	0.126	0.0721	0.1967	0.1382	0.07841	0.2165	0.9996	0.0805	0.04126	0.135
Northern Cape	Frances Baard(DC9)	3	0.1029	0.05921	0.1605	0.0524	0.02539	0.09201	0.5014	0.1024	0.05881	0.1594
Gauteng	Ekurhuleni(EKU)	3	0.07632	0.0379	0.1304	0.05052	0.02234	0.09407	0.4531	0.1519	0.08918	0.2311
KwaZulu-Natal	eThekwini(ETH)	3	0.1044	0.06208	0.1582	0.05228	0.02551	0.0907	0.5028	0.2659	0.194	0.3456
Free State	Mangaung(MAN)	4	0.0977	0.04448	0.1762	0.05627	0.0198	0.118	0.5279	0.1446	0.073	0.2422
Eastern Cape	Nelson Mandela Bay(NMA)	4	0.1177	0.05435	0.2048	0.03282	0.003788	0.1215	0.183	0.2203	0.1292	0.3297
Gauteng	City of Tshwane(TSH)	4	0.04195	0.02183	0.07053	0.04327	0.02172	0.07455	0.2748	0.06547	0.03711	0.1032

Gauteng	City of Johannesburg(JHB)	4	0.05362	0.02747	0.09045	0.08226	0.04506	0.1328	0.9462	0.07729	0.04359	0.122
Eastern Cape	Buffalo City(BUF)	4	0.1071	0.02688	0.2502	0.06609	0.004739	0.284	0.4216	0.2966	0.1304	0.5039
Western Cape	City of Cape Town(CPT)	4	0.05357	0.01625	0.1266	0.05948	0.02668	0.108	0.6312	0.144	0.08642	0.2156
Western Cape	West Coast(DC1)	4	0.03934	0.01405	0.08209	0.04707	0.01736	0.09626	0.3722	0.0695	0.03037	0.1278
Eastern Cape	Cacadu(DC10)	4	0.08965	0.04781	0.1477	0.0372	0.01513	0.07357	0.1773	0.2337	0.1483	0.3357
Eastern Cape	Amathole(DC12)	4	0.09697	0.0427	0.1797	0.04584	0.01202	0.119	0.3369	0.2448	0.1342	0.3844
Eastern Cape	Chris Hani(DC13)	4	0.0985	0.05515	0.1562	0.03396	0.01411	0.06593	0.1177	0.1778	0.1128	0.2565
Eastern Cape	Joe Gqabi(DC14)	4	0.1651	0.09877	0.2497	0.02564	0.009752	0.05238	0.0337	0.1461	0.08581	0.2231
Eastern Cape	O.R.Tambo(DC15)	4	0.09858	0.05902	0.1492	0.02015	0.00569	0.04963	0.0234	0.248	0.1795	0.3243
Free State	Xhariep(DC16)	4	0.132	0.0749	0.2078	0.04248	0.01675	0.08698	0.2766	0.09104	0.04874	0.1498
Free State	Lejweleputswa(DC18)	4	0.122	0.07281	0.1858	0.05533	0.02769	0.09611	0.5723	0.0928	0.05353	0.1454
Free State	Thabo Mofutsanyane(DC19)	4	0.08804	0.0483	0.1434	0.05133	0.02209	0.1006	0.4545	0.1221	0.07062	0.1902
Western Cape	Cape Winelands(DC2)	4	0.05301	0.02622	0.09161	0.04811	0.01891	0.0987	0.3859	0.1004	0.05856	0.155
Free State	Fezile Dabi(DC20)	4	0.07487	0.03629	0.1317	0.0557	0.02053	0.12	0.5098	0.1863	0.1055	0.2914
KwaZulu-Natal	Ugu(DC21)	4	0.08087	0.04628	0.1257	0.02403	0.01021	0.04599	0.0137	0.1765	0.1201	0.2429
KwaZulu-Natal	UMgungundlovu(DC22)	4	0.07222	0.04072	0.1148	0.02711	0.01228	0.05051	0.0275	0.1508	0.09742	0.2161
KwaZulu-Natal	Uthukela(DC23)	4	0.09537	0.06296	0.1349	0.04508	0.02461	0.07314	0.3102	0.149	0.1071	0.1977
KwaZulu-Natal	Umzinyathi(DC24)	4	0.08876	0.04291	0.1587	0.03362	0.0132	0.07021	0.1265	0.1551	0.07914	0.2634
KwaZulu-Natal	Amajuba(DC25)	4	0.08474	0.04995	0.13	0.04012	0.01569	0.08313	0.2337	0.1143	0.07171	0.1671
KwaZulu-Natal	Zululand(DC26)	4	0.08751	0.05018	0.137	0.02945	0.01255	0.05628	0.0531	0.1838	0.1226	0.257
KwaZulu-Natal	Umkhanyakude(DC27)	4	0.07549	0.03499	0.1334	0.03548	0.007931	0.09936	0.201	0.1457	0.08301	0.2255
KwaZulu-Natal	Uthungulu(DC28)	4	0.1133	0.06716	0.1723	0.02959	0.01217	0.05756	0.0595	0.1915	0.1287	0.2654
KwaZulu-Natal	iLembe(DC29)	4	0.07838	0.04204	0.1275	0.02559	0.01013	0.05105	0.0291	0.1555	0.09758	0.2266
Western Cape	Overberg(DC3)	4	0.05185	0.02124	0.09931	0.03229	0.01083	0.06888	0.121	0.2068	0.1281	0.3012
Mpumalanga	Gert Sibande(DC30)	4	0.1043	0.06356	0.1574	0.05363	0.02795	0.09083	0.5413	0.1444	0.0936	0.2071
Mpumalanga	Nkangala(DC31)	4	0.05487	0.02938	0.09093	0.05037	0.02271	0.09553	0.4369	0.1337	0.08241	0.1993
Mpumalanga	Ehlanzeni(DC32)	4	0.07037	0.03825	0.1135	0.04061	0.01924	0.07247	0.2207	0.125	0.07788	0.1838
Limpopo	Mopani(DC33)	4	0.08139	0.04131	0.1371	0.0522	0.02285	0.09752	0.4876	0.09153	0.04813	0.1507
Limpopo	Vhembe(DC34)	4	0.136	0.07485	0.2154	0.0229	0.006895	0.05217	0.0307	0.06845	0.0308	0.1226
Limpopo	Capricorn(DC35)	4	0.1021	0.0564	0.1629	0.03331	0.01386	0.06439	0.1071	0.1062	0.05974	0.1679
Limpopo	Waterberg(DC36)	4	0.07594	0.0438	0.119	0.03911	0.01884	0.06888	0.1829	0.07391	0.04271	0.1157
North West	Bojanala(DC37)	4	0.07324	0.03805	0.1231	0.04879	0.01852	0.1018	0.3996	0.08255	0.0442	0.1354
North West	Ngaka Modiri Molema(DC38)	4	0.08968	0.04975	0.1432	0.06858	0.03463	0.1169	0.8085	0.1027	0.0589	0.1597
North West	Dr Ruth Segomotsi Mompati(DC39)	4	0.08981	0.0507	0.1417	0.06858	0.03573	0.1152	0.8171	0.05378	0.02783	0.09063
Western Cape	Eden(DC4)	4	0.06746	0.02562	0.1406	0.06725	0.02077	0.1613	0.6227	0.1326	0.0657	0.2266
North West	Dr Kenneth Kaunda(DC40)	4	0.07375	0.0384	0.1247	0.05763	0.0269	0.1057	0.5973	0.08794	0.04717	0.1453
Gauteng	Sedibeng(DC42)	4	0.0667	0.03669	0.1086	0.04837	0.02411	0.08407	0.404	0.1202	0.07249	0.1817
KwaZulu-Natal	Sisonke(DC43)	4	0.07345	0.03796	0.1223	0.02278	0.008378	0.04704	0.0171	0.09571	0.05265	0.152
Eastern Cape	Alfred Nzo(DC44)	4	0.1462	0.08241	0.2285	0.02308	0.008373	0.04881	0.0215	0.133	0.07426	0.2097
Northern Cape	John Taolo Gaetsewe(DC45)	4	0.1016	0.05288	0.1678	0.08257	0.03947	0.1444	0.908	0.05625	0.02532	0.1022
Limpopo	Greater Sekhukhune(DC47)	4	0.1042	0.06319	0.1569	0.0478	0.02407	0.08215	0.3925	0.06652	0.03735	0.1057
Gauteng	West Rand(DC48)	4	0.05351	0.02476	0.09764	0.04323	0.01775	0.08442	0.2941	0.102	0.05209	0.1728
Western Cape	Central Karoo(DC5)	4	0.07641	0.03834	0.1311	0.04408	0.01875	0.08464	0.3085	0.09028	0.04692	0.15
Northern Cape	Namakwa(DC6)	4	0.06789	0.03284	0.1195	0.06312	0.02825	0.1166	0.6835	0.07444	0.03661	0.1288
Northern Cape	Pixley ka Seme(DC7)	4	0.09222	0.0517	0.148	0.05126	0.02437	0.09368	0.461	0.1182	0.06756	0.1862
Northern Cape	Siyanda(DC8)	4	0.09641	0.05257	0.1551	0.07439	0.03692	0.1278	0.8633	0.04772	0.02213	0.08534

For peer review only

Northern Cape	Frances Baard(DC9)	4	0.1118	0.06732	0.1696	0.0418	0.01993	0.07405	0.2446	0.06456	0.03501	0.1053
Gauteng	Ekurhuleni(EKU)	4	0.03506	0.01562	0.06488	0.06292	0.03084	0.1093	0.7175	0.0923	0.05138	0.1467
KwaZulu-Natal	eThekwini(ETH)	4	0.1238	0.07914	0.179	0.01876	0.007401	0.03737	0.0025	0.2876	0.2172	0.3641
Free State	Mangaung(MAN)	5	0.1069	0.04453	0.2024	0.0437	0.01171	0.1124	0.3047	0.1709	0.07981	0.2987
Eastern Cape	Nelson Mandela Bay(NMA)	5	0.09343	0.03104	0.1916	0.02627	0.003955	0.07768	0.1082	0.1705	0.07581	0.2982
Gauteng	City of Tshwane(TSH)	5	0.05958	0.03037	0.1013	0.03678	0.0165	0.0685	0.1517	0.08472	0.0465	0.1366
Gauteng	City of Johannesburg(JHB)	5	0.0661	0.03092	0.1179	0.05088	0.02127	0.09775	0.4556	0.09294	0.04727	0.1566
Eastern Cape	Buffalo City(BUF)	5	0.09476	0.01111	0.3266	0.05182	0.003448	0.2323	0.3254	0.1994	0.05385	0.4304
Western Cape	City of Cape Town(CPT)	5	0.0901	0.03945	0.165	0.07019	0.02612	0.1409	0.7271	0.1801	0.09786	0.2857
Western Cape	West Coast(DC1)	5	0.07424	0.02694	0.1538	0.04857	0.01469	0.1112	0.3902	0.1091	0.04406	0.2102
Eastern Cape	Cacadu(DC10)	5	0.07736	0.03719	0.1368	0.03561	0.0123	0.07905	0.1703	0.1172	0.06046	0.1957
Eastern Cape	Amathole(DC12)	5	0.07544	0.02655	0.1615	0.03551	0.008888	0.09441	0.1953	0.1796	0.07963	0.3242
Eastern Cape	Chris Hani(DC13)	5	0.06621	0.03073	0.1193	0.02794	0.01004	0.05946	0.0625	0.1679	0.09502	0.2621
Eastern Cape	Joe Gqabi(DC14)	5	0.07787	0.03574	0.1415	0.02486	0.0085	0.05426	0.038	0.1632	0.08761	0.2637
Eastern Cape	O.R.Tambo(DC15)	5	0.08704	0.04483	0.1456	0.01868	0.005663	0.04195	0.0088	0.1802	0.1114	0.2642
Free State	Xhariep(DC16)	5	0.09556	0.0479	0.1641	0.03589	0.01292	0.07778	0.1673	0.1124	0.05746	0.1901
Free State	Lejweleputswa(DC18)	5	0.1138	0.06199	0.1856	0.04089	0.01768	0.07859	0.2391	0.1099	0.05962	0.1789
Free State	Thabo Mofutsanyane(DC19)	5	0.09437	0.04871	0.1608	0.03939	0.01565	0.082	0.2159	0.1264	0.06736	0.2088
Western Cape	Cape Winelands(DC2)	5	0.0925	0.04628	0.1591	0.0541	0.02154	0.1071	0.5056	0.1108	0.05848	0.1826
Free State	Fezile Dabi(DC20)	5	0.09295	0.04332	0.1689	0.04421	0.01546	0.09892	0.3122	0.1629	0.08234	0.2761
KwaZulu-Natal	Ugu(DC21)	5	0.06515	0.03118	0.1141	0.0291	0.01078	0.06025	0.0689	0.2041	0.1294	0.2942
KwaZulu-Natal	UMgungundlovu(DC22)	5	0.06007	0.02975	0.1049	0.03159	0.01277	0.06323	0.0896	0.1687	0.1008	0.255
KwaZulu-Natal	Uthukela(DC23)	5	0.07366	0.0369	0.1261	0.03635	0.01498	0.07121	0.1587	0.1181	0.06564	0.1869
KwaZulu-Natal	Umzinyathi(DC24)	5	0.06816	0.03548	0.1145	0.03264	0.01343	0.06394	0.1007	0.1344	0.07878	0.2062
KwaZulu-Natal	Amajuba(DC25)	5	0.08702	0.04654	0.1428	0.03216	0.01317	0.06332	0.0924	0.115	0.06492	0.1807
KwaZulu-Natal	Zululand(DC26)	5	0.08179	0.04205	0.1385	0.02757	0.01073	0.05563	0.0468	0.1585	0.09278	0.2425
KwaZulu-Natal	Umkhanyakude(DC27)	5	0.08356	0.0337	0.16	0.04427	0.01307	0.1007	0.3307	0.1108	0.04926	0.1988
KwaZulu-Natal	Uthungulu(DC28)	5	0.1054	0.05513	0.1743	0.03221	0.01203	0.0662	0.1086	0.1777	0.1062	0.2671
KwaZulu-Natal	iLembe(DC29)	5	0.06697	0.03073	0.1211	0.03029	0.009424	0.07162	0.1084	0.163	0.09109	0.2561
Western Cape	Overberg(DC3)	5	0.09568	0.04146	0.1769	0.04942	0.01204	0.1302	0.3803	0.2146	0.1186	0.3356
Mpumalanga	Gert Sibande(DC30)	5	0.08942	0.04921	0.1451	0.03912	0.01816	0.07228	0.1947	0.129	0.07546	0.1993
Mpumalanga	Nkangala(DC31)	5	0.07231	0.03739	0.1233	0.04704	0.02126	0.08835	0.3698	0.1076	0.05845	0.1752
Mpumalanga	Ehlanzeni(DC32)	5	0.07384	0.03625	0.1281	0.03836	0.01564	0.07516	0.2001	0.07158	0.03446	0.1248
Limpopo	Mopani(DC33)	5	0.1004	0.04689	0.1784	0.05964	0.02286	0.1209	0.5918	0.08324	0.03695	0.1522
Limpopo	Vhembe(DC34)	5	0.1096	0.04638	0.202	0.04795	0.01387	0.1095	0.3858	0.08445	0.03257	0.1648
Limpopo	Capricorn(DC35)	5	0.1135	0.05661	0.1935	0.04223	0.01582	0.08683	0.279	0.1163	0.05806	0.1971
Limpopo	Waterberg(DC36)	5	0.06451	0.03326	0.1091	0.03943	0.01745	0.07391	0.2079	0.1024	0.05697	0.1639
North West	Bojanala(DC37)	5	0.0774	0.03672	0.1391	0.04117	0.01433	0.09099	0.2626	0.1169	0.05926	0.1986
North West	Ngaka Modiri Molema(DC38)	5	0.07094	0.03304	0.1271	0.04752	0.01927	0.09351	0.3857	0.1055	0.05326	0.1784
North West	Dr Ruth Segomotsi Mompati(DC39)	5	0.07974	0.03953	0.1378	0.05313	0.02293	0.1008	0.4994	0.07539	0.03659	0.132
Western Cape	Eden(DC4)	5	0.07396	0.03057	0.1424	0.06293	0.01782	0.1599	0.5534	0.1085	0.04862	0.1979
North West	Dr Kenneth Kaunda(DC40)	5	0.09486	0.04913	0.1616	0.04531	0.0195	0.08782	0.3321	0.1054	0.05508	0.1771
Gauteng	Sedibeng(DC42)	5	0.06668	0.03408	0.1142	0.04831	0.02213	0.08981	0.4012	0.1106	0.06083	0.1787
KwaZulu-Natal	Sisonke(DC43)	5	0.06397	0.02633	0.1234	0.02421	0.007352	0.05607	0.0432	0.2003	0.1123	0.3113
Eastern Cape	Alfred Nzo(DC44)	5	0.06469	0.02775	0.1226	0.02584	0.00854	0.0577	0.0509	0.1635	0.0862	0.2667
Northern Cape	John Taolo Gaetsewe(DC45)	5	0.08431	0.03505	0.1603	0.0562	0.01981	0.1194	0.5218	0.08058	0.03258	0.1563

Limpopo	Greater Sekhukhune(DC47)	5	0.07424	0.03669	0.1286	0.04955	0.02151	0.09391	0.4254	0.06453	0.03101	0.1137
Gauteng	West Rand(DC48)	5	0.0625	0.02798	0.117	0.03643	0.01352	0.07603	0.1751	0.1122	0.05466	0.1958
Western Cape	Central Karoo(DC5)	5	0.0802	0.03706	0.1455	0.05129	0.0196	0.1052	0.448	0.09237	0.04354	0.164
Northern Cape	Namakwa(DC6)	5	0.09181	0.03883	0.1782	0.05104	0.01953	0.1054	0.4406	0.0862	0.03898	0.1578
Northern Cape	Pixley ka Seme(DC7)	5	0.08998	0.04711	0.1524	0.04131	0.01733	0.08273	0.2484	0.1102	0.0568	0.1881
Northern Cape	Siyanda(DC8)	5	0.1188	0.06163	0.1973	0.04994	0.02016	0.09848	0.4293	0.06679	0.02976	0.1224
Northern Cape	Frances Baard(DC9)	5	0.1008	0.05443	0.1649	0.03683	0.01559	0.07081	0.1581	0.07535	0.03818	0.1285
Gauteng	Ekurhuleni(EKU)	5	0.05108	0.02232	0.09517	0.05776	0.02487	0.109	0.5892	0.1092	0.05715	0.1803
KwaZulu-Natal	eThekwini(ETH)	5	0.06257	0.02875	0.1122	0.02369	0.007809	0.0518	0.0304	0.1526	0.08896	0.2329

Supplementary 8: post hoc power analysis

We performed a post hoc power analysis to assess the minimum effect size detectable among infants which has the smallest number of observations. The post hoc power analysis suggests that the sample size in the smallest age group has the power to detect a small effect size (w~0.1 based on Cohens rules of thumb [Cohen, 1988]) when using a chi-square test with 2x9 cells (maximum number of cells tested in our analyses i.e. binary nutritional classification versus province of residence) with 80% power and 5% alpha or type I error.

χ^2 tests - Goodness-of-fit tests: Contingency tables

**Analysis:**	Post hoc: Compute achieved power		
**Input:**	Effect size w	=	0.11
	α err prob	=	0.05
	Total sample size	=	1277
	Df	=	8
**Output:**	Noncentrality parameter λ	=	15.4517000
	Critical χ^2	=	15.5073131
	Power (1-β err prob)	=	0.8133607

Cohen, J (1988) Statistical power analysis for the behavioral sciences (2nd ed.). Hillsdale, NJ: Erlbaum.

A summary guideline for effect size determinations is also provided in Kotrlik, JW and Williams, HA (2003) The incorporation of effect size in information technology, learning, and performance research. *Information Techology, Learning, and Performance Journal* **21(1)** 1-7.

**Effect Size**	**Use**	**Small**	**Medium**	**Large**
Correlation inc Phi		0.1	0.3	0.5
Cramer's V	r x c frequency tables	0.1	0.3	0.5
Difference in arcsines	Comparing two proportions	0.2	0.5	0.8
η^2	Anova	0.01	0.06	0.14
omega-squared	Anova; See Field (2013)	0.01	0.06	0.14
Multivariate eta-squared	one-way MANOVA	0.01	0.06	0.14
Cohen's f	one-way an(c)ova (regression)	0.1	0.25	0.4
η^2	Multiple regression	0.02	0.13	0.26
κ^2	Mediation analysis	0.01	0.09	0.25
Cohen's f	Multiple Regression	0.14	0.39	0.59
Cohen's d	t-tests	0.2	0.5	0.8
Cohen's ω	chi-square	0.1	0.3	0.5
Odds Ratios	2 by 2 tables	1.5	3.5	9
Odds Ratios	p vs 0.5	0.55	0.65	0.75
Average Spearman rho	Friedman test	0.1	0.3	0.5

For peer review only

BMJ Open

Spatial-temporal trends and risk factors for under-nutrition and obesity among children (<5 years) in South Africa, 2008-2017: findings from a nationally representative longitudinal panel survey

Journal:	*BMJ Open*
Manuscript ID	bmjopen-2019-034476.R2
Article Type:	Original research
Date Submitted by the Author:	13-Mar-2020
Complete List of Authors:	Sartorius, Benn; London School of Hygiene and Tropical Medicine Faculty of Infectious and Tropical Diseases, Disease Control<br>Sartorius, Kurt; University of KwaZulu-Natal, Public Health Medicine, School of Nursing and Public Health<br>Green, Rosemary; London School of Hygiene and Tropical Medicine, 2 Department of Population Health<br>Lutge, Elizabeth; South African Government Department, KwaZulu Natal Department of Health<br>Scheelbeek, Pauline; London School of Hygiene & Tropical Medicine, Epidemiology and Population Health<br>Tanser, Frank; Africa Centre for Health and Population Studies; University of KwaZulu-Natal<br>Dangour, Alan; London School of Hygiene & Tropical Medicine, Dept of Population Health<br>Slotow, Rob; University of KwaZulu-Natal
**Primary Subject Heading**:	Nutrition and metabolism
Secondary Subject Heading:	Epidemiology, Public health
Keywords:	PUBLIC HEALTH, Community child health < PAEDIATRICS, NUTRITION & DIETETICS

SCHOLARONE™
Manuscripts

I, the Submitting Author has the right to grant and does grant on behalf of all authors of the Work (as defined in the below author licence), an exclusive licence and/or a non-exclusive licence for contributions from authors who are: i) UK Crown employees; ii) where BMJ has agreed a CC-BY licence shall apply, and/or iii) in accordance with the terms applicable for US Federal Government officers or employees acting as part of their official duties; on a worldwide, perpetual, irrevocable, royalty-free basis to BMJ Publishing Group Ltd ("BMJ") its licensees and where the relevant Journal is co-owned by BMJ to the co-owners of the Journal, to publish the Work in this journal and any other BMJ products and to exploit all rights, as set out in our licence.

The Submitting Author accepts and understands that any supply made under these terms is made by BMJ to the Submitting Author unless you are acting as an employee on behalf of your employer or a postgraduate student of an affiliated institution which is paying any applicable article publishing charge ("APC") for Open Access articles. Where the Submitting Author wishes to make the Work available on an Open Access basis (and intends to pay the relevant APC), the terms of reuse of such Open Access shall be governed by a Creative Commons licence – details of these licences and which Creative Commons licence will apply to this Work are set out in our licence referred to above.

Other than as permitted in any relevant BMJ Author's Self Archiving Policies, I confirm this Work has not been accepted for publication elsewhere, is not being considered for publication elsewhere and does not duplicate material already published. I confirm all authors consent to publication of this Work and authorise the granting of this licence.

For peer review only

Title page

Spatial-temporal trends and risk factors for under-nutrition and obesity among children (<5 years) in South Africa, 2008-2017: findings from a nationally representative longitudinal panel survey

Running title: under-nutrition and obesity among children in South Africa

Sartorius B[1-3]*, Sartorius K[2,4], Green R[5], Lutge E[2,6], Scheelbeek P[5], Tanser F[2,7-9], Dangour AD[5], Slotow R[10,11]

1. Department of Disease Control, London School of Hygiene & Tropical Medicine, London, UK
2. Department of Public Health Medicine, School of Nursing and Public Health, University of KwaZulu-Natal, Durban, South Africa
3. Department of Health Metrics Sciences, School of Medicine, University of Washington, Seattle, USA
4. Faculty of Commerce, Law and Management, University of the Witwatersrand, Johannesburg, South Africa
5. Department of Population Health, London School of Hygiene & Tropical Medicine, London, UK
6. Health Research and Knowledge Management, KwaZulu-Natal Department of Health, KwaZulu-Natal, South Africa
7. Africa Health Research Institute (AHRI), KwaZulu-Natal, South Africa
8. Centre for the AIDS Programme of Research in South Africa (CAPRISA), University of KwaZulu-Natal, Durban, South Africa
9. Institute of Epidemiology and Health Care, University College London, London, United Kingdom
10. School of Life Sciences, University of Kwazulu-Natal, Pietermaritzburg Campus, South Africa
11. Department of Genetics, Evolution and Environment, University College, London, UK

*Corresponding author: Dr Benn Sartorius; Address: Department of Disease Control, London School of Hygiene and Tropical Medicine, Keppel Street, Bloomsbury, London, WC1E7HT, United Kingdom; Tel.: +44 (0)207 927 2164; E-mail: benn.sartorius1@lshtm.ac.uk

Word count: 4095

For peer review only

Summary

Objectives: To assess space-time trends in malnutrition and associated risk factors among children (<5 years) in South Africa

Design: multi-round national panel survey using multistage random sampling

Setting: national, community-based

Participants: Community-based sample of children and adults. Sample size: 3,254 children in wave 1 (2008) to 4,710 children in wave 5 (2017).

Primary outcomes: Stunting, wasting/thinness and obesity among children (<5). Classification were based on anthropometric (height and weight) z-scores using WHO growth standards.

Results: Between 2008 and 2017 a larger decline nationally in stunting among children (<5) was observed from 11.0% to 7.6% (p=0.007), compared to thinness/wasting (5.2% to 3.8%, p=0.131) and obesity (14.5% to 12.9%, p=0.312). A geographic nutritional gradient was observed with obesity more pronounced in the east of the country and thinness/wasting more pronounced in the west. Approximately 73% of districts had an estimated wasting prevalence below the 2025 target threshold of 5% in 2017 while 83% and 88% of districts achieved the necessary relative reduction in stunting and no increase in obesity respectively from 2012 to 2017 in line with 2025 targets. African ethnicity, male gender, low birth weight, lower socio-economic and maternal/paternal education status and rural residence were significantly associated with stunting. Children in lower income and food insecure households with young malnourished mothers were significantly more likely to be thin/wasted while African children, with higher birth weights, living in lower income households in KwaZulu-Natal and Eastern Cape were significantly more likely to be obese.

Conclusions: While improvements in stunting have been observed, thinness/wasting and obesity prevalence remain largely unchanged. The geographic and socio-demographic heterogeneity in childhood malnutrition has implications for equitable attainment of global nutritional targets for 2025, with many districts having dual epidemics of under- and over-nutrition. Effective sub-national level public health planning and tailored interventions are required to address this challenge.

Keywords: nutritional status, nutritional transition, undernutrition, obesity, children, South Africa

Strengths and limitations of this study

For peer review only

- Utilises data from a nationally representative repeated panel data at individual/household level over a 10-year period (5 survey waves).
- Employed a fully Bayesian space-time shared component model to produce more stable estimates of malnutrition burden at provincial and district level among children under five years of age in South Africa.
- Panel design allows assessment of change in malnutrition burden within the same individuals/households observed at multiple time points.
- Missing or invalid weight/height measurements may have introduced selection bias if not missing at random, and may thus have affected both the internal validity and the representativeness the findings.
- As primary panel study was not designed/powered for provincial and lower geographic level analysis, we cannot discount the resultant impact on precision/random variability when analysing at provincial/district level (administrative tier just below province) and further stratification by socio-demographic correlates.

For peer review only

Background

Despite reductions in malnutrition 150.8 million children (22.2%) under five are stunted and a further 50.5 million children are wasted [1]. Furthermore rapidly rising trend in overweight and obesity in children and adults [2-4] [5] has emerged as one of the most serious global public health issues of the 21st century [6]. Sub-Saharan Africa (SSA) has among the highest levels of child malnutrition[1] globally. This problem is particularly illustrated by South Africa [7], a middle income country with high levels of wealth/economic inequality that is undergoing rapid socioeconomic and lifestyle changes that have precipitated a nutritional transition, high prevalence of overweight/obesity in children [8]. The dual burdens of undernutrition and overweight/obesity are not distributed in a spatially homogenous manner [9], and the health risks associated with malnutrition vary by age, gender, ethnicity and geographical location [10].

Progress to tackle all forms of child malnutrition remain much too slow [1]. In order to support the delivery of public health interventions that will be most effective at reducing malnutrition, an understanding of the geographical distribution of malnutrition is required. Limited data are collected at lower administrative unit level making it difficult to identify specific groups of high-risk individuals and thus, determine the most suitable and

[1] Child malnutrition is defined as a pathological state as a result of inadequate nutrition, including undernutrition due to insufficient intake of dietary energy and other key nutrients resulting in stunting (low height for age) or wasting (low weight-for-length) and overweight and obesity due to excessive consumption of dietary energy and reduced levels of physical activity.

For peer review only - http://bmjopen.bmj.com/site/about/guidelines.xhtml

cost-effective opportunities and solutions. Previous studies of nutritional status of the South African population have mostly focused on adults [11 12]. Here we use a large, nationally-representative data from multiple rounds of the National Income Dynamics Study over the period 2008 to 2017 to assess space-time trends in the burden of malnutrition and associated risk factors among children under 5 years of age in South Africa.

Methods

We include a Strengthening the Reporting of Observational studies in Epidemiology (STROBE) statement [13] checklist in Supplementary Material 1.

Data

Data were taken from the five panel (cross-sectional) waves of the South African National Income Dynamics Study (SA-NIDS)[14 15] (http://www.nids.uct.ac.za/nids-data/data-access; https://www.datafirst.uct.ac.za/dataportal/index.php/catalog/NIDS/), the first national panel study in South Africa. SA-NIDS was undertaken by the South African Labour and Development Research Unit based at the School of Economics at the University of Cape Town. The surveys took place in 2008, 2010-11, 2012, 2014-15 and 2017. These are named waves 1-5 respectively. A detailed description of the data collection methods can be found elsewhere [14]. In short, a stratified, two-stage random cluster sample design was employed to sample households for inclusion at baseline using proportionally allocated stratification, based on the 52 district councils (DCs) in South Africa[14]. Within each DC (primary sampling unit [PSU]), clusters of dwelling units were systematically drawn. The household level response rate was 69% and the individual response rate within households was 93%. Survey enumerators attempted to collect weight and height measurements of all individuals (including children) in selected households.

For peer review only

Study population

We restricted our analysis to children <5 years of age.

Outcomes

We calculated height for age (HA) and BMI-for-age (BA) z-scores using the WHO 2007 growth standards [16 17]. We generated z-scores by transformation of child anthropometric data using the “lambda mu sigma” method (‘zanthro’ function in Stata 15). As recommended, weight-for-length was used in children 0 to <2 years of age, and BMI-for-age in children 2 years of age and older [18]. We defined obesity as weight-for-length z-score ≥+2 for

For peer review only - http://bmjopen.bmj.com/site/about/guidelines.xhtml

children under 2 years of age and BMI for age z-score of >2+ for children age 2 and older [18]. We defined wasting as weight-for-length z-score < -2 for children under 2 years of age and thinness as BMI for age z-score < -2 for children 2 years and older. Stunting was defined as HA z-score of < -2.

Geographic and socio-demographic variables

To identify relevant inequalities under-nutrition and obesity indicators were stratified temporally (survey year), geographically (province and residence location type: urban informal settlements, urban formal, tribal/rural) and by important socio-demographic categories (Gender: Female/Male; ethnicity: Black/African, Coloured, Indian/Asian, White/Caucasian; Maternal: age; education status; body mass index; household socio-economic status (income) classified into quantiles [1=lowest, 5=highest].

Data analysis

Analyses were performed using Stata software version 15 [StataCorp. 2017. Stata Statistical Software: Release 15. College Station, TX: StataCorp LLC]. Given the multistage random sampling design of the primary study, clustering and survey design effects were accounted for using sample weights to estimate standard error and 95% confidence intervals (CIs) around mean anthropometric z-score point estimates, both overall and stratified by other socio-demographic variables such ethnicity and gender, socio-economic status, and residence location type. Extrapolated population totals of malnourished children (< 5) by yearly age were estimated using the survey weights.

Space-time Bayesian modelling: We assessed for the presence of univariate and bivariate spatial autocorrelation for the three anthropometric classifications using Moran's I statistics. This analysis was performed using GeoDa [19]. Based on these tests it appeared that there was no prominent bivariate spatial autocorrelation between the three measures but that each measure was significantly heterogeneous across space , warranting the use of a separate spatial-temporal model for each nutritional outcome. These additional analyses are presented in Supplementary Material 2.

We employed Bayesian spatial-temporal modelling approach in an attempt to stabilise estimates at district level given that the primary sampling design was not developed to provide point estimates at this level of geographic disaggregation and resultant zero prevalence estimates for particular districts and waves. We choose a Bayesian spatial-temporal formulation to model each of the anthropometric outcomes independently using an autoregressive approach. We employed a Bayesian hierarchical binomial model that simultaneously attempts to estimate the

For peer review only - http://bmjopen.bmj.com/site/about/guidelines.xhtml

stable spatial and temporal structured patterns and as well as from these stable components using an unstructured space-time interaction term [20].

Let Y_{1ij}, Y_{2ij} and Y_{3ij} be the number of stunted, thin and obese children respectively for the ith area and jth period, i =1,...,I, j =1,...,J, and n_{ij} the total number of children sampled in a given area and period. We assumed that Y_{1ij}, Y_{2ij} and Y_{3ij} follow binomial distributions i.e. $Y_{1ij} \sim$ binomial (n_{1ij}, π_{1ij}), $Y_{2ij} \sim$ binomial (n_{2ij}, π_{2ij}), $Y_{3ij} \sim$ binomial (n_{3ij}, π_{3ij}), i =1,...,53, j =1,..., 5, where π it is the risk (prevalence) of stunting, thinness or obesity in region i in period j. We define the logit of the prevalence for a given anthropometric outcome as follows:

$$\text{logit}(\pi_{1ij}) = \alpha_1 + \phi_{1i} + \gamma_{1j} + \nu_{1ij}$$

$$\text{logit}(\pi_{2ij}) = \alpha_2 + \phi_{2i} + \gamma_{2j} + \nu_{2ij}$$

$$\text{logit}(\pi_{3ij}) = \alpha_3 + \phi_{3i} + \gamma_{3j} + \nu_{3ij}$$

$$\nu \sim \text{Normal}(0,\ \sigma^2_\nu),\ i = 1, \ldots, I \text{ and } j=1,\ldots,J$$

$$\phi \sim \text{CAR.normal}(\sigma^2_\phi),\ \text{for } i=1,\ldots,I$$

$$\gamma = (\gamma_1, \gamma_2, \ldots, \gamma_J) \sim \text{CAR.normal}(\sigma^2_\gamma)\alpha \sim \text{Uniform}(-\infty, +\infty)$$

where $\alpha_{1\text{-}3}$ are the overall baseline risk (intercept) for each nutritional outcome, $\phi_{1\text{-}3}$, the spatial random effects, assume intrinsic Gaussian conditionally autoregressive distributions [21] (abbreviated above as CAR.normal), whereby the spatially correlated random effect of the ith region (φ_i) is based on the sum of its weighted neighbourhood values. We used an adjacency matrix of common boundaries (neighbours) of a given region when modelling this parameter. The CAR approach can also be used to model the temporal random effects. A first-order (pre and post) random walk CAR.normal, utilising a period adjacency matrix, was used as prior distributions for the temporal random effects, $\gamma_{1\text{-}3}$. The heterogeneous or unstructured random effects are represented by $\nu_{1\text{-}3}$ and were included to ensure sufficient flexibility for estimates in close regions that is not captured by the spatially structured terms. We assumed uniform priors for the model intercepts to ensure model identifiability. As the CAR.normal distribution is parameterised to include a sum-to-zero constraint on the random effects, we thus included a separate intercept term, α, in each model, which were assigned improper uniform priors (on the whole real line) using the dflat() distribution function in WinBUGS. We chose inverse gamma distributions for the variance parameters above with values of 0.5 and 0.0005 as suggested by Wakefield et al [22]::

$$\sigma^2_\nu,\ \sigma^2_\phi,\ \sigma^2_\gamma \sim \text{Gamma}(0.5, 0.0005)$$

For peer review only - http://bmjopen.bmj.com/site/about/guidelines.xhtml

163 To aid interpretation of prevalence point estimates in line with WHO 2025 nutritional targets we also estimated exceedance probabilities associated with the target thresholds for each nutritional outcome, namely: 40% reduction in stunting from 2012 to 2015, reduce and maintain wasting to <5% by 2025 and no increase in obesity by 2025 [23]. We employed Richardson's criterion, in which probabilities in excess of 0.8 were deemed to be significant [24].

168 Survey weighted prevalence's were applied to sample size totals by district and panel to obtain a survey weighted numerator counts for each outcome (Y_{1ij}, Y_{2ij}, Y_{3ij} above) from the binomial distribution. The space-time models were fitted in WINBUGS using Markov chain Monte Carlo (MCMC) simulation and non-informative priors. The full WINBUGS model code is provided in the Supplementary Material (3). A summary of the space-time random effect posteriors is presented in Supplementary 4. Sensitivity of the estimates to prior specification was assessed by repeating the analysis with different hyper parameters (Supplementary 4). We used two-chain MCMC simulation for parameter estimation, a burn-in of 10000 iterations, and Gelman-Rubin statistics/plots [25] were used to assess model convergence/stability and where the Monte Carlo error for each parameter of interest was less than 5% of the sample standard deviation (Supplementary Material 5). For model validation, we firstly compared the observed and fitted prevalence values to assess overall model adequacy and fit (using model Deviance Information Criterion [DIC] and comparison of observed vs fitted prevalence estimate) and secondly, performed an out of sample validation using a random 10% sample with observed data (Supplementary Material 6). The model was run until the Monte Carlo error for each parameter of interest was < 5% of the sample standard deviation. Posterior prevalence estimates and 95% Bayesian credibility intervals for stunting, thinness/wasting and obesity at provincial and district level were mapped using ArcGIS 10.6.1 [ESRI 2011. ArcGIS Desktop: Release 10. Redlands, CA: Environmental Systems Research Institute].

184 *Risk factors analysis*: Survey weighed two-way tabulations of key socio-demographic covariates, year and child nutritional status were performed to produce correctly weighted prevalence estimates. Tests of independence for complex survey data survey (weighted Pearson's chi-square test) was utilised to assess the significance of bivariate associations between malnutrition burden and year as well as socio-demographic covariates.

188 **Ethical approval**: Approval for the primary study was granted by the Ethics Committee of the University of Cape Town. The current study is a secondary data analysis of an open access dataset and does not require further ethical approval.

For peer review only - http://bmjopen.bmj.com/site/about/guidelines.xhtml

Patient and Public Involvement: As this was a data analysis utilising secondary data from a national community based panel survey, the development of the research question was not informed by the study subjects. Likewise, we could not involve study participants in the design of this study. Study participants were not involved in conduct of the primary study. Results will be disseminated in the form of peer reviewed article as well as through presentation to senior members of our National Department of Health and KwaZulu-Natal Department of Health.

Results

Study population

The sample of children <5 years of age in the 7,301 households included in the SA-NIDS survey increased from 3,254 children at baseline (2008) to 4,710 children in wave 5 (2017) (Supplementary Material 7). With the exception of children under 1 year of age and survey wave 2 in 2010/11, valid weight and height measurements were taken from 85-90% of children sampled between the age of 1 and 5 on average (Supplementary Material 7). An additional sensitivity analysis comparing distributions of various socio-demographic characteristics by missing weight/height status was also performed (Supplementary Section 8). These findings suggest that children with missing weight/height were largely missing at random, with the exception of age and province. A summary of the characteristics of the study sample by year can be found in Table 1.

Temporal changes in burden of malnutrition from 2008 to 2017)

Between 2008 and 2017, the prevalence of stunting among children aged under 5 years decreased from 11.0% to 7.6% (p=0.007) (Table 2). Over the same period, both the prevalence of wasting/thinness (and the prevalence of obesity decreased (from 5.2 to 3.8%, p= 0.131 and 14.5% to 12.9%, p= 0.312 respectively). The prevalence of thinness was higher (p<0.001) in children under 2 years of age (8% [95%CI: 5.0-11.8%] in 2008; 6% [95%CI: 4.1-9.1%] in 2017) compared to 4% (95%CI: 3.2-6.2%) in 2008 and 3% (95%CI: 2.0-4.5%) in 2017 among children 2 years and older . The prevalence of obesity was also higher among children under 2 years of age and increased over the study period (18.4% [95%CI: 13.7-24.1%] in 2008 vs 21.7% [95%CI: 19.3-24.2%]in 2017, p=0.091).

Space-time burden of malnutrition at provincial and district level

Under nutrition: In 2008, the highest prevalence of stunting was estimated in the Free State (18% .1followed by Eastern Cape (14.8%) and Limpopo (14.0%) . By 2017 the highest prevalence of stunting was still observed in

For peer review only

For peer review only - http://bmjopen.bmj.com/site/about/guidelines.xhtml

Free State (10%) followed by Northern Cape (9.6%) and Limpopo (8.5%) (Figure 1 – panel a1). One district in Free State (Lejweleputswa), 2 in Limpopo (Capricorn; Mopani) and one each in Northern Cape (Siyanda), North West (Dr Kenneth Kaunda), Eastern Cape (O.R.Tambo) and KwaZulu-Natal (Uthungulu) had a posterior median smoothed prevalence of stunting in excess of 10% in 2017 (Figure 1– panel a2, Supplementary 9). Forty-three (or 83%) of districts achieved a 17% reduction (necessary reduction over the period to achieve 40% reduction from 2012 to 2025) in stunting prevalence from 2012 to 2017. Of these 43 districts, 19 (or 44%) significantly achieved this threshold based on exceedance probability (p>0.80).

North West province had the highest burden of thinness/wasting in 2008 (10.1%) followed by Gauteng (9.5%) and Western Cape (8.2%) (Figure 2a). By 2017, the highest burden was observed in Western Cape (at 5.8%) followed by Northern West (5.0%) and North Cape (4.9%) (Figure 2b) i.e.2 of 9 provinces were still above the 5% target threshold for wasting in 2017. There appeared to be a general gradient of higher burden of thinness/wasting in the western half of country in 2017 (lower burden in KwaZulu-Natal and northern districts of Eastern Cape) (Figure 2b). Our estimates suggest that 38/52 (or 73%) districts in 2017 were below the 5% target prevalence threshold compared to 21/52 (or 40%) in 2012. Based on exceedance probability associated with the 5% target threshold, approximately half (or 18/38) of the aforementioned districts with an estimated thinness/wasting prevalence below 5% in 2017 where below this threshold with high probability (exceedance p>0.8) (Supplementary 9). Three of the five districts with the highest posterior median smoothed prevalence of wasting in 2017 were located in Western Cape (City of Cape Town [6.8%]; Central Karoo [6.4%]; Eden [6.1%]) with the remaining two in the top five located in Eastern Cape (Buffalo City [7.9%]) and Gauteng (Sedibeng [6.6%]) (Supplementary 9).

Obesity: In 2008, the highest posterior median smoothed prevalence of obesity was estimated in Eastern Cape (22.5%) followed by KwaZulu-Natal (18.3%) and Western Cape (18.1%) (Figure 3a). A decade later in 2017, the highest prevalence of childhood obesity was still estimated to be in the Eastern Cape (16.7%), followed by KwaZulu-Natal (15.6%)and Western Cape (15.0%). Six districts had an increase in obesity from 2012 to 2017, namely: 3 in Limpopo (Capricorn, Vhembe, Waterberg), 1 in Free State (Mangaung), 1 in Eastern Cape (Amathole) and 1 in North West (Bojanala) (Supplementary 9). In contrast to the wasting gradient highlighted above (higher burden in the western half of the country), the burden of obesity in 2017 appeared to be much higher in the eastern half of the country (particularly KwaZulu-Natal and Eastern Cape) (Figure 3b), with the exception of certain districts in Western Cape. Eight of the tope 10 highest obesity prevalence districts in 2017 were located in KwaZulu-Natal (Sisonke [21.4%], Ugu [20.8%], Uthungulu [18.6%] and iLembe [18.0%) and Eastern Cape (Buffalo City Metropolitan [22.8%], Amathole [19.6%], Chris Hani [18.5%[O.R Tambo [17.9%]). The other two

For peer review only - http://bmjopen.bmj.com/site/about/guidelines.xhtml

For peer review only

districts in the 10 highest obesity prevalence districts in 2017 were located in Western Cape (Overberg [22.0%] and City of Cape Town [18.5%]) (Supplementary 9).

Figure 1: Bayesian posterior median smoothed prevalence of stunting by province (and wave) and district level prevalence (equal intervals, 2017) among children <5 years

Figure 2: Bayesian posterior median smoothed prevalence of thinness/wasting by province (and wave) and district level prevalence (equal intervals, 2017) among children <5 years

Figure 3: Bayesian posterior median smoothed prevalence of obesity by province (and wave) and district level prevalence (equal intervals, 2017) among children <5 years

Factors associated with child nutritional status

A bivariate analysis of demographic, maternal, socio-economic and household factors at individual nutritional status level suggests that African ethnicity (p<0.001), male gender (p=0.002), low birth weight (<0.001), residing in lower socio-economic status household (p<0.001), province of residence (p=0.012), lower maternal/paternal education status (p<0.001, 0.020 respectively) and residence in a rural/tribal authority area (p<0.001) were significantly associated with stunting (Table 3). Children living in lower income households (p=0.053), lower food security (as measured through child hunger in last year) (p<0.001), province of residence (p=0.002), having a younger mother (<20) (p=0.012) and mother having a lower BMI classification (p=0.005) was significantly associated with thinness/wasting status. Children of African ethnicity (p<0.001), higher birth weight (p=0.006), living in lower income households (p=0.001) in KwaZulu-Natal and Eastern Cape (p<0.001) as well as paternal educational attainment (p=0.033) were significantly associated with obesity status (Table 3).

Discussion

Main findings: The present study illustrates that while stunting has declined among South African children over the last 10 years, wasting and obesity appear largely unchanged, suggesting that development and public health interventions have had a variable impact. Stunting prevalence appears relatively evenly spread across South Africa, but obesity burden is more pronounced in the east of the country, whereas thinness/wasting is more pronounced in the west. In terms of progress towards WHO 2025 nutritional targets, 14 of 52 (27%) districts had an estimated wasting prevalence still exceeding 5% prevalence in 2017 as well as 17% (9/52) and 12% (6/52) districts not attaining the relative reduction in stunting prevalence required or with an increase in obesity prevalence respectively from 2012 to 2017. A further concerning pattern observed was the increasing prevalence

For peer review only - http://bmjopen.bmj.com/site/about/guidelines.xhtml

of obesity in children under the age of two years. Key socio-demographic factors associated with malnutrition status were identified which likely underpins the spatial patterns (and heterogeneity) observed across the country. African children with lower birth weights residing in lower income households in rural areas with less educated mothers and fathers were particular more likely to be stunted. Children in lower income, food insecure households with malnourished young mothers appeared particularly more likely to be thin/wasted while African children, with higher birth weights, living in lower income households in KwaZulu-Natal and Eastern Cape were also more likely to be obese. Furthermore, low household income appeared to be positively associated with all 3 nutritional types. Declining childhood stunting rates from 2008-2017 may well have resulted from government initiatives to support food security and child health (among other things), but our findings of distinct geographic and socio-demographic variability in undernutrition and obesity rates suggest that tackling malnutrition in South Africa is complex. Models and targets for nationally-driven intervention need to be carefully specified according to local environments and socio-economic profiles.

Contribution to existing literature*:* Two previous studies in South Africa among primary school aged children dating back 25+ years (1993and 1994 respectively) utilised cross sectional data[26 27], thus limiting insight into temporal trends. Furthermore, the study by Jinabhai et al. [19] was restricted to KwaZulu-Natal limiting national representativeness. Another cross sectional study in South African in 2001-2003 among primary school children in five South African Provinces suggested that relative to 1993 prevalence of undernutrition had decreased while obesity had increased[27 28]. Thus these previous data are now outdated, were largely focused on primary school aged children as well as cross sectional in nature and geographically restricted.

This is also the first spatial-temporal Bayesian shared component analysis of malnutrition trends among children in South Africa utilising geographically representative repeated panel data over a 10-year period. The current study focusing on children under 5 year of age suggests that there is prominent geographic heterogeneity in malnutrition burden in South Africa in this youngest age group. This is in line with findings from other settings in Africa that have documented similar spatial heterogeneity [29] and persistence of these malnutrition inequalities has been demonstrated in an 80 country study further highlighting this ongoing public health conundrum [30 31]. Our results demonstrate a strong west to east gradient of higher underweight burden on the western side of South Africa and greater obesity on the eastern seaboard (Eastern Cape and KwaZulu-Natal). A map of poverty and

For peer review only - http://bmjopen.bmj.com/site/about/guidelines.xhtml

inequality in South Africa [2] illustrates the co-existence of high levels of poverty and inequality in many parts of KwaZulu-Natal and the Eastern Cape with high levels of overweight/obesity. This is further confirmed by our individual child level analysis which suggested a significantly higher obesity prevalence in lower income households. Metropolitan areas displayed high levels of nutritional inequality that complement national studies of poverty and inequality [32].

Under and over nutrition status appeared positively associated with lower household income classification. This finding of stunting and wasting disproportionately affecting the poor has been often demonstrated [33]. Other studies in Africa in particular have documented similar patterns i.e. children living in low SES households, children who live in peripheral areas and whose mothers had little or no schooling were at significantly higher risk of malnutrition [34]. The inconsistent challenges facing health authorities are occurring in the face of rapid urbanization and industrialization that simultaneously attract both the rich and the poor to live in the same geographic districts [35].The heterogeneous geographic relationship between household income and undernutrition is also affected by the allocation of household income that is a function of maternal education, access to markets, infrastructure and sanitation [36]. Additionally, these data suggest that there is a strong and highly significant association between higher food insecurity (child hunger frequency in the preceding year) and increased thinness/wasting. Community and government based packages of support need to be highly targeted to the poorest and most food insecure households to further reduce inequality in this regard and maximise reductions in malnutrition.

Our findings suggest that children with low birth weight (due to pre-term delivery, fetal/intrauterine growth restriction or a combination of the two) were significantly more likely to be stunted than normal weight babies and this has been demonstrated in many other low and middle income settings (for example [37]). Socioeconomic status/factors are known risk factors for LBW [38]and may in part explain the significant association found between stunting and lower household income. South Africa has the higher number of incident and prevalent HIV infections globally [39]. A further important contextual risk factor for LBW is maternal HIV status. A systematic review and large observational studies focussing on low and middle incoming countries, suggest a strong and significant association between maternal HIV infection and LBW [40 41]. Evidence from South Africa also suggests the anthropometric z-score of HIV-infected children appear to be consistently lower when compared to HIV-exposed but uninfected children [42]. We also observed a significantly higher prevalence of stunting among male

For peer review only

[2] https://southafrica-info.com/people/mapping-poverty-in-south-africa/

For peer review only - http://bmjopen.bmj.com/site/about/guidelines.xhtml

children which has been demonstrated previously in a meta-analysis for sub-Saharan Africa [43], the suggested cause of which might be that male children are more vulnerable to health inequalities relative to female children of the same age. Strengthening community-based packages of care and community health worker (CHW) performance/skills in rural and high burden geographies are key strategies to improve primary health care delivery through better identification of women at higher risk of poor birth outcomes (e.g. HIV positive, history of previous poor birth outcomes and/or currently malnourished), higher referral rates for facility births, and improved linkage to other health as well as social services [44]. Lastly given the high adolescent fertility rates in many parts of South Africa [45], there is also much scope to improve CHW identification of households with higher risk malnourished adolescent girls prior to pregnancy to ensure more optimal linkage to government and social support to ensure adequate nutrition as well as improved awareness regarding family planning practices e.g. ensuring adequate birth spacing [46].

Obesity in children has a complex aetiology that includes a wide range of socioeconomic, demographic, environmental and cultural variables [47]such as household composition, mother's education, household income, household size, environmental factors, rural versus urban location, and sanitation [9 48]. The high burden of obesity is likely associated with a progressive increases in the per capita food supply and consumption of high calorific foods (e.g. fat, sugar, fast and/or processed foods) in South Africa[49]. This rapidly changing dietary pattern has, in part, been attributed to urbanisation, growing and expanding supermarkets /formal food retailers, and the availability of fast/processed foods [50]. An interesting finding in these data was the significant positive association between child obesity status and residing in a lower income household. This association has been demonstrated previously [51-53] and this evidence base is growing. This conforms with the idea that lower and higher income households/families often have a higher obesity risk than middle income households i.e. so called U-shaped association. Lower income or economically deprived families often replace health fresh food options with cheaper and more calorific processed foods [52]. Multiple studies have demonstrated that the majority of low-income South Africans have a low dietary diversity, and, therefore, consume a limited food range consisting predominantly of a starchy staple such as bread and maize, with low intakes of vegetables and fruit [49]. Future work will characterise food purchasing patterns (and changes over time) among households in South Africa which will be compared with paired longitudinal anthropometric measurements to identify specific dietary patterns associated with child nutritional status.

For peer review only - http://bmjopen.bmj.com/site/about/guidelines.xhtml

361 Lastly and contextually, body mass is culturally influenced in South Africa, and the high level of obesity in
362 KwaZulu-Natal and Eastern Cape may at least in part be a result of cultural beliefs that associate overweight with
363 wealth and good health [54]. Geographic patterns of higher obesity in South Africa appeared to overlap areas of high
364 poverty particular on the eastern side of the country[3] and thus not solely concentrated among higher socio-
365 economic households.

366 ***Strengths:*** To our knowledge this is the first spatial-temporal analysis of malnutrition trends among children
367 under five years of age in South Africa. We used standardised anthropometric measurements of children and their
368 mothers from a nationally representative repeated panel data over a 10-year period. The panel nature of the design
369 allows assessment of change in malnutrition burden within the same individuals/households observed at multiple
370 time points. A further strength was the implementation of a fully Bayesian space-time shared component model to
371 produce more stable joint estimates of malnutrition by province, district and year.

372 ***Weaknesses***: The study has several limitations. Firstly, missing or invalid weight/height measurements (especially
373 in wave 2, and among infants – Supplementary Material 7) may have introduced selection bias (if not missing at
374 random), and may thus have affected both the internal validity and the representativeness the findings in the
375 broader South African context. Secondly as the primary panel study was not designed/powered for provincial
376 [14]and lower geographic level analysis, we cannot discount the resultant impact on precision/random variability
377 when analysing at provincial/district level (administrative tier just below province) and further stratification by
378 socio-demographic correlates. Thirdly, we cannot discount the effect of inter-observer variability across different
379 study districts, despite extensive interviewer training and standardization of study protocols. All anthropometric
380 measurements (e.g. weight, height) were taken in duplicate in NIDS [26] which would have ensured better
381 reliability.

382 ***Cost of malnutrition, policy and research needs***: Estimating the cost of child malnutrition in South Africa is
383 extremely complicated and no locally-determined cost data exist. Data from the United States, suggest that the
384 incremental lifetime direct medical cost for a 10-year-old obese child relative to a 10-year-old normal weight child
385 ranges from USD 12 660 to USD 19 630 [55]. Estimates of the cost of treating wasted children are approximately
386 USD 200 per child [56] while stunting has been consistently linked to worse economic outcomes in adulthood [57] and
387 estimates suggest that, on average, the future per capita income penalty for a stunted individual could be as large

[3] https://southafrica-info.com/people/mapping-poverty-in-south-africa/

For peer review only - http://bmjopen.bmj.com/site/about/guidelines.xhtml

388 as 9-10% in SSA [58]. Urgent investments are needed to accelerate the reduction of all forms of malnutrition, as
389 well as to curb the obesity epidemic among young children in South Africa. There is also considerable evidence
390 indicates that childhood wasting and stunting can be reduced by 60% and 20% respectively using ten nutrition-
391 specific interventions [59], with an estimated return on investment (ROI) of 18:1, i.e. for USD 1 spent on
392 implementing effective programmes there would be USD 18 return in future economic benefits [60]. Very few
393 obesity prevention interventions targeting children have been effective and a comprehensive multifaceted strategy
394 tackling diet, physical inactivity, coupled with psychosocial support and local food environment change may
395 prove more effective. Nutrition policies tackling child obesity must promote household nutrition security and
396 healthy growth, decrease overconsumption of nutrient-poor foods, better shield children from increasingly
397 pervasive marketing of energy-dense, nutrient-poor foods and sugar sweetened beverages as well as reduction of
398 growing physical inactivity [61].

399 Our findings suggest the need to implement evidence-based child health strategies and policy (e.g. further social
400 grant support to vulnerable and impoverished households) that is tailored to specific geographies and socially
401 disadvantaged sub-populations. A higher prevalence of child thinness/wasting among younger mothers (<25) in
402 poorer, food insecure household, highlights the importance of policies that enable younger mothers to adequately
403 care for their children in all settings. Integrated nutrition programs in low and middle income countries (LMIC)
404 have had a substantial impact on child nutrition and health via a combination of multisector targeted interventions
405 [62]. Furthermore implementation and/or strengthening of school-based food program can provide a launching pad
406 for preventive programs including education and awareness, provision of healthier/more nutrition food options
407 and micronutrient supplementation, deworming, increased immunization coverage and improved growth
408 monitoring as well as counselling [62]. This may be especially true of obese children where high prevalence was
409 observed in higher income households with higher food purchasing power and where local food environments are
410 likely is likely to be an important contextual determinant. A further contextual trend which may further compound
411 this problem is the rapidly rising median household income observed over the period (from ZAR1400 in 2008 to
412 ZAR 3640 by 2017).

413 **Conclusions**

414 The heterogeneity of malnutrition is a feature of spatial inequality and rapid urbanization that has manifested in
415 widening levels of inequality in South Africa's districts and a need to reassess where nutrition programmes need
416 to be further decentralised to the highest risk municipalities and local communities to maximise effectiveness.

For peer review only - http://bmjopen.bmj.com/site/about/guidelines.xhtml
For peer review only

This work provides the first district level ranking of childhood overweight, thinness/wasting and stunting and allows a differentiated pro-active tailored intervention to be developed for each municipal district. The dual epidemic of undernutrition and overweight/obesity requires differential geographical policy inputs in metropolitan areas and districts across the rural-urban divide. The current and future health cost of malnutrition among South African children is likely substantial based on previous costing estimates. There is an urgent need to address nutrition problems among preschool aged children in South Africa and other low and middle income countries. Effective public health planning and geographically/contextually tailored interventions are required at sub-national level to address this challenge. The analytical framework employed in this study we believe will have definite utility in other settings.

References

1. Development Initiatives. 2018 Global Nutrition Report: Shining a light to spur action on nutrition. Bristol, UK: Development Initiatives, 2018.
2. Finucane MM, Stevens G, Cowan M, et al. Global Burden of Metabolic Risk Factors of Chronic Diseases Collaborating Group (Body Mass Index) National, regional, and global trends in body-mass index since 1980: systematic analysis of health examination surveys and epidemiological studies with 960 country-years and 9.1 million participants. *Lancet* 2011;377(9765):557-67.
3. Collaborators TGO. Health Effects of Overweight and Obesity in 195 Countries over 25 Years. *New England Journal of Medicine*;0(0):null. doi: 10.1056/NEJMoa1614362
4. Collaboration NRF. Trends in adult body-mass index in 200 countries from 1975 to 2014: a pooled analysis of 1698 population-based measurement studies with 19· 2 million participants. *The Lancet* 2016;387(10026):1377-96.
5. Bleich SN, Vercammen KA, Zatz LY, et al. Interventions to prevent global childhood overweight and obesity: a systematic review. *The Lancet Diabetes & Endocrinology* 2017
6. Malik VS, Willett WC, Hu FB. Global obesity: trends, risk factors and policy implications. *Nature Reviews Endocrinology* 2013;9(1):13-27.
7. Bain LE, Awah PK, Geraldine N, et al. Malnutrition in Sub–Saharan Africa: burden, causes and prospects. *Pan African Medical Journal* 2013;15(1)
8. Rossouw HA, Grant CC, Viljoen M. Overweight and obesity in children and adolescents: The South African problem. *South African journal of science* 2012;108(5-6):31-37.
9. Keino S, Plasqui G, Ettyang G, et al. Determinants of stunting and overweight among young children and adolescents in sub-Saharan Africa. *Food and nutrition bulletin* 2014;35(2):167-78.
10. Osgood-Zimmerman A, Millear AI, Stubbs RW, et al. Mapping child growth failure in Africa between 2000 and 2015. *Nature* 2018;555(7694):41.
11. Cois A, Day C. Obesity trends and risk factors in the South African adult population. *BMC obesity* 2015;2(1):42.
12. Sartorius B, Veerman LJ, Manyema M, et al. Determinants of obesity and associated population attributability, South Africa: Empirical evidence from a national panel survey, 2008-2012. *PloS one* 2015;10(6):e0130218.
13. Von Elm E, Altman DG, Egger M, et al. The Strengthening the Reporting of Observational Studies in Epidemiology (STROBE) statement: guidelines for reporting observational studies. *Journal of clinical epidemiology* 2008;61(4):344-49.
14. Leibbrandt M, Woolard I, de Villiers L. Methodology: Report on NIDS wave 1. *Technical paper* 2009;1

For peer review only - http://bmjopen.bmj.com/site/about/guidelines.xhtml

15. Southern Africa Labour and Development Research Unit. National Income Dynamics Study 2017, Wave 5 [dataset]. Version 1.0.0 In: Pretoria: Department of Planning M, and Evaluation [funding agency]. Cape Town: Southern Africa Labour and Development Research Unit [implementer], 2018. , ed.: Cape Town: DataFirst [distributor], 2018., 2018.

16. Health WHONf. WHO child growth standards: growth velocity based on weight, length and head circumference: methods and development: World Health Organization 2009.

17. Organization WH, Health WHONf. WHO child growth standards: head circumference-for-age, arm circumference-for-age, triceps skinfold-for-age and subscapular skinfold-for-age: methods and development: World Health Organization 2007.

18. Gibson RS. Principles of nutritional assessment: Oxford university press, USA 2005.

19. Anselin L, Syabri I, Kho Y. GeoDa: an introduction to spatial data analysis. *Geographical analysis* 2006;38(1):5-22.

20. Abellan JJ, Richardson S, Best N. Use of space–time models to investigate the stability of patterns of disease. *Environmental health perspectives* 2008;116(8):1111-19.

21. Besag J, York J, Mollié A. Bayesian image restoration, with two applications in spatial statistics. *Annals of the institute of statistical mathematics* 1991;43(1):1-20.

22. Wakefield J, Best N, Waller L. Bayesian approaches to disease mapping. *Spatial epidemiology: methods and applications* 2000:104-07.

23. Organization WH. Global nutrition targets 2025: Policy brief series: World Health Organization, 2014.

24. Richardson S, Thomson A, Best N, et al. Interpreting posterior relative risk estimates in disease-mapping studies. *Environmental Health Perspectives* 2004;112(9):1016-25.

25. Gelman A, Rubin DB. Inference from iterative simulation using multiple sequences. *Statistical science* 1992;7(4):457-72.

26. Zere E, McIntyre D. Inequities in under-five child malnutrition in South Africa. *International journal for equity in health* 2003;2(1):7.

27. Jinabhai C, Taylor M, Sullivan K. Implications of the prevalence of stunting, overweight and obesity amongst South African primary school children: a possible nutritional transition? *European journal of clinical nutrition* 2003;57(2):358.

28. Armstrong ME, Lambert MI, Lambert EV. Secular trends in the prevalence of stunting, overweight and obesity among South African children (1994–2004). *European journal of clinical nutrition* 2011;65(7):835.

29. Kandala N-B, Madungu TP, Emina JB, et al. Malnutrition among children under the age of five in the Democratic Republic of Congo (DRC): does geographic location matter? *BMC public health* 2011;11(1):261.

30. Bredenkamp C, Buisman LR, Van de Poel E. Persistent inequalities in child undernutrition: evidence from 80 countries, from 1990 to today. *International Journal of Epidemiology* 2014;43(4):1328-35.

31. Di Cesare M, Bhatti Z, Soofi SB, et al. Geographical and socioeconomic inequalities in women and children's nutritional status in Pakistan in 2011: an analysis of data from a nationally representative survey. *The Lancet Global Health* 2015;3(4):e229-e39.

32. Ataguba JE, Akazili J, McIntyre D. Socioeconomic-related health inequality in South Africa: evidence from General Household Surveys. *International journal for equity in health* 2011;10(1):48.

33. Van de Poel E, Hosseinpoor AR, Speybroeck N, et al. Socioeconomic inequality in malnutrition in developing countries. *Bulletin of the World Health Organization* 2008;86:282-91.

34. Delpeuch F, Traissac P, Martin-Prével Y, et al. Economic crisis and malnutrition: socioeconomic determinants of anthropometric status of preschool children and their mothers in an African urban area. *Public health nutrition* 2000;3(1):39-47.

35. Oyvat C. Agrarian structures, urbanization, and inequality. *World Development* 2016;83:207-30.

36. Hoddinott J, Haddad L. Does female income share influence household expenditures? Evidence from Côte d'Ivoire. *oxford Bulletin of Economics and Statistics* 1995;57(1):77-96.

For peer review only - http://bmjopen.bmj.com/site/about/guidelines.xhtml

37. Aryastami NK, Shankar A, Kusumawardani N, et al. Low birth weight was the most dominant predictor associated with stunting among children aged 12–23 months in Indonesia. *BMC Nutrition* 2017;3(1):16.
38. de Bernabé JV, Soriano T, Albaladejo R, et al. Risk factors for low birth weight: a review. *European Journal of Obstetrics & Gynecology and Reproductive Biology* 2004;116(1):3-15.
39. Frank TD, Carter A, Jahagirdar D, et al. Global, regional, and national incidence, prevalence, and mortality of HIV, 1980–2017, and forecasts to 2030, for 195 countries and territories: a systematic analysis for the Global Burden of Diseases, Injuries, and Risk Factors Study 2017. *The Lancet HIV* 2019
40. Xiao P-L, Zhou Y-B, Chen Y, et al. Association between maternal HIV infection and low birth weight and prematurity: a meta-analysis of cohort studies. *BMC pregnancy and childbirth* 2015;15(1):246.
41. Althabe F, Moore JL, Gibbons L, et al. Adverse maternal and perinatal outcomes in adolescent pregnancies: The Global Network's Maternal Newborn Health Registry study. *Reproductive health* 2015;12(2):S8.
42. Patel D, Bland R, Coovadia H, et al. Breastfeeding, HIV status and weights in South African children: a comparison of HIV-exposed and unexposed children. *Aids* 2010;24(3):437-45.
43. Wamani H, Åstrøm AN, Peterson S, et al. Boys are more stunted than girls in sub-Saharan Africa: a meta-analysis of 16 demographic and health surveys. *BMC pediatrics* 2007;7(1):17.
44. Redding S, Conrey E, Porter K, et al. Pathways community care coordination in low birth weight prevention. *Maternal and child health journal* 2015;19(3):643-50.
45. Statistics South Africa. Recorded live births 2012. www.statssa.gov.za/publications/P0305/P03052011.pdf. 2012
46. Organization WH. WHA Global nutrition targets 2025: low birth weight policy brief. Geneva: WHO, 2014.[Accessed on: June 1st, 2017].
47. Monasta L, Batty G, Cattaneo A, et al. Early-life determinants of overweight and obesity: a review of systematic reviews. *Obesity reviews* 2010;11(10):695-708.
48. Kruger R, Kruger H, Macintyre U. The determinants of overweight and obesity among 10-to 15-year-old schoolchildren in the North West Province, South Africa–the THUSA BANA (Transition and Health during Urbanisation of South Africans; BANA, children) study. *Public health nutrition* 2006;9(3):351-58.
49. Pereira LM. The future of South Africa's food system: What is research telling us. *SA Food Lab South Africa* 2014
50. McLachlan M, Landman A. Nutrition-sensitive agriculture–a South African perspective. *Food security* 2013;5(6):857-71.
51. Brophy S, Cooksey R, Gravenor MB, et al. Risk factors for childhood obesity at age 5: analysis of the millennium cohort study. *BMC public health* 2009;9(1):467.
52. Brunt H, Lester N, Davies G, et al. Childhood overweight and obesity: is the gap closing the wrong way? *Journal of public health* 2008;30(2):145-52.
53. Kim TJ, von dem Knesebeck O. Income and obesity: what is the direction of the relationship? A systematic review and meta-analysis. *BMJ open* 2018;8(1):e019862.
54. Rossouw HA, Grant CC, Viljoen M. Overweight and obesity in children and adolescents: the South African problem: review article. *South African Journal of Science* 2012;108(5-6):1-7.
55. Finkelstein EA, Graham WCK, Malhotra R. Lifetime direct medical costs of childhood obesity. *Pediatrics* 2014:peds. 2014-0063.
56. Horton S, Shekar M, Ajay M. Scaling up nutrition: What will it cost?: The World Bank 2009.
57. McGovern ME, Krishna A, Aguayo VM, et al. A review of the evidence linking child stunting to economic outcomes. *International journal of epidemiology* 2017;46(4):1171-91.
58. Sartorius K, Sartorius B, Winkler C, et al. 1. Abstract 2. Introduction 3. miRNA biogenesis, mRNA silencing and export into the extracellular space 3.1. Intracellular mRNA silencing 3.2. Extracellular mRNA silencing.

For peer review only - http://bmjopen.bmj.com/site/about/guidelines.xhtml

59. Vaivada T, Gaffey MF, Das JK, et al. Evidence-based interventions for improvement of maternal and child nutrition in low-income settings: what's new? *Current Opinion in Clinical Nutrition & Metabolic Care* 2017;20(3):204-10.

60. Hoddinott J, Alderman H, Behrman JR, et al. The economic rationale for investing in stunting reduction. *Maternal & Child Nutrition* 2013;9:69-82.

61. Lobstein T, Jackson-Leach R, Moodie ML, et al. Child and adolescent obesity: part of a bigger picture. *The lancet* 2015;385(9986):2510-20.

62. UNICEF. Progress for children: a report card on nutrition: Unicef 2006.

Authors' contributions

BS contributed to the conceptualisation/design, methodology, data analysis, drafted the initial manuscript and approved the final version of the manuscript. KS, RG, EL, PS and FT reviewed/edited the manuscript for critically important intellectual content and approved the final version of the manuscript. AD and RS participated in funding acquisition, conceptualisation/design, supervision, critically reviewed/edited the manuscript for critically important intellectual content and approved the final version of the manuscript.

Funding

This study forms part of the Sustainable and Healthy Food Systems (SHEFS) project supported by the Wellcome Trust's Our Planet, Our Health programme (grant number 205200/Z/16/Z). The funders of the study had no role in study design, data collection, data analysis, data interpretation or writing of the report.

Competing interests

None declared.

Patient consent for publication

Not required.

Ethics approval

This study utilised open access data and hence ethical approval was not necessary.

Data availability statement

Data are publically available at https://www.datafirst.uct.ac.za/dataportal/index.php/catalog/NIDS/about

For peer review only

Tables

Table 1: Socio-demographic characteristics of sampled children by survey round

Variable	Category	Wave 1: 2008	Wave 2: 2010/11	Wave 3: 2012	Wave 4: 2014/15	Wave 5: 2017
		n (%)	n (%)	n (%)	n (%)	n (%)
Age (in years)	<1	661 (20.3)	517 (14.6)	652 (17)	886 (19.7)	813 (17.3)
	1-1.99	661 (20.3)	621 (17.5)	691 (18)	875 (19.5)	909 (19.3)
	2-2.99	670 (20.6)	751 (21.2)	764 (19.9)	863 (19.2)	996 (21.1)
	3-3.99	642 (19.7)	840 (23.7)	826 (21.5)	914 (20.3)	992 (21.1)
	4-4.99	620 (19.1)	820 (23.1)	909 (23.7)	960 (21.3)	1000 (21.2)
Gender	Male	1640 (50.4)	1773 (50)	1856 (48.3)	2173 (48.3)	2325 (49.4)
	Female	1614 (49.6)	1770 (49.9)	1986 (51.7)	2322 (51.6)	2385 (50.6)
Ethnicity [i]	African	2723 (83.7)	3047 (85.9)	3307 (86.1)	3898 (86.7)	4048 (85.9)
	Coloured	429 (13.2)	423 (11.9)	455 (11.8)	532 (11.8)	523 (11.1)
	Asian/Indian	32 (1)	26 (0.7)	24 (0.6)	30 (0.7)	0 (0)
	White	70 (2.2)	53 (1.5)	56 (1.5)	29 (0.6)	0 (0)
Birthweight	LBW (<2.5 kgs)	249 (7.7)	267 (7.5)	364 (9.5)	459 (10.2)	460 (9.8)
	NBW (≥2.5 kgs)	2401 (73.8)	2553 (71.9)	3110 (80.9)	3605 (80.1)	3563 (75.6)
	HBW (≥4 kgs)	105 (3.2)	99 (2.8)	121 (3.1)	156 (3.5)	157 (3.3)
	Non-HBW (<4kgs)	2545 (78.2)	2721 (76.7)	3353 (87.3)	3908 (86.9)	3866 (82.1)
	Missing BW	604 (18.6)	729 (20.5)	368 (9.6)	434 (9.6)	687 (14.6)
Low monthly household income	<R2500	1737 (53.4)	1804 (50.8)	1660 (43.2)	1484 (33)	1202 (25.5)
	≥R2500	552 (17)	1014 (28.6)	1686 (43.9)	2749 (61.1)	3109 (66)
Child hungry in last year (food security) [ii]	Never	2148 (66)				
	Seldom	333 (10.2)				
	Sometimes	583 (17.9)	N/A			
	Often	149 (4.6)				
	Always	35 (1.1)				
Province	Eastern Cape	437 (13.4)	442 (12.5)	437 (11.4)	545 (12.1)	545 (11.6)
	Free State	163 (5)	171 (4.8)	200 (5.2)	244 (5.4)	242 (5.1)
	Gauteng	274 (8.4)	346 (9.7)	381 (9.9)	455 (10.1)	538 (11.4)
	KwaZulu-Natal	1057 (32.5)	1076 (30.3)	1188 (30.9)	1449 (32.2)	1534 (32.6)
	Limpopo	293 (9)	348 (9.8)	423 (11)	497 (11)	471 (10)
	Mpumalanga	231 (7.1)	257 (7.2)	283 (7.4)	307 (6.8)	356 (7.6)
	North West	226 (6.9)	240 (6.8)	269 (7)	293 (6.5)	296 (6.3)
	Northern Cape	243 (7.5)	224 (6.3)	258 (6.7)	316 (7)	322 (6.8)
	Western Cape	330 (10.1)	344 (9.7)	367 (9.6)	368 (8.2)	368 (7.8)
Environment	Rural Formal	324 (10)	350 (9.9)	343 (8.9)	389 (8.6)	449 (9.5)
	Tribal Authority Area	1583 (48.6)	1526 (43)	1801 (46.9)	2154 (47.9)	2135 (45.3)
	Urban Formal	1133 (34.8)	1221 (34.4)	1319 (34.3)	1498 (33.3)	1702 (36.1)
	Urban Informal	214 (6.6)	228 (6.4)	257 (6.7)	303 (6.7)	317 (6.7)
Mother BMI	Underweight	85 (2.6)	78 (2.2)	58 (1.5)	98 (2.2)	135 (2.9)
	Normal	1010 (31)	1105 (31.1)	1250 (32.5)	1373 (30.5)	1485 (31.5)
	Overweight	734 (22.6)	850 (24)	962 (25)	1054 (23.4)	1053 (22.4)
	Obese	932 (28.6)	987 (27.8)	1054 (27.4)	1377 (30.6)	1382 (29.3)
	Missing	493 (15.2)	529 (14.9)	518 (13.5)	596 (13.3)	655 (13.9)
Mother age	<20	234 (7.2)	238 (6.7)	259 (6.7)	316 (7)	322 (6.8)
	20-24	807 (24.8)	872 (24.6)	971 (25.3)	1100 (24.5)	1062 (22.5)
	25-34	1213 (37.3)	1413 (39.8)	1566 (40.8)	1853 (41.2)	2004 (42.5)

For peer review only

For peer review only - http://bmjopen.bmj.com/site/about/guidelines.xhtml

	35-44	583 (17.9)	581 (16.4)	633 (16.5)	682 (15.2)	772 (16.4)
	45+	81 (2.5)	92 (2.6)	82 (2.1)	86 (1.9)	98 (2.1)
	Missing	336 (10.3)	353 (9.9)	331 (8.6)	461 (10.2)	452 (9.6)
Mother education	None	131 (4)	115 (3.2)	76 (2)	48 (1.1)	81 (1.7)
	Primary	505 (15.5)	419 (11.8)	405 (10.5)	387 (8.6)	97 (2.1)
	Secondary	1871 (57.5)	2265 (63.8)	2654 (69.1)	3176 (70.6)	3130 (66.5)
	Tertiary	132 (4.1)	141 (4)	172 (4.5)	240 (5.3)	707 (15)
	Missing	615 (18.9)	609 (17.2)	535 (13.9)	647 (14.4)	695 (14.8)

i: 139 misclassified or missing in 2017
ii: only included in wave 1 questionnaire

Table 2: Burden of stunting, thinness/wasting and obesity among children by age and survey round

Survey wave	Age (in years)	N (valid HAZ)	n (stunted)	Prop: Stunted i	Estimated Population stunted	N (valid BMIZ)	n (thin /wasted)	Prop: Thinness ii	Estimated Population thinness	n (obese)	Prop: obese iii	Estimated Population obese
2008	0	220	31	0.14 (0.09, 0.22)	153648 (81545, 273371)	180	21	0.12 (0.07, 0.2)	133882 (66374, 251867)	32	0.1 (0.06, 0.15)	107783 (59737, 185749)
	1	419	29	0.08 (0.05, 0.13)	91903 (48436, 164369)	386	24	0.06 (0.03, 0.11)	66566 (29263, 143661)	76	0.22 (0.16, 0.3)	253021 (159436, 383096)
	2	453	62	0.15 (0.1, 0.21)	159241 (96989, 250626)	419	10	0.03 (0.01, 0.07)	34613 (12484, 87598)	70	0.14 (0.1, 0.19)	148357 (93148, 227510)
	3	489	55	0.11 (0.08, 0.15)	111595 (69906, 172639)	470	19	0.04 (0.02, 0.07)	39715 (20205, 75821)	67	0.17 (0.12, 0.24)	176235 (104092, 284620)
	4	498	48	0.09 (0.06, 0.13)	93391 (54519, 154136)	461	25	0.05 (0.03, 0.08)	52031 (27083, 96623)	34	0.08 (0.05, 0.12)	80282 (45874, 135732)
	**0-5**	**2079**	**225**	**0.11 (0.09, 0.13) iv**	**591550 (451494, 766049)**	**1916**	**99**	**0.05 (0.04, 0.07) iv**	**277743 (196715, 385904)**	**279**	**0.14 (0.12, 0.17) iv**	**778865 (599156, 996439)**
2010/11	0	75	24	0.33 (0.16, 0.57)	289420 (114550, 577181)	69	7	0.1 (0.04, 0.23)	88499 (30258, 228461)	22	0.39 (0.21, 0.61)	340820 (153454, 615984)
	1	236	20	0.06 (0.03, 0.11)	63995 (25204, 132218)	215	11	0.07 (0.03, 0.14)	69776 (25204, 173842)	52	0.29 (0.19, 0.41)	299127 (159624, 499489)
	2	340	61	0.22 (0.16, 0.29)	267019 (166414, 407708)	314	17	0.06 (0.03, 0.11)	76344 (35363, 155183)	72	0.22 (0.16, 0.29)	270818 (167454, 414761)
	3	427	52	0.11 (0.07, 0.16)	130531 (73921, 220389)	402	20	0.03 (0.02, 0.06)	39208 (16427, 85938)	78	0.16 (0.11, 0.23)	195314 (114988, 313258)
	4	422	62	0.17 (0.12, 0.24)	205730 (122130, 329629)	394	19	0.03 (0.02, 0.06)	39494 (17639, 84450)	65	0.17 (0.12, 0.24)	208842 (126152, 329629)
	**0-5**	**1500**	**219**	**0.16 (0.13, 0.19)**	**862302 (633920, 1148376)**	**1394**	**74**	**0.05 (0.03, 0.07)**	**265877 (167080, 405309)**	**289**	**0.21 (0.17, 0.26)**	**1159133 (835398, 1565968)**
2012	0	271	59	0.2 (0.14, 0.28)	181464 (108101, 288795)	250	38	0.2 (0.12, 0.3)	179118 (95658, 311389)	55	0.19 (0.12, 0.28)	169192 (94880, 284482)
	1	544	78	0.13 (0.09, 0.17)	132310 (80796, 207206)	538	27	0.08 (0.05, 0.13)	80862 (40842, 150046)	138	0.23 (0.18, 0.28)	234062 (157153, 334626)
	2	629	72	0.1 (0.07, 0.14)	116230 (68690, 187924)	629	49	0.05 (0.03, 0.07)	55866 (30861, 97391)	147	0.23 (0.18, 0.29)	269508 (176205, 392309)
	3	710	82	0.11 (0.08, 0.16)	142259 (82987, 232297)	692	29	0.03 (0.02, 0.06)	43898 (20928, 87296)	102	0.15 (0.11, 0.2)	191943 (117798, 297399)
	4	771	112	0.16 (0.12, 0.2)	221293 (142258, 330201)	762	30	0.03 (0.02, 0.05)	43556 (20731, 87406)	118	0.18 (0.14, 0.22)	250658 (167278, 362573)
	**0-5**	**2925**	**403**	**0.13 (0.11, 0.16)**	**762303 (567517, 1001855)**	**2871**	**173**	**0.06 (0.05, 0.07)**	**328768 (230074, 458914)**	**560**	**0.19 (0.17, 0.22)**	**1112487 (853832, 1415525)**
2014/15	0	434	74	0.12 (0.08, 0.18)	144201 (81319, 240730)	421	37	0.1 (0.06, 0.18)	123211 (59233, 240730)	78	0.17 (0.12, 0.23)	197209 (117461, 313223)
	1	801	53	0.06 (0.04, 0.08)	67916 (39433, 112566)	801	24	0.03 (0.01, 0.08)	39657 (9858, 101845)	169	0.23 (0.18, 0.28)	266780 (179421, 379240)
	2	785	65	0.08 (0.05, 0.12)	85985 (48668, 146305)	781	16	0.02 (0.01, 0.03)	16222 (6309, 39015)	128	0.16 (0.12, 0.22)	170803 (106348, 263349)
	3	853	82	0.08 (0.06, 0.11)	89857 (54478, 143034)	845	24	0.04 (0.02, 0.07)	40865 (18323, 86890)	79	0.12 (0.08, 0.15)	133857 (83637, 205862)
	4	899	67	0.06 (0.04, 0.09)	77887 (45801, 127320)	897	19	0.02 (0.01, 0.05)	30376 (12301, 71898)	56	0.06 (0.04, 0.11)	82300 (38662, 166265)
	**0-5**	**3772**	**341**	**0.08 (0.06, 0.09)**	**441281 (327611, 581707)**	**3745**	**120**	**0.04 (0.03, 0.05)**	**213012 (130004, 333338)**	**510**	**0.14 (0.12, 0.17)**	**834444 (618820, 1098053)**
2017	0	372	50	0.13 (0.08, 0.19)	125347 (68160, 218303)	357	32	0.12 (0.07, 0.2)	121396 (62270, 221478)	70	0.18 (0.12, 0.25)	174538 (104344, 278066)
	1	760	55	0.08 (0.05, 0.11)	95527 (56435, 153804)	742	23	0.03 (0.02, 0.07)	42416 (17767, 94222)	146	0.23 (0.19, 0.29)	285123 (194388, 403216)
	2	833	63	0.07 (0.05, 0.11)	94807 (54147, 158550)	830	20	0.03 (0.02, 0.07)	43976 (18786, 99279)	130	0.15 (0.12, 0.19)	191812 (127079, 280056)
	3	875	77	0.08 (0.05, 0.12)	99890 (54439, 175689)	872	14	0.02 (0.01, 0.06)	30726 (10888, 79204)	77	0.07 (0.05, 0.1)	88889 (54439, 138247)
	4	900	59	0.05 (0.04, 0.07)	57363 (34849, 91231)	899	23	0.03 (0.01, 0.05)	29923 (13628, 62962)	47	0.06 (0.04, 0.08)	63912 (36990, 105365)
	**0-5**	**3740**	**304**	**0.08 (0.06, 0.09) iv**	**445295 (326192, 593240)**	**3700**	**112**	**0.04 (0.03, 0.05) iv**	**223236 (136790, 345514)**	**470**	**0.13 (0.11, 0.15) iv**	**758650 (583989, 964831)**

For peer review only

For peer review only - http://bmjopen.bmj.com/site/about/guidelines.xhtml

**At last observation**	**0-5**	**10711**	**1049**	**0.09 (0.08, 0.10)**	**1 397 020 (1 177 247, 1 616 793)**	**10467**	**391**	**0.04 (0.03, 0.05)**	**560 806 (448 656, 672 957)**	**1,438**	**0.14 (0.13, 0.16)**	**2 048 650 (1 722 242, 2 375 058)**

i: HAZ ≤ -2 SD; ii BMI for age z-score ≤ -2SD; iii BMI for age z-score ≥ +2SD

iv: Significance tests (survey weighted logistic regression) among children 0-5: stunting (2017 vs 2008) p=0.007; thinness/wasting (2017 vs 2008) p=0.131; obesity (2017 vs 2008) p=0.312

Table 3: Demographic, socio-economic and maternal factors associated with nutritional status among children under 5 years, 2008-2017

**Variable**	**Category**	**Stunted**		**p-value**	**Thin/wasted**		**p-value**	**Obese**		**p-value**
		**Yes (% col)**	**No (% col)**		**Yes (% col)**	**No (% col)**		**Yes (% col)**	**No (% col)**	
Ethnicity	African	0.939 [.9027,.9619]	0.871 [.8284,.9039]		0.885 [.8155,.9306]	0.879 [.8383,.9108]		0.931 [.9017,.9522]	0.870 [.8255,.9044]	
	Coloured	0.053 [.0311,.0879]	0.074 [.0486,.1116]		0.076 [.0415,.1344]	0.072 [.0474,.1081]		0.052 [.0333,.0789]	0.076 [.0495,.1152]	
	Asian/Indian	0.003 [4.0e-04,.0202]	0.012 [.0049,.0294]		0.015 [.0026,.077]	0.011 [.0046,.0278]		0.004 [8.4e-04,.0141]	0.013 [.0053,.0317]	
	White	0.006 [.0017,.0179]	0.039 [.0238,.0627]	**<0.001**	0.025 [.0083,.0711]	0.037 [.0229,.0605]	0.823	0.014 [.0066,.0287]	0.041 [.0248,.067]	**<0.001**
Gender	Male	0.562 [.5204,.603]	0.496 [.4797,.5121]		0.514 [.4543,.5742]	0.501 [.4846,.5182]		0.523 [.488,.5575]	0.498 [.481,.5151]	
	Female	0.438 [.397,.4796]	0.504 [.4879,.5203]	**0.002**	0.486 [.4258,.5457]	0.499 [.4818,.5154]	0.686	0.477 [.4425,.512]	0.502 [.4849,.519]	0.178
Birthweight	LBW (<2.5 kgs)	0.148 [.1143,.1891]	0.098 [.0849,.1117]		0.13 [.0891,.1867]	0.098 [.0858,.1111]		0.072 [.0554,.0938]	0.104 [.0919,.118]	
	NBW (≥2.5 kgs)	0.852 [.8109,.8857]	0.903 [.8883,.9151]	**<0.001**	0.87 [.8133,.9109]	0.902 [.8889,.9142]	0.163	0.928 [.9062,.9446]	0.896 [.882,.9081]	**0.006**
	HBW (≥4 kgs)	Not applicable			Not applicable			0.056 [.0419,.0751]	0.04 [.0323,.0496]	
	Non-HBW (<4kgs)							0.944 [.9249,.9581]	0.96 [.9504,.9677]	**0.037**
Income quantile	Lowest	0.294 [.2567,.3334]	0.199 [.1824,.2156]		0.234 [.1805,.2973]	0.203 [.1872,.2195]		0.226 [.1936,.2617]	0.2 [.1834,.2181]	
	Low	0.205 [.1714,.2423]	0.187 [.1714,.2028]		0.214 [.1698,.2656]	0.188 [.173,.2029]		0.203 [.1725,.2377]	0.186 [.1723,.2005]	
	Middle	0.183 [.1555,.2148]	0.200 [.1853,.2154]		0.169 [.1305,.2167]	0.201 [.1871,.2162]		0.18 [.1501,.2135]	0.204 [.1891,.2189]	
	High	0.197 [.1579,.243]	0.186 [.1714,.2021]		0.184 [.1394,.2377]	0.191 [.1751,.2074]		0.182 [.1445,.2269]	0.192 [.1769,.2079]	
	Highest	0.122 [.0924,.1583]	0.229 [.2015,.2585]	**<0.001**	0.2 [.1494,.2612]	0.218 [.1906,.2476]	0.481	0.209 [.1673,.2586]	0.218 [.1915,.2478]	0.422
Low monthly household income	<R2500	0.566 [.5213,.6101]	0.417 [.3929,.4409]		0.488 [.4228,.5544]	0.423 [.3994,.4469]		0.481 [.4406,.5214]	0.416 [.392,.4396]	
	≥R2500	0.434 [.3899,.4787]	0.583 [.5591,.6071]	**<0.001**	0.512 [.4456,.5772]	0.577 [.5531,.6006]	**0.053**	0.519 [.4786,.5594]	0.584 [.5604,.608]	**0.001**
Child hungry in last year (food security) i	Never	0.689 [.595,.7701]	0.697 [.6568,.7346]		0.512 [.3895,.6337]	0.704 [.6643,.7401]		0.707 [.6302,.773]	0.693 [.6522,.7318]	
	Seldom	0.127 [.0669,.2286]	0.096 [.0766,.1193]		0.111 [.056,.2074]	0.097 [.0765,.1219]		0.076 [.0499,.1138]	0.102 [.0787,.13]	
	Sometimes	0.126 [.0807,.1919]	0.155 [.1303,.184]		0.317 [.219,.4354]	0.148 [.1243,.1752]		0.154 [.0994,.231]	0.155 [.1316,.1822]	
	Often	0.054 [.0265,.1049]	0.043 [.0276,.0653]		0.052 [.0222,.1181]	0.042 [.0272,.0655]		0.052 [.0272,.0981]	0.041 [.0269,.0621]	
	Always	0.004 [.0011,.0144]	0.009 [.0048,.0173]	0.505	0.007 [.001,.0504]	0.009 [.0049,.0171]	**<0.001**	0.011 [.0039,.0313]	0.009 [.0048,.016]	0.645
Province	Eastern Cape	0.165 [.1137,.2336]	0.132 [.0978,.1765]		0.075 [.0492,.1137]	0.137 [.1007,.1838]		0.19 [.1321,.2643]	0.124 [.0916,.1652]	
	Free State	0.066 [.0441,.0961]	0.050 [.036,.0678]		0.032 [.0169,.0611]	0.052 [.0376,.0709]		0.045 [.0298,.068]	0.052 [.0379,.071]	
	Gauteng	0.188 [.132,.2606]	0.236 [.1819,.2996]		0.298 [.1952,.4272]	0.231 [.1784,.2937]		0.173 [.1234,.2365]	0.246 [.1891,.3128]	
	KwaZulu-Natal	0.218 [.1619,.2857]	0.227 [.1801,.2819]		0.161 [.1151,.2195]	0.228 [.1804,.2835]		0.293 [.217,.3834]	0.212 [.1691,.262]	
	Limpopo	0.143 [.0947,.2088]	0.109 [.0816,.1444]	**0.012**	0.129 [.0823,.195]	0.113 [.0842,.1491]	**0.002**	0.074 [.0514,.105]	0.121 [.0902,.1599]	**<0.001**

For peer review only - http://bmjopen.bmj.com/site/about/guidelines.xhtml

	Mpumalanga	0.085 [.0541,.1318]	0.083 [.0621,.1102]		0.096 [.0611,.1487]	0.082 [.0611,.1098]		0.074 [.0506,.1079]	0.085 [.0626,.1131]	
	North West	0.055 [.0355,.0833]	0.05 [.035,.0709]		0.06 [.0376,.0943]	0.05 [.0346,.0712]		0.038 [.0252,.056]	0.053 [.0362,.076]	
	Northern Cape	0.022 [.0141,.0333]	0.023 [.0163,.031]		0.033 [.0217,.0489]	0.022 [.0159,.0303]		0.011 [.0072,.0156]	0.025 [.0178,.0341]	
	Western Cape	0.06 [.0321,.1089]	0.091 [.0606,.134]		0.116 [.0638,.2016]	0.086 [.0572,.1262]		0.103 [.0626,.1641]	0.084 [.0554,.1254]	
	Rural/Tribal authority	0.519 [.4417,.5963]	0.451 [.3933,.5091]		0.429 [.3428,.5201]	0.46 [.4021,.5193]		0.466 [.3857,.5479]	0.457 [.4002,.5158]	
	Urban Informal	0.122 [.0737,.1943]	0.101 [.0628,.1592]		0.1 [.0557,.1743]	0.102 [.0636,.161]		0.133 [.0691,.239]	0.097 [.0618,.148]	
Environment	Urban Formal	0.359 [.292,.4319]	0.448 [.389,.509]	**<0.001**	0.47 [.3734,.5696]	0.437 [.3787,.4979]	0.647	0.402 [.3261,.4821]	0.446 [.3868,.5066]	0.111
	Underweight	0.041 [.0271,.0604]	0.022 [.0178,.0282]		0.051 [.0298,.0867]	0.023 [.018,.0281]		0.019 [.01,.0351]	0.025 [.0198,.0311]	
	Normal	0.397 [.3521,.444]	0.344 [.3213,.3683]		0.418 [.3455,.4946]	0.348 [.3251,.3724]		0.327 [.2853,.3708]	0.356 [.332,.3815]	
	Overweight	0.268 [.2311,.3092]	0.273 [.2565,.289]		0.249 [.199,.3064]	0.272 [.2565,.2881]		0.26 [.23,.2922]	0.273 [.2567,.2899]	
Mother BMI	Obese	0.294 [.2452,.3479]	0.361 [.3342,.3882]	**0.003**	0.282 [.2137,.3615]	0.357 [.3298,.3853]	**0.005**	0.395 [.3514,.4396]	0.346 [.3175,.3753]	0.135
	<20	0.073 [.0562,.0947]	0.048 [.0419,.0555]		0.112 [.0574,.206]	0.047 [.041,.0532]		0.057 [.0456,.0701]	0.049 [.0418,.0562]	
	20-24	0.219 [.1852,.2571]	0.230 [.2152,.2459]		0.258 [.201,.3252]	0.23 [.2138,.2461]		0.265 [.2272,.3069]	0.224 [.2091,.2405]	
	25-34	0.468 [.4191,.5175]	0.491 [.4705,.5107]		0.398 [.3311,.4691]	0.492 [.4713,.5118]		0.472 [.425,.5189]	0.49 [.4691,.511]	
	35-44	0.215 [.1731,.2638]	0.210 [.191,.2297]		0.213 [.1682,.2667]	0.211 [.1923,.2301]		0.191 [.1536,.236]	0.214 [.1952,.2348]	
Mother age	45+	0.025 [.0161,.0381]	0.021 [.0177,.0256]	0. 156	0.019 [.0089,.0388]	0.022 [.018,.0261]	**0.007**	0.015 [.0095,.024]	0.023 [.0186,.0278]	0.121
	None	0.023 [.0136,.0397]	0.018 [.0144,.0226]		0.025 [.0127,.0479]	0.019 [.0148,.0239]		0.025 [.0157,.0406]	0.018 [.014,.023]	
	Primary	0.121 [.0921,.1576]	0.072 [.0625,.0835]		0.132 [.095,.1804]	0.071 [.061,.0825]		0.067 [.0488,.0925]	0.075 [.0647,.0869]	
	Secondary	0.799 [.7529,.8385]	0.796 [.7777,.8134]		0.715 [.6506,.7712]	0.802 [.7832,.8203]		0.803 [.7595,.8398]	0.798 [.7785,.8152]	
Mother education	Tertiary	0.057 [.0364,.0868]	0.114 [.0985,.1307]	**<0.001**	0.129 [.0862,.1874]	0.108 [.0925,.1251]	**0.001**	0.105 [.077,.1405]	0.11 [.0938,.1275]	0.568
	None	0.003 [8.0e-04,.0082]	0.003 [.0017,.0051]		0.005 [6.7e-04,.0333]	0.003 [.0017,.0051]		0.002 [6.8e-04,.0053]	0.003 [.0018,.0057]	
	Primary	0.646 [.5533,.7282]	0.56 [.5162,.6028]		0.565 [.4542,.6703]	0.556 [.5118,.5984]		0.584 [.499,.6637]	0.551 [.505,.5971]	
	Secondary	0.275 [.2008,.3629]	0.389 [.3468,.4334]		0.382 [.2783,.4965]	0.387 [.3448,.431]		0.318 [.2475,.3976]	0.398 [.3529,.445]	
Father education	Tertiary	0.077 [.0413,.1403]	0.048 [.035,.0651]	**0.020**	0.048 [.0206,.1099]	0.055 [.0389,.0761]	0.960	0.097 [.0502,.1779]	0.047 [.0338,.0658]	**0.033**

i: only included in wave 1 questionnaire

For peer review only

a) Stunting prevalence by province and survey wave

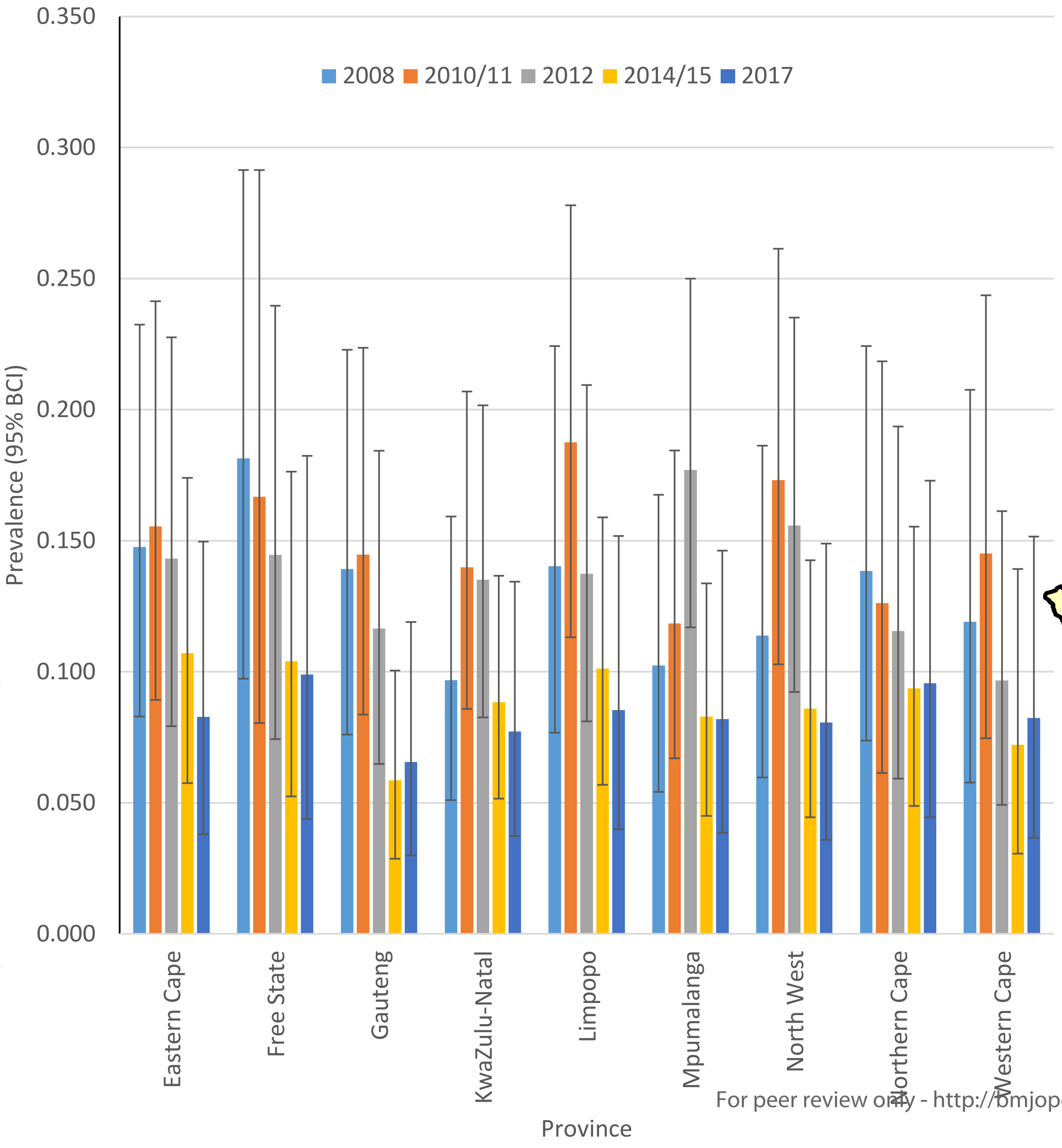

b) Stunting prevalence by district in 2017

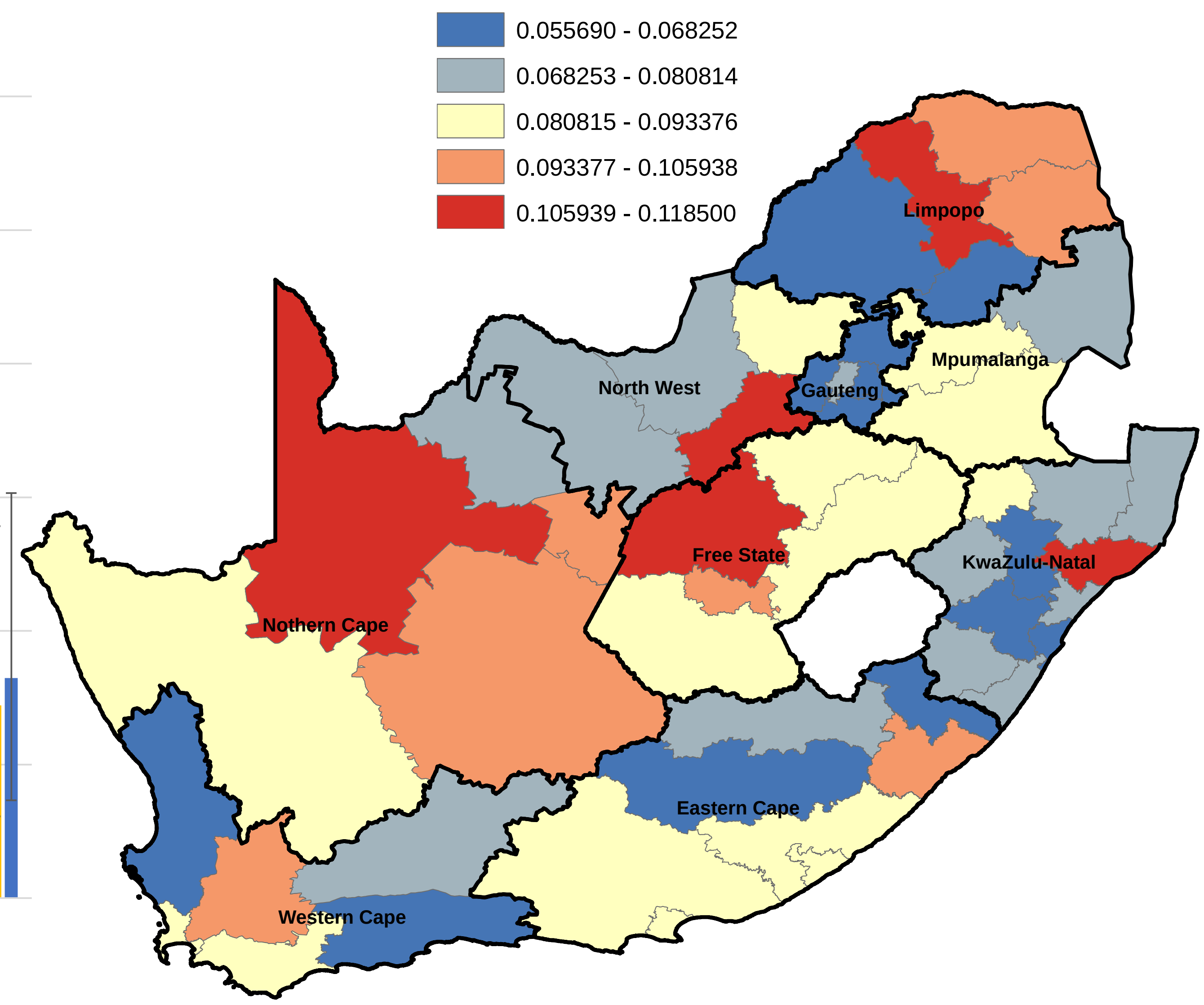

a) Thinness/wasting prevalence by province and survey wave

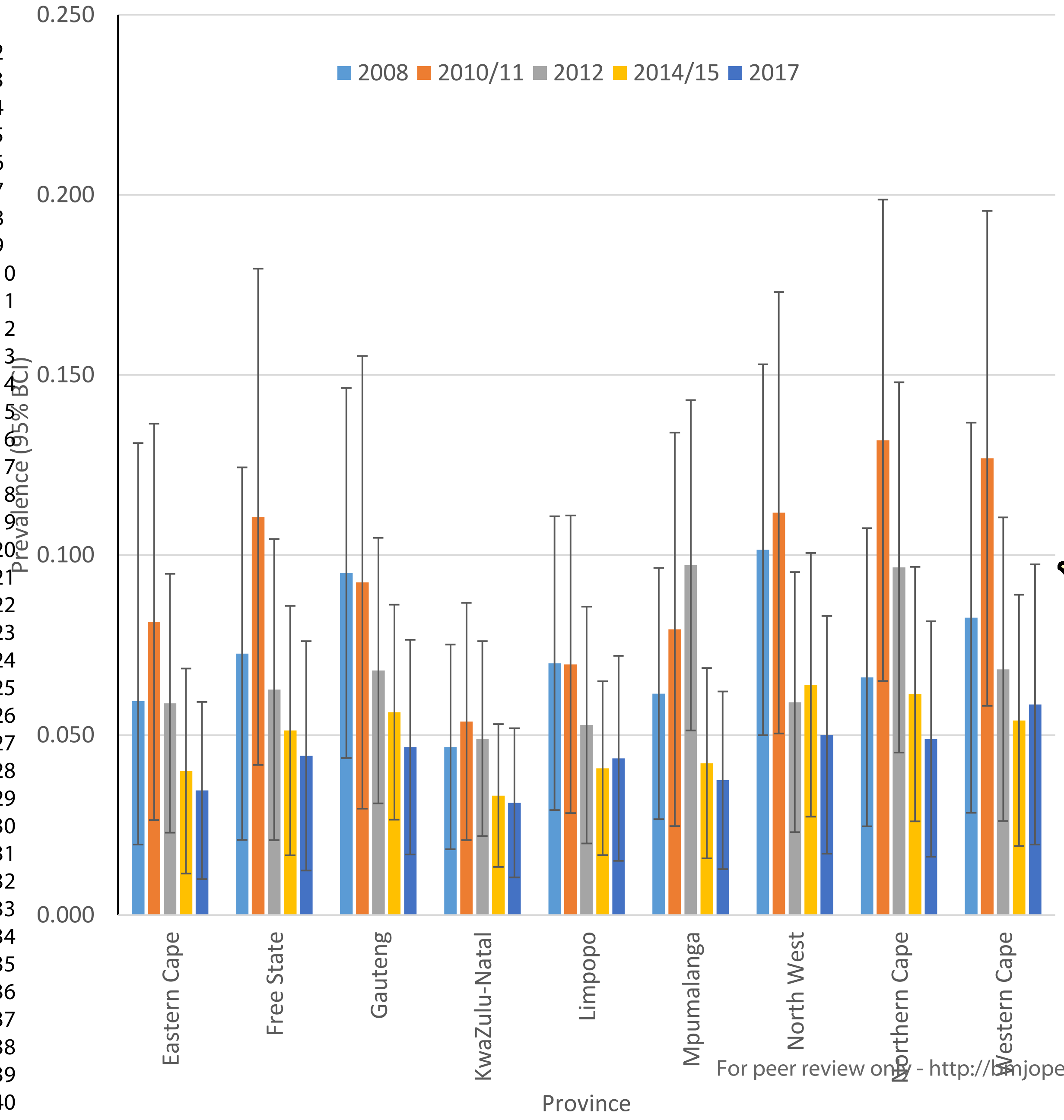

b) Thinness/wasting prevalence by district in 2017

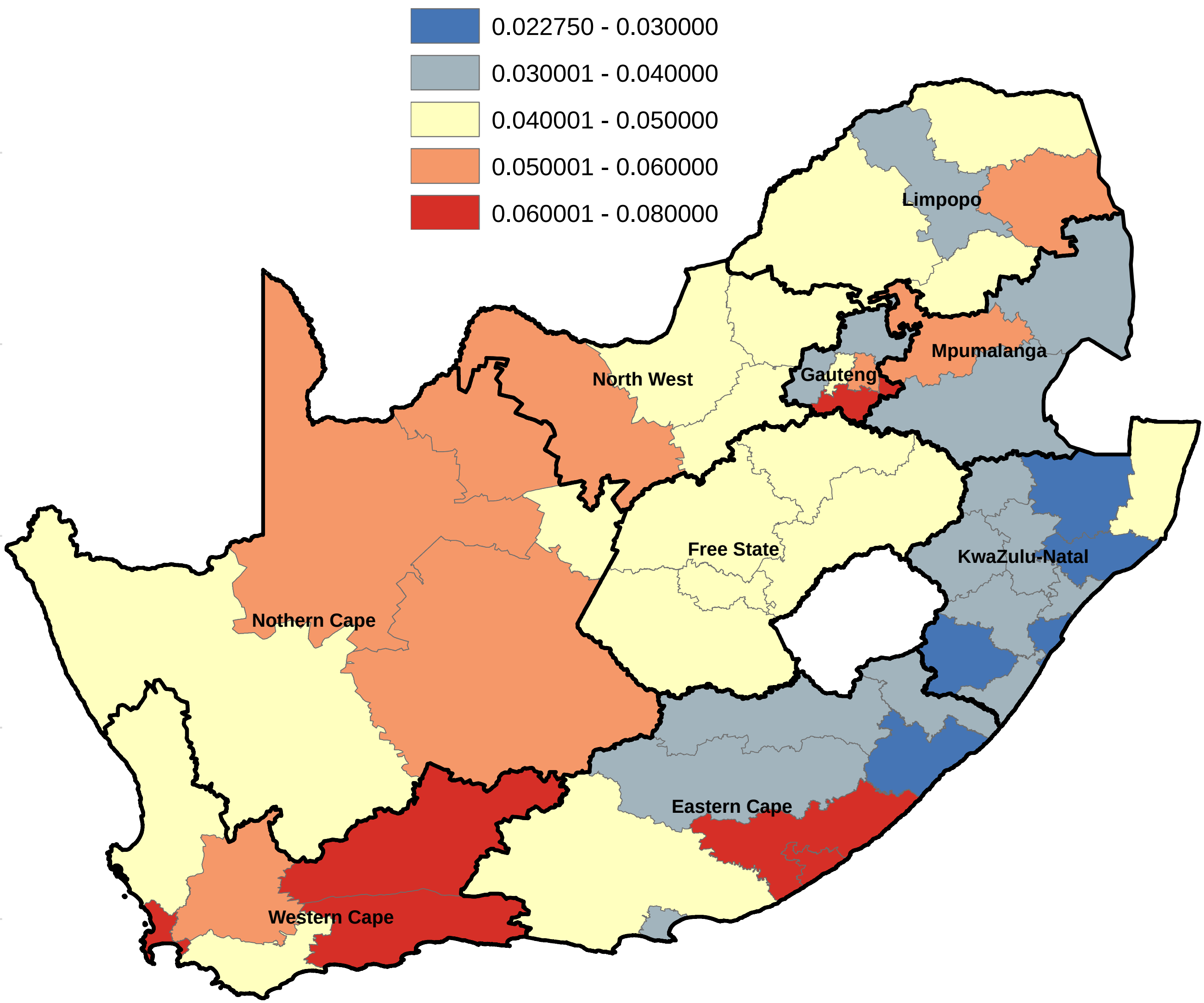

a) Obesity prevalence by province and survey wave

b) Obesity prevalence by district in 2017

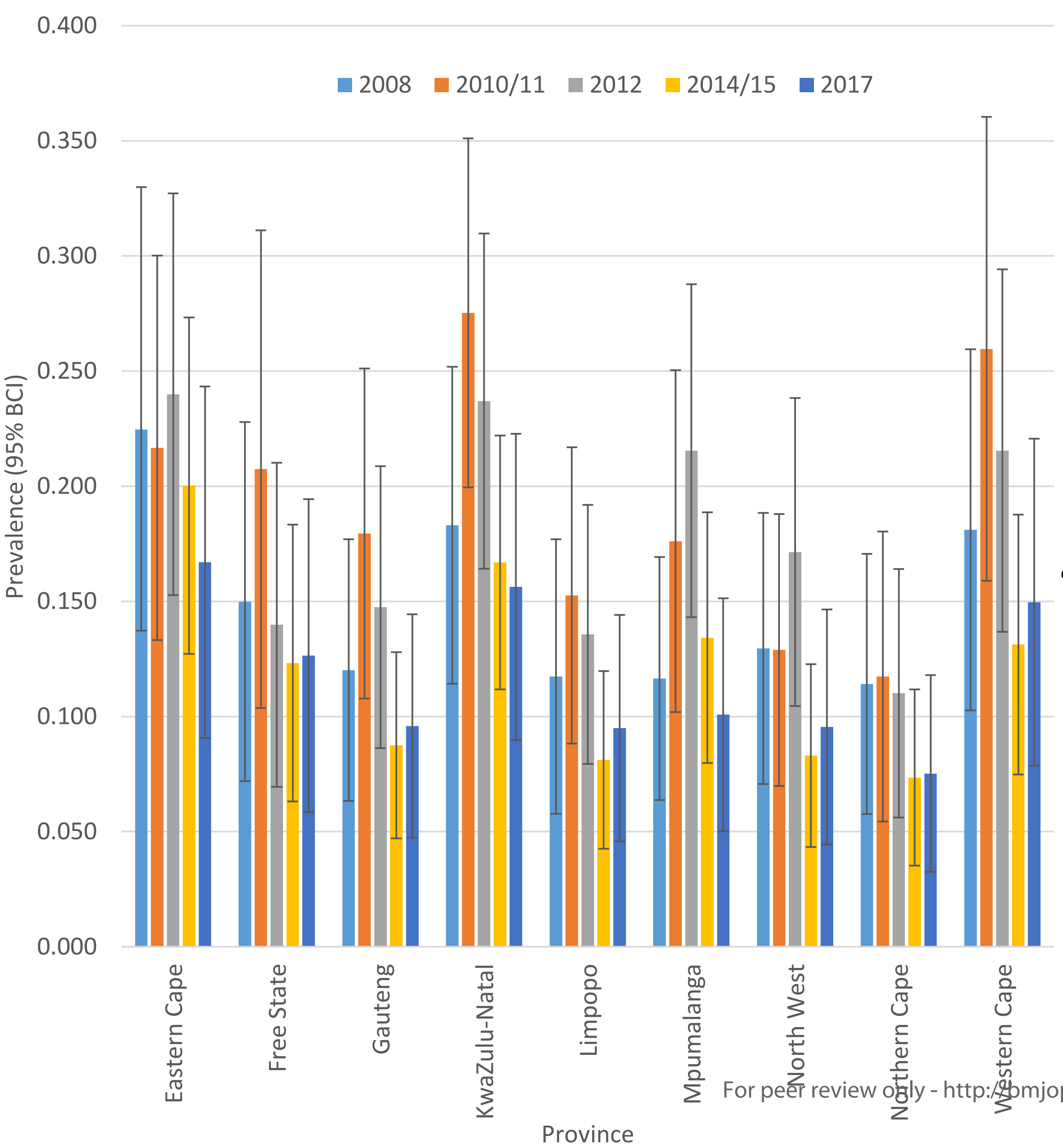

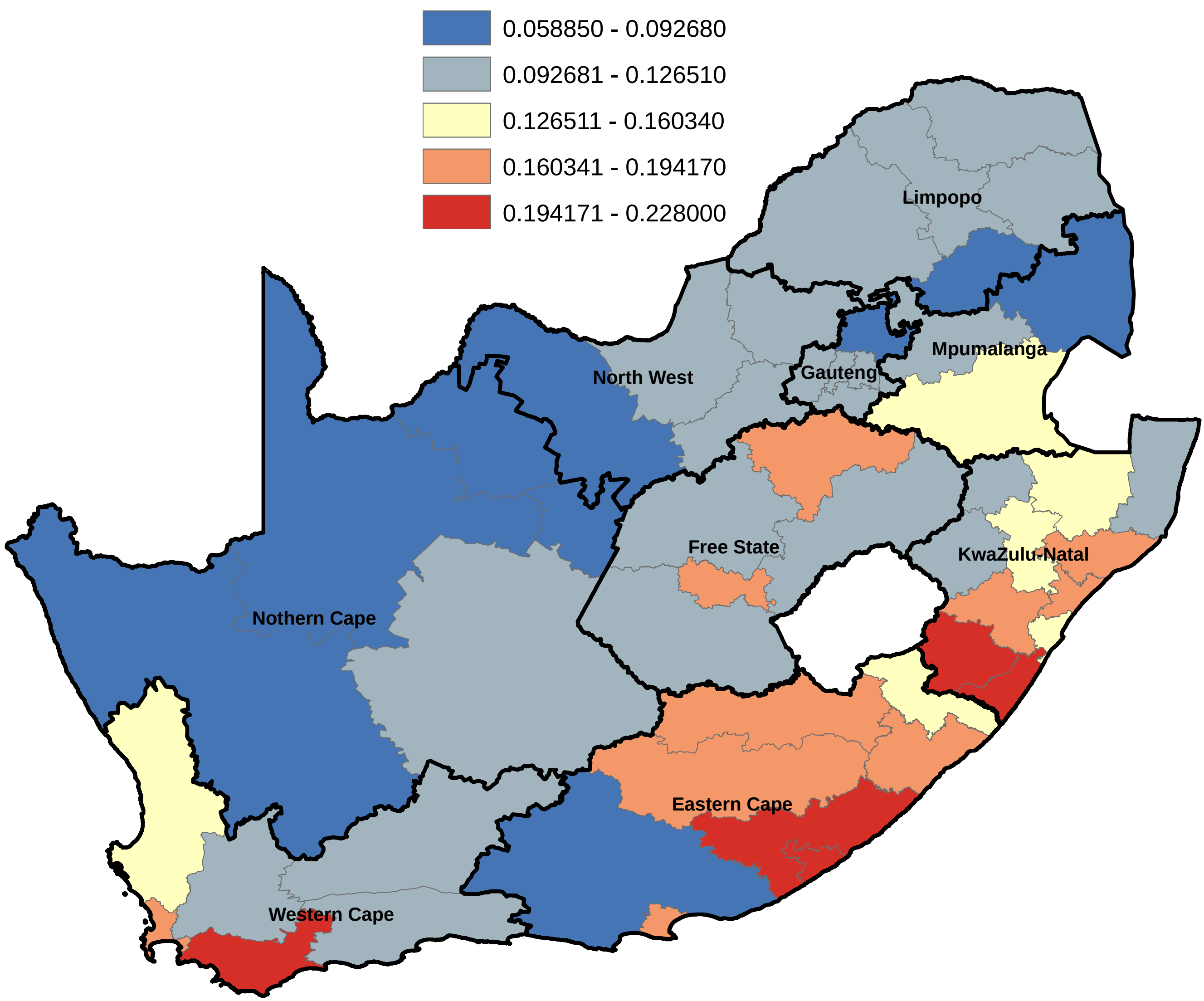

Supplementary Material

Supplementary 1: STROBE Statement—Checklist of items that should be included in reports of cross-sectional studies

	Item No	Recommendation	Page/line numbers
**Title and abstract**	1	(*a*) Indicate the study's design with a commonly used term in the title or the abstract	2/30
		(*b*) Provide in the abstract an informative and balanced summary of what was done and what was found	2/28-47
**Introduction**			
Background/rationale	2	Explain the scientific background and rationale for the investigation being reported	3-4/67-84
Objectives	3	State specific objectives, including any prespecified hypotheses	4/82-84
**Methods**			
Study design	4	Present key elements of study design early in the paper	4/89-100
Setting	5	Describe the setting, locations, and relevant dates, including periods of recruitment, exposure, follow-up, and data collection	4/89-100
Participants	6	(*a*) Give the eligibility criteria, and the sources and methods of selection of participants	4/95-102
Variables	7	Clearly define all outcomes, exposures, predictors, potential confounders, and effect modifiers. Give diagnostic criteria, if applicable	4-5/103-117
Data sources/ measurement	8*	For each variable of interest, give sources of data and details of methods of assessment (measurement). Describe comparability of assessment methods if there is more than one group	4/89-93; 5/113-117
Bias	9	Describe any efforts to address potential sources of bias	4/89-91; 8/200-2017
Study size	10	Explain how the study size was arrived at	4/89-91; Supplementary 10
Quantitative variables	11	Explain how quantitative variables were handled in the analyses. If applicable, describe which groupings were chosen and why	4-5/104-110; 5/113-117
Statistical methods	12	(*a*) Describe all statistical methods, including those used to control for confounding	5-7/118-189
		(*b*) Describe any methods used to examine subgroups and interactions	5/113-117; 7/186-189
		(*c*) Explain how missing data were addressed	8/204-207; Supplementary 7 & 8
		(*d*) If applicable, describe analytical methods taking account of sampling strategy	5/120-123; 7/172-174; 7/186-189

For peer review only

		(*e*) Describe any sensitivity analyses	8/204-205; Supplementary 4b, 8
**Results**			
Participants	13*	(a) Report numbers of individuals at each stage of study—eg numbers potentially eligible, examined for eligibility, confirmed eligible, included in the study, completing follow-up, and analysed	8/200-207; Supplementary 7
		(b) Give reasons for non-participation at each stage	8/200-207; Supplementary 7
		(c) Consider use of a flow diagram	Described using narrative text: 8/200-207; Table: Supplementary 7
Descriptive data	14*	(a) Give characteristics of study participants (eg demographic, clinical, social) and information on exposures and potential confounders	Table 1
		(b) Indicate number of participants with missing data for each variable of interest	8/200-207; Table 1; Supplementary 7
Outcome data	15*	Report numbers of outcome events or summary measures	Table 1-3
Main results	16	(*a*) Give unadjusted estimates and, if applicable, confounder-adjusted estimates and their precision (eg, 95% confidence interval). Make clear which confounders were adjusted for and why they were included	8/209-273; Table 1-3
		(*b*) Report category boundaries when continuous variables were categorized	4/104-110
		(*c*) If relevant, consider translating estimates of relative risk into absolute risk for a meaningful time period	N/A
Other analyses	17	Report other analyses done—eg analyses of subgroups and interactions, and sensitivity analyses	Supplementary 2,6,7,8,9,10
**Discussion**			
Key results	18	Summarise key results with reference to study objectives	10-11/275-283
Limitations	19	Discuss limitations of the study, taking into account sources of potential bias or imprecision. Discuss both direction and magnitude of any potential bias	14/377-386
Interpretation	20	Give a cautious overall interpretation of results considering objectives, limitations, multiplicity of analyses, results from similar studies, and other relevant evidence	11-15/296-417
Generalisability	21	Discuss the generalisability (external validity) of the study results	11-13/296-370
**Other information**			
Funding	22	Give the source of funding and the role of the funders for the present study and, if applicable, for the original study on which the present article is based	19/581-583

For peer review only

Supplementary 2: Spatial autocorrelation analyses for the 3 anthropometric outcomes (univariate and bivariate)

Pairwise correlation for anthropometric outcomes and bivariate spatial autocorrelation

We have performed additional supplementary analyses (suing GeoDa: Anselin L, Syabri I, Kho Y. GeoDa: an introduction to spatial data analysis. Geographical analysis. 2006 Jan;38(1):5-22) which assesses pairwise correlation/association between the 3 outcomes as well as bivariate Moran's I to assess if there was significant spatial autocorrelation between the outcomes. This analysis suggests that there is no significant association between stunting and thinness/wasting while there is weak positive but significant spatial autocorrelation between stunting and obesity prevalence as well as weak negative spatial correlation between thinness and obesity (please see detailed analyses below).

```
. spearman stunted_svy thin_svy

 Number of obs =      256
Spearman's rho =       0.0729

Test of Ho: stunted_svy and thin_svy are independent
    Prob > |t| =       0.2452

. gllamm stunted_svy thin_svy, i(id)

number of level 1 units = 256
number of level 2 units = 52

Condition Number = 14.594452

gllamm model

log likelihood = 283.93295

------------------------------------------------------------------------------
 stunted_svy |      Coef.   Std. Err.      z    P>|z|     [95% Conf. Interval]
-------------+----------------------------------------------------------------
    thin_svy |   .0385636   .0726234     0.53   0.595    -.1037757    .1809028
       _cons |   .1082981   .0061531    17.60   0.000     .0962381     .120358
------------------------------------------------------------------------------

Variance at level 1
------------------------------------------------------------------------------

  .00637033 (.00056306)

Variances and covariances of random effects
------------------------------------------------------------------------------

***level 2 (id)

    var(1): 2.643e-24 (5.133e-14)
------------------------------------------------------------------------------
```

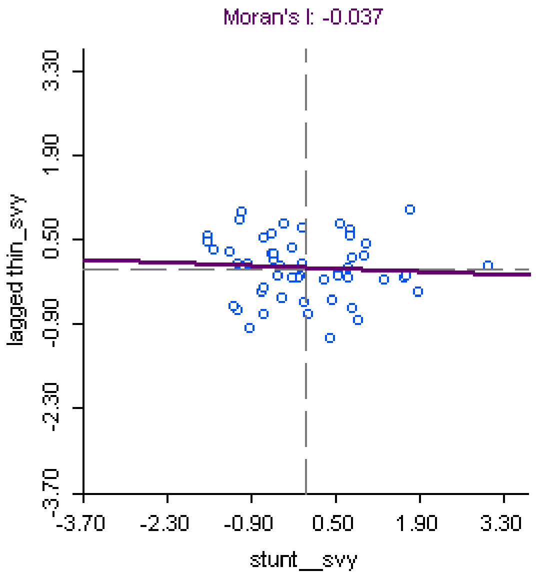

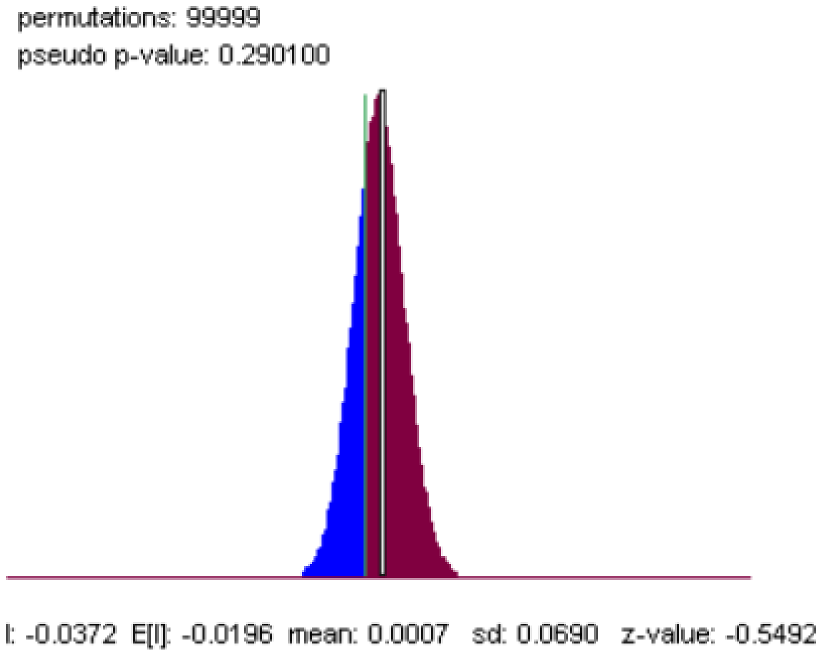


```
. spearman stunted_svy obese_svy

 Number of obs =      256
Spearman's rho =       0.2051

Test of Ho: stunted_svy and obese_svy are independent
    Prob > |t| =       0.0010

. gllamm stunted_svy obese_svy , i(id)

number of level 1 units = 256
number of level 2 units = 52

Condition Number = 10.565877

gllamm model

log likelihood = 292.58012

------------------------------------------------------------------------------
 stunted_svy |      Coef.   Std. Err.      z    P>|z|     [95% Conf. Interval]
-------------+----------------------------------------------------------------
```

```
   obese_svy |   .1980684   .0475478     4.17   0.000     .1048765    .2912604
       _cons |   .0791266   .0090305     8.76   0.000     .0614272    .0968261
------------------------------------------------------------------------------

Variance at level 1
------------------------------------------------------------------------------

  .00580379 (.00057983)

Variances and covariances of random effects
------------------------------------------------------------------------------

***level 2 (id)

    var(1): .00015837 (.00029997)
------------------------------------------------------------------------------
```

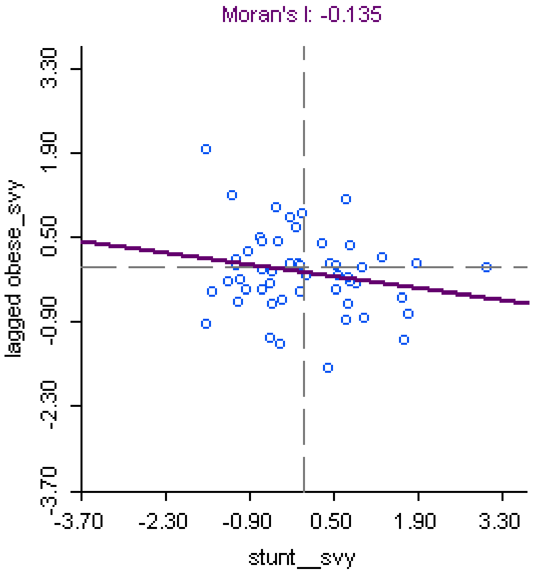

permutations: 99999
pseudo p-value: 0.025380

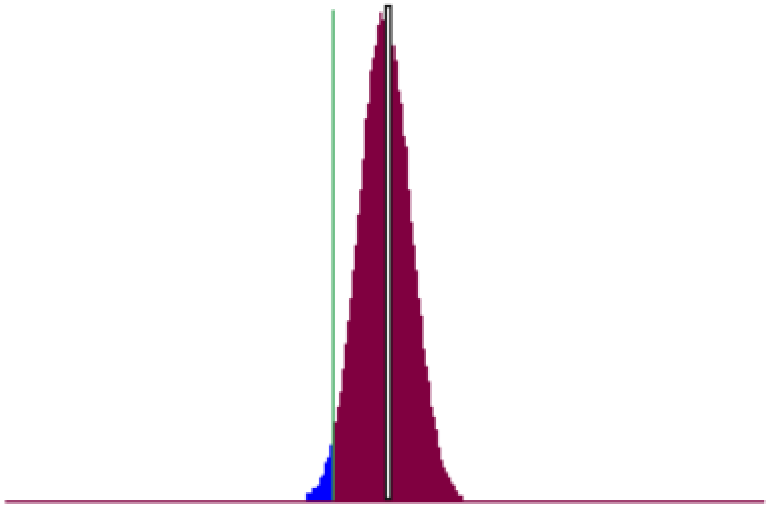

I: -0.1350 E[I]: -0.0196 mean: 0.0001 sd: 0.0689 z-value: -1.9600

```
. spearman thin_svy obese_svy

 Number of obs =     256
Spearman's rho =      -0.1424

Test of Ho: thin_svy and obese_svy are independent
```

```
    Prob > |t| =       0.0227

. gllamm thin_svy obese_svy , i(id)

number of level 1 units = 256
number of level 2 units = 52

Condition Number = 10.976401

gllamm model

log likelihood = 324.36079

------------------------------------------------------------------------------
    thin_svy |      Coef.   Std. Err.      z    P>|z|     [95% Conf. Interval]
-------------+----------------------------------------------------------------
   obese_svy |   -.067802    .040258    -1.68   0.092    -.1467062    .0111022
       _cons |   .0602269   .0078037     7.72   0.000     .0449319    .0755218
------------------------------------------------------------------------------

Variance at level 1
------------------------------------------------------------------------------

  .00447574 (.00044278)

Variances and covariances of random effects
------------------------------------------------------------------------------

***level 2 (id)

    var(1): .00018259 (.00023176)
------------------------------------------------------------------------------
```

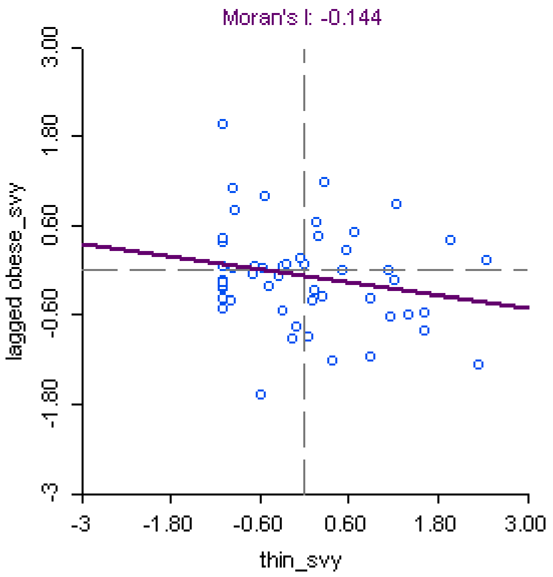

For peer review only

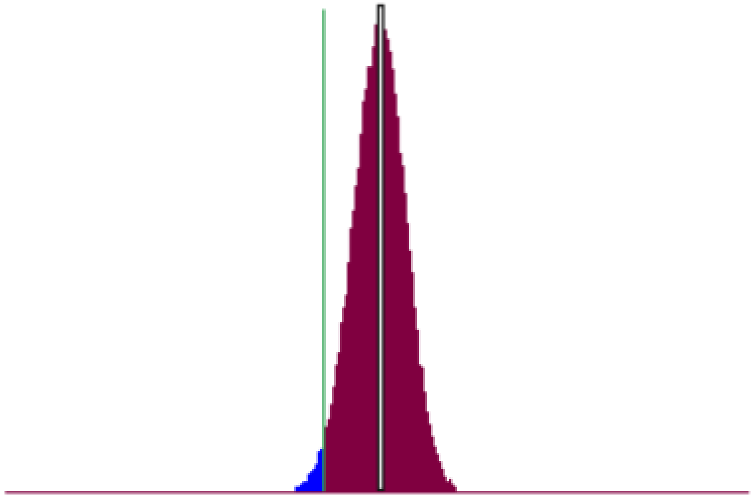

With regards to the shared temporal effect this we think can be retained as all 3 outcomes appear to have a negative coefficient associated with increasing panel or wave.

```
. gllamm stunted_svy year , i(id)

number of level 1 units = 256
number of level 2 units = 52

Condition Number = 31.724715

gllamm model

log likelihood = 293.64743

------------------------------------------------------------------------------
 stunted_svy |      Coef.   Std. Err.      z    P>|z|     [95% Conf. Interval]
-------------+----------------------------------------------------------------
        year |  -.0153423   .0033894    -4.53   0.000    -.0219855   -.0086992
       _cons |   .1563577   .0112694    13.87   0.000     .1342702    .1784453
------------------------------------------------------------------------------

Variance at level 1
------------------------------------------------------------------------------

  .00590475 (.00052191)

Variances and covariances of random effects
------------------------------------------------------------------------------


***level 2 (id)

    var(1): 8.887e-19 (4.854e-11)
------------------------------------------------------------------------------

. gllamm thin_svy year , i(id)

number of level 1 units = 256
number of level 2 units = 52

Condition Number = 37.175479

gllamm model

log likelihood = 327.11892

------------------------------------------------------------------------------
```

```
    thin_svy |      Coef.   Std. Err.      z    P>|z|     [95% Conf. Interval]
-------------+----------------------------------------------------------------
        year |  -.0084373   .0028941    -2.92   0.004    -.0141096    -.002765
       _cons |   .0749857   .0098979     7.58   0.000     .0555862    .0943852
------------------------------------------------------------------------------

Variance at level 1
------------------------------------------------------------------------------

  .00430301 (.00042507)

Variances and covariances of random effects
------------------------------------------------------------------------------


***level 2 (id)

    var(1): .00027197 (.0002388)
------------------------------------------------------------------------------

. gllamm obese_svy year , i(id)

number of level 1 units = 256
number of level 2 units = 52

Condition Number = 21.597249

gllamm model

log likelihood = 215.4003

------------------------------------------------------------------------------
   obese_svy |      Coef.   Std. Err.      z    P>|z|     [95% Conf. Interval]
-------------+----------------------------------------------------------------
        year |  -.0112194   .0043125    -2.60   0.009    -.0196717   -.0027671
       _cons |   .1905201   .0155017    12.29   0.000     .1601374    .2209029
------------------------------------------------------------------------------

Variance at level 1
------------------------------------------------------------------------------

  .00954712 (.00094327)

Variances and covariances of random effects
------------------------------------------------------------------------------

***level 2 (id)

    var(1): .00175973 (.00074487)
------------------------------------------------------------------------------
```

Univariate spatial autocorrelation

Based on the univariate Moran's I statistics for each anthropometric outcome there appeared to be significant spatial heterogeneity present for all 3 outcomes.

For peer review only

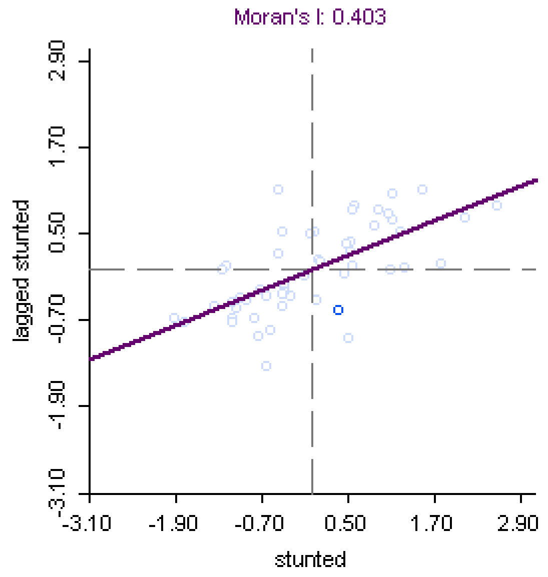
Moran's I: 0.403
lagged stunted
stunted
-3.10 -1.90 -0.70 0.50 1.70 2.90

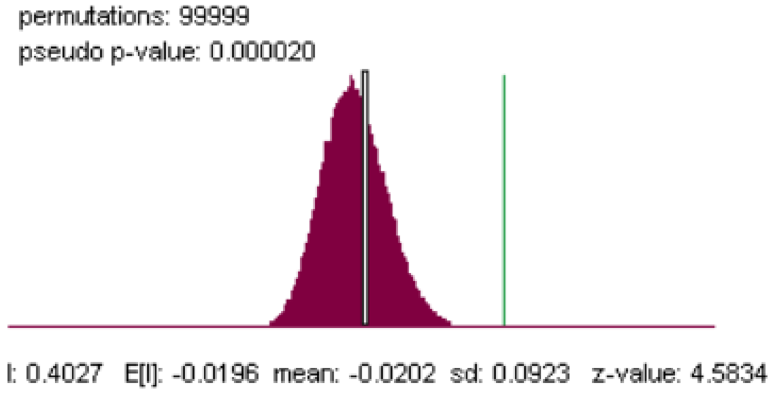
permutations: 99999
pseudo p-value: 0.000020
I: 0.4027 E[I]: -0.0196 mean: -0.0202 sd: 0.0923 z-value: 4.5834

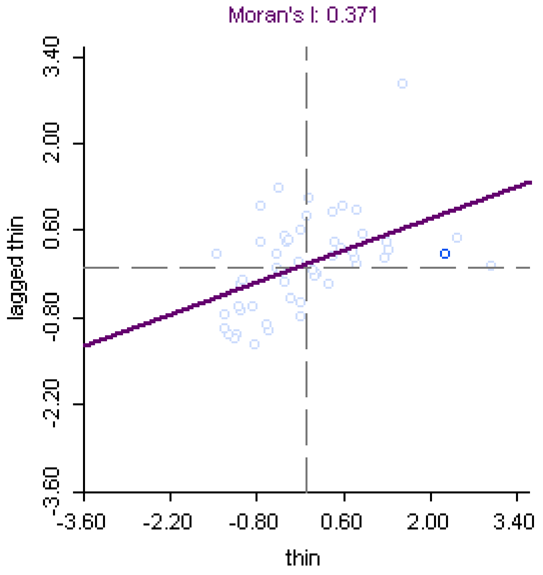
Moran's I: 0.371
lagged thin
thin
-3.60 -2.20 -0.80 0.60 2.00 3.40

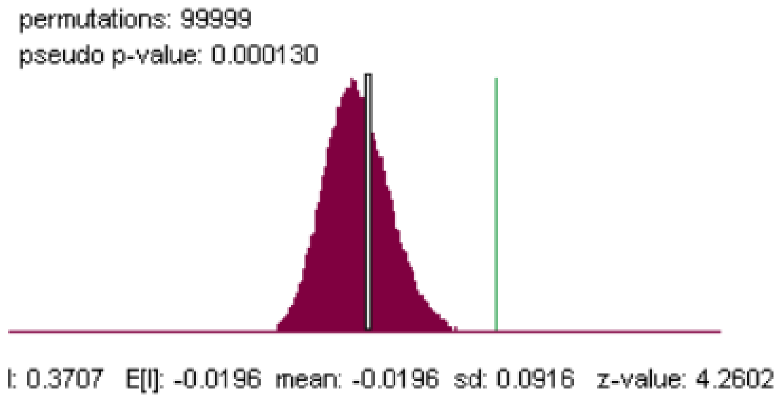

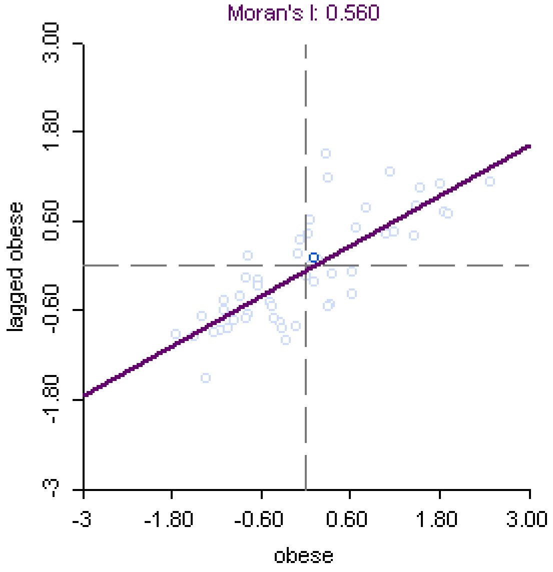

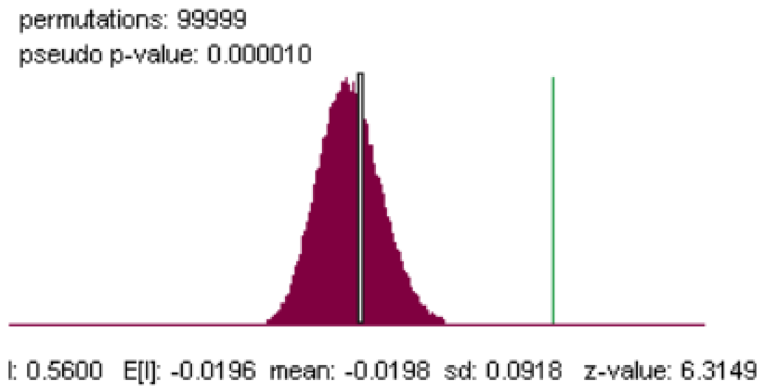

For peer review only - http://bmjopen.bmj.com/site/about/guidelines.xhtml

Supplementary 3: Win BUGS code for Bayesian space-time binomial model

```
model
{
	for( i in 1 : N ) {
		for( j in 1 : T ) {
		#Likelihood

		stunted[i,j] ~ dbin(p1[i,j],child[i,j])
		logit(p1[i,j])<-alpha1+phi1[i]+gamma1[j]+nu1[i,j]

		thin[i,j] ~ dbin(p2[i,j],child[i,j])
		logit(p2[i,j])<-alpha2+phi2[i]+gamma2[j]+nu2[i,j]
		exceedance2[i,j]<-step(p2[i,j]-0.05)	# reduce and maintain wasting to <5%

		obese[i,j] ~ dbin(p3[i,j],child[i,j])
		logit(p3[i,j])<-alpha3+phi3[i]+gamma3[j]+nu3[i,j]
		}
	exceedance1[i,5]<-step((1-p1[i,5]/p1[i,3])-0.17) #17% is target reduction by 2017 from 2012
							# assuming target 40% reduction by 2025
	exceedance3[i,5]<-step(p3[i,5]/p3[i,3]-1)		# no increase in obesity from 2012 to 2017
	}

# - Space
phi1[1:52]~car.normal(adj[],weights[],num[],tau.phi[1])
phi2[1:52]~car.normal(adj[],weights[],num[],tau.phi[2])
phi3[1:52]~car.normal(adj[],weights[],num[],tau.phi[3])

for(k in 1:240) {weights[k]<-1}

# - Time:

gamma1[1:T]~car.normal(adj.t[],weights.t[],num.t[],tau.gamma[1])
gamma2[1:T]~car.normal(adj.t[],weights.t[],num.t[],tau.gamma[2])
gamma3[1:T]~car.normal(adj.t[],weights.t[],num.t[],tau.gamma[3])

for(t in 1:1) {
	weights.t[t] <- 1;
	adj.t[t] <- t+1;
	num.t[t] <- 1
	}
for(t in 2:(T-1)) {
	weights.t[2+(t-2)*2] <- 1;
	adj.t[2+(t-2)*2] <- t-1
	weights.t[3+(t-2)*2] <- 1;
	adj.t[3+(t-2)*2] <- t+1;
	num.t[t] <- 2
	}
for(t in T:T) {
	weights.t[(T-2)*2 + 2] <- 1;
	adj.t[(T-2)*2 + 2] <- t-1;
	num.t[t] <- 1
	}

#Space-time Interaction terms
for(i in 1:N){
	for(j in 1:T){
	nu1[i,j]~dnorm(0, tau.nu[1])
	nu2[i,j]~dnorm(0, tau.nu[2])
	nu3[i,j]~dnorm(0, tau.nu[3])
```

For peer review only

```
        }
}

#Hyperprior specification

for(i in 1:3){
tau.phi[i]~dgamma(0.5, 0.0005)
tau.gamma[i]~dgamma(0.5, 0.0005)
tau.nu[i]~dgamma(0.5, 0.0005)
}

alpha1~dflat()
alpha2~dflat()
alpha3~dflat()

}
}
```

For peer review only

Supplementary 4: a) Model random effects posteriors and b) sensitivity analysis of hyper parameter selection

a)

Spatial random effects (phi)

Stunting

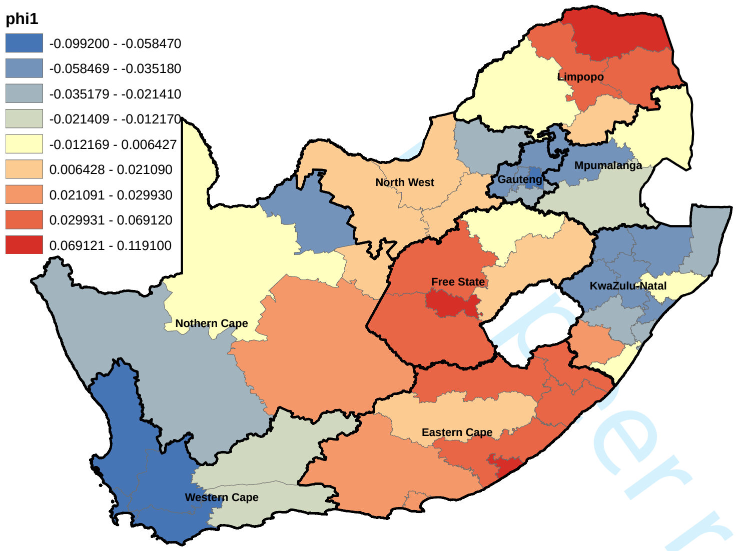

Thinness/wasting

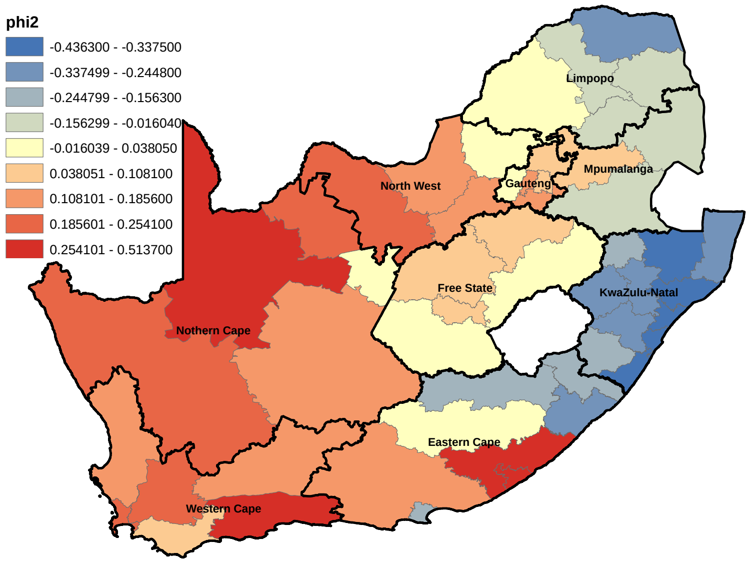

Obesity

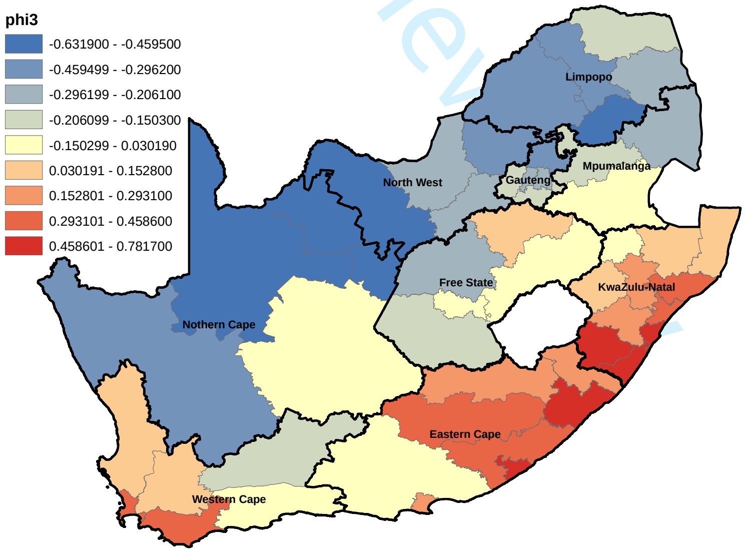

Unstructured effects (2017) (nu)

Stunting

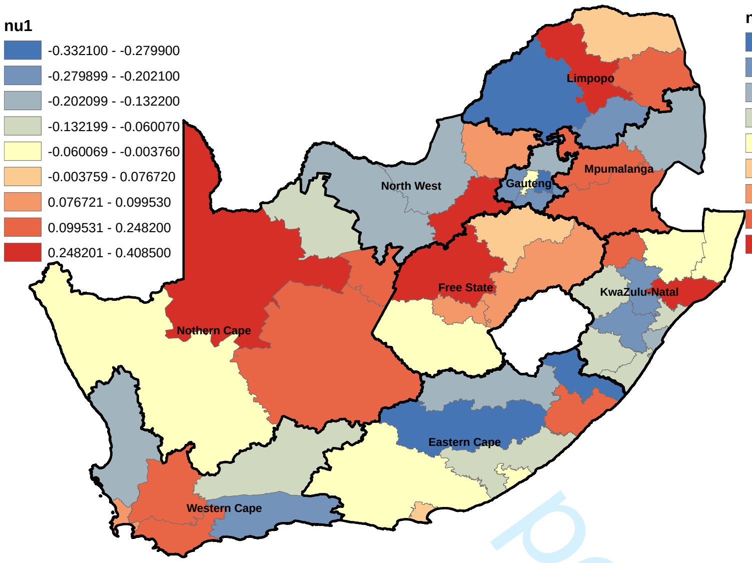

Thinness/wasting

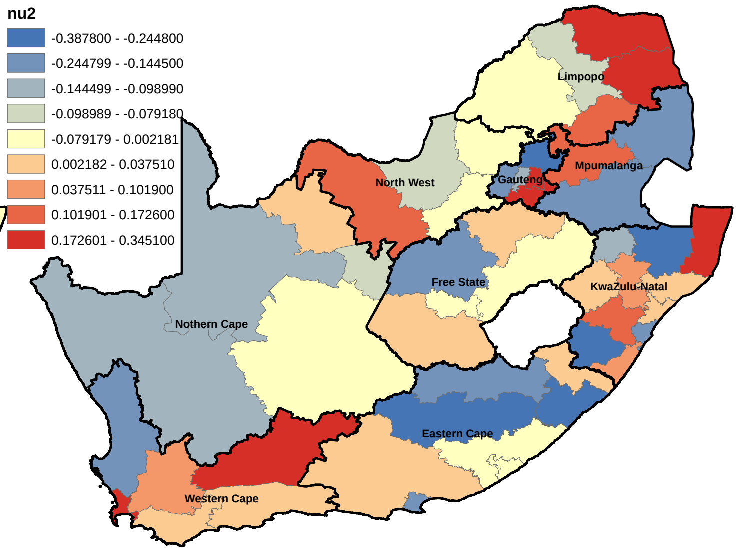

Obesity

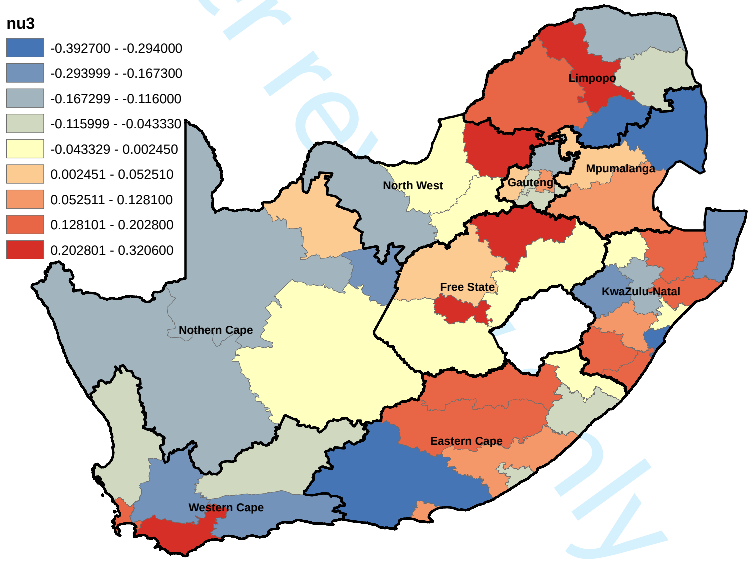

Temporal random effects (gamma)

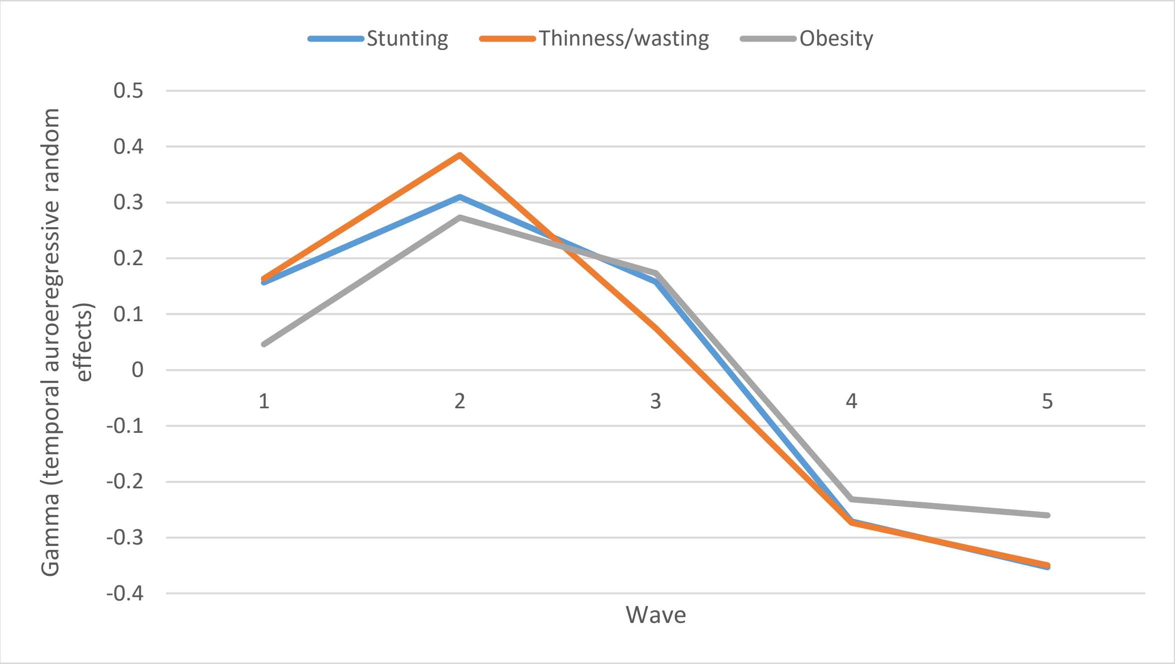

b)

We concluded an additional sensitivity analysis to confirm whether the choice of hyper parameter may have affected the prevalence estimates. For the variance parameters, namely σ^2_ν, σ^2_ϕ, σ^2_γ we assumed Gamma(0.5,0.0005) distributions as recommended by Wakefield (Wakefield J, Best N, Waller L. Bayesian approaches to disease mapping. Spatial epidemiology: methods and applications 2000:104-07.) for the Baysian prevalence/exceedance probability estimates presented in the main text. We also tested whether changes to this prior may have affected the estimates. Other choices for this prior (Lawson A, Browne W, Vidal Rodeiro C. Disease Mapping with WinBUGS and MLWin. Chichester: John Wiley & Sons; 2003) that are commonly used include.

Gamma (0.001, 0.001)

Gamma (0.01,0.01)

Pairwise scatterplots of the posterior prevalence for the various gamma distribution choices for the hyper parameters below suggest that the model estimates were largely insensitive to the choice of distribution assumed:

For peer review only - http://bmjopen.bmj.com/site/about/guidelines.xhtml

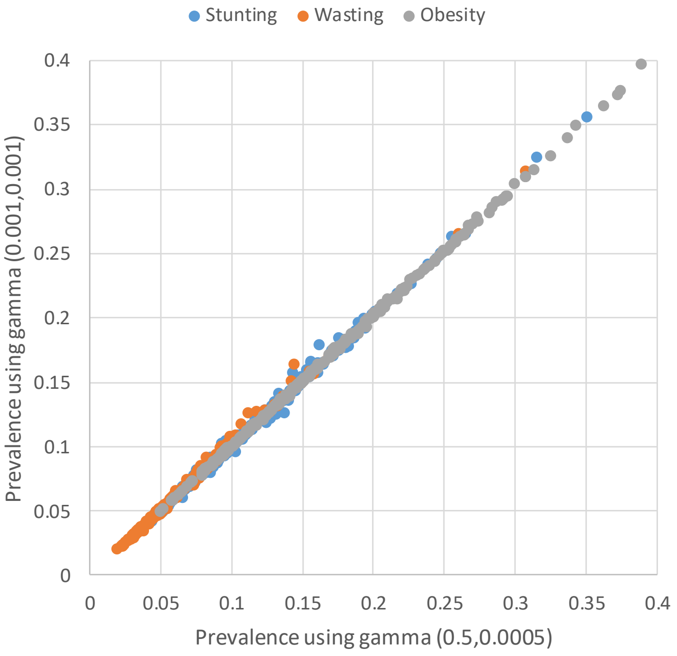
Stunting
Wasting
Obesity
Prevalence using gamma (0.001,0.001)
Prevalence using gamma (0.5,0.0005)
0
0.05
0.1
0.15
0.2
0.25
0.3
0.35
0.4

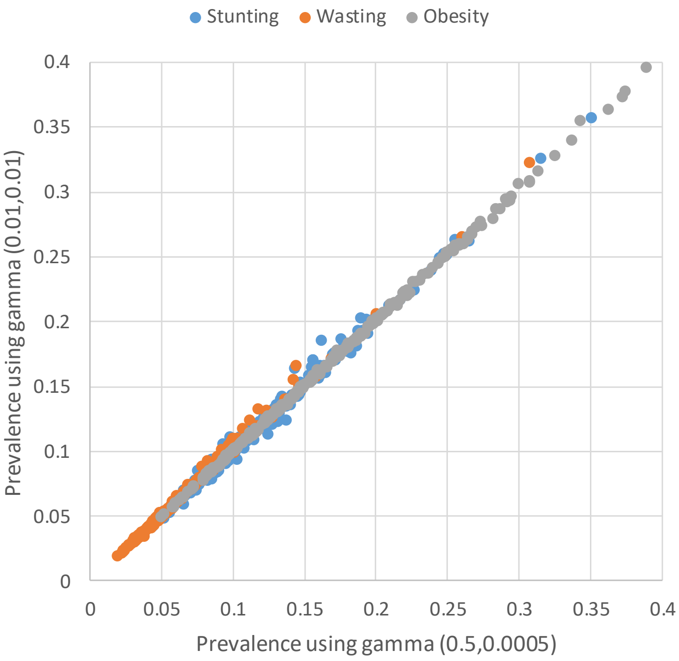
Stunting
Wasting
Obesity
Prevalence using gamma (0.01,0.01)
Prevalence using gamma (0.5,0.0005)
0
0.05
0.1
0.15
0.2
0.25
0.3
0.35
0.4

Supplementary 5: a) Model convergence [Gelman-Rubin statistics/plots]

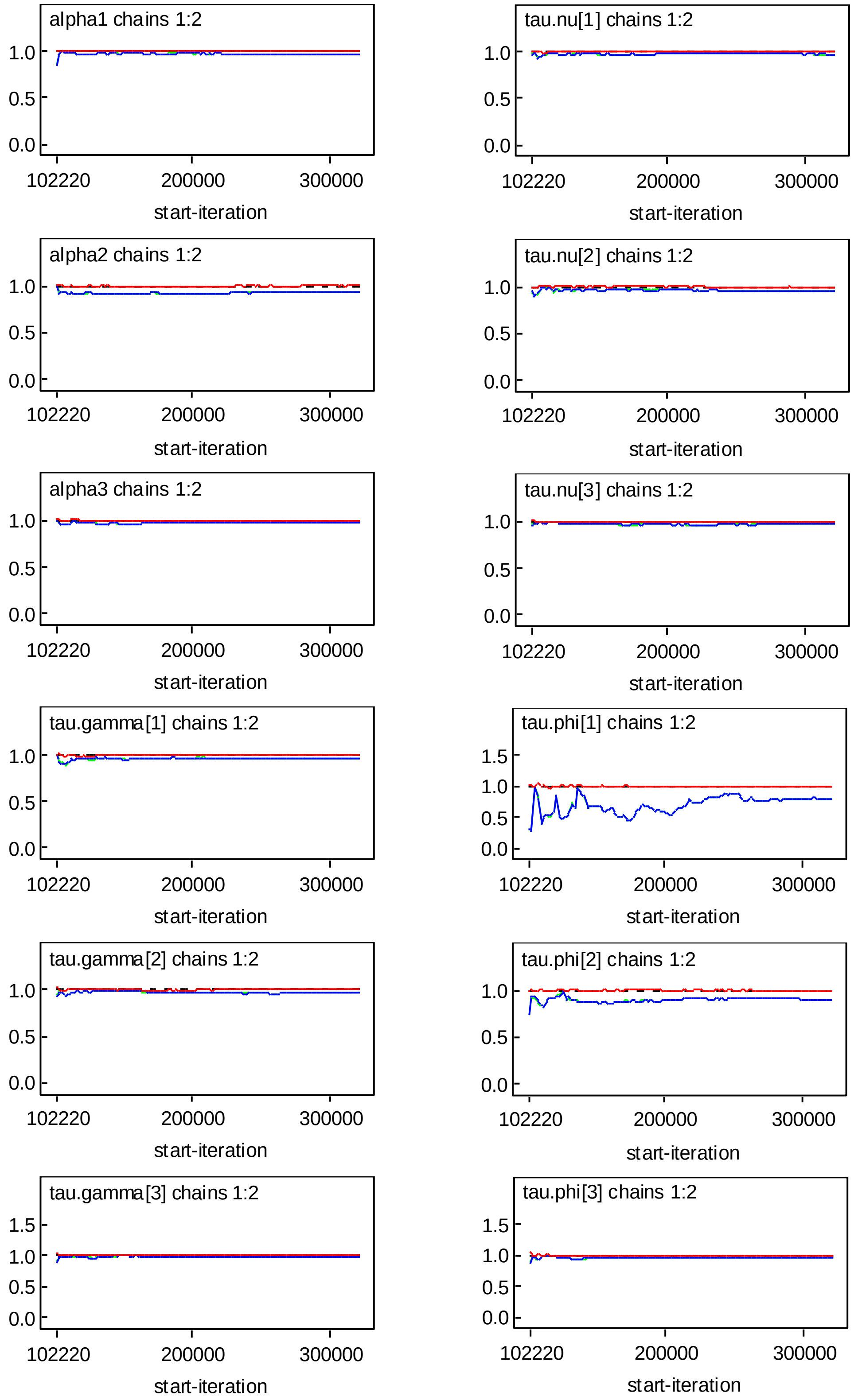

Supplementary 6: Model fit and out of sample validation

Overall model fit

Dbar = post.mean of -2logL; Dhat = -2LogL at post.mean of stochastic nodes

	Dbar	Dhat	pD	DIC
obese	1110.400	969.250	141.149	1251.550
stunted	1036.090	910.101	125.987	1162.080
thin	695.343	602.042	93.301	788.643
total	2841.830	2481.390	360.437	3202.270

Comparison of survey prevalence versus model fitted prevalence by anthropometric measure

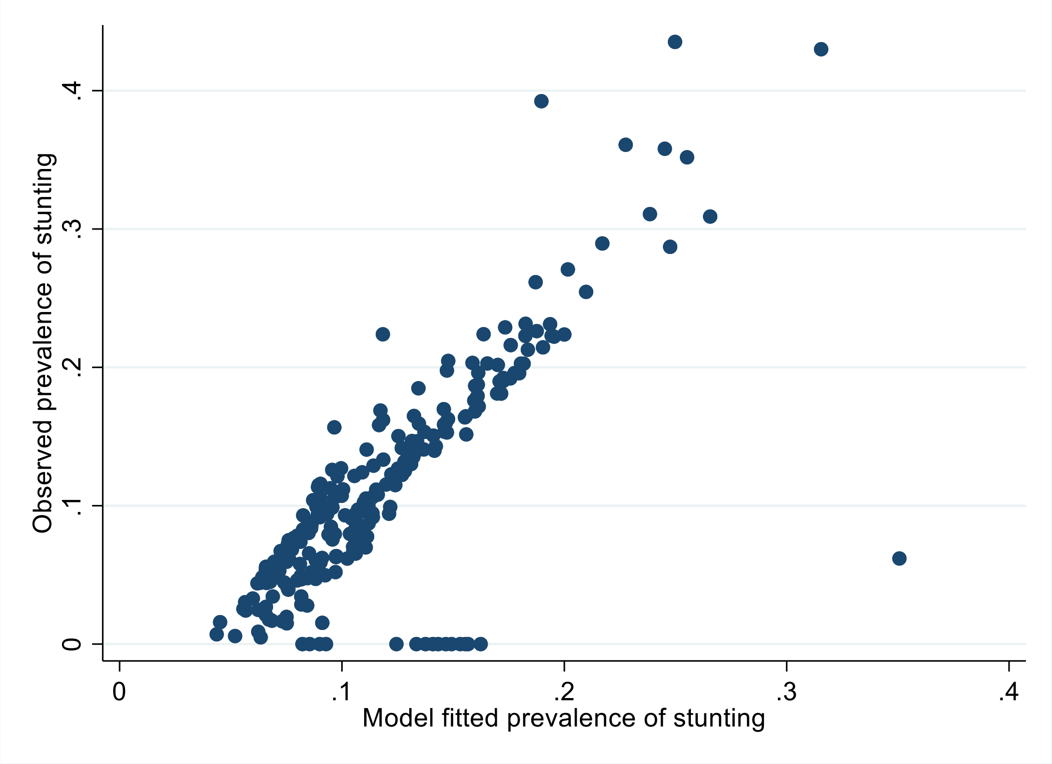


```
. spearman stunted_svy p1 if stunted_svy~=0

 Number of obs =     241
Spearman's rho =       0.9190

Test of Ho: stunted_svy and p1 are independent
    Prob > |t| =       0.0000

. spearman stunted_svy p1

 Number of obs =     256
Spearman's rho =       0.7729

Test of Ho: stunted_svy and p1 are independent
    Prob > |t| =       0.0000
```

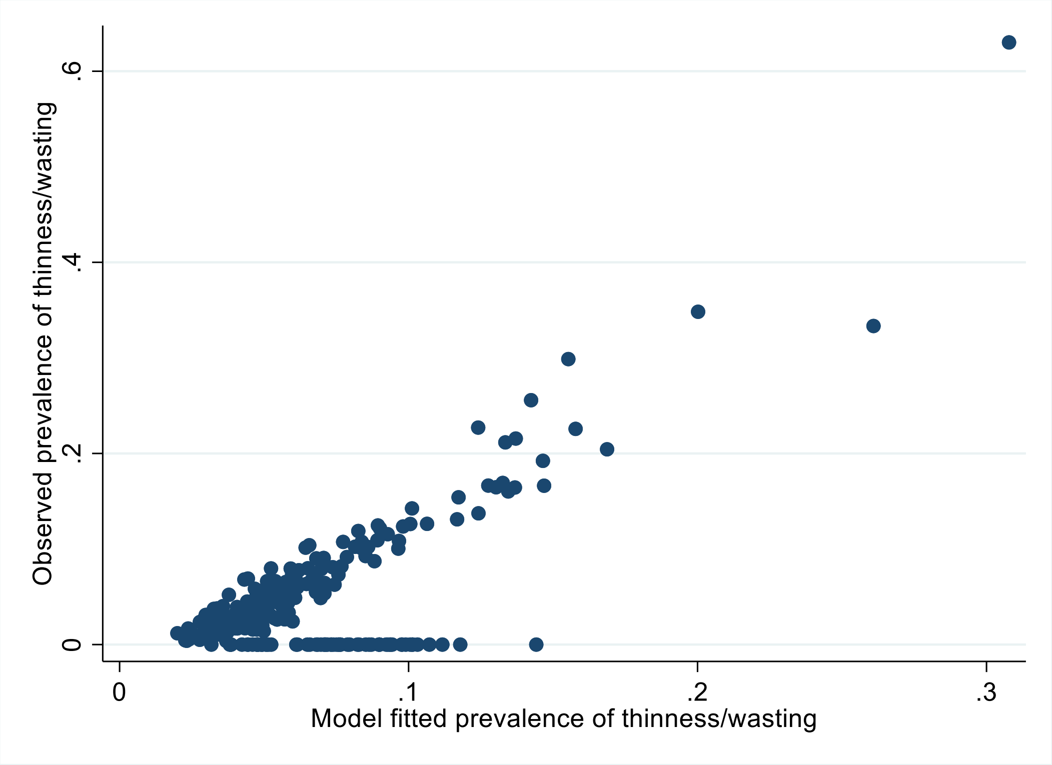


```
. spearman thin_svy p2 if thin_svy~=0

 Number of obs =     191
Spearman's rho =       0.9019

Test of Ho: thin_svy and p2 are independent
    Prob > |t| =       0.0000

. spearman thin_svy p2

 Number of obs =     256
Spearman's rho =       0.2972

Test of Ho: thin_svy and p2 are independent
    Prob > |t| =       0.0000
```

For peer review only

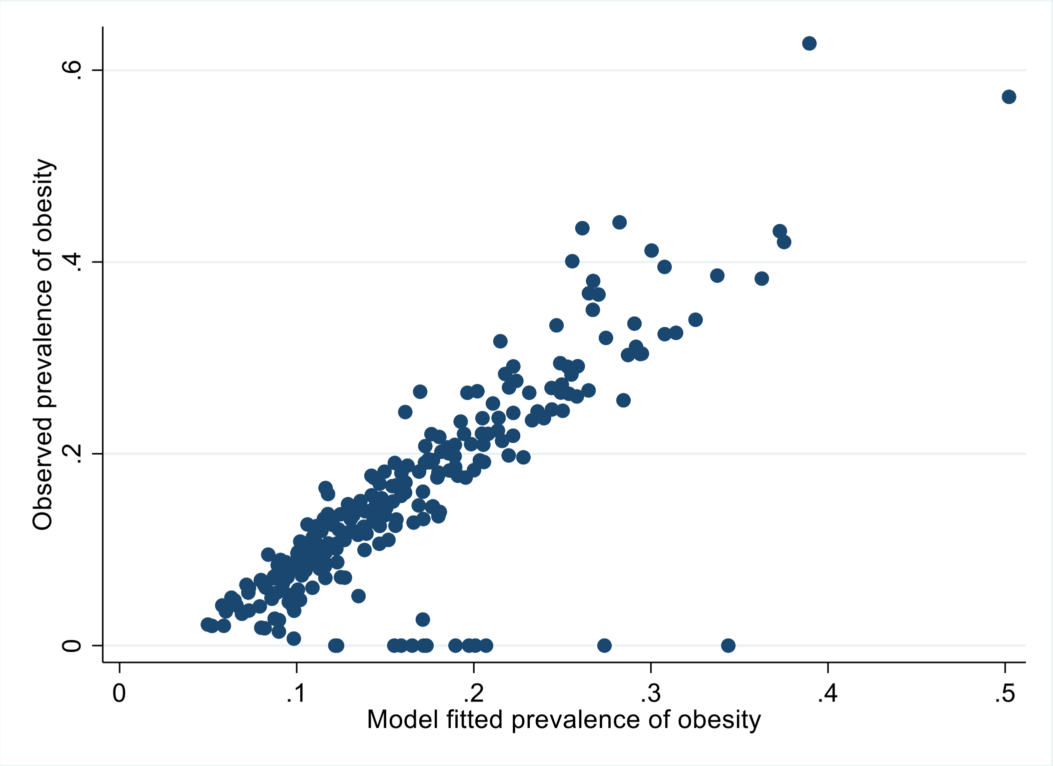


```
. spearman obese_svy p3 if obese_svy~=0

 Number of obs =     243
Spearman's rho =       0.9485

Test of Ho: obese_svy and p3 are independent
    Prob > |t| =       0.0000

. spearman obese_svy p3

 Number of obs =     256
Spearman's rho =       0.8179

Test of Ho: obese_svy and p3 are independent
    Prob > |t| =       0.0000
```

Out of sample validation/prediction (10% random sample)

Of the 37 out of sample validation points, 31 (or 84%) of the observed prevalence of stunting where within the 95% uncertainty interval for the predicted posterior prevalence, 28/37 (78%) for thinness/wasting and 31/37 (84%) for obesity.

id	wave	District	Prov	Stunted (observed)	Thin/wasted (observed)	Obese (observed)	Stunted (posterior)	95% BCI		Thin/ wasted (posterior)	95% BCI		Obese (posterior)	95% BCI	
3	4	City of Tshwane (TSH)	Gauteng	0.01584	0.028309	0.03565	0.09138	0.0344	0.1911	0.05339	0.01411	0.1343	0.1104	0.04251	0.226
4	1	City of Johannesburg (JHB)	Gauteng	0.075611	0.015782	0.187624	0.1285	0.05006	0.26	0.08426	0.0217	0.2094	0.1389	0.05482	0.2789
5	1	Buffalo City (BUF)	Eastern Cape	0.392359	0.255737	0.255737	0.1366	0.04961	0.2864	0.105	0.02015	0.3356	0.3173	0.1231	0.5788
8	3	Cacadu (DC10)	Eastern Cape	0.063566	0.063563	0.071285	0.1308	0.05085	0.261	0.07077	0.01873	0.1759	0.1926	0.07914	0.3611
9	5	Amathole (DC12)	Eastern Cape	0.034391	0.048998	0.263574	0.08987	0.03286	0.1895	0.0618	0.01346	0.1731	0.1882	0.07342	0.363
12	1	O.R.Tambo (DC15)	Eastern Cape	0.222196	0.015888	0.246151	0.138	0.05288	0.2758	0.06349	0.01635	0.1665	0.2552	0.111	0.4522
13	3	Xhariep (DC16)	Free State	0.052021	0.022786	0.086915	0.1352	0.0517	0.2722	0.06617	0.01758	0.1673	0.1631	0.06518	0.3172
14	4	Lejweleputswa (DC18)	Free State	0.164856	0.059159	0.08698	0.09708	0.0363	0.2027	0.0539	0.01409	0.1363	0.1095	0.04191	0.2254
15	3	Thabo Mofutsanyane (DC19)	Free State	0.228885	0.030611	0.106143	0.1284	0.04956	0.2567	0.06452	0.0173	0.1614	0.1861	0.07662	0.353

16	5	Cape Winelands (DC2)	Western Cape	0.125904	0.065507	0.089544	0.08173	0.02978	0.176	0.05496	0.01371	0.1417	0.1369	0.05382	0.2708
19	3	UMgungundlovu (DC22)	KwaZulu-Natal	0.133616	0.107423	0.213388	0.1224	0.0468	0.2492	0.05064	0.01301	0.136	0.2251	0.0955	0.4095
20	3	Uthukela (DC23)	KwaZulu-Natal	0.140663	0.029259	0.244155	0.1202	0.04537	0.2464	0.05216	0.01372	0.1382	0.1857	0.07645	0.3524
21	5	Umzinyathi (DC24)	KwaZulu-Natal	0.045311	0.037798	0.117828	0.08302	0.03081	0.1783	0.03554	0.008943	0.09847	0.1524	0.05855	0.3055
23	4	Zululand (DC26)	KwaZulu-Natal	0.082797	0.026059	0.193396	0.08843	0.03282	0.1885	0.03922	0.009809	0.1077	0.1404	0.0551	0.2804
25	3	Uthungulu (DC28)	KwaZulu-Natal	0.178442	0.022595	0.239129	0.1247	0.04783	0.251	0.0497	0.01282	0.1345	0.2337	0.09804	0.4257
26	3	iLembe (DC29)	KwaZulu-Natal	0.196082	0.045007	0.198271	0.1188	0.04489	0.2454	0.04939	0.01221	0.1343	0.2598	0.1132	0.4611
27	4	Overberg (DC3)	Western Cape	0.044303	0.006543	0.22102	0.08827	0.03194	0.1892	0.05854	0.01459	0.1527	0.1828	0.07273	0.3521
28	1	Gert Sibande (DC30)	Mpumalanga	0.039364	0.016922	0.087611	0.1301	0.05057	0.2615	0.06794	0.0188	0.1708	0.1692	0.06952	0.3252
28	3	Gert Sibande (DC30)	Mpumalanga	0.13142	0.169157	0.242636	0.126	0.04846	0.252	0.05976	0.01619	0.1517	0.1798	0.07408	0.3441
30	2	Ehlanzeni (DC32)	Mpumalanga	0.082747	0.049337	0.118953	0.1462	0.05729	0.2896	0.07806	0.02038	0.2002	0.1458	0.05572	0.2908
30	3	Ehlanzeni (DC32)	Mpumalanga	0.308996	0.102135	0.263661	0.1244	0.04733	0.252	0.05775	0.01508	0.1496	0.1336	0.05113	0.2685
32	4	Vhembe (DC34)	Limpopo	0.159305	0.005527	0.060438	0.1034	0.03696	0.2201	0.04615	0.01114	0.1254	0.127	0.04623	0.2623
33	4	Capricorn (DC35)	Limpopo	0.098606	0.024444	0.137144	0.09882	0.0363	0.2066	0.05008	0.0131	0.1289	0.09301	0.0339	0.198
35	1	Bojanala (DC37)	North West	0.061806	0.026407	0.041916	0.1308	0.05145	0.2631	0.07596	0.01993	0.1909	0.1472	0.05754	0.2922
35	3	Bojanala (DC37)	North West	0.051943	0.050775	0.07316	0.127	0.0494	0.2549	0.06689	0.01764	0.1705	0.1567	0.06121	0.3078
36	4	Ngaka Modiri Molema (DC38)	North West	0.098734	0.085696	0.112994	0.09498	0.03531	0.1977	0.05315	0.01375	0.1366	0.1163	0.04431	0.2389
37	4	Dr Ruth Segomotsi Mompati (DC39)	North West	0.074933	0.081794	0.020508	0.09534	0.03553	0.199	0.05775	0.01489	0.1462	0.09674	0.03614	0.2034
40	1	Sedibeng (DC42)	Gauteng	0.202795	0.225723	0.106432	0.1281	0.04987	0.26	0.07473	0.02019	0.1852	0.1554	0.06286	0.3048
40	2	Sedibeng (DC42)	Gauteng	0.152953	0.100409	0.132566	0.1455	0.05777	0.2879	0.08836	0.02332	0.2164	0.1801	0.07327	0.3438
41	4	Sisonke (DC43)	KwaZulu-Natal	0.058274	0.010655	0.058335	0.09652	0.03541	0.2031	0.04685	0.01187	0.123	0.2282	0.09509	0.4207
42	5	Alfred Nzo (DC44)	Eastern Cape	0.020967	0.040195	0.143771	0.09156	0.03377	0.1927	0.03907	0.009915	0.1059	0.1643	0.06519	0.3221
45	1	West Rand (DC48)	Gauteng	0.085192	0.124663	0.07049	0.1293	0.05015	0.2589	0.07178	0.01918	0.1799	0.1645	0.06532	0.3235
46	5	Central Karoo (DC5)	Western Cape	0.059525	0.10142	0.080496	0.08649	0.0322	0.1821	0.05009	0.01282	0.1309	0.1124	0.04168	0.2323
51	1	Ekurhuleni (EKU)	Gauteng	0.146754	0.026242	0.026557	0.1237	0.04661	0.2538	0.07674	0.02017	0.1912	0.1587	0.06215	0.3162
51	4	Ekurhuleni (EKU)	Gauteng	0.007073	0.079849	0.08599	0.08755	0.03179	0.1863	0.05287	0.01372	0.1358	0.1248	0.0475	0.2561
52	4	eThekwini (ETH)	KwaZulu-Natal	0.141696	0.011844	0.311557	0.08787	0.03228	0.1884	0.03885	0.009519	0.1088	0.196	0.07941	0.3735
52	5	eThekwini (ETH)	KwaZulu-Natal	0.055993	0.016832	0.124759	0.08245	0.03015	0.1764	0.0343	0.008309	0.09693	0.1901	0.07589	0.3647

```
. spearman stunted_svy stuntedvpost if validation_sample2==1

 Number of obs =      37
Spearman's rho =       0.4445

Test of Ho: stunted_svy and stuntedvpost are independent
    Prob > |t| =       0.0058
```

For peer review only

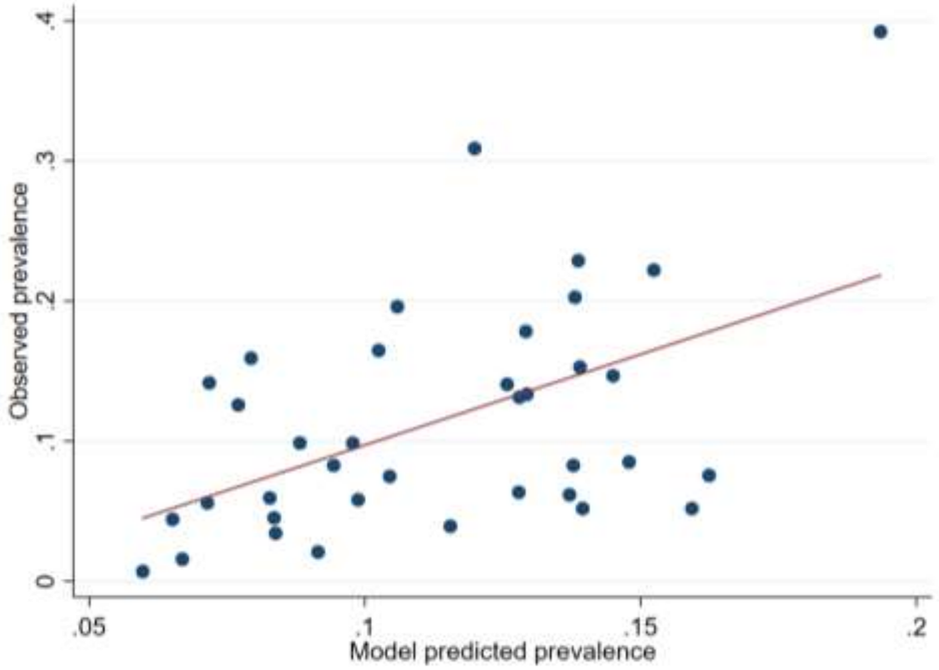


```
. spearman thin_svy thinvpost if validation_sample2==1

 Number of obs =      37
Spearman's rho =       0.2048

Test of Ho: thin_svy and thinvpost are independent
    Prob > |t| =       0.2239
```

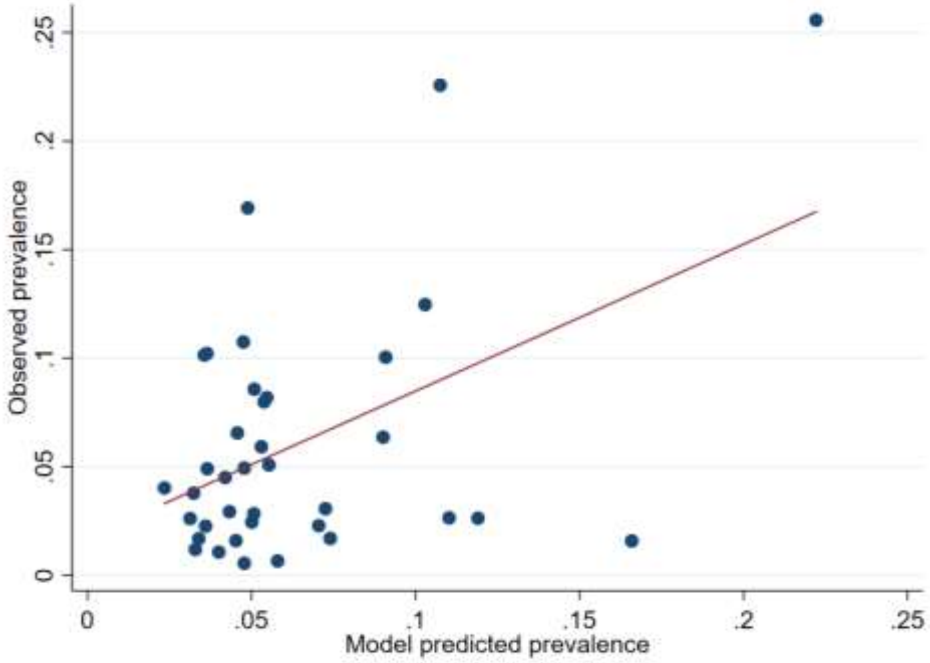


```
. spearman thin_svy thinvpost if validation_sample2==1 & thin_svy~=0

 Number of obs =      37
Spearman's rho =       0.2048

Test of Ho: thin_svy and thinvpost are independent
    Prob > |t| =       0.2239
```

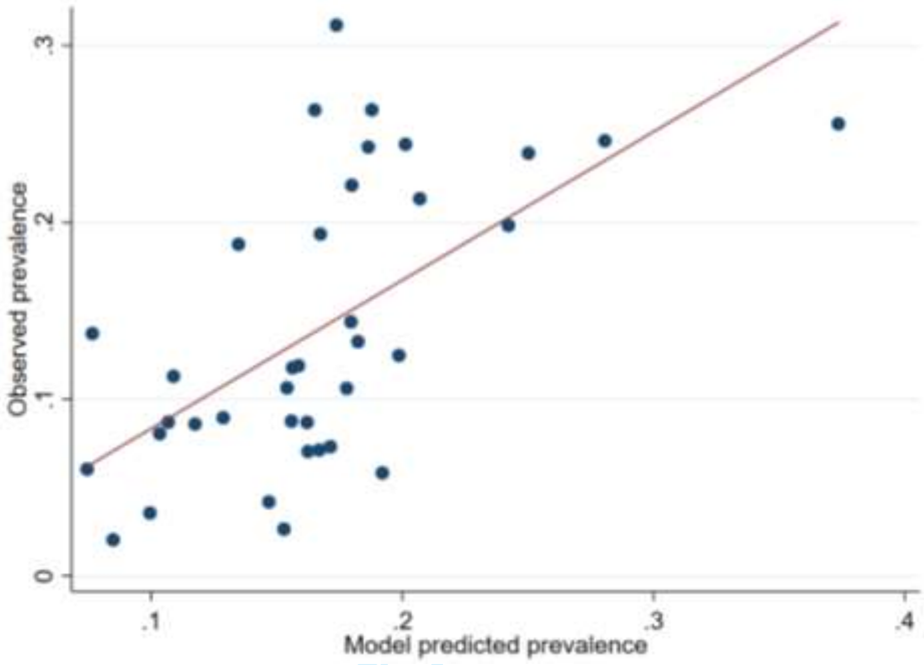

For peer review only

Supplementary 7: Description of the study sample across survey rounds

Survey wave	Age (in years)	Sampled	Estimated population size using survey weights	95% CI		% sampled with height/weight measurement
2008	0	661	1092027	948199	1235854	35.9%
	1	661	1151665	1009086	1294244	67.9%
	2	670	1088458	960285	1216632	71.0%
	3	642	1034244	902011	1166477	81.0%
	4	620	1016227	882185	1150270	83.5%
	<5	3254	5382621	5005478	5759764	
2010/11	0	517	866786	720440	1013132	16.2%
	1	621	1032184	840129	1224239	42.5%
	2	751	1225419	1040085	1410753	49.3%
	3	840	1206389	1026681	1386097	53.3%
	4	820	1196800	1031500	1362101	53.3%
	<5	3549	5527578	4914106	6141050	
2012	0	652	902357	777704	1027010	45.1%
	1	691	1039354	887868	1190839	87.7%
	2	764	1183609	995508	1371711	87.6%
	3	826	1257820	1036042	1479598	89.6%
	4	909	1405034	1191438	1618631	87.3%
	<5	3842	5788174	5112765	6463583	
2014/15	0	886	1185863	1003941	1367786	50.3%
	1	875	1162949	985828	1340070	92.9%
	2	863	1060232	901257	1219207	92.7%
	3	914	1160946	985127	1336765	94.0%
	4	960	1298110	1098342	1497879	94.3%
	<5	4498	5868101	5200170	6536031	
2017	0	813	987763	841487	1134040	47.8%
	1	909	1215360	1045099	1385622	86.4%
	2	996	1293408	1105038	1481779	84.6%
	3	992	1264427	1088783	1440071	88.9%
	4	1000	1129184	973431	1284937	90.4%
	<5	4710	5890142	5261158	6519126	

For peer review only

Supplementary 8: Sensitivity analyses for missing weight and height

Summary: A comparison of missing weight/height proportions by various socio-demographic variables suggests that many were likely missing at random. Distributions of race, gender, household income, low birthweight, food security status, mother education category and father education category were not significantly different when comparing children with missing weight/height measurements to those with a valid weight/height measurement (please see analysis output below). However, age did significantly differ by missing status in that infants (<1 year of age) were significantly more likely to have a missing weight/height measurement compared to children aged 1-4 years. There also appeared to be significant differences in missing weight/height status by province of residence i.e. children in Mpumalanga, Western Cape fir example had higher proportions of missing weight/height measurements among children under 5 (p<0.001). Furthermore, missing weight/height measurements for children were more significantly more likely among those children with younger mothers (<25 years of age).

```
. svy: tab race_ missing_height_weight if race_~=0, row ci
(running tabulate on estimation sample)

Number of strata   =        53                  Number of obs     =      16,649
Number of PSUs     =     1,076                  Population size   =  25,331,414
                                                Design df         =       1,023

-------------------------------------------------------
          |            missing_height_weight
    race_ |             0              1          Total
----------+--------------------------------------------
  African |         .8129          .1871              1
          | [.8006,.8246]  [.1754,.1994]
          |
 Coloured |         .7803          .2197              1
          | [.7437,.8129]  [.1871,.2563]
          |
 Asian/In |         .7593          .2407              1
          |  [.5708,.882]   [.118,.4292]
          |
    White |           .74            .26              1
          |  [.643,.8182]   [.1818,.357]
          |
    Total |         .8066          .1934              1
          | [.7945,.8181]  [.1819,.2055]
-------------------------------------------------------
  Key:  row proportion
        [95% confidence interval for row proportion]

  Pearson:
    Uncorrected   chi2(3)         =   32.5162
    Design-based  F(2.49, 2551.53)=    1.7810     P = 0.1588

. svy: tab gender_ missing_height_weight if race_~=0, row ci
(running tabulate on estimation sample)

Number of strata   =        53                  Number of obs     =      19,138
Number of PSUs     =     1,218                  Population size   =  28,354,881
                                                Design df         =       1,165

-------------------------------------------------------
          |            missing_height_weight
  gender_ |             0              1          Total
----------+--------------------------------------------
     Male |         .8065          .1935              1
          | [.7926,.8196]  [.1804,.2074]
          |
   Female |         .8102          .1898              1
          | [.7951,.8245]  [.1755,.2049]
          |
    Total |         .8083          .1917              1
          |  [.7972,.819]   [.181,.2028]
-------------------------------------------------------
  Key:  row proportion
        [95% confidence interval for row proportion]

  Pearson:
    Uncorrected   chi2(1)         =    0.4400
    Design-based  F(1, 1165)      =    0.1697     P = 0.6805
```

For peer review only

```
. svy: tab age_ missing_height_weight, row ci
(running tabulate on estimation sample)

Number of strata   =        53                  Number of obs     =     19,201
Number of PSUs     =     1,227                  Population size   =  28,456,616
                                                Design df         =      1,174

-------------------------------------------------------
          |            missing_height_weight
     age_ |              0               1         Total
----------+--------------------------------------------
        0 |          .4596           .5404             1
          | [.4362,.4832]  [.5168,.5638]
          |
        1 |          .8581           .1419             1
          | [.8308,.8816]  [.1184,.1692]
          |
        2 |          .8764           .1236             1
          | [.8573,.8933]  [.1067,.1427]
          |
        3 |          .8952           .1048             1
          | [.8726,.9142]  [.0858,.1274]
          |
        4 |          .9015           .0985             1
          |  [.8847,.916]   [.084,.1153]
          |
    Total |          .8083           .1917             1
          | [.7972,.8189]  [.1811,.2028]
-------------------------------------------------------
  Key:  row proportion
        [95% confidence interval for row proportion]

  Pearson:
    Uncorrected   chi2(4)         = 3267.7805
    Design-based  F(3.41, 3999.27)=  238.9174     P = 0.0000

. svy: tab hh_inc missing_height_weight, row ci
(running tabulate on estimation sample)

Number of strata   =        53                  Number of obs     =     18,289
Number of PSUs     =     1,195                  Population size   =  26,887,499
                                                Design df         =      1,142

-------------------------------------------------------
          |            missing_height_weight
   hh_inc |              0               1         Total
----------+--------------------------------------------
        1 |          .8032           .1968             1
          | [.7792,.8251]  [.1749,.2208]
          |
        2 |          .8286           .1714             1
          |  [.8012,.853]   [.147,.1988]
          |
        3 |          .8289           .1711             1
          | [.8084,.8475]  [.1525,.1916]
          |
        4 |          .8076           .1924             1
          | [.7751,.8365]  [.1635,.2249]
          |
        5 |          .7862           .2138             1
          |  [.7578,.812]   [.188,.2422]
          |
    Total |          .8096           .1904             1
          | [.7982,.8205]  [.1795,.2018]
-------------------------------------------------------
  Key:  row proportion
        [95% confidence interval for row proportion]

  Pearson:
    Uncorrected   chi2(4)         =   32.2620
    Design-based  F(3.67, 4186.36)=    1.9756     P = 0.1017

. svy: tab province missing_height_weight, row ci
(running tabulate on estimation sample)

Number of strata   =        53                  Number of obs     =     19,201
Number of PSUs     =     1,227                  Population size   =  28,456,616
```

For peer review only

```
                                                 Design df         =      1,174

-------------------------------------------------------
          |            missing_height_weight
 province |              0               1         Total
----------+--------------------------------------------
  Eastern |          .8421           .1579             1
          |  [.819,.8627]   [.1373,.181]
          |
 Free Sta |           .833            .167             1
          | [.7968,.8638]  [.1362,.2032]
          |
  Gauteng |          .7866           .2134             1
          | [.7637,.8078]  [.1922,.2363]
          |
 KwaZulu- |          .8448           .1552             1
          | [.8255,.8624]  [.1376,.1745]
          |
  Limpopo |          .8422           .1578             1
          | [.8184,.8634]  [.1366,.1816]
          |
 Mpumalan |          .7557           .2443             1
          | [.7187,.7892]  [.2108,.2813]
          |
 North We |          .8011           .1989             1
          |  [.7725,.827]   [.173,.2275]
          |
 Northern |          .7921           .2079             1
          | [.7674,.8149]  [.1851,.2326]
          |
  Western |          .7422           .2578             1
          |  [.7064,.775]   [.225,.2936]
          |
    Total |          .8083           .1917             1
          | [.7972,.8189]  [.1811,.2028]
-------------------------------------------------------
  Key:  row proportion
        [95% confidence interval for row proportion]

  Pearson:
    Uncorrected   chi2(8)         =  171.9467
    Design-based  F(6.89, 8090.45)=    9.8218     P = 0.0000

. svy: tab LBW missing_height_weight, row ci
(running tabulate on estimation sample)

Number of strata   =        53                  Number of obs     =     16,606
Number of PSUs     =     1,128                  Population size   = 24,829,511
                                                Design df         =      1,075

-------------------------------------------------------
          |            missing_height_weight
      LBW |              0               1         Total
----------+--------------------------------------------
        0 |          .8164           .1836             1
          | [.8044,.8278]  [.1722,.1956]
          |
        1 |          .8106           .1894             1
          | [.7788,.8388]  [.1612,.2212]
          |
    Total |          .8158           .1842             1
          | [.8045,.8266]  [.1734,.1955]
-------------------------------------------------------
  Key:  row proportion
        [95% confidence interval for row proportion]

  Pearson:
    Uncorrected   chi2(1)         =    0.3369
    Design-based  F(1, 1075)      =    0.1307     P = 0.7178

. svy: tab foodsecurity_proxy missing_height_weight, row ci
(running tabulate on estimation sample)

Number of strata   =        53                   Number of obs     =      5,017
Number of PSUs     =       438                   Population size   =  8,843,019
                                                 Design df         =        385
```

For peer review only

```
-------------------------------------------------------
foodsecur |            missing_height_weight
ity_proxy |              0               1          Total
----------+--------------------------------------------
        1 |          .7719           .2281              1
          | [.7467,.7952]   [.2048,.2533]
          |
        2 |          .8019           .1981              1
          | [.7352,.8551]   [.1449,.2648]
          |
        3 |          .7596           .2404              1
          | [.6983,.8119]   [.1881,.3017]
          |
        4 |          .8284           .1716              1
          | [.7561,.8825]   [.1175,.2439]
          |
        5 |          .7869           .2131              1
          | [.6923,.8583]   [.1417,.3077]
          |
    Total |          .7751           .2249              1
          |  [.753,.7959]    [.2041,.247]
-------------------------------------------------------
  Key:  row proportion
        [95% confidence interval for row proportion]

  Pearson:
    Uncorrected   chi2(4)         =    6.4682
    Design-based  F(3.04, 1168.67)=    0.8267     P = 0.4803

. svy: tab mthagegrp missing_height_weight, row ci
(running tabulate on estimation sample)

Number of strata   =        53                  Number of obs     =     17,335
Number of PSUs     =     1,192                  Population size   =  26,432,345
                                                Design df         =      1,139

-------------------------------------------------------
RECODE of |
mth_age_f |            missing_height_weight
inal      |              0               1          Total
----------+--------------------------------------------
        1 |          .6691           .3309              1
          |  [.628,.7077]    [.2923,.372]
          |
        2 |          .7787           .2213              1
          | [.7553,.8004]   [.1996,.2447]
          |
        3 |          .8128           .1872              1
          | [.7961,.8285]   [.1715,.2039]
          |
        4 |          .8329           .1671              1
          | [.8096,.8539]   [.1461,.1904]
          |
        5 |          .8629           .1371              1
          | [.7939,.9113]   [.0887,.2061]
          |
    Total |          .8006           .1994              1
          | [.7894,.8115]   [.1885,.2106]
-------------------------------------------------------
  Key:  row proportion
        [95% confidence interval for row proportion]

  Pearson:
    Uncorrected   chi2(4)         =  169.4906
    Design-based  F(3.89, 4436.22)=   15.6564     P = 0.0000

. svy: tab mth_edu2 missing_height_weight, row ci
(running tabulate on estimation sample)

Number of strata   =        53                  Number of obs     =     16,352
Number of PSUs     =     1,169                  Population size   =  25,254,660
                                                Design df         =      1,116

-------------------------------------------------------
          |            missing_height_weight
 mth_edu2 |              0               1          Total
----------+--------------------------------------------
```

For peer review only

```
        0 |       .8043        .1957            1
          | [.7537,.8466]  [.1534,.2463]
          |
        1 |       .7848        .2152            1
          | [.7529,.8136]  [.1864,.2471]
          |
        2 |       .7976        .2024            1
          | [.7848,.8098]  [.1902,.2152]
          |
        3 |       .8122        .1878            1
          | [.7734,.8457]  [.1543,.2266]
          |
    Total |       .7982        .2018            1
          | [.7868,.8092]  [.1908,.2132]
-------------------------------------------------------
  Key:  row proportion
        [95% confidence interval for row proportion]

  Pearson:
    Uncorrected   chi2(3)         =    3.7648
    Design-based  F(2.41, 2688.60)=    0.5454     P = 0.6124

. svy: tab fth_educat missing_height_weight, row ci
(running tabulate on estimation sample)

Number of strata   =        53                Number of obs     =      4,574
Number of PSUs     =       755                Population size   =  8,485,206
                                              Design df         =        702

-------------------------------------------------------
fth_educa |           missing_height_weight
t         |             0             1         Total
----------+--------------------------------------------
        0 |       .9417        .0583            1
          |  [.732,.9896]   [.0104,.268]
          |
        1 |       .7923        .2077            1
          | [.7634,.8185]  [.1815,.2366]
          |
        2 |        .803         .197            1
          |  [.759,.8406]   [.1594,.241]
          |
        3 |       .7618        .2382            1
          | [.6661,.8368]  [.1632,.3339]
          |
    Total |       .7948        .2052            1
          | [.7719,.8159]  [.1841,.2281]
-------------------------------------------------------
  Key:  row proportion
        [95% confidence interval for row proportion]

  Pearson:
    Uncorrected   chi2(3)         =    3.8826
    Design-based  F(2.17, 1522.03)=    0.5250     P = 0.6062
```

For peer review only

Supplementary 9: Full posterior prevalence estimates with 95% Bayesian uncertainty intervals (UIs) by district and year. Also includes exceedance probabilities for 17% reduction in stunting from wave 3 (2012) to wave 5 (2017) - to achieve 40% reduction from 2012 to 2025, 5% target threshold for wasting prevalence and no increase in obesity from wave 3 (2012) to wave 5 (2017) as per 2025 nutritional targets.

Province	District	wave	stunting	95% UI		Exceedance probability 17% reduction from wave 3 to 5	thinness	95% UI		Exceedance probability 5% target threshold	obesity	95% UI		Exceedance probability - no increase from wave 3 to 5
Eastern Cape	Alfred Nzo(DC44)	1	9.2%	4.4%	16.1%	N/A	6.1%	2.3%	12.1%	0.6223	16.9%	9.3%	26.2%	N/A
KwaZulu-Natal	Amajuba(DC25)	1	9.7%	5.1%	15.7%	N/A	5.1%	2.1%	9.8%	0.4572	16.1%	9.8%	24.0%	N/A
Eastern Cape	Amathole(DC12)	1	14.8%	6.4%	27.4%	N/A	12.4%	3.9%	26.8%	0.9399	28.2%	14.4%	46.2%	N/A
North West	Bojanala(DC37)	1	10.2%	4.6%	18.4%	N/A	5.7%	1.9%	12.3%	0.5349	9.7%	4.3%	17.7%	N/A
Eastern Cape	Buffalo City(BUF)	1	19.0%	8.3%	35.1%	N/A	14.2%	4.0%	33.4%	0.9435	28.5%	13.0%	48.8%	N/A
Eastern Cape	Cacadu(DC10)	1	21.7%	12.9%	32.5%	N/A	8.0%	2.2%	19.8%	0.7199	18.2%	10.3%	28.0%	N/A
Western Cape	Cape Winelands(DC2)	1	12.5%	4.7%	25.8%	N/A	9.7%	4.4%	17.1%	0.9475	18.7%	10.8%	28.3%	N/A
Limpopo	Capricorn(DC35)	1	12.4%	6.5%	20.6%	N/A	10.1%	4.6%	18.2%	0.9578	12.2%	4.6%	25.1%	N/A
Western Cape	Central Karoo(DC5)	1	16.0%	9.0%	24.9%	N/A	7.6%	3.2%	14.3%	0.8194	13.9%	7.6%	22.2%	N/A
Eastern Cape	Chris Hani(DC13)	1	9.7%	4.7%	17.0%	N/A	7.4%	2.1%	18.3%	0.6688	27.5%	17.5%	38.9%	N/A
Western Cape	City of Cape Town(CPT)	1	8.1%	4.0%	13.8%	N/A	9.0%	2.4%	21.6%	0.7817	15.6%	8.9%	24.0%	N/A
Gauteng	City of Johannesburg(JHB)	1	9.6%	4.8%	15.9%	N/A	4.6%	1.6%	9.4%	0.3591	16.3%	9.5%	24.7%	N/A
Gauteng	City of Tshwane(TSH)	1	18.3%	11.1%	27.2%	N/A	12.8%	6.5%	20.8%	0.9967	9.5%	4.9%	15.8%	N/A
North West	Dr Kenneth Kaunda(DC40)	1	13.4%	6.5%	23.0%	N/A	13.4%	5.8%	24.4%	0.9898	14.7%	7.2%	25.0%	N/A
North West	Dr Ruth Segomotsi Mompati(DC39)	1	11.2%	6.4%	17.5%	N/A	13.5%	7.5%	20.9%	0.9997	10.0%	5.5%	15.9%	N/A
Western Cape	Eden(DC4)	1	13.7%	6.8%	23.5%	N/A	9.8%	2.5%	25.0%	0.81	21.8%	12.0%	34.2%	N/A
Mpumalanga	Ehlanzeni(DC32)	1	10.7%	5.7%	17.3%	N/A	4.0%	1.5%	8.0%	0.2425	8.9%	4.6%	14.9%	N/A
Gauteng	Ekurhuleni(EKU)	1	13.2%	6.5%	22.0%	N/A	5.5%	1.9%	11.3%	0.5078	9.0%	4.0%	16.2%	N/A
Free State	Fezile Dabi(DC20)	1	12.7%	5.8%	23.0%	N/A	7.6%	2.0%	19.2%	0.692	25.6%	13.8%	40.1%	N/A
Northern Cape	Frances Baard(DC9)	1	13.2%	7.4%	20.7%	N/A	5.6%	2.3%	10.6%	0.5542	8.9%	4.5%	15.1%	N/A
Mpumalanga	Gert Sibande(DC30)	1	7.6%	3.8%	13.0%	N/A	4.1%	1.5%	8.2%	0.2558	11.5%	6.4%	18.1%	N/A
Limpopo	Greater Sekhukhune(DC47)	1	14.6%	8.1%	22.9%	N/A	7.9%	3.5%	14.3%	0.8598	7.3%	3.4%	12.9%	N/A
Eastern Cape	Joe Gqabi(DC14)	1	12.8%	6.8%	21.0%	N/A	4.6%	1.7%	9.6%	0.3627	18.6%	10.9%	28.4%	N/A
Northern Cape	John Taolo Gaetsewe(DC45)	1	8.4%	3.7%	15.3%	N/A	6.0%	2.1%	12.5%	0.5885	11.5%	5.6%	20.0%	N/A
Free State	Lejweleputswa(DC18)	1	11.1%	5.0%	19.7%	N/A	7.0%	2.5%	14.6%	0.714	9.9%	4.4%	17.8%	N/A
Free State	Mangaung(MAN)	1	35.1%	21.0%	51.4%	N/A	7.6%	2.0%	19.6%	0.6713	16.5%	6.3%	33.5%	N/A
Limpopo	Mopani(DC33)	1	8.2%	3.8%	14.5%	N/A	5.6%	2.1%	11.2%	0.5388	9.6%	4.7%	16.6%	N/A
Northern Cape	Namakwa(DC6)	1	14.6%	7.6%	24.1%	N/A	7.4%	2.8%	14.6%	0.7848	10.5%	4.9%	18.1%	N/A
Eastern Cape	Nelson Mandela Bay(NMA)	1	11.2%	5.6%	18.9%	N/A	5.5%	2.0%	11.2%	0.5236	25.3%	15.5%	37.0%	N/A
North West	Ngaka Modiri Molema(DC38)	1	11.1%	5.8%	18.2%	N/A	5.7%	2.2%	11.0%	0.5684	18.1%	10.6%	27.3%	N/A
Mpumalanga	Nkangala(DC31)	1	13.3%	7.3%	21.1%	N/A	11.7%	5.7%	20.0%	0.9902	15.4%	8.6%	23.7%	N/A
Eastern Cape	O.R.Tambo(DC15)	1	19.5%	12.6%	27.8%	N/A	3.5%	1.3%	7.0%	0.1417	24.4%	16.5%	33.4%	N/A
Western Cape	Overberg(DC3)	1	11.5%	5.8%	19.4%	N/A	5.4%	1.9%	10.9%	0.4959	17.7%	9.9%	27.8%	N/A
Northern Cape	Pixley ka Seme(DC7)	1	25.0%	13.7%	39.7%	N/A	8.3%	2.3%	20.3%	0.7481	26.1%	14.7%	40.7%	N/A
Gauteng	Sedibeng(DC42)	1	16.5%	9.2%	26.2%	N/A	15.8%	7.9%	26.3%	0.9993	12.3%	6.4%	20.3%	N/A
KwaZulu-Natal	Sisonke(DC43)	1	18.4%	11.3%	26.9%	N/A	8.1%	3.8%	14.5%	0.894	20.6%	13.1%	29.4%	N/A
Northern Cape	Siyanda(DC8)	1	13.3%	7.2%	21.2%	N/A	7.0%	2.7%	13.3%	0.7479	9.1%	4.4%	15.6%	N/A
Free State	Thabo Mofutsanyane(DC19)	1	12.8%	6.2%	22.1%	N/A	7.2%	2.0%	18.0%	0.653	17.5%	9.1%	28.2%	N/A
KwaZulu-Natal	UMgungundlovu(DC22)	1	8.5%	4.1%	14.5%	N/A	4.5%	1.6%	9.1%	0.3328	20.5%	12.6%	30.2%	N/A
KwaZulu-Natal	Ugu(DC21)	1	8.1%	4.4%	13.1%	N/A	2.9%	1.1%	5.8%	0.0602	19.1%	12.6%	26.7%	N/A

For peer review only

KwaZulu-Natal	Umkhanyakude(DC27)	1	11.2%	5.6%	18.9%	N/A	8.3%	3.5%	15.9%	0.8631	13.8%	7.2%	22.5%	N/A
KwaZulu-Natal	Umzinyathi(DC24)	1	12.8%	4.9%	26.1%	N/A	5.5%	1.4%	14.4%	0.4431	19.0%	7.7%	36.5%	N/A
KwaZulu-Natal	Uthukela(DC23)	1	5.6%	3.0%	9.1%	N/A	4.2%	2.0%	7.5%	0.2652	10.7%	6.7%	15.5%	N/A
KwaZulu-Natal	Uthungulu(DC28)	1	11.4%	6.4%	17.8%	N/A	3.2%	1.2%	6.6%	0.1071	19.0%	12.0%	27.4%	N/A
Limpopo	Vhembe(DC34)	1	25.5%	15.4%	38.0%	N/A	4.5%	1.5%	9.9%	0.3466	20.2%	11.3%	31.3%	N/A
Limpopo	Waterberg(DC36)	1	12.0%	6.3%	19.6%	N/A	6.5%	2.7%	12.2%	0.6979	12.1%	6.4%	19.5%	N/A
Western Cape	West Coast(DC1)	1	10.4%	4.6%	18.7%	N/A	8.5%	2.3%	21.0%	0.7495	26.7%	15.6%	40.5%	N/A
Gauteng	West Rand(DC48)	1	11.0%	5.0%	19.9%	N/A	8.9%	3.4%	18.0%	0.8797	11.6%	5.3%	20.5%	N/A
Free State	Xhariep(DC16)	1	20.2%	11.3%	31.3%	N/A	7.1%	2.0%	17.3%	0.6479	10.2%	4.8%	18.0%	N/A
KwaZulu-Natal	Zululand(DC26)	1	8.2%	4.0%	14.0%	N/A	3.6%	1.3%	7.5%	0.1747	13.9%	7.9%	21.8%	N/A
KwaZulu-Natal	eThekwini(ETH)	1	8.1%	4.1%	13.7%	N/A	3.5%	1.3%	7.2%	0.15	19.5%	12.3%	28.5%	N/A
KwaZulu-Natal	iLembe(DC29)	1	10.7%	5.7%	17.4%	N/A	5.1%	1.3%	13.2%	0.4016	37.3%	27.2%	48.2%	N/A
Eastern Cape	Alfred Nzo(DC44)	2	16.2%	8.8%	25.8%	N/A	7.3%	1.9%	18.1%	0.659	18.0%	10.0%	28.0%	N/A
KwaZulu-Natal	Amajuba(DC25)	2	7.6%	4.0%	12.5%	N/A	6.6%	3.1%	11.6%	0.7634	11.6%	6.7%	17.7%	N/A
Eastern Cape	Amathole(DC12)	2	15.6%	5.8%	31.2%	N/A	30.8%	14.7%	51.8%	1	27.4%	11.7%	48.8%	N/A
North West	Bojanala(DC37)	2	10.8%	5.4%	18.4%	N/A	9.0%	2.4%	22.1%	0.7872	15.6%	8.6%	24.9%	N/A
Eastern Cape	Buffalo City(BUF)	2	16.2%	6.0%	33.4%	N/A	14.4%	3.0%	40.0%	0.8966	34.4%	14.5%	60.0%	N/A
Eastern Cape	Cacadu(DC10)	2	17.3%	9.9%	26.7%	N/A	9.8%	2.7%	24.0%	0.8257	9.8%	4.7%	16.9%	N/A
Western Cape	Cape Winelands(DC2)	2	10.7%	5.5%	17.7%	N/A	16.9%	9.5%	26.4%	0.9999	24.4%	15.6%	34.5%	N/A
Limpopo	Capricorn(DC35)	2	17.8%	10.2%	27.3%	N/A	8.5%	3.8%	15.5%	0.8982	8.8%	4.1%	15.5%	N/A
Western Cape	Central Karoo(DC5)	2	11.2%	5.7%	18.5%	N/A	10.1%	2.7%	24.5%	0.842	17.2%	6.6%	33.5%	N/A
Eastern Cape	Chris Hani(DC13)	2	10.9%	5.7%	17.9%	N/A	9.0%	2.4%	22.0%	0.7881	24.0%	15.3%	34.0%	N/A
Western Cape	City of Cape Town(CPT)	2	18.1%	11.2%	26.3%	N/A	14.7%	8.3%	22.7%	0.9998	37.5%	27.7%	48.0%	N/A
Gauteng	City of Johannesburg(JHB)	2	15.6%	9.5%	23.2%	N/A	9.9%	2.7%	24.2%	0.8305	14.9%	8.9%	22.3%	N/A
Gauteng	City of Tshwane(TSH)	2	17.3%	10.7%	25.4%	N/A	9.2%	2.5%	22.2%	0.8056	19.5%	12.3%	28.0%	N/A
North West	Dr Kenneth Kaunda(DC40)	2	24.5%	13.9%	37.9%	N/A	10.1%	2.7%	24.2%	0.8362	16.1%	8.3%	26.7%	N/A
North West	Dr Ruth Segomotsi Mompati(DC39)	2	17.0%	10.5%	24.8%	N/A	12.4%	6.7%	19.7%	0.9974	13.1%	7.4%	20.2%	N/A
Western Cape	Eden(DC4)	2	22.8%	12.1%	36.7%	N/A	11.8%	3.1%	29.0%	0.8861	20.1%	8.1%	38.6%	N/A
Mpumalanga	Ehlanzeni(DC32)	2	10.6%	5.8%	17.0%	N/A	6.0%	2.6%	11.1%	0.6463	13.3%	7.6%	20.3%	N/A
Gauteng	Ekurhuleni(EKU)	2	10.8%	5.5%	18.2%	N/A	9.4%	2.5%	23.1%	0.8025	21.1%	12.8%	31.3%	N/A
Free State	Fezile Dabi(DC20)	2	14.9%	5.7%	29.8%	N/A	9.4%	2.5%	23.3%	0.8069	18.1%	9.0%	30.2%	N/A
Northern Cape	Frances Baard(DC9)	2	14.2%	8.1%	21.9%	N/A	4.7%	1.8%	9.3%	0.3905	10.3%	5.5%	16.7%	N/A
Mpumalanga	Gert Sibande(DC30)	2	14.2%	8.6%	21.0%	N/A	8.6%	2.3%	21.1%	0.769	21.4%	14.3%	29.5%	N/A
Limpopo	Greater Sekhukhune(DC47)	2	18.0%	11.2%	26.0%	N/A	5.8%	2.5%	10.8%	0.6066	9.4%	5.0%	15.1%	N/A
Eastern Cape	Joe Gqabi(DC14)	2	10.5%	5.3%	17.6%	N/A	7.6%	2.0%	19.1%	0.6798	20.0%	12.1%	29.7%	N/A
Northern Cape	John Taolo Gaetsewe(DC45)	2	9.1%	4.0%	16.7%	N/A	14.7%	6.6%	25.7%	0.9957	11.6%	5.6%	20.3%	N/A
Free State	Lejweleputswa(DC18)	2	19.4%	11.1%	29.9%	N/A	13.0%	6.2%	22.4%	0.9947	15.9%	6.3%	31.0%	N/A
Free State	Mangaung(MAN)	2	15.6%	7.4%	26.6%	N/A	9.4%	2.4%	23.5%	0.7897	19.7%	7.8%	38.7%	N/A
Limpopo	Mopani(DC33)	2	12.2%	6.3%	19.9%	N/A	6.8%	2.7%	13.0%	0.7201	20.5%	12.2%	30.6%	N/A
Northern Cape	Namakwa(DC6)	2	14.7%	5.7%	29.1%	N/A	10.7%	3.0%	26.0%	0.8658	15.5%	5.9%	30.8%	N/A
Eastern Cape	Nelson Mandela Bay(NMA)	2	11.4%	6.0%	18.3%	N/A	6.0%	2.4%	11.6%	0.6202	17.6%	10.5%	26.6%	N/A
North West	Ngaka Modiri Molema(DC38)	2	19.0%	11.9%	27.9%	N/A	11.7%	6.0%	19.3%	0.9936	9.6%	4.9%	15.9%	N/A
Mpumalanga	Nkangala(DC31)	2	9.7%	4.8%	16.3%	N/A	9.3%	2.6%	22.1%	0.8075	17.3%	7.1%	33.3%	N/A
Eastern Cape	O.R.Tambo(DC15)	2	24.8%	16.8%	33.8%	N/A	4.4%	1.7%	8.6%	0.3192	31.4%	22.3%	41.3%	N/A
Western Cape	Overberg(DC3)	2	14.1%	5.2%	28.3%	N/A	8.8%	3.7%	16.3%	0.9011	25.0%	15.7%	35.8%	N/A
Northern Cape	Pixley ka Seme(DC7)	2	15.3%	5.9%	30.5%	N/A	10.1%	2.7%	24.1%	0.8434	19.0%	7.6%	35.7%	N/A
Gauteng	Sedibeng(DC42)	2	14.7%	8.4%	22.9%	N/A	9.6%	4.5%	16.9%	0.9546	14.6%	8.3%	22.5%	N/A
KwaZulu-Natal	Sisonke(DC43)	2	20.0%	12.9%	28.6%	N/A	7.5%	1.9%	18.9%	0.6726	50.2%	39.8%	60.8%	N/A

For peer review only

Northern Cape	Siyanda(DC8)	2	10.5%	5.2%	17.5%	N/A	26.1%	16.1%	38.3%	1	8.6%	4.1%	15.0%	N/A
Free State	Thabo Mofutsanyane(DC19)	2	16.1%	8.2%	27.1%	N/A	13.7%	6.0%	25.5%	0.9908	27.0%	15.7%	41.1%	N/A
KwaZulu-Natal	UMgungundlovu(DC22)	2	19.4%	12.3%	28.3%	N/A	6.8%	1.8%	17.3%	0.6059	16.6%	9.9%	24.6%	N/A
KwaZulu-Natal	Ugu(DC21)	2	17.2%	11.3%	23.9%	N/A	5.0%	2.3%	8.8%	0.4501	36.3%	28.2%	44.7%	N/A
KwaZulu-Natal	Umkhanyakude(DC27)	2	18.3%	9.6%	29.7%	N/A	7.1%	1.7%	18.6%	0.6238	30.8%	18.6%	44.7%	N/A
KwaZulu-Natal	Umzinyathi(DC24)	2	14.5%	5.5%	29.2%	N/A	6.6%	1.7%	17.0%	0.5943	22.6%	9.3%	42.2%	N/A
KwaZulu-Natal	Uthukela(DC23)	2	11.1%	7.3%	15.6%	N/A	3.0%	1.3%	5.5%	0.0477	25.4%	19.6%	31.7%	N/A
KwaZulu-Natal	Uthungulu(DC28)	2	16.0%	9.5%	24.2%	N/A	6.0%	2.5%	11.2%	0.6329	28.7%	19.9%	38.6%	N/A
Limpopo	Vhembe(DC34)	2	31.6%	20.1%	44.8%	N/A	7.2%	1.8%	19.1%	0.6245	30.0%	18.9%	43.1%	N/A
Limpopo	Waterberg(DC36)	2	18.2%	11.1%	26.6%	N/A	7.1%	3.1%	12.8%	0.7899	14.8%	8.6%	22.5%	N/A
Western Cape	West Coast(DC1)	2	13.8%	5.0%	28.6%	N/A	10.3%	2.6%	25.9%	0.8387	20.7%	8.2%	39.2%	N/A
Gauteng	West Rand(DC48)	2	11.1%	5.4%	19.3%	N/A	7.1%	2.7%	14.2%	0.7375	22.4%	13.0%	33.8%	N/A
Free State	Xhariep(DC16)	2	15.7%	6.0%	31.4%	N/A	8.7%	2.4%	21.7%	0.7647	24.7%	14.4%	37.2%	N/A
KwaZulu-Natal	Zululand(DC26)	2	11.4%	6.0%	18.4%	N/A	6.1%	2.5%	11.9%	0.6421	12.7%	6.8%	20.2%	N/A
KwaZulu-Natal	eThekwini(ETH)	2	15.5%	9.6%	22.6%	N/A	4.9%	2.1%	9.2%	0.4327	32.5%	23.8%	42.1%	N/A
KwaZulu-Natal	iLembe(DC29)	2	8.9%	4.4%	14.9%	N/A	5.2%	2.1%	10.3%	0.4818	29.4%	20.1%	39.7%	N/A
Eastern Cape	Alfred Nzo(DC44)	3	17.0%	9.7%	26.5%	N/A	6.2%	2.5%	12.3%	0.6337	25.0%	15.7%	36.2%	N/A
KwaZulu-Natal	Amajuba(DC25)	3	12.2%	6.8%	19.4%	N/A	4.5%	1.8%	8.8%	0.341	14.3%	8.3%	21.9%	N/A
Eastern Cape	Amathole(DC12)	3	12.1%	5.4%	22.2%	N/A	10.1%	3.5%	20.9%	0.901	17.1%	7.8%	29.7%	N/A
North West	Bojanala(DC37)	3	8.6%	4.1%	14.7%	N/A	5.7%	2.2%	11.1%	0.5831	10.3%	5.2%	17.2%	N/A
Eastern Cape	Buffalo City(BUF)	3	14.3%	5.2%	30.4%	N/A	11.2%	2.2%	33.1%	0.7951	39.0%	20.0%	61.1%	N/A
Eastern Cape	Cacadu(DC10)	3	9.8%	4.7%	16.9%	N/A	6.6%	2.6%	13.0%	0.7016	12.5%	6.3%	20.5%	N/A
Western Cape	Cape Winelands(DC2)	3	9.0%	4.3%	15.7%	N/A	5.0%	1.7%	10.3%	0.4359	17.1%	9.6%	26.4%	N/A
Limpopo	Capricorn(DC35)	3	17.1%	10.5%	25.3%	N/A	4.6%	1.8%	8.9%	0.3588	11.2%	6.1%	17.9%	N/A
Western Cape	Central Karoo(DC5)	3	11.6%	6.0%	19.0%	N/A	4.9%	1.8%	10.0%	0.4155	13.8%	7.5%	22.1%	N/A
Eastern Cape	Chris Hani(DC13)	3	12.4%	6.9%	19.6%	N/A	8.9%	4.2%	15.6%	0.9323	22.2%	14.2%	31.4%	N/A
Western Cape	City of Cape Town(CPT)	3	10.1%	5.7%	15.8%	N/A	4.8%	2.0%	9.0%	0.403	23.3%	16.2%	31.5%	N/A
Gauteng	City of Johannesburg(JHB)	3	14.7%	8.9%	21.8%	N/A	10.6%	5.6%	17.3%	0.9889	14.5%	8.6%	21.6%	N/A
Gauteng	City of Tshwane(TSH)	3	9.4%	5.2%	15.1%	N/A	6.1%	2.8%	10.9%	0.675	14.6%	9.0%	21.8%	N/A
North West	Dr Kenneth Kaunda(DC40)	3	15.9%	8.0%	26.5%	N/A	7.6%	2.1%	19.0%	0.6966	17.6%	9.1%	29.4%	N/A
North West	Dr Ruth Segomotsi Mompati(DC39)	3	21.0%	13.3%	30.0%	N/A	4.6%	1.7%	9.1%	0.3583	13.5%	7.7%	21.1%	N/A
Western Cape	Eden(DC4)	3	10.6%	4.6%	19.4%	N/A	20.0%	9.4%	34.8%	0.9997	13.5%	6.2%	23.4%	N/A
Mpumalanga	Ehlanzeni(DC32)	3	26.6%	19.0%	35.2%	N/A	8.6%	4.5%	14.0%	0.9527	23.1%	16.1%	31.0%	N/A
Gauteng	Ekurhuleni(EKU)	3	8.0%	3.9%	13.7%	N/A	4.3%	1.6%	8.8%	0.3087	14.5%	8.2%	22.6%	N/A
Free State	Fezile Dabi(DC20)	3	13.4%	6.3%	23.9%	N/A	7.1%	1.9%	17.8%	0.6345	17.2%	8.2%	29.2%	N/A
Northern Cape	Frances Baard(DC9)	3	9.1%	4.7%	15.3%	N/A	5.8%	2.4%	11.0%	0.6016	10.2%	5.4%	16.6%	N/A
Mpumalanga	Gert Sibande(DC30)	3	12.9%	7.6%	19.4%	N/A	13.3%	7.5%	20.6%	0.9999	22.2%	14.9%	30.6%	N/A
Limpopo	Greater Sekhukhune(DC47)	3	11.0%	6.6%	16.6%	N/A	4.1%	1.8%	7.6%	0.2448	14.2%	8.9%	20.8%	N/A
Eastern Cape	Joe Gqabi(DC14)	3	18.8%	11.2%	28.5%	N/A	3.7%	1.3%	7.8%	0.2	29.1%	19.5%	40.1%	N/A
Northern Cape	John Taolo Gaetsewe(DC45)	3	8.8%	4.1%	15.6%	N/A	5.9%	2.1%	11.9%	0.5754	14.4%	7.6%	23.5%	N/A
Free State	Lejweleputswa(DC18)	3	14.8%	7.9%	23.7%	N/A	7.4%	3.1%	13.8%	0.798	12.2%	6.3%	20.1%	N/A
Free State	Mangaung(MAN)	3	17.6%	9.3%	28.5%	N/A	7.0%	1.7%	18.0%	0.6214	15.6%	7.8%	26.0%	N/A
Limpopo	Mopani(DC33)	3	23.9%	14.7%	34.6%	N/A	9.0%	3.9%	16.5%	0.92	26.7%	17.0%	37.8%	N/A
Northern Cape	Namakwa(DC6)	3	13.3%	6.6%	22.3%	N/A	9.8%	4.1%	18.2%	0.9336	11.6%	5.5%	20.2%	N/A
Eastern Cape	Nelson Mandela Bay(NMA)	3	16.1%	9.1%	25.0%	N/A	4.9%	1.7%	10.2%	0.4129	20.3%	11.8%	30.9%	N/A
North West	Ngaka Modiri Molema(DC38)	3	16.0%	9.8%	23.5%	N/A	6.6%	2.9%	11.8%	0.7373	24.9%	16.9%	33.9%	N/A
Mpumalanga	Nkangala(DC31)	3	7.3%	3.3%	13.2%	N/A	5.7%	2.1%	11.3%	0.5696	16.9%	9.5%	26.4%	N/A
Eastern Cape	O.R.Tambo(DC15)	3	13.1%	7.4%	20.3%	N/A	3.2%	1.1%	6.5%	0.1062	30.8%	21.9%	40.7%	N/A

For peer review only

Western Cape	Overberg(DC3)	3	8.8%	4.3%	15.0%	N/A	7.3%	1.9%	18.6%	0.6533	33.8%	23.3%	45.2%	N/A
Northern Cape	Pixley ka Seme(DC7)	3	13.4%	5.0%	26.8%	N/A	15.5%	6.7%	28.7%	0.9958	15.2%	7.3%	26.6%	N/A
Gauteng	Sedibeng(DC42)	3	10.6%	5.6%	17.2%	N/A	5.9%	2.4%	11.0%	0.609	13.2%	7.6%	20.7%	N/A
KwaZulu-Natal	Sisonke(DC43)	3	14.6%	9.2%	21.3%	N/A	9.3%	5.0%	15.0%	0.9734	29.5%	21.7%	38.0%	N/A
Northern Cape	Siyanda(DC8)	3	14.1%	8.2%	21.8%	N/A	13.7%	7.5%	21.7%	0.9988	8.2%	4.1%	13.6%	N/A
Free State	Thabo Mofutsanyane(DC19)	3	17.3%	9.2%	28.2%	N/A	5.2%	1.7%	11.3%	0.4635	14.7%	7.3%	24.4%	N/A
KwaZulu-Natal	UMgungundlovu(DC22)	3	12.9%	7.3%	20.2%	N/A	7.7%	3.5%	13.9%	0.8559	21.6%	13.8%	30.9%	N/A
KwaZulu-Natal	Ugu(DC21)	3	17.6%	11.8%	24.3%	N/A	5.3%	2.5%	9.3%	0.5197	25.8%	18.7%	33.9%	N/A
KwaZulu-Natal	Umkhanyakude(DC27)	3	10.4%	5.6%	16.8%	N/A	3.2%	1.1%	6.7%	0.1092	19.0%	11.9%	27.3%	N/A
KwaZulu-Natal	Umzinyathi(DC24)	3	12.7%	4.9%	25.5%	N/A	5.0%	1.3%	12.9%	0.3875	21.1%	8.4%	39.7%	N/A
KwaZulu-Natal	Uthukela(DC23)	3	13.7%	9.5%	18.4%	N/A	3.4%	1.7%	5.9%	0.091	23.6%	18.2%	29.5%	N/A
KwaZulu-Natal	Uthungulu(DC28)	3	16.1%	9.9%	23.6%	N/A	3.3%	1.2%	6.6%	0.1242	23.7%	16.0%	32.3%	N/A
Limpopo	Vhembe(DC34)	3	7.5%	3.4%	13.7%	N/A	3.4%	1.1%	7.4%	0.1458	9.7%	4.7%	16.4%	N/A
Limpopo	Waterberg(DC36)	3	12.5%	7.3%	19.5%	N/A	6.5%	1.8%	15.9%	0.5857	9.2%	4.9%	15.2%	N/A
Western Cape	West Coast(DC1)	3	7.5%	3.3%	13.6%	N/A	7.7%	3.1%	14.6%	0.8136	19.9%	11.2%	30.5%	N/A
Gauteng	West Rand(DC48)	3	18.7%	10.2%	29.8%	N/A	4.9%	1.6%	10.4%	0.3999	19.3%	10.5%	30.5%	N/A
Free State	Xhariep(DC16)	3	9.7%	4.5%	17.1%	N/A	4.8%	1.6%	10.1%	0.3945	12.3%	6.0%	20.5%	N/A
KwaZulu-Natal	Zululand(DC26)	3	12.8%	7.1%	20.0%	N/A	3.6%	1.3%	7.4%	0.1669	25.9%	17.4%	36.1%	N/A
KwaZulu-Natal	eThekwini(ETH)	3	9.5%	5.7%	14.5%	N/A	4.9%	2.3%	8.6%	0.4256	26.5%	19.6%	33.9%	N/A
KwaZulu-Natal	iLembe(DC29)	3	16.1%	9.0%	25.4%	N/A	4.4%	1.5%	9.4%	0.3189	22.0%	13.0%	32.6%	N/A
Eastern Cape	Alfred Nzo(DC44)	4	14.7%	8.2%	23.1%	N/A	2.8%	0.9%	6.3%	0.0755	13.5%	7.3%	21.5%	N/A
KwaZulu-Natal	Amajuba(DC25)	4	8.0%	4.5%	12.6%	N/A	3.9%	1.0%	10.1%	0.2334	10.7%	6.4%	16.0%	N/A
Eastern Cape	Amathole(DC12)	4	9.3%	3.9%	17.8%	N/A	6.9%	1.6%	17.7%	0.5961	22.2%	11.3%	36.2%	N/A
North West	Bojanala(DC37)	4	8.9%	4.5%	15.0%	N/A	4.9%	1.3%	12.6%	0.3855	8.9%	4.5%	14.9%	N/A
Eastern Cape	Buffalo City(BUF)	4	10.0%	3.9%	19.9%	N/A	8.2%	1.6%	25.1%	0.6461	25.5%	12.5%	42.7%	N/A
Eastern Cape	Cacadu(DC10)	4	9.0%	4.4%	15.7%	N/A	4.2%	1.4%	8.7%	0.2758	26.5%	16.8%	38.0%	N/A
Western Cape	Cape Winelands(DC2)	4	6.2%	3.0%	10.7%	N/A	6.2%	1.6%	15.6%	0.5444	10.6%	5.9%	16.5%	N/A
Limpopo	Capricorn(DC35)	4	9.6%	5.1%	15.5%	N/A	3.4%	1.3%	6.9%	0.1341	11.8%	6.7%	18.5%	N/A
Western Cape	Central Karoo(DC5)	4	8.6%	4.1%	15.1%	N/A	4.6%	1.6%	9.4%	0.3614	7.9%	3.6%	14.2%	N/A
Eastern Cape	Chris Hani(DC13)	4	9.1%	4.7%	14.9%	N/A	3.8%	1.5%	7.6%	0.1996	15.9%	9.5%	23.7%	N/A
Western Cape	City of Cape Town(CPT)	4	8.5%	3.0%	18.8%	N/A	7.0%	3.3%	12.2%	0.8015	15.4%	9.5%	22.5%	N/A
Gauteng	City of Johannesburg(JHB)	4	6.6%	3.5%	10.7%	N/A	8.4%	4.6%	13.4%	0.9533	8.0%	4.5%	12.6%	N/A
Gauteng	City of Tshwane(TSH)	4	4.5%	2.1%	7.9%	N/A	3.7%	1.5%	7.0%	0.1582	6.0%	3.1%	10.1%	N/A
North West	Dr Kenneth Kaunda(DC40)	4	6.9%	2.8%	13.1%	N/A	5.4%	1.8%	11.6%	0.4939	8.0%	3.5%	15.0%	N/A
North West	Dr Ruth Segomotsi Mompati(DC39)	4	8.0%	4.2%	13.3%	N/A	7.0%	3.3%	12.2%	0.8128	5.2%	2.5%	9.2%	N/A
Western Cape	Eden(DC4)	4	9.0%	3.3%	19.0%	N/A	6.6%	1.6%	17.3%	0.5772	13.2%	6.1%	23.0%	N/A
Mpumalanga	Ehlanzeni(DC32)	4	6.6%	3.5%	10.8%	N/A	3.4%	1.4%	6.4%	0.1089	12.5%	7.6%	18.3%	N/A
Gauteng	Ekurhuleni(EKU)	4	4.4%	2.0%	7.8%	N/A	6.5%	3.0%	11.5%	0.7371	9.3%	5.0%	15.0%	N/A
Free State	Fezile Dabi(DC20)	4	6.7%	2.8%	12.8%	N/A	5.2%	1.3%	13.4%	0.4179	21.5%	11.8%	33.9%	N/A
Northern Cape	Frances Baard(DC9)	4	10.9%	6.1%	17.1%	N/A	3.4%	1.3%	7.0%	0.139	7.2%	3.6%	12.2%	N/A
Mpumalanga	Gert Sibande(DC30)	4	12.5%	7.3%	19.1%	N/A	4.7%	2.0%	9.0%	0.3821	13.1%	7.7%	19.9%	N/A
Limpopo	Greater Sekhukhune(DC47)	4	11.9%	7.2%	17.6%	N/A	5.4%	2.5%	9.6%	0.5501	5.8%	3.0%	9.6%	N/A
Eastern Cape	Joe Gqabi(DC14)	4	16.4%	9.4%	25.3%	N/A	3.2%	1.1%	6.9%	0.1167	13.4%	7.3%	21.2%	N/A
Northern Cape	John Taolo Gaetsewe(DC45)	4	9.7%	5.2%	16.0%	N/A	8.4%	3.9%	14.6%	0.9098	6.5%	3.1%	11.4%	N/A
Free State	Lejweleputswa(DC18)	4	13.2%	7.4%	20.8%	N/A	5.4%	2.2%	10.4%	0.5142	9.4%	4.8%	15.4%	N/A
Free State	Mangaung(MAN)	4	8.5%	3.7%	15.8%	N/A	5.4%	1.8%	11.8%	0.4869	15.0%	7.7%	24.9%	N/A
Limpopo	Mopani(DC33)	4	6.9%	3.3%	12.0%	N/A	5.3%	2.1%	10.3%	0.5005	8.7%	4.4%	14.5%	N/A
Northern Cape	Namakwa(DC6)	4	7.6%	3.4%	13.9%	N/A	7.1%	2.8%	13.8%	0.7477	8.7%	4.1%	15.4%	N/A

Eastern Cape	Nelson Mandela Bay(NMA)	4	10.6%	5.5%	17.6%	N/A	4.2%	1.0%	11.9%	0.282	18.9%	11.3%	28.4%	N/A
North West	Ngaka Modiri Molema(DC38)	4	9.4%	5.2%	15.0%	N/A	7.1%	3.4%	12.3%	0.8173	10.9%	6.3%	17.0%	N/A
Mpumalanga	Nkangala(DC31)	4	5.7%	2.6%	10.2%	N/A	5.1%	1.4%	12.8%	0.3983	15.5%	9.0%	23.7%	N/A
Eastern Cape	O.R.Tambo(DC15)	4	8.6%	4.9%	13.3%	N/A	3.8%	0.9%	10.4%	0.2255	24.9%	18.4%	32.3%	N/A
Western Cape	Overberg(DC3)	4	6.3%	2.9%	11.2%	N/A	3.1%	1.0%	6.6%	0.1012	20.5%	12.8%	29.5%	N/A
Northern Cape	Pixley ka Seme(DC7)	4	7.6%	3.2%	14.2%	N/A	4.9%	1.7%	10.6%	0.4143	14.2%	7.0%	24.3%	N/A
Gauteng	Sedibeng(DC42)	4	8.7%	4.4%	14.6%	N/A	3.4%	1.2%	7.1%	0.1509	12.9%	7.2%	20.4%	N/A
KwaZulu-Natal	Sisonke(DC43)	4	7.0%	3.7%	11.5%	N/A	2.5%	0.9%	5.2%	0.0337	10.1%	5.6%	15.7%	N/A
Northern Cape	Siyanda(DC8)	4	9.0%	4.8%	14.9%	N/A	6.8%	3.0%	12.3%	0.761	5.0%	2.2%	9.0%	N/A
Free State	Thabo Mofutsanyane(DC19)	4	6.9%	3.0%	12.8%	N/A	4.8%	1.3%	12.3%	0.3625	11.8%	5.9%	20.0%	N/A
KwaZulu-Natal	UMgungundlovu(DC22)	4	6.4%	3.2%	10.9%	N/A	2.3%	0.8%	4.9%	0.0216	14.8%	9.1%	21.7%	N/A
KwaZulu-Natal	Ugu(DC21)	4	7.3%	4.1%	11.4%	N/A	2.9%	1.2%	5.6%	0.0537	18.0%	12.4%	24.6%	N/A
KwaZulu-Natal	Umkhanyakude(DC27)	4	7.4%	3.6%	12.4%	N/A	3.8%	1.0%	10.2%	0.23	14.1%	8.2%	21.7%	N/A
KwaZulu-Natal	Umzinyathi(DC24)	4	8.8%	3.3%	18.9%	N/A	3.6%	0.9%	9.8%	0.1991	15.2%	5.8%	30.8%	N/A
KwaZulu-Natal	Uthukela(DC23)	4	9.6%	6.4%	13.3%	N/A	5.0%	2.8%	7.8%	0.4454	14.7%	10.8%	19.4%	N/A
KwaZulu-Natal	Uthungulu(DC28)	4	11.4%	6.9%	17.2%	N/A	3.2%	1.3%	6.4%	0.093	18.9%	12.6%	26.1%	N/A
Limpopo	Vhembe(DC34)	4	13.5%	7.8%	20.7%	N/A	2.4%	0.7%	5.2%	0.0304	8.3%	4.2%	13.8%	N/A
Limpopo	Waterberg(DC36)	4	8.2%	4.4%	13.1%	N/A	3.5%	1.4%	6.7%	0.1388	7.3%	3.8%	12.0%	N/A
Western Cape	West Coast(DC1)	4	5.2%	2.2%	9.9%	N/A	4.5%	1.6%	9.5%	0.34	8.2%	3.7%	14.7%	N/A
Gauteng	West Rand(DC48)	4	6.2%	2.6%	11.9%	N/A	4.1%	1.3%	8.9%	0.2656	11.4%	5.4%	19.8%	N/A
Free State	Xhariep(DC16)	4	13.4%	6.9%	22.1%	N/A	4.8%	1.3%	12.4%	0.3584	8.6%	3.9%	15.1%	N/A
KwaZulu-Natal	Zululand(DC26)	4	8.3%	4.5%	13.2%	N/A	2.9%	1.1%	5.9%	0.0707	17.7%	11.5%	25.0%	N/A
KwaZulu-Natal	eThekwini(ETH)	4	12.7%	8.2%	17.9%	N/A	2.0%	0.8%	4.1%	0.0055	29.2%	22.4%	36.5%	N/A
KwaZulu-Natal	iLembe(DC29)	4	7.0%	3.6%	11.6%	N/A	2.8%	1.0%	5.7%	0.0561	15.0%	9.3%	22.0%	N/A
Eastern Cape	Alfred Nzo(DC44)	5	6.6%	2.7%	12.7%	0.958	3.6%	1.1%	8.1%	0.1844	15.1%	7.7%	25.1%	0.0655
KwaZulu-Natal	Amajuba(DC25)	5	8.9%	4.4%	15.0%	0.6443	3.1%	1.1%	6.7%	0.1028	11.1%	5.9%	18.0%	0.2338
Eastern Cape	Amathole(DC12)	5	8.2%	3.1%	17.0%	0.669	6.1%	1.6%	15.4%	0.5297	19.6%	8.5%	35.6%	0.6116
North West	Bojanala(DC37)	5	8.7%	3.9%	16.4%	0.3584	4.6%	1.2%	12.2%	0.3387	11.6%	5.4%	20.5%	0.6028
Eastern Cape	Buffalo City(BUF)	5	9.3%	3.2%	20.6%	0.6684	7.9%	1.4%	25.4%	0.608	22.8%	9.8%	41.2%	0.0726
Eastern Cape	Cacadu(DC10)	5	8.1%	3.5%	15.3%	0.5055	4.9%	1.3%	12.7%	0.3818	9.0%	4.0%	16.5%	0.2162
Western Cape	Cape Winelands(DC2)	5	9.6%	4.4%	17.0%	0.3039	5.8%	2.2%	11.7%	0.5642	11.2%	5.5%	19.0%	0.1291
Limpopo	Capricorn(DC35)	5	11.9%	5.9%	20.2%	0.6993	3.7%	1.2%	8.1%	0.1959	11.8%	5.8%	20.2%	0.5339
Western Cape	Central Karoo(DC5)	5	7.5%	3.2%	14.4%	0.7188	6.4%	2.3%	13.8%	0.6329	9.9%	4.4%	18.2%	0.2008
Eastern Cape	Chris Hani(DC13)	5	6.2%	2.6%	11.8%	0.8805	3.5%	1.1%	7.7%	0.1712	18.5%	10.3%	28.9%	0.2741
Western Cape	City of Cape Town(CPT)	5	8.3%	3.8%	14.8%	0.5419	6.8%	2.7%	13.4%	0.7178	18.5%	10.4%	28.3%	0.2048
Gauteng	City of Johannesburg(JHB)	5	7.6%	3.7%	13.3%	0.8987	4.3%	1.5%	8.7%	0.2993	9.3%	4.7%	15.7%	0.1068
Gauteng	City of Tshwane(TSH)	5	6.6%	3.2%	11.5%	0.6727	3.5%	1.2%	7.1%	0.1517	8.0%	4.0%	13.4%	0.0405
North West	Dr Kenneth Kaunda(DC40)	5	11.8%	5.3%	21.7%	0.6061	5.0%	1.5%	11.5%	0.4061	10.3%	4.5%	19.2%	0.108
North West	Dr Ruth Segomotsi Mompati(DC39)	5	7.2%	3.2%	13.2%	0.9883	6.0%	2.3%	11.9%	0.5997	7.3%	3.3%	13.4%	0.0628
Western Cape	Eden(DC4)	5	6.8%	2.7%	13.4%	0.7027	6.1%	1.4%	15.7%	0.5276	10.9%	4.8%	20.1%	0.3119
Mpumalanga	Ehlanzeni(DC32)	5	7.2%	3.4%	12.7%	0.9998	3.4%	1.2%	7.1%	0.1361	6.9%	3.2%	12.3%	0.0002
Gauteng	Ekurhuleni(EKU)	5	5.6%	2.4%	10.5%	0.6546	5.9%	2.3%	11.7%	0.6005	11.1%	5.7%	18.6%	0.2311
Free State	Fezile Dabi(DC20)	5	8.9%	3.7%	17.5%	0.6807	4.8%	1.3%	12.4%	0.3609	17.0%	8.0%	29.8%	0.4831
Northern Cape	Frances Baard(DC9)	5	9.5%	4.6%	16.6%	0.3076	4.0%	1.4%	8.6%	0.2516	6.6%	3.0%	12.1%	0.1522
Mpumalanga	Gert Sibande(DC30)	5	9.0%	4.3%	15.9%	0.6795	3.4%	1.1%	7.5%	0.1421	13.6%	7.2%	22.2%	0.0581
Limpopo	Greater Sekhukhune(DC47)	5	6.8%	3.0%	12.3%	0.7849	4.7%	1.7%	9.8%	0.3635	5.9%	2.6%	10.8%	0.0085
Eastern Cape	Joe Gqabi(DC14)	5	7.4%	3.0%	14.0%	0.9632	3.2%	0.9%	7.5%	0.1286	17.3%	8.8%	28.8%	0.0514
Northern Cape	John Taolo Gaetsewe(DC45)	5	7.1%	3.0%	13.7%	0.5346	5.5%	1.8%	11.8%	0.4975	8.4%	3.7%	15.7%	0.0982

For peer review only - http://bmjopen.bmj.com/site/about/guidelines.xhtml

Free State	Lejweleputswa(DC18)	5	11.7%	5.7%	20.3%	0.5537	4.1%	1.3%	9.0%	0.2609	10.2%	4.8%	18.0%	0.3243
Free State	Mangaung(MAN)	5	9.8%	4.1%	18.4%	0.8263	4.7%	1.2%	12.4%	0.3582	16.1%	7.5%	28.5%	0.5143
Limpopo	Mopani(DC33)	5	10.0%	4.6%	17.9%	0.9661	5.2%	1.8%	11.2%	0.4653	9.6%	4.4%	17.2%	0.0022
Northern Cape	Namakwa(DC6)	5	8.2%	3.0%	17.9%	0.731	4.9%	1.5%	11.2%	0.4009	8.6%	3.6%	16.5%	0.2484
Eastern Cape	Nelson Mandela Bay(NMA)	5	8.9%	4.0%	16.1%	0.8491	3.2%	0.9%	7.5%	0.1316	17.2%	9.0%	27.6%	0.3095
North West	Ngaka Modiri Molema(DC38)	5	7.0%	3.1%	12.9%	0.95	4.4%	1.5%	9.1%	0.3177	10.1%	4.9%	17.1%	0.0032
Mpumalanga	Nkangala(DC31)	5	8.9%	4.0%	16.6%	0.2224	5.1%	1.8%	11.0%	0.4396	11.1%	5.3%	19.5%	0.1345
Eastern Cape	O.R.Tambo(DC15)	5	10.1%	5.4%	16.4%	0.5924	2.3%	0.7%	5.1%	0.0279	17.9%	11.0%	26.1%	0.0172
Western Cape	Overberg(DC3)	5	9.0%	4.1%	16.1%	0.3381	4.9%	1.2%	12.9%	0.3739	22.0%	12.8%	33.7%	0.0586
Northern Cape	Pixley ka Seme(DC7)	5	9.7%	4.0%	18.7%	0.5992	5.2%	1.4%	13.3%	0.4273	12.3%	4.8%	24.8%	0.311
Gauteng	Sedibeng(DC42)	5	6.4%	2.6%	12.1%	0.7712	6.6%	2.4%	13.3%	0.6657	10.0%	4.6%	17.8%	0.2336
KwaZulu-Natal	Sisonke(DC43)	5	7.7%	3.5%	14.2%	0.887	2.8%	0.8%	6.3%	0.0772	21.4%	12.6%	32.4%	0.1028
Northern Cape	Siyanda(DC8)	5	11.7%	5.9%	19.6%	0.5127	5.2%	1.9%	10.6%	0.4716	6.3%	2.7%	11.8%	0.2676
Free State	Thabo Mofutsanyane(DC19)	5	9.0%	3.7%	17.3%	0.8498	4.4%	1.2%	11.4%	0.3145	12.4%	5.5%	22.6%	0.3373
KwaZulu-Natal	UMgungundlovu(DC22)	5	6.6%	2.9%	12.2%	0.8795	3.8%	1.3%	8.2%	0.2098	17.5%	9.8%	27.3%	0.2441
KwaZulu-Natal	Ugu(DC21)	5	7.6%	3.8%	12.9%	0.9731	3.3%	1.2%	6.7%	0.1131	20.8%	13.3%	29.7%	0.1827
KwaZulu-Natal	Umkhanyakude(DC27)	5	7.8%	3.6%	14.2%	0.6034	4.4%	1.5%	9.7%	0.3244	11.3%	5.5%	19.3%	0.0658
KwaZulu-Natal	Umzinyathi(DC24)	5	6.4%	2.9%	11.5%	0.8143	3.4%	1.2%	7.3%	0.1398	12.9%	6.8%	20.7%	0.1568
KwaZulu-Natal	Uthukela(DC23)	5	7.2%	3.5%	12.5%	0.9174	3.2%	1.1%	6.8%	0.1074	11.4%	6.2%	18.1%	0.0034
KwaZulu-Natal	Uthungulu(DC28)	5	11.1%	5.8%	18.0%	0.7098	3.0%	1.0%	6.5%	0.0927	18.6%	11.3%	27.6%	0.1876
Limpopo	Vhembe(DC34)	5	9.7%	4.5%	17.3%	0.1707	4.3%	1.4%	9.5%	0.3067	9.8%	4.6%	17.2%	0.5039
Limpopo	Waterberg(DC36)	5	6.0%	2.7%	10.9%	0.913	4.1%	1.5%	8.4%	0.2555	10.6%	5.4%	17.8%	0.634
Western Cape	West Coast(DC1)	5	6.6%	2.6%	13.2%	0.4791	4.5%	1.3%	10.6%	0.329	12.7%	5.6%	23.0%	0.1249
Gauteng	West Rand(DC48)	5	6.3%	2.5%	12.9%	0.9742	3.7%	1.1%	8.6%	0.2027	11.4%	5.0%	20.8%	0.0971
Free State	Xhariep(DC16)	5	8.5%	3.6%	16.2%	0.4699	4.4%	1.2%	11.5%	0.3171	10.6%	4.8%	19.2%	0.3661
KwaZulu-Natal	Zululand(DC26)	5	7.6%	3.6%	13.5%	0.7981	2.3%	0.7%	5.2%	0.033	15.9%	8.9%	24.9%	0.0506
KwaZulu-Natal	eThekwini(ETH)	5	6.7%	3.2%	11.5%	0.6792	2.4%	0.8%	5.2%	0.0314	14.7%	8.6%	22.4%	0.0119
KwaZulu-Natal	iLembe(DC29)	5	7.0%	3.2%	12.9%	0.9372	3.2%	0.8%	8.7%	0.1436	18.0%	10.3%	27.9%	0.2679

For peer review only

Supplementary 10: post hoc power analysis

We performed a post hoc power analysis to assess the minimum effect size detectable among infants which has the smallest number of observations. The post hoc power analysis suggests that the sample size in the smallest age group has the power to detect a small effect size (w~0.1 based on Cohens rules of thumb [Cohen, 1988]) when using a chi-square test with 2x9 cells (maximum number of cells tested in our analyses i.e. binary nutritional classification versus province of residence) with 80% power and 5% alpha or type I error.

χ^2 tests - Goodness-of-fit tests: Contingency tables

**Analysis:**	Post hoc: Compute achieved power		
**Input:**	Effect size w	=	0.11
	α err prob	=	0.05
	Total sample size	=	1277
	Df	=	8
**Output:**	Noncentrality parameter λ	=	15.4517000
	Critical χ^2	=	15.5073131
	Power (1-β err prob)	=	0.8133607

Cohen, J (1988) Statistical power analysis for the behavioral sciences (2nd ed.). Hillsdale, NJ: Erlbaum.

A summary guideline for effect size determinations is also provided in Kotrlik, JW and Williams, HA (2003) The incorporation of effect size in information technology, learning, and performance research. *Information Techology, Learning, and Performance Journal* **21(1)** 1-7.

Effect Size	Use	Small	Medium	Large
Correlation inc Phi		0.1	0.3	0.5
Cramer's V	r x c frequency tables	0.1	0.3	0.5
Difference in arcsines	Comparing two proportions	0.2	0.5	0.8
η^2	Anova	0.01	0.06	0.14
omega-squared	Anova; See Field (2013)	0.01	0.06	0.14
Multivariate eta-squared	one-way MANOVA	0.01	0.06	0.14
Cohen's f	one-way an(c)ova (regression)	0.1	0.25	0.4
η^2	Multiple regression	0.02	0.13	0.26
κ^2	Mediation analysis	0.01	0.09	0.25
Cohen's f	Multiple Regression	0.14	0.39	0.59
Cohen's d	t-tests	0.2	0.5	0.8
Cohen's ω	chi-square	0.1	0.3	0.5
Odds Ratios	2 by 2 tables	1.5	3.5	9
Odds Ratios	p vs 0.5	0.55	0.65	0.75
Average Spearman rho	Friedman test	0.1	0.3	0.5

For peer review only

STROBE Statement—Checklist of items that should be included in reports of cross-sectional studies

	Item No	Recommendation	Page/line numbers
**Title and abstract**	1	(*a*) Indicate the study's design with a commonly used term in the title or the abstract	2/30
		(*b*) Provide in the abstract an informative and balanced summary of what was done and what was found	2/28-47
**Introduction**			
Background/rationale	2	Explain the scientific background and rationale for the investigation being reported	3-4/67-84
Objectives	3	State specific objectives, including any prespecified hypotheses	4/82-84
**Methods**			
Study design	4	Present key elements of study design early in the paper	4/89-100
Setting	5	Describe the setting, locations, and relevant dates, including periods of recruitment, exposure, follow-up, and data collection	4/89-100
Participants	6	(*a*) Give the eligibility criteria, and the sources and methods of selection of participants	4/95-102
Variables	7	Clearly define all outcomes, exposures, predictors, potential confounders, and effect modifiers. Give diagnostic criteria, if applicable	4-5/103-117
Data sources/ measurement	8*	For each variable of interest, give sources of data and details of methods of assessment (measurement). Describe comparability of assessment methods if there is more than one group	4/89-93; 5/113-117
Bias	9	Describe any efforts to address potential sources of bias	4/89-91; 8/200-2017
Study size	10	Explain how the study size was arrived at	4/89-91; Supplementary 10
Quantitative variables	11	Explain how quantitative variables were handled in the analyses. If applicable, describe which groupings were chosen and why	4-5/104-110; 5/113-117
Statistical methods	12	(*a*) Describe all statistical methods, including those used to control for confounding	5-7/118-189
		(*b*) Describe any methods used to examine subgroups and interactions	5/113-117; 7/186-189
		(*c*) Explain how missing data were addressed	8/204-207; Supplementary 7 & 8
		(*d*) If applicable, describe analytical methods taking account of sampling strategy	5/120-123; 7/172-174; 7/186-189

For peer review only

		(*e*) Describe any sensitivity analyses	8/204-205; Supplementary 4b, 8
**Results**			
Participants	13*	(a) Report numbers of individuals at each stage of study—eg numbers potentially eligible, examined for eligibility, confirmed eligible, included in the study, completing follow-up, and analysed	8/200-207; Supplementary 7
		(b) Give reasons for non-participation at each stage	8/200-207; Supplementary 7
		(c) Consider use of a flow diagram	Described using narrative text: 8/200-207; Table: Supplementary 7
Descriptive data	14*	(a) Give characteristics of study participants (eg demographic, clinical, social) and information on exposures and potential confounders	Table 1
		(b) Indicate number of participants with missing data for each variable of interest	8/200-207; Table 1; Supplementary 7
Outcome data	15*	Report numbers of outcome events or summary measures	Table 1-3
Main results	16	(*a*) Give unadjusted estimates and, if applicable, confounder-adjusted estimates and their precision (eg, 95% confidence interval). Make clear which confounders were adjusted for and why they were included	8/209-273; Table 1-3
		(*b*) Report category boundaries when continuous variables were categorized	4/104-110
		(*c*) If relevant, consider translating estimates of relative risk into absolute risk for a meaningful time period	N/A
Other analyses	17	Report other analyses done—eg analyses of subgroups and interactions, and sensitivity analyses	Supplementary 2,6,7,8,9,10
**Discussion**			
Key results	18	Summarise key results with reference to study objectives	10-11/275-283
Limitations	19	Discuss limitations of the study, taking into account sources of potential bias or imprecision. Discuss both direction and magnitude of any potential bias	14/377-386
Interpretation	20	Give a cautious overall interpretation of results considering objectives, limitations, multiplicity of analyses, results from similar studies, and other relevant evidence	11-15/296-417
Generalisability	21	Discuss the generalisability (external validity) of the study results	11-13/296-370
**Other information**			
Funding	22	Give the source of funding and the role of the funders for the present study and, if applicable, for the original study on which the present article is based	19/581-583

For peer review only
